# Claudin-12 is not required for blood–brain barrier tight junction function

**DOI:** 10.1186/s12987-019-0150-9

**Published:** 2019-09-12

**Authors:** Mariana Castro Dias, Caroline Coisne, Pascale Baden, Gaby Enzmann, Lillian Garrett, Lore Becker, Sabine M. Hölter, German Mouse Clinic Consortium, Antonio Aguilar-Pimentel, Thure Adler, Dirk H. Busch, Nadine Spielmann, Kristin Moreth, Wolfgang Hans, Oana Amarie, Jochen Graw, Jan Rozman, Ildiko Radc, Frauke Neff, Julia Calzada-Wack, Birgit Rathkolb, Eckhard Wolf, Thomas Klopstock, Wolfgang Wurst, Johannes Beckers, Manuela Östereicher, Gregor Miller, Holger Maier, Claudia Stoeger, Stefanie Leuchtenberger, Valérie Gailus-Durner, Helmut Fuchs, Martin Hrabě de Angelis, Urban Deutsch, Britta Engelhardt

**Affiliations:** 1 Theodor Kocher Institute, University of Bern, Freiestr. 1, 3012 Bern, Switzerland; 2 German Mouse Clinic, Institute of Experimental Genetics, Helmholtz Center Munich, Neuherberg, Germany; 3 Institute of Developmental Genetics, Helmholtz Center Munich, Neuherberg, Germany; 4 Member of German Center for Diabetes Research (DZD), Neuherberg, Germany; 5 Chair of Experimental Genetics, School of Life Science Weihenstephan, Technische Universität München, Freising, Germany

**Keywords:** Claudin-12, Tight junctions, Blood–brain barrier, Experimental autoimmune encephalomyelitis

## Abstract

**Background:**

The blood–brain barrier (BBB) ensures central nervous system (CNS) homeostasis by strictly controlling the passage of molecules and solutes from the bloodstream into the CNS. Complex and continuous tight junctions (TJs) between brain endothelial cells block uncontrolled paracellular diffusion of molecules across the BBB, with claudin-5 being its dominant TJs protein. However, claudin-5 deficient mice still display ultrastructurally normal TJs, suggesting the contribution of other claudins or tight-junction associated proteins in establishing BBB junctional complexes. Expression of claudin-12 at the BBB has been reported, however the exact function and subcellular localization of this atypical claudin remains unknown.

**Methods:**

We created claudin-12-lacZ-knock-in C57BL/6J mice to explore expression of claudin-12 and its role in establishing BBB TJs function during health and neuroinflammation. We furthermore performed a broad standardized phenotypic check-up of the mouse mutant.

**Results:**

Making use of the lacZ reporter allele, we found claudin-12 to be broadly expressed in numerous organs. In the CNS, expression of claudin-12 was detected in many cell types with very low expression in brain endothelium. Claudin-12^lacZ/lacZ^ C57BL/6J mice lacking claudin-12 expression displayed an intact BBB and did not show any signs of BBB dysfunction or aggravated neuroinflammation in an animal model for multiple sclerosis. Determining the precise localization of claudin-12 at the BBB was prohibited by the fact that available anti-claudin-12 antibodies showed comparable detection and staining patterns in tissues from wild-type and claudin-12^lacZ/lacZ^ C57BL/6J mice.

**Conclusions:**

Our present study thus shows that claudin-12 is not essential in establishing or maintaining BBB TJs integrity. Claudin-12 is rather expressed in cells that typically lack TJs suggesting that claudin-12 plays a role other than forming classical TJs. At the same time, in depth phenotypic screening of clinically relevant organ functions of claudin-12^lacZ/lacZ^ C57BL/6J mice suggested the involvement of claudin-12 in some neurological but, more prominently, in cardiovascular functions.

## Introduction

The endothelial blood–brain barrier (BBB) in the central nervous system (CNS) microvessels protects the CNS from changes in the blood, thus ensuring CNS homeostasis, which is a prerequisite for proper function of CNS neurons. The anatomical basis of the BBB is represented by unique intercellular tight junctional (TJs) complexes formed between BBB endothelial cells, inhibiting paracellular diffusion of water-soluble molecules [1, 2]. In addition to sealing the paracellular pathway, BBB endothelial cells keep out unwanted compounds from the brain by their lack of fenestrae and low pinocytotic activity [3]. To meet the high demand of the neuronal cells for energy and to drive efflux of toxic metabolites from the CNS, BBB endothelial cells express unique combinations of enzymes and transporters [4].

The TJs of the BBB are distinct from those of other endothelial cells with respect to their high complexity and continuity of junctional strands that rather resemble those of the epithelial cells [5]. Freeze fracture studies demonstrated that in contrast to the peripheral vasculature, which shows predominantly E-face associated TJs, TJs of the BBB are mainly P-face associated [6]. P-face association of TJs-strands has been shown to correlate with barrier properties of the BBB [7]. Additionally, the protein expression signature at the TJs is very unique, being composed of occludin, junctional adhesion molecules and members of the claudins family [2]. The claudins are integral membrane proteins exclusively found in the TJs of all epithelia and endothelial cells, and they are essential and sufficient in establishing TJs and thus paracellular diffusion barriers [8]. In mammals, there are presently 27 known members of the claudin family, which exert different functions and present tissue and developmental stage specific expression patterns [9]. Thus, a different combination of claudins establishes specific TJs, which ultimately regulates their tightness [10]. It is known that some claudins can tighten the cleft between two adjacent cells, such as claudin-1 and claudin-3 [11, 12], while others form paracellular pores that contribute to a controlled passage of ions and water through the TJs, e.g. claudin-2 and claudin-16 [13, 14]. However, for some claudins, a precise function is not yet known. In the BBB endothelium, it was suggested that claudin-3, claudin-5 and claudin-12 contribute to the tightness of this barrier [15]. Claudin-5 is the most predominant claudin expressed in the BBB TJs and is essential for the establishment of BBB TJs during development, since its absence leads to perinatal death of mice due to increased permeability of the BBB to small molecular tracers [15, 16]. Moreover, with an inducible knock-down mouse model, it was seen that suppression of claudin-5 in the TJs leads to the disruption of BBB integrity and ultimately to seizures and behavioral changes [17], which demonstrates the importance of claudin-5 in the maintenance of this paracellular barrier in the adult. However, claudin-5-deficient mice display morphologically intact TJs suggesting the presence of other proteins localized to BBB TJs. Claudin-3 was described to be involved in the induction and maintenance of the BBB [18, 19]. However, expression of claudin-3 at the BBB TJs has been repeatedly questioned [20, 21] and more recently, its absence from the BBB endothelium was confirmed by employing a combination of methods including immunostaining, Western Blotting and single cell RNAseq analysis (scRNAseq) of the brain endothelium [1, 22]. scRNAseq of brain endothelial cells has also confirmed lack of expression of the TJ sealing claudin-1 at the BBB, as previously described [4, 22–24]. As claudin-5 by itself induces E-face associated TJs this has raised the question if another member of the claudin family could contribute to the formation of P-face associated TJs in the BBB [16]. Expression of claudin-12, an unusual member of the claudin family, has additionally been described at the BBB [15] and its expression in brain endothelial cells was recently confirmed by us [1]. Claudin-12 is an atypical member of the claudin family because it does not have a PDZ binding motif, which mediates the interaction of claudins with the cytoskeleton, by allowing the binding to the intracellular scaffolding proteins ZO-1, ZO-2 and ZO-3 [25]. Thus, its potential contribution to BBB TJs still remains unknown.

To answer this question, we generated a claudin-12^lacZ/lacZ^ C57BL/6J mouse, with a lacZ cassette inserted in the open reading frame (ORF) of claudin-12, which allows us not only to use it as a reporter gene for claudin-12 expression, but also to take advantage of the null allele to investigate the function of this protein. We observed broad expression of claudin-12 in numerous tissues and most prominently in smooth and striated muscle cells. Within the brain, expression of claudin-12 was detected in many different cell types with most prominent expression in neurons and astrocytes. Determining the subcellular localization of claudin-12 protein was prohibited due to the lack of antibodies specifically and selectively detecting claudin-12 protein. Nonetheless, our study rules out an essential role for claudin-12 in regulating BBB integrity under non-inflammatory or neuroinflammatory conditions, as we did not observe any aggravation of experimental autoimmune encephalomyelitis (EAE), an animal model of multiple sclerosis (MS). Yet, an in-depth phenotypic study of the claudin-12^lacZ/lacZ^ C57BL/6J mice points to possible roles of claudin-12 in select neurological functions and, more prominently, in cardiovascular functions.

## Materials and methods

### Generation of claudin-12^lacZ/lacZ^ C57BL/6J mice

Three gene-targeted embryonic stem cell (ES) clones (Cldn12_13208A-A6, Cldn12_13208AE2 and Cldn12_13208AF12) from KOMP project number KO1756 were requested from the KOMP repository at UC Davis. The mutation was generated on the Velocigen platform by Regeneron Pharmaceuticals (Tarrytown, NJ). The insertion of Velocigene cassette ZEN-Ub1 created a deletion of 760 bps between positions 5507663–5508422 of Chromosome 5 (Genome Build37) (*J:136110*). This deletion replaces the entire coding sequence residing only within in exon 5 with a lacZ cassette and a LoxP flanked neomycin-selection cassette. The deletion includes 28 bps upstream of the translational start site and 21 bps downstream of the stop codon. Parental ES cells are VGB6, which are derived from the C57BL/6NTac inbred mouse strain. The sequence of this allele is available here: https://www.i-dcc.org/imits/targ_rep/alleles/38187/escell-clone-genbank-file. ES clone Cldn12_13208A-A6 was injected into BALB/c blastocysts at the Institute of Laboratory Animal Science, University of Zurich. Five chimeric males were born and mated to C57BL/6J albino females. One of the chimeric males produced a single black male out of 40 offspring. The single black male was mated to C57BL/6J females and the offspring genotyped by PCR using the following primers: Cldn12_GT_SD (5′-CTCCTAGCCTCATCCGACTGAAACG-3′), Cldn12_ ΔGT3_TDF (5′-CTGCTGTTCGTTTGGTATTGTGCATG-3′) and PGK 3′UTR FW1 (5′-GGGTGGGATTAGATAAATGCCTGCTCT-3′). PCR-cycling conditions were: 94 °C for 4 min., and 35 repeats of 94 °C for 30 s., 62 °C for 30 s., 72 °C for 60 s., followed by 94 °C for 4 min. The wild-type (WT) allele was detected by a 586 bp band, while the neo-allele gave rise to a 775 bp band. To avoid any influence of the ubiquitin-C promoter driving the neomycin-resistance gene on the claudin-12 phenotype, the LoxP flanked neo-cassette was deleted by crossing claudin-12 neo heterozygous mice to ZP3-Cre (Tg(ZP3-cre)93Knw) mice expressing cre in the female germ line (oocytes) [26] (a gift from Pawel Pelczar). After deletion of the neo-cassette, claudin-12 mutated mice were genotyped by PCR using the following primers: Cldn12_FW2 (5′-TTTCTGATAGGATGGGTAGGTGGT GG-3′), Cldn12_REV2 (5′-CAGGCCCGTGTAAATCGTCAGGT-3′), LacZ-5′REV1 (5′-GAGCGAGTAACAACCCGTCGGATTCT-3′). PCR-cycling conditions were: 94 °C for 4 min., and 35 repeats of 94 °C for 30 s., 62 °C for 30 s., 72 °C for 60 s., followed by 94 °C for 4 min. The WT allele was detected by a 425 bp band, while the claudin-12-lacZ-allele gave rise to a 607 bp band.

### German Mouse Clinic

The claudin-12^lacZ/lacZ^ mouse line was backcrossed to C57BL/6J four times prior to the German Mouse Clinic (GMC) Primary Screen. Health status was confirmed to be specific pathogen-free according to FELASA recommendations. Using the platform established at the GMC, we performed a primary phenotypic analysis of a total of 60 (15 mice each of each genotype and sex) [27–29] exactly as described in the Additional file 6.

### Mouse housing

Mice were housed in individually ventilated cages (IVC) under specific pathogen-free conditions at 22 °C and 55% relative humidity with free access to chow and water. Animal procedures executed were approved by the Veterinary Office of the Canton Bern (permit no. BE55/09, BE42/14, BE72/15, BE31/17). At the GMC mice were maintained in IVC cages with water and standard mouse chow (Altromin no. 1314) according to the GMC housing conditions and German laws. All tests performed at the GMC were approved by the responsible authority of the district government of Upper Bavaria.

### Experimental autoimmune encephalomyelitis (EAE)

Active EAE was induced in 8–12-week-old female claudin-12^lacZ/lacZ^ C57BL/6J mice, claudin-12^lacZ/+^ C57BL/6J mice and their WT C57BL/6J littermates exactly as previously described [30, 31]. Weights and clinical disease activity were assessed twice daily and scored as follows: 0, healthy; 0.5, limp tail; 1, hind leg paraparesis; 2, hind leg paraplegia; 3, hind leg paraplegia with incontinence implementing the 3R rules as described in [32]. Two independent experiments were performed.

### Isolation of brain microvessels

Primary mouse brain microvessels were isolated from WT and claudin-12^lacZ/lacZ^ C57BL/6J mice as previously described in detail [33]. The only modification to this previous protocol was that instead of the final plating step, microvessels were incubated in a red blood cell lysis buffer (0.83% ammonium chloride and Tris–HCl, pH = 7.5), for 5 min, RT. After two washing steps, microvessels were lysed in HES lysis buffer (10 mM HEPES, 1 mM EDTA solution, 250 mM sucrose solution), in the presence of protease inhibitor cOmplete ULTRA Tablets, Mini, EDTA-free, EASYpack (1 tablet/10 mL) (Roche Diagnostics, Mannheim, Germany), and kept at − 20 °C.

### SDS-PAGE

In accordance to the isolation of brain microvessels, muscle tissue from WT and claudin-12^lacZ/lacZ^ C57BL/6J mice was also lysed in HES lysis buffer (10 mM HEPES, 1 mM EDTA solution, 250 mM sucrose solution), in the presence of protease inhibitor cOmplete ULTRA Tablets, Mini, EDTA-free, EASYpack (1 tablet/10 mL) (Roche Diagnostics, Mannheim, Germany). Protein concentration was measured using the Pierce™ BCA Protein Assay Kit (Thermo Scientific™ Pierce™ Protein Biology, Waltham, USA), according to the manufacturer’s instructions. 20 μg of each sample were boiled at 95°, for 5 min, and loaded onto a 10% SDS-polyacrylamide gel and transferred to a nitrocellulose membrane (Amersham Protan, GE Healthcare, United Kingdom), using a Trans-Blot Turbo transfer system (BioRad Laboratories, Hercules, CA, USA), according to the manufacturer’s instructions. Membranes were blocked with Rockland Buffer (Rockland, Limerick, PA, USA) for 1 h at RT and incubated overnight at 4 °C with three different rabbit anti-mouse claudin-12 antibodies (IBL, cat. no 18801; Abcam cat. no ab107061; Invitrogen, cat. no 388200), mouse anti-mouse β-actin (Merck, cat. no A5316), rabbit anti-α-tubulin (Abcam, cat. no ab4074), rabbit anti-claudin-5 (Thermo Fisher Scientific, cat. no 34-1600), rabbit anti-occludin (Thermo Fisher Scientific, cat. no 71-1500), or rabbit anti-ZO-1 (Thermo Fisher Scientific, cat. no 61-7300). On the following day, membranes were washed and incubated with secondary antibodies goat-anti-rabbit Alexa Fluor^®^ 680 (Thermo Fisher Scientific, cat. No A21109) and goat anti-mouse IRDye^®^ TM 800 (Rockland Immunochemicals, cat. no 605-732-125), for 1 h at RT. Proteins were detected by the Odyssey near infrared imaging system and software (LI-COR Biotechnology, Lincoln, NE, USA). Band intensity for the three independent experiments was quantified using the ImageJ software (NIH, Bethesda, MD, USA) and normalized against β-actin.

### Quantitative real-time PCR analysis (qRT-PCR)

RNA was extracted from the heart tissue of WT and claudin-12^lacZ/lacZ^ C57BL/6J mice by using the High Pure RNA Isolation kit (Hoffman-La Roche, Basel, Switzerland). cDNA was obtained from each sample’s total isolated RNA with the SuperScript III First-Strand Synthesis System (Invitrogen, Carlsbad, CA, USA) and the qRT-PCR was done as previously described [4]. The pairs of primers that were used in this study are the following: 5′-CTGAGTTCACTAAGCTGACTTTGG-3′ (sense primer within exon 3) and 5′-CCTGTCTGCGCCTCTGAT-3′ (anti-sense primer within exon 4) for the 5´prime untranslated region (UTR) of claudin-12 mRNA; 5′-TGCTTGGAGAAACGCTGATT-3′ (sense primer within exon 4) and 5′-GTGGCTGCGTGGACATCT-3′ (anti-sense primer within the ORF integral to exon 5) for the open reading frame (ORF); 5′-TGCTTGGAGAAACGCTGATT-3′ (sense primer within exon 4) and 5′-GTCTGTCCTAGCTTCCTCACTG-3′ for lacZ detection. ΔC_T_ value was obtained (average C_T_ value of target gene − average C_T_ value of S16) and relative expression values of three independent experiments were calculated according to the comparative 2^−ΔΔCt^ method (ΔΔC_T_ = Δ C_T_ sample − Δ C_T_ WT).

### LacZ staining of tissue sections

WT, Tie2-lacZ and claudin-12^lacZ/+^ C57BL/6J mice were anesthetized with Isoflurane Baxter (Arovet, Dietikon, Switzerland) and perfused with 1% paraformaldehyde (PFA). As positive controls, Tie2-lacZ (B6.FVB-Tg (TIE2-lac)182Sato) transgenic mice were used [34]. Brain, retina, liver, heart, tongue, skeletal muscle, intestine and kidney were removed, washed twice in PBS (pH = 7.4) and fixed with 1% PFA + 5 mM EDTA + 2 mM MgCl_2_, for 4 h at 4 °C. The tissue was next incubated in 18% sucrose, overnight at 4 °C, prior to embedding in Tissue-Tek O.C.T. compound (Sakura Finetek, The Netherlands) and snap-freezing [35]. 20 µm cryosections were cut from these tissues, air dried at RT for 4 h, and fixed in 1% PFA + 5 mM EDTA + 2 mM MgCl_2_ for 3 min. Afterwards, a rehydration step was performed with PBS (pH = 7.4). For LacZ staining, sections were incubated overnight with 0.1% X-Gal at 37 °C in the dark. Next, sections were washed once in distilled water, incubated for 1 min in 1% Neutral Red (Sigma-Aldrich, St. Louis, Missouri, USA) and differentiated in distilled water. Then, cryosections were dehydrated in consecutive steps in EtOH 75%, EtOH 85%, EtOH 95% and EtOH 100%, and finally in Xylol before mounting in Entellan^®^ (Merck Millipore, Darmstadt, Germany). Three independent experiments were performed and were analysed using a Nikon Eclipse E600 microscope connected to a Nikon Digital Camera DXM1200F with the Nikon NIS-Elements BR3.10 software (Nikon, Egg, Switzerland). Images were processed and mounted using Adobe Illustrator software (Adobe Systems, CA, USA).

### Immunofluorescence staining of tissue sections

WT and claudin-12^lacZ/lacZ^ C57BL/6J mice were anesthetized with Isoflurane Baxter (Arovet, Dietikon, Switzerland) and perfused with 1% PFA. Brains and liver were removed, embedded in Tissue-Tek^®^ OCT compound (Sakura Finetek, The Netherlands) and snap-frozen. Cryosections were cut at 6 μm or 10 μm thickness and fixed in either ice cold acetone or 2% PFA for 10 min and air-dried. Cryosections were stained as described before [36, 37]. Sections were incubated for 1 h, RT, with the following primary antibodies: two different rabbit anti-mouse claudin-12 antibodies (IBL, cat. no 18801; Invitrogen, cat. no 388200), rat anti-PECAM-1 (in house, clone Mec13.3), rabbit anti-β-galactosidase (Thermo Fisher Scientific, cat. no A-11132), rat anti-CD140b (eBioscience, cat. no 14-1402-82), mouse anti-GFAP (Sigma-Aldrich, cat- no G3893), mouse anti-NeuN (clone A60, Millipore, cat. no MAB377), rabbit anti-fibronectin (DAKO, cat. no A0245) and biotinylated goat anti-mouse IgG (Vector, cat. no BA-9200). After the washing steps, the sections were incubated with the following secondary antibodies: goat polyclonal IgG Cy3 anti-rabbit (Jackson ImmunoResearch, cat. no 11-165-144), donkey anti-rat IgG (H+L) Alexa Fluor 488 (Thermo Fisher Scientific, cat. no A-21208), donkey anti-rabbit IgG (H+L) Alexa Fluor 488 (Thermo Fisher Scientific, cat. no A-21206), donkey anti-mouse IgG (H+L) Alexa Fluor 488 (Thermo Fisher Scientific, cat. no A-32766), Streptavidin-Cy3 (Vector, cat. no SA-1300) and with DAPI (1:1000, Thermo Fisher Scientific, Carlsbad, CA, USA), for 1 h, RT. Fluorescence stainings were performed in at least three independent experiments and were analysed using a Nikon Eclipse E600 microscope connected to a Nikon Digital Camera DXM1200F with the Nikon NIS-Elements BR3.10 software (Nikon, Egg, Switzerland). Images were processed and mounted using Adobe Illustrator software (Adobe Systems, CA, USA).

### Statistics

Statistical analysis was performed using GraphPad Prism 6.0 software. To compare two groups, an unpaired t-test with Welch’s correction was performed. For the analysis of the EAE experiments, a Mann–Whitney U-test was performed. Results are shown as mean ± SD and a p < 0.05 was considered significant. If not stated otherwise, data generated by the German Mouse Clinic was analyzed using R (Version 3.2.3). Tests for genotype effects were made by using t-test, Wilcoxon rank sum test, linear models, or ANOVA and posthoc tests, or Fisher’s exact test depending on the assumed distribution of the parameter and the questions addressed to the data. A p-value < 0.05 has been used as level of significance; a correction for multiple testing has not been performed.

## Results

### Generation of claudin-12^lacZ/lacZ^ C57BL/6J mice

**Fig. 1:**
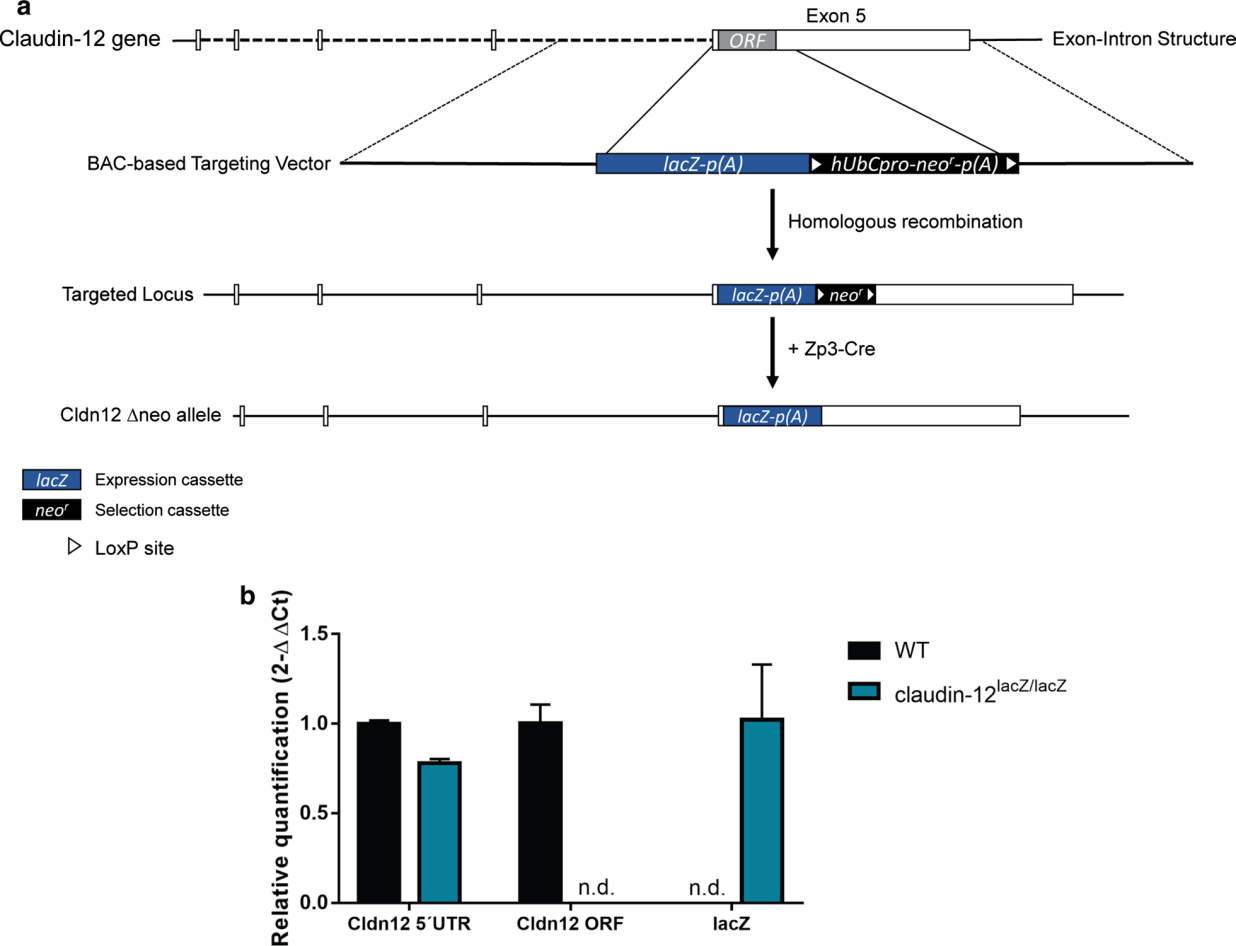
Claudin-12 targeting strategy in C57BL/6J mice. **a** Schematic representation of the knock-out strategy. The open reading frame of claudin-12 is located in exon 5 and is targeted by a BAC-based targeting vector that contains an expression and neomycin-selection cassettes. In the targeted allele, a deletion of 760 bps is present, which removes the entire coding sequence. Arrows indicate LoxP sites surrounding the selection cassette that are targeted by Cre recombinase. **b** Relative expression of different regions of the claudin-12 gene in heart muscle samples isolated from WT and claudin-12^lacZ/lacZ^ C57BL/6J mice was assessed by qRT-PCR. For each region of the gene, technical triplicates in three independent experiments were measured. Relative quantification is represented by the 2^−ΔΔCt^ value. *Cldn12* claudin-12, *UTR* untranslatable region, *ORF* open reading frame, *n.d.* not detectable

Insertion of the Velocigene cassette ZEN-Ub1 within the claudin-12 gene created a deletion of 760 bps. This deletion replaces most of exon 4, including the entire open reading frame (ORF) of claudin-12, with a lacZ cassette (Fig. 1a). Gene targeting was performed in VGB6 ES cells (derived from C57BL/6NTac mice) and correct targeting was assessed employing the Velocigen platform, including genotyping by quantitative PCR to verify loss of a claudin-12 WT allele (Velocigene citation). Injection of claudin-12-targeted ES cells into BALB/c blastocysts and consecutive embryo transfer gave rise to 5 chimeric males, one of which allowed for germ line transmission of the mutated claudin-12 allele. Heterozygous F1 mice were produced by mating with C57BL/6J females. Heterozygous offspring were mated to Zp3-Cre mice to remove the floxed neo-cassette. The claudin-12^lacZ/+^ mice were further backcrossed to C57BL/6J.

To further confirm the deletion of the claudin-12 ORF after insertion of the lacZ cassette and based on the histochemical analysis described below, we performed a transcript analysis by qRT-PCR of heart samples of WT and claudin-12^lacZ/lacZ^ C57BL/6J mice (Fig. 1b). As expected, we observed that both WT and claudin-12^lacZ/lacZ^ C57BL/6J heart samples had transcripts for the upstream region of the ORF and while in WT C57BL/6J mice the presence of the ORF was detected, in claudin-12^lacZ/lacZ^ C57BL/6J mice this was not the case (Fig. 1b). LacZ was only detected in claudin-12^lacZ/lacZ^ C57BL/6J heart samples, confirming the successful insertion of the lacZ cassette in the claudin-12 ORF (Fig. 1b). As a next step, we then looked at the viability of the claudin-12^lacZ/lacZ^ C57BL/6J mice. Interestingly, claudin-12^lacZ/lacZ^ mice survived to weaning age at less than the expected Mendelian ratio (17.2%) (Table 1). Nonetheless, adult mutant mice were fertile and healthy. No difference in the mortality of adult claudin-12 null mice versus WT littermates was observed.

**Table 1:** Claudin-12 genotype ratios of mice at weaning age, with the total number of mice obtained per genotype (WT, claudin-12^lacZ/+^ and claudin-12^lacZ/lacZ^) and per sex, with the corresponding percentage

Claudin-12 genotype	Mice weaned		Female mice		Male mice	
	#	%	#	%	#	%
WT	102	27.4	52	13.9	50	13.4
lacZ/+	206	55.4	103	27.7	103	27.7
lacZ/lacZ	64	17.2	24	6.5	40	10.8
Total	372	100	179	48.1	193	51.9

### Claudin-12 is expressed in many organs including the CNS

Taking advantage of the lacZ reporter gene in our claudin-12^lacZ/+^ C57BL/6J mouse line, we investigated claudin-12 expression in tissue sections from several organs of the mouse including liver, heart, kidney, intestine, skeletal muscle, tongue, retina and the brain, by analyzing β-galactosidase activity with the chromogenic substrate X-gal by histochemistry. We detected prominent β-galactosidase activity in skeletal muscle cells and cardiac myocytes, as well as in smooth muscle cells of the tongue and the intestine, suggesting an important role of claudin-12 in muscular function. Furthermore, epithelial cells in the kidney and the intestine as well as hepatocytes in the liver showed prominent β-galactosidase staining (Additional file 1).

**Fig. 2:**
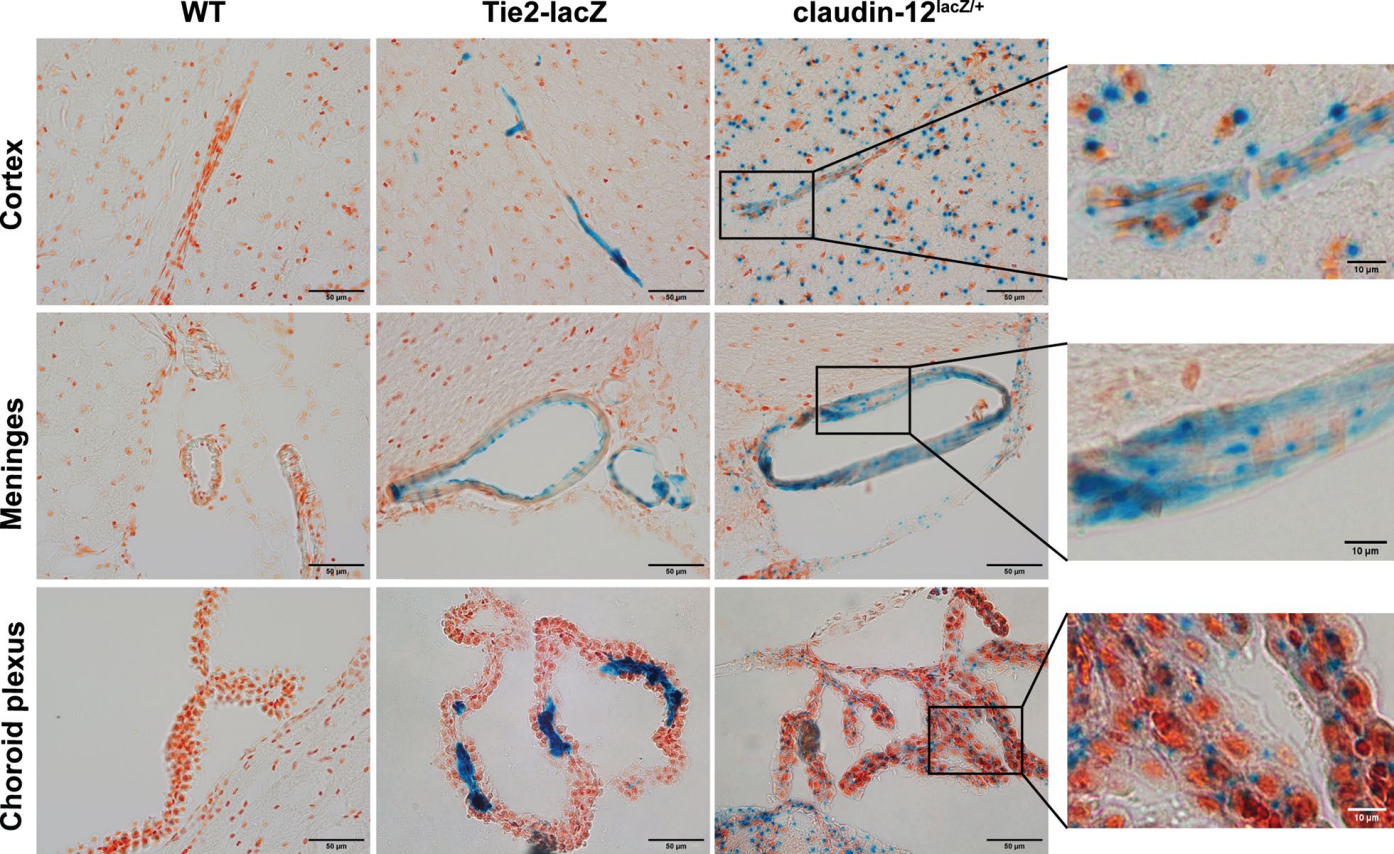
Claudin-12 expression in the CNS. Immunohistochemical staining of frozen brain sections from WT, Tie2-lacZ and claudin-12^lacZ/+^ C57BL/6J mice for β-galactosidase activity (blue), with examples of the cortex, meninges and choroid plexus. Three independent stainings were done. Boxed areas are shown in higher magnification. Scale bars = 10 μm and 50 μm, as indicated

To determine if claudin-12 is expressed at the BBB, we investigated β-galactosidase activity in brain sections from claudin-12^lacZ/+^ C57BL/6J mice compared to WT mice, as a negative control, and of Tie2-lacZ mice, as control for β-galactosidase activity in endothelial cells [34]. As expected, no β-galactosidase activity was detected in the WT brain tissue (Fig. 2). In the brain tissue from Tie2-lacZ mice, we detected abundant β-galactosidase activity in the endothelial cells of the vasculature in the brain parenchyma and the meninges, as well as in the choroid plexus as reported previously [34] (Fig. 2). In contrast, brain tissue sections from claudin-12^lacZ/+^ C57BL/6J mice did not show any endothelial cell specific β-galactosidase activity. We rather observed a characteristically punctate pattern of β-galactosidase activity in numerous cells in the CNS throughout the entire brain parenchyma, in the meninges, in the choroid plexus, and also in retinal tissue (Fig. 2 and Additional file 2). At the same time, β-galactosidase activity was visible in the smooth muscle cell layer of larger vessels in the brain parenchyma as well as in the meninges, but we did not detect any specific lacZ activity in the endothelial cells of these larger vessels or in endothelial cells of microvessels forming the BBB (Fig. 2). Our data shows that claudin-12 expression in the CNS is not restricted to the vasculature and that vascular expression seems to be more prominent in smooth muscle cells or pericytes than in the endothelial cells per se. At this point, we took advantage of a publicly available datasets of single-cell transcriptomics of all the major vascular and vessel-associated cell types from the adult mouse brain (http://betsholtzlab.org/VascularSingleCells/database.html) [22, 38] and of bulk RNA datasets from purified neurons, astrocytes, microglia, endothelial cells, pericytes, and oligodendrocytes from mouse cortex [24], and looked at occurrence of claudin-12 mRNA. These datasets show low amounts of claudin-12 mRNA expression in the brain of mice, in cells such as pericytes, oligodendrocytes, vascular smooth muscle cells, astrocytes, fibroblasts and neurons and endothelial cells of the vasculature. Thus, our detection of β-galactosidase activity in numerous cells in the brain (Fig. 2) is in accordance to these previous findings.

**Fig. 3:**
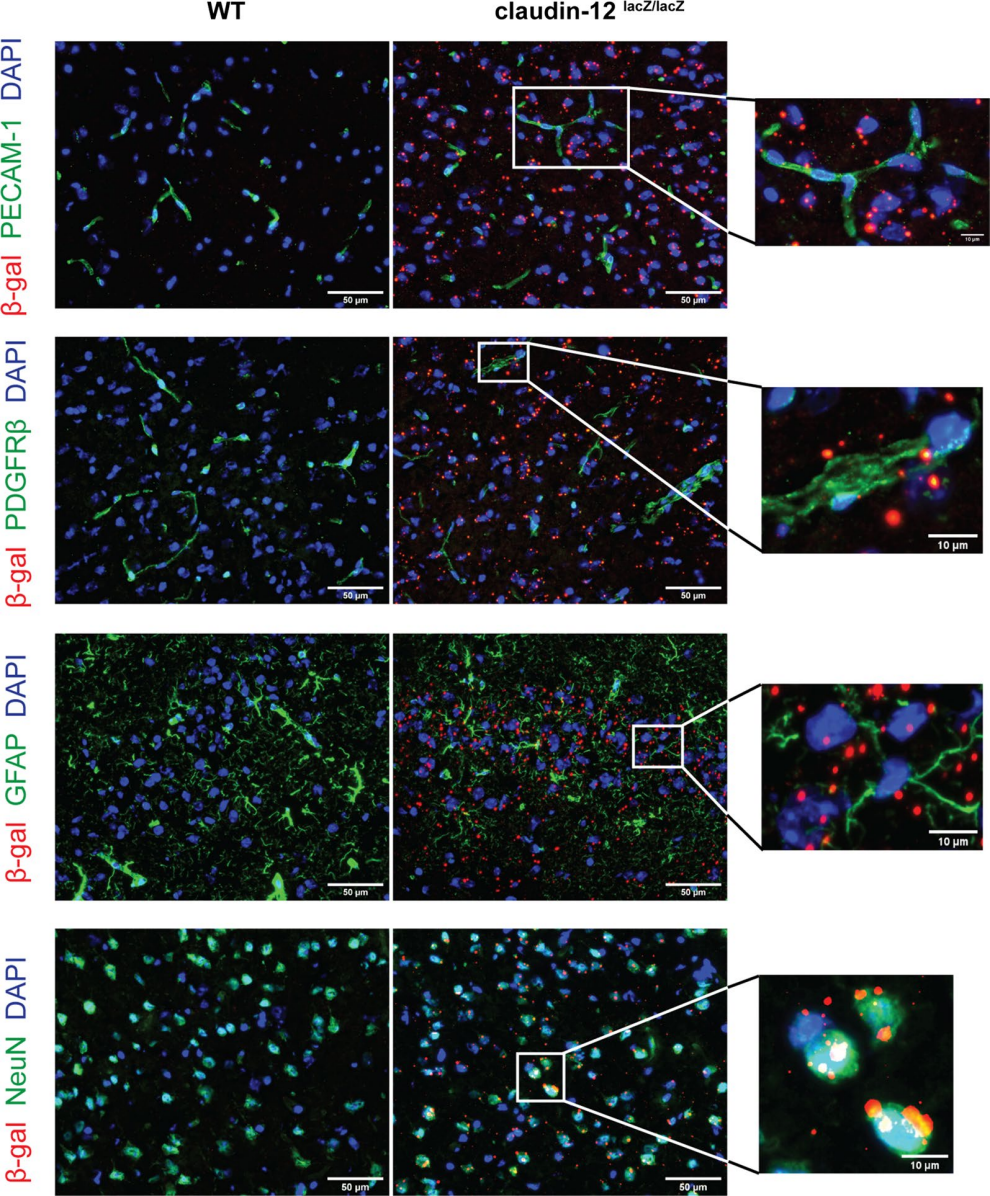
Claudin-12 is expressed by different cell types in the CNS. Immunofluorescence staining of frozen brain sections from WT and claudin-12^lacZ/lacZ^ C57BL/6J mice, for β-galactosidase (red) and endothelial cells (PECAM-1), pericytes (PDGFRβ), astrocytes (GFAP) and neurons (NeuN) (green), in the brain cortex. Nuclei are stained with DAPI (blue). Boxed areas are shown in higher magnification. β-gal stands for β-galactosidase. Scale bars = 10 and 50 μm, as indicated. Two independent series of stainings were performed

We next aimed to localize claudin-12 expression to specific cells in the brain tissue. Taking advantage of an antibody recognizing β-galactosidase, we performed immunofluorescence stainings on brain sections and confirmed that β-galactosidase staining was absent from brain tissue sections from WT C57BL/6J mice, but again detectable throughout the brain tissue from claudin-12^lacZ/lacZ^ C57BL/6J mice (Fig. 3 and Additional file 3). We detected positive immunostaining for β-galactosidase in rare PECAM-1-positive endothelial cells and in few PDGFRβ-positive pericytes (Fig. 3 and Additional file 3). At the same time, β-galactosidase staining was more frequently localized in GFAP-positive astrocytes and most abundant in NeuN-positive neurons in the cortex, in the hippocampus and in the cerebellum (Fig. 3 and Additional file 3). Taken together, our data show that claudin-12 is expressed at low levels in different CNS cell types and is mainly expressed by astrocytes and neurons.

### Lack of reagents to detect expression and localization of claudin-12 protein

**Fig. 4:**
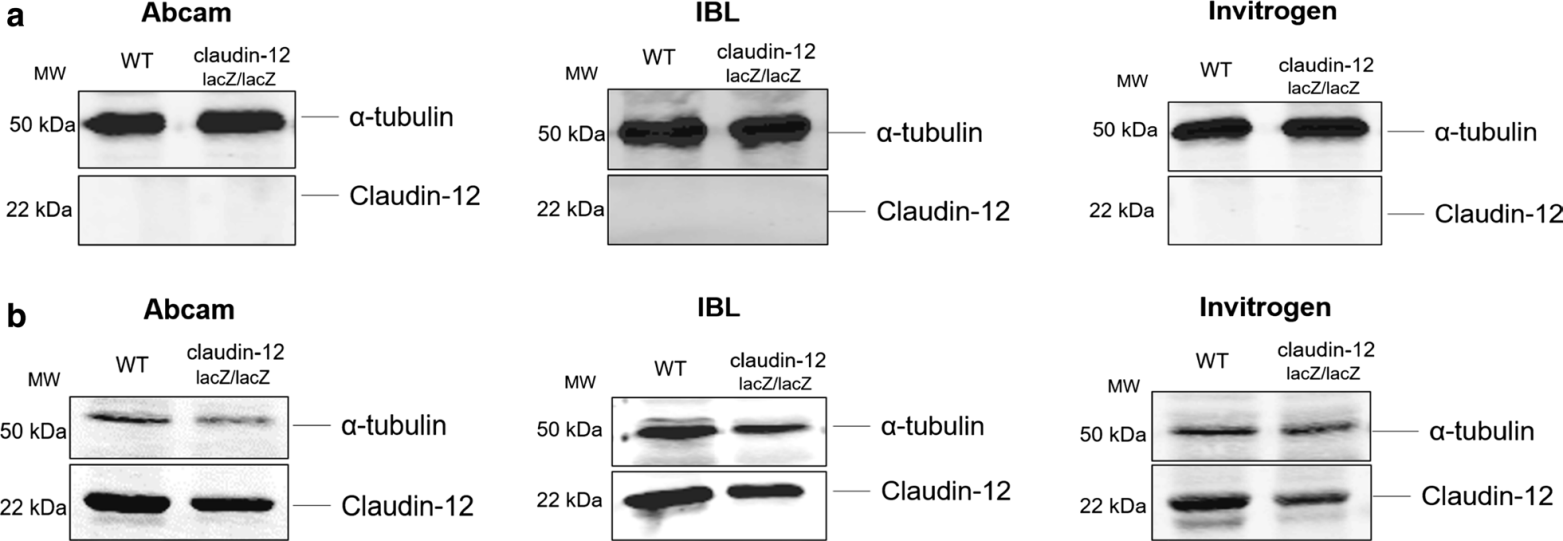
Lack of reagents allowing to detect expression of claudin-12 protein. Immunoblot analysis of claudin-12 protein levels in freshly isolated brain microvessels (**a**) and in skeletal muscle (**b**) from WT and claudin-12^lacZ/lacZ^ C57BL/6J mice, using three different polyclonal anti-claudin-12 antibodies. Three independent immunoblots for both skeletal muscle and brain microvessels were analysed

Since we could not readily identify prominent claudin-12 expression in brain endothelial cells by detection of lacZ reporter activity, we attempted to identify claudin-12 protein expression by immunostaining in brain tissue sections from WT and claudin-12^lacZ/lacZ^ C57BL/6J mice. We used three different commercially available anti-claudin-12 antibodies, and obtained contradictory results. In fact, the antibody from IBL detects endothelial claudin-12 in brain sections of WT but also in mutant mice (Additional file 4A), while the antibody from Invitrogen failed to produce any positive immunostaining for claudin-12 in brain tissues in both (data not shown). We then tried to detect claudin-12 in liver sections, to test the suitability of the available antibodies in a tissue where high expression of claudin-12 was observed by us (Additional file 1). As observed for the brain, we found the antibody from IBL to detect claudin-12 in the liver of WT and mutant mice, while the one from Invitrogen completely failed to stain claudin-12 in both tissues (Additional file 4B). Finally, we aimed to detect claudin-12 protein expression by SDS-PAGE to completely exclude that the contradictory results are not due to technical issues. Thus, besides the samples from brain microvessels from WT and claudin-12^lacZ/lacZ^ C57BL/6J, we also used samples from skeletal muscle from WT and claudin-12^lacZ/lacZ^ C57BL/6J mice as a control, since it is one of the organs that showed very high β-galactosidase activity. Using the same antibodies as before, we again obtained inconclusive results, since we did not detect any band for claudin-12 in both WT and in the claudin-12^lacZ/lacZ^ brain microvessel samples with any of the antibodies (Fig. 4a). However, in the samples from skeletal muscle we detected a 22 kDa band in both, the WT samples, but also in the claudin-12^lacZ/lacZ^ samples (Fig. 4b). This set of results suggests that the available antibodies fail to detect the low expression levels of claudin-12 protein in CNS blood vessels from WT mice. At the same time, our observations underscore the available claudin-12 antibodies do not allow for specific detection of claudin-12 by Western Blot, which is most likely due to a cross-reactivity with another claudin, as we detected a band of comparable size to claudin-12 in the skeletal muscle of the claudin-12^lacZ/lacZ^ samples, which lack expression of claudin-12.

### Absence of claudin-12 does not affect BBB integrity nor aggravate EAE

**Fig. 5:**
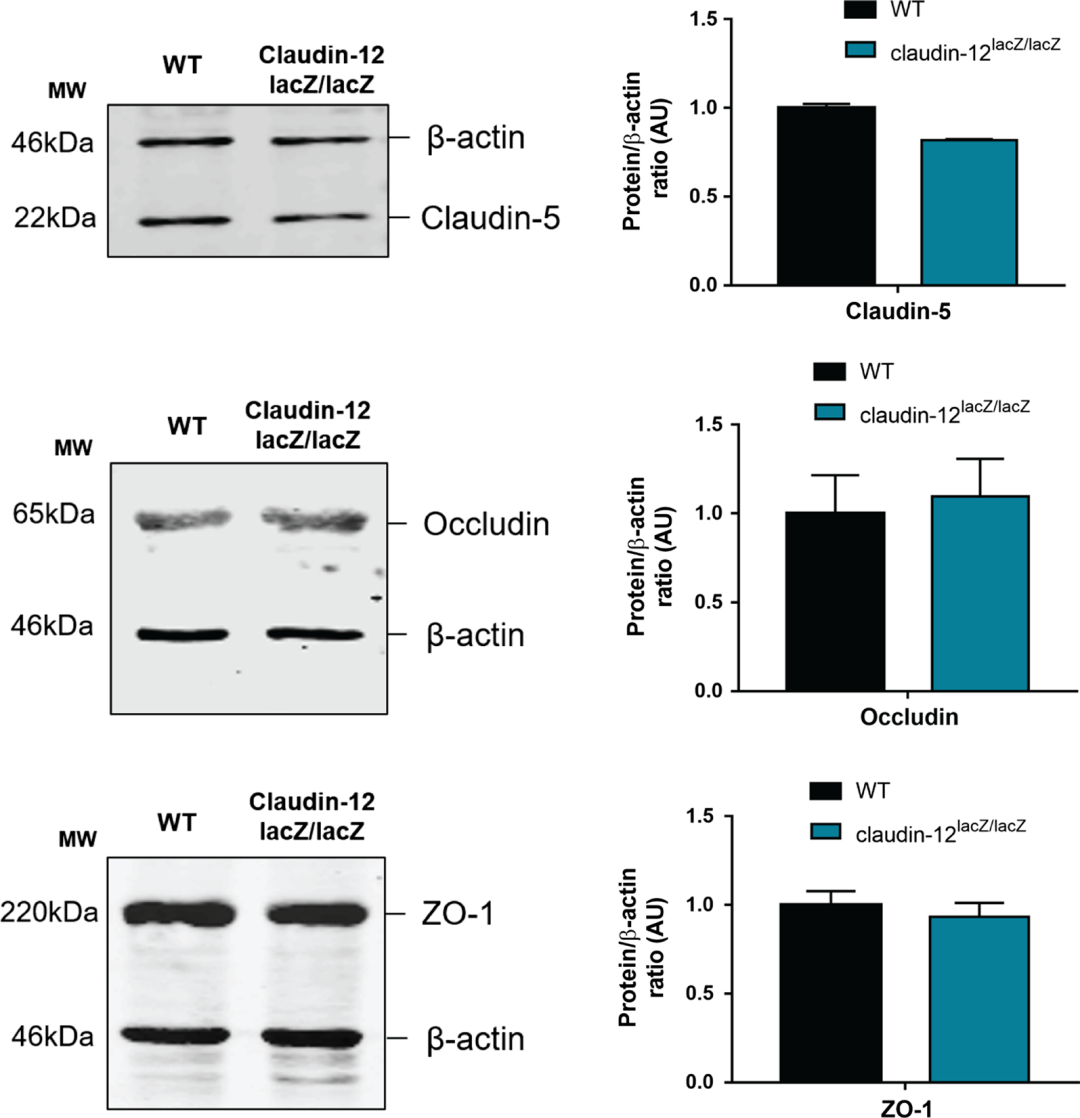
Absence of claudin-12 does not affect the expression of tight junction proteins in brain microvessels. Immunoblot analysis and quantification of the protein levels of claudin-5, occludin and ZO-1 in freshly isolated brain microvessels from WT and claudin-12^lacZ/lacZ^ C57BL/6J mice. Bar graphs show mean ± SD of two independent experiments

**Fig. 6:**
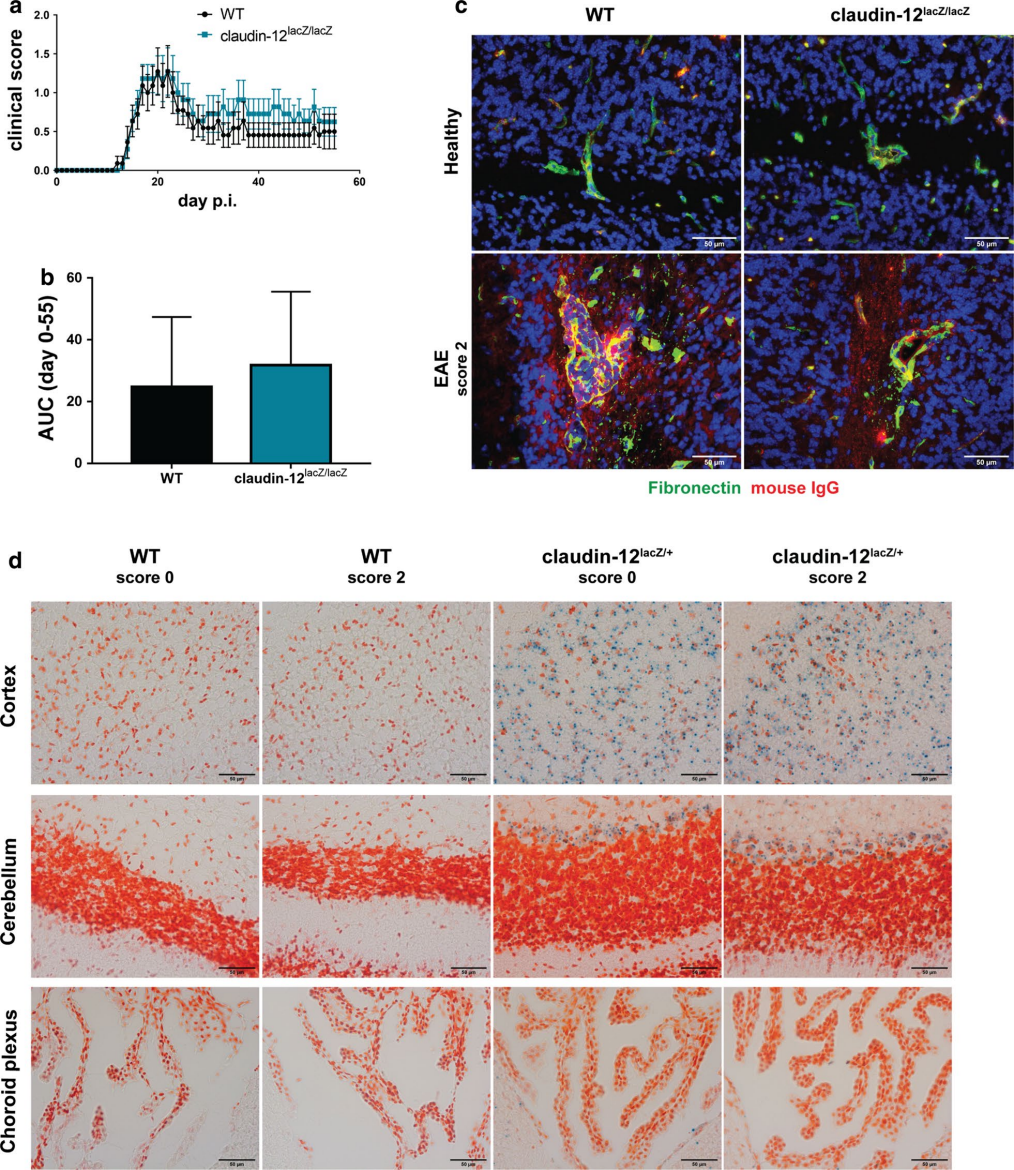
Absence of claudin-12 does not affect BBB integrity nor aggravate the development of EAE. **a** Graph of the clinical disease course of one representative MOG_aa35-55_ induced aEAE in WT (black line; n = 11) and claudin-12^lacZ/lacZ^ C57BL/6J mice (blue line; n = 11) is shown. Average disease scores ± SEM as assessed twice daily are shown. **b** Disease severity determined by the area under the curve (AUC) and analyzed until day 55. Bar graphs represent the mean ± SD of two independent experiments including 21 WT and 21 claudin-12^lacZ/lacZ^ C57BL/6J mice. **c** Immunofluorescence staining for fibronectin (green) and mouse IgG (red) on frozen brain sections from WT and claudin-12^lacZ/lacZ^ C57BL/6J is shown. Upper row—healthy mice, lower row mice suffering from EAE. Two independent stainings were performed. Scale bar = 50 μm. **d** Immunohistochemical staining of frozen brain sections from WT and claudin-12^lacZ/+^ C57BL/6J mice after induction of aEAE. β-galactosidase (blue) staining can be seen in examples of the cortex, cerebellum and choroid plexus. Animals with score 0 were induced with aEAE but did not develop any clinical symptom, whereas animals with score 2 presented hindleg paraplegia. Three independent stainings were done. Scale bar = 50 μm

Despite the low expression levels of claudin-12 detected in this study, we next asked if lack of claudin-12 expression will affect composition and integrity of BBB TJs. To this end, we first analyzed expression of tight junction-associated proteins in brain microvessels from WT or from claudin-12^lacZ/lacZ^ C57BL/6J healthy mice. We detected no difference in the protein expression levels of claudin-5, occludin and ZO-1 in freshly isolated brain microvessles from claudin-12^lacZ/lacZ^ C57BL/6J versus WT mice (Fig. 5). To study a potential role of claudin-12 in the BBB during neuroinflammation we next took advantage of the animal model of MS, EAE, that mimics two major pathological hallmarks for MS, namely BBB breakdown and immune cell infiltration. TJ disruption contributes to BBB dysfunction in MS and is well mimicked in EAE [23, 39], and thus, we induced active EAE in WT and claudin-12^lacZ/lacZ^ C57BL/6J mice. No significant difference was found between WT and mutant mice when looking at onset of the clinical disease, the disease incidence (99% in WT and claudin-12^lacZ/lacZ^ mice) and the overall disease severity as measured as the area under the curve for WT and claudin-12^lacZ/lacZ^ C57BL/6J mice (Fig. 6a, b). Thus, lack of claudin-12 did not aggravate the development of EAE.

To determine if absence of claudin-12 nevertheless affects BBB integrity, in healthy conditions and during EAE, we performed an immunofluorescence staining for endogenous vascular tracers, namely fibronectin and mouse IgG (Fig. 6c). Under healthy conditions, we did not detect any immunostaining for fibronectin or mouse IgG beyond the confines of the brain parenchymal vasculature in WT and claudin-12^lacZ/lacZ^ C57BL/6J mice (Fig. 6c). On the other hand, we found significant leakage for both vascular molecules into the brain tissue in mice suffering from EAE, as expected (Fig. 6c). At the same time and in accordance to the clinical disease course we did not observed any apparent differences in the amount of leakage of fibronectin or mouse IgG across the BBB between WT and claudin-12^lacZ/lacZ^ C57BL/6J mice (Fig. 6c). Taken together, these data underscore that in the C57BL/6J mouse claudin-12 does not play an essential role in maintaining BBB integrity in health or neuroinflammation.

To determine if claudin-12 may play a different role in BBB function as compared to regulating TJ integrity we investigated if claudin-12 expression was upregulated under inflammatory conditions. To this end, we induced EAE in WT and claudin-12^lacZ/+^ C57BL/6J mice and collected brain tissue at the peak of the disease (day 17). We then performed histochemistry in brain sections from these mice and analysed β-galactosidase activity using the chromogenic substrate X-gal. As expected, lacZ staining was only visible at the level of the cortex in heterozygous animals, while the WT littermates did not show any traces of staining (Fig. 6d). Nonetheless, when we compared claudin-12 expression in animals that did not develop any disease phenotype (score 0) to animals that reached a clinical score of 2, there was no difference in the observed staining (Fig. 6d). Therefore, we can conclude that under neuroinflammatory conditions, claudin-12 expression is not upregulated.

### Phenotyping of claudin-12^lacZ/lacZ^ C57BL/6J mice

As the next step, and by acknowledging the broad expression observed for claudin-12 in a diverse range of organs, we submitted the claudin-12^lacZ/lacZ^ C57BL/6J mice to a systematic multiparameter phenotypic analysis performed by the German Mouse Clinic (Helmholtz Centre Munich). The specific aim of this study was to understand if lack of claudin-12 in the CNS leads to neurological alterations independent from BBB maintenance or if lack of claudin-12 in other organs would affect entirely different physiological functions. Thus, a standardized phenotypic analysis of a total of 30 WT mice (15 of each sex) and 30 claudin-12^lacZ/lacZ^ C57BL/6 mice (15 of each sex) was performed at the age of 10 to 21 weeks, determining over 550 different parameters in the areas of behavior, neurology, the eye, steroid metabolism, bone and cartilage, nociception, clinical chemistry and hematology, immunology, allergy, pathology, cardiology and molecular phenotyping. Overall, we found several phenotypic changes in claudin-12^lacZ/lacZ^ C57BL/6J mice compared to controls. An overview table of the results can be found in Additional file 5 and the results are summarized in Additional File 6 and published at the GMC phenomap (https://www.mouseclinic.de). In the context of understanding potential roles of claudin-12 in the CNS, it is interesting to note that analysis of basic behavioral and motor functions in the Open Field assay showed decreased locomotor activity in claudin-12^lacZ/lacZ^ C57BL/6J mice and decreased anxiety in female claudin-12^lacZ/lacZ^ C57BL/6J mice. Assessment of auditory function did not show significant alterations but only minor changes in auditory brain stem response or acoustic startle reflex. Interestingly, while showing normal retinal thickness, both male and female claudin-12^lacZ/lacZ^ C57BL/6J mice showed significantly reduced axial eye lengths compared to controls.

Beyond phenotypic changes associated with the CNS we found female but not male mutants presented with alterations in energy metabolism and in cardiovascular functions. In contrast, male but not female mutants showed increased body weight when compared to controls and increased absolute and normalized liver and spleen weights. Taken together claudin-12^lacZ/lacZ^ C57BL/6J mice displayed a number of mostly subtle phenotypic changes, which in part were sex specific and correlate to the ubiquitous expression of claudin-12 found in this study.

## Discussion

The BBB plays a crucial role in maintaining CNS homeostasis, by preventing free diffusion of solutes into the brain, with the BBB TJs playing an essential role in sealing the paracellular cleft between the BBB endothelial cells. The claudin family of TJs proteins are crucial in establishing and maintaining TJ function at the BBB [2]. During recent years, there is an increasing understanding of the role of each individual claudin in contributing to tissue specific TJ functions [40]. Thus, to understand BBB TJ function it is essential to understand which claudins are expressed in brain endothelial cells and localized to BBB TJs. Bearing in mind the important role each claudin plays in maintaining barrier function under physiological conditions and how disturbance of their localization at the TJs can contribute to barrier breakdown, it is urgent to fully clarify the molecular claudin makeup of BBB TJs. Claudin-5 is the most enriched claudin in the vascular endothelium of the BBB. Its absence is associated with BBB integrity breakdown, an early feature in the development of MS [41], and it is also implied in the pathology of stroke, traumatic brain injury and schizophrenia [17, 42]. However, it was seen that claudin-5 knock-out mice still present morphologically intact TJs [16]. As claudins are essential and sufficient for TJ induction this means that another claudin that is present at the BBB TJs contributes to TJs formation in the absence of claudin-5. In the past years, claudin-1 and claudin-3 were repeatedly suggested to be part of the BBB TJs [3, 18]. However, our recent studies have shown that these claudins are not expressed in brain endothelial cells and can thus not contribute to BBB TJs [1, 23]. Claudin-12 is an additional claudin, expression of which has been described in BBB TJs [15]. Claudin-12 is an unusual claudin, lacking a PDZ binding motif in its C-terminal domain, which is required to connect claudins via scaffolding proteins such as ZO-1 to the cytoskeleton [25]. The possible function of claudin-12 at the BBB has remained unknown to date.

To tackle this question, in the present study we created claudin-12^lacZ/+^ and claudin-12^lacZ/lacZ^ C57BL/6J mice allowing to explore claudin-12 expression as well as the impact of claudin-12 deletion on BBB TJ integrity. Making use of the lacZ reporter allele, we found a characteristic punctate β-galactosidase activity in numerous cells throughout the entire CNS. Our findings are in accordance to the staining pattern previously observed by the International Mouse Phenotyping Consortium (IMPC) when analysing reporter gene expression of a lacZ allele of different claudin-12 mutant mice (Cldn12^tm1b(EUCOMM)Wtsi^) by whole mount lacZ staining of adult Cldn12^tm1b(EUCOMM)Wtsi^ mice (http://www.mousephenotype.org/data/search/impc_images?kw=%22cldn12%22). This previous dataset thus underscores ubiquitous expression of claudin-12 throughout the brain and furthermore shows that claudin-12 expression is enriched in the grey matter of the spinal cord.

In our present study, vascular expression of claudin-12 based on β-galactosidase activity was clearly visible in the smooth muscle cell layer of larger vessels in the brain parenchyma, as well as in the meninges rather than in the endothelial cells of these vessels. In turn, it could be specifically detected by endothelial-cell-specific lacZ activity in Tie2-lacZ reporter mice. Furthermore, we failed to detect lacZ activity in endothelial cells of microvessels forming the BBB in our brain tissue sections. This suggests that if claudin-12 is expressed in brain endothelial cells, this is at a very low level. This observation was confirmed by our immunofluorescence stainings, which allowed to localize expression of the β-galactosidase reporter in sparse PECAM-1-positive brain endothelial cells and few PDGFRβ-positive pericytes. At the same time, positive immunostaining for the β-galactosidase reporter was found in GFAP-positive astrocytes and most abundantly in NeuN-positive neurons further underscoring low but ubiquitous expression of claudin-12 in the brain of the mouse.

In fact, previous scRNAseq transcriptomic analysis of brain vessels isolated from developing (P7) and adult mice confirms low levels of expression of claudin-12 mRNA in a wide range of vascular and CNS vessel associated cell types (https://markfsabbagh.shinyapps.io/vectrdb/ and http://betsholtzlab.org/VascularSingleCells/database.html) [22, 38, 43]. Average reads for claudin-12 mRNA were found to be low and similar in endothelial cells along the vascular tree and were found to be higher in smooth muscle cells compared to brain endothelial cells. In addition, low expression of claudin-12 was also detected in microglial cells, oligodendrocytes, astrocytes and neurons [22, 24] (http://www.brainrnaseq.org/). Last but not least, brain endothelial expression levels of claudin-12 were found comparable to those detected using the same methodology in lung endothelial cells. Our present observations underscore low to undetectable expression of claudin-12 in brain endothelium and visible expression in vascular smooth muscle cells, pericytes, astrocytes and neurons. As these cells do not typically form TJs, this suggests that claudin-12 plays a role other than forming classical TJs. Expression data on claudin-12 mRNA in isolated brain microvessels and the detected regulation of claudin-12 mRNA expression must therefore be carefully interpreted as it may reflect regulation of claudin-12 expression in pericytes, smooth muscle cells or even in astrocytes, rather than the brain endothelial cells proper [44].

To determine the subcellular localization of claudin-12 in the CNS vasculature we made use of commercially available antibodies to claudin-12 and performed side-by-side immunostainings on brain sections of WT and claudin-12^lacZ/lacZ^ C57BL/6J mice. We could not observe any difference in the staining patterns produced by the anti claudin-12 antibodies in the brain sections of WT and claudin-12^lacZ/lacZ^ C57BL/6J mice. This observation therefore prohibits reliable subcellular localization of claudin-12 in the CNS vasculature and suggests that antibodies recognizing claudin-12 cross-react with other molecules present in CNS microvessels. Cross-reactivity of claudin-12 detecting antibodies was confirmed by Western Blots, which showed detection of a similar sized band in tissue samples from claudin-12^lacZ/lacZ^ C57BL/6J mice.

In light of our previous observations that claudin-3 targeting antibodies produce an identical staining pattern in CNS microvessels in claudin-3 deficient and WT C57BL/6J mice [1], it seems that claudin-targeting antibodies often produce false-positive detection of the respective claudin due to cross-reactivity with other claudins, which may be due to the highly conserved nature of the immunogenic domains of this family of proteins [45]. Employing presently available claudin-12 antibodies we could therefore not reproduce the prominent immunostaining for claudin-12 originally observed at the BBB in mice when using a home-made anti-claudin-12 antibody [15]. Lack of reliable antibodies detecting claudin-12 thus also questions previous observations of claudin-12 protein expression in the human brain endothelial cell line hCMEC/D3 [46]. However, significant expression of claudin-12 mRNA has also been detected in this cell line [47]. At the same time, transcriptome profiling of freshly isolated cells from the human brain (available at http://www.brainrnaseq.org) shows even lower expression levels for claudin-12 mRNA in human brain endothelial cells as observed in the mouse, but at the same time confirms expression of claudin-12 in cells other than brain endothelium in the CNS [48]. Thus, the final confirmation if claudin-12 is expressed at the protein level in CNS blood vessels and if so its precise cellular and subcellular localization within the CNS vascular cells in mouse and man remains to be determined.

In accordance to the available transcriptome profiles on brain cells [22, 24], we observed that claudin-12 is expressed in various cells of the brain vasculature as well as in astrocytes and neurons. Thus, a functional impact of claudin-12 in the settings of neuroinflammation remains possible. We therefore hypothesized that its absence could still affect BBB integrity. BBB breakdown is a major hallmark of MS and is associated with loss of TJs (summarized in [2]). It has previously been shown that impaired BBB integrity, e.g. in PECAM-1-deficient mice, aggravates EAE [49], while endothelial cell-specific ectopic expression of claudin-1 inhibits BBB breakdown during EAE and ameliorates chronic disease [23]. Therefore, we here compared development of clinical EAE in claudin-12^lacZ/lacZ^ C57BL/6J mice to that in their WT littermates. We did not observe any differences in clinical EAE between WT and claudin-12^lacZ/lacZ^ C57BL/6J mice. We also did not detect any differences in BBB integrity or immune phenotype, e.g. upregulation of adhesion molecules in claudin-12^lacZ/lacZ^ C57BL/6J mice versus WT C57BL/6J mice during EAE. Thus, although claudin-12 is expressed in various cells of the CNS including vascular cells, its absence does not affect BBB integrity during autoimmune neuroinflammation.

Conclusions about claudin-12 expression in the present study rely on available mRNA data sets [22, 24, 38] and on the novel information provided in the present study based on claudin-12 lacZ reporter gene expression. Our present observations on claudin-12 expression using our claudin-12 reporter mouse allowed for robust detection of β-galactosidase staining in various tissues, such as skeletal muscle, liver, intestine and kidney, suggesting that while claudin-12 is expressed in different cells of the CNS, its expression is stronger in the periphery. These findings are consistent with the previous observations on claudin-12 expression in the Cldn12^tm1b(EUCOMM)Wtsi^ mice (http://www.mousephenotype.org/data/search/impc_images?kw=%22cldn12%22).

Due to the presence of claudin-12 in a wide range of organs, we decided to perform a systematic multiparameter phenotypic analysis of our claudin-12^lacZ/lacZ^ C57BL/6J mouse and compare it to WT littermates.

The mutant mice showed some behavioral impairments. While it is currently not possible to draw conclusions concerning the exact nature of these alterations, our findings are bolstered by the reported behavioral changes in the aforementioned IMPC line (Cldn12tm1b(EUCOMM)Wtsi). Although there are differences between the two lines and the observed effects are subtle, behavioral alterations now described in both suggest that loss of claudin-12 likely affects brain function to some degree, most probably due to its lack of expression in neurons. It is therefore of interest to better understand the potential role of claudin-12 in the different cells of the brain. Claudin-12 has in addition been shown to be expressed in the inner ear as well as well as in retina, as confirmed by us in the present study [50, 51]. Thus, the subtle alterations in auditory and retinal function might also be interesting for further analysis. Moreover, it is important to highlight that some morphological and functional alterations of the heart were detected in claudin-12^lacZ/lacZ^ C57BL/6J mice in the cardiovascular screen. Here, additional studies are required to elucidate the role and functional implications of claudin-12 in the murine cardiac muscle. Also, the fact that claudin-12 is strongly expressed in skeletal muscle and is reported to be involved in vitamin D-dependent calcium absorption [52] asks for further clarifications in the role of this protein in the muscle, although under standard conditions no changes have been detected for muscle function analyzed by grip strength.

## Conclusion

In summary, with this study we suggest that claudin-12 expression is detectable, but low, in brain endothelial cells. Expression of claudin-12 is observed in additional cells of the vasculature like pericytes and in many cell types of the brain parenchyma, including astrocytes and neurons. Due to the lack of availability of antibodies reliably detecting claudin-12, we could not determine presence and subcellular localization of claudin-12 protein in the microvascular endothelium. Claudin-12 expression in the CNS is not upregulated under inflammatory stimuli and its absence does not impair BBB integrity or aggravate the development of EAE. This suggests that claudin-12 is not required for maintaining BBB integrity. However, lack of claudin-12 promoted broad neurological and behavioral changes, the exact mechanisms underlying this phenotype to be investigated.

## Supplementary information

**Additional file 1.** Claudin-12 expression in non-CNS tissue. Immunohistochemical staining for β-galactosidase, in blue, in frozen sections from heart, skeletal muscle, tongue, liver, intestine and kidney, from WT and claudin-12^lacZ/+^ C57BL/6J mice. Three independent stainings were done. Scale bar = 100 μm.

**Additional file 2.** Claudin-12 expression in CNS tissue. Immunohistochemical staining for β-galactosidase activity (blue), in frozen sections from the eye of claudin-12^lacZ/+^ C57BL/6J mice and of a Tie2-lacZ mouse, as a positive control. Images show a similar detection of β-galactosidase activity in cells not associated with the vasculature in the brain cortex and in the retina of claudin-12^lacZ/+^ C57BL/6J mice. Arrows point to vessel associated staining in the brain of the claudin-12^lacZ/+^ C57BL/6J mouse and to endothelial cell specific staining in the Tie2-LacZ mouse. * highlights the typical punctate staining pattern for β-galactosidase activity in the brain. Boxed areas are shown in higher magnification. Three independent stainings were performed. Scale bars = 10 and 50 μm, as indicated.

**Additional file 3.** Claudin-12 is expressed by different CNS cell types. Multi-color immunofluorescence staining of frozen brain sections from WT and claudin-12^lacZ/lacZ^ C57BL/6J mice, for β-galactosidase in red and PECAM-1 (endothelial cells), PDGFRβ (pericytes), GFAP (astrocytes) and NeuN (neurons) in green, in the hippocampus and cerebellum. Nuclei are stained with DAPI (blue). β-gal stands for β-galactosidase. Two independent stainings were done. Scale bar = 50 μm.

**Additional file 4.** Lack of reagents allowing to localize expression of claudin-12 protein. **(A)** Immunofluorescence staining of frozen brain sections from WT and claudin-12^lacZ/lacZ^ C57BL/6J mice with the anti-claudin-12 antibody from IBL represented in green produces indistinguishable vascular and apparently junction associated staining in the brain tissues of both, WT and the claudin-12^lacZ/lacZ^ C57BL/6J mice. Scale bar = 50 μm. **(B)** Immunofluorescence staining of frozen liver sections from WT and claudin-12^lacZ/lacZ^ C57BL/6J mice, using two different antibodies for claudin-12, represented in red. Notice how the antibody from IBL stains WT and claudin-12^lacZ/lacZ^ tissue, while the anti-claudin-12 antibody from Invitrogen does not recognize claudin-12 in neither of the samples. Nuclei are stained with DAPI (blue). Three independent stainings were done. Scale bar = 100 μm.

**Additional file 5.** Overview of tests performed by the German Mouse Clinic and summary of results.

**Additional file 6.** Complete phenotyping report of claudin-12^lacZ/lacZ^knock-in C57BL/6J mice.

## Data Availability

The datasets generated and presented in the current study are available in the German Mouse Clininc phenomap (https://www.mouseclinic.de).

## Abbreviations

BBB: blood–brain barrier
CNS: central nervous system
TJs: tight junctions
WT: wild-type
P-face: protoplasmic face
E-face: exoplasmic face
scRNAseq: single cell RNAseq
ORF: open reading frame
EAE: experimental autoimmune encephalomyelitis
MS: multiple sclerosis
ES: embryonic stem cell
GMC: German Mouse Clinic
