# Supplementary figures and images for: Claudin-12 is not required for blood–brain barrier tight junction function
Source: Fluids Barriers CNS. 2019 Sep 12;16:30. doi: 10.1186/s12987-019-0150-9 (PMC6739961; doi:10.1186/s12987-019-0150-9)

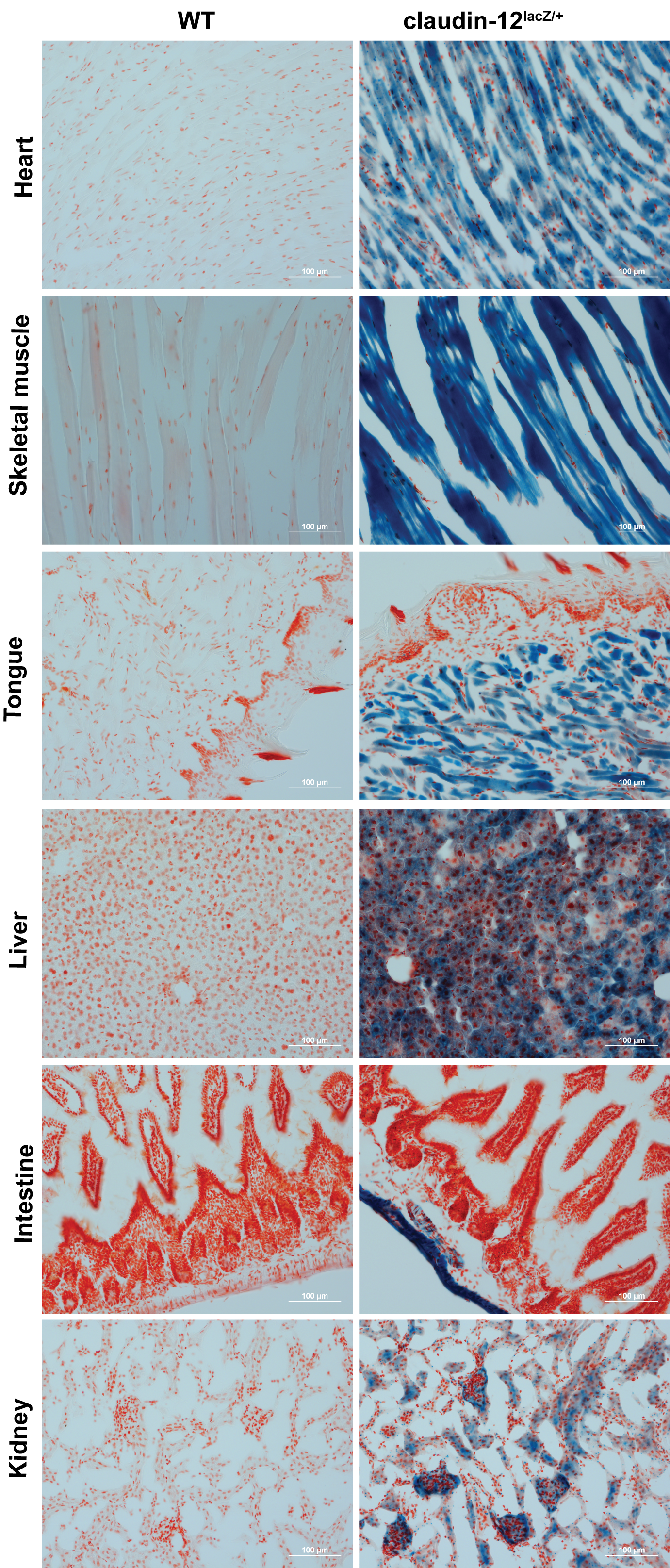

Supplement: Supplementary file 1 — Additional file 1. Claudin-12 expression in non-CNS tissue. Immunohistochemical staining for β-galactosidase, in blue, in frozen sections from heart, skeletal muscle, tongue, liver, intestine and kidney, from WT and claudin-12lacZ/+ C57BL/6J mice. Three independent stainings were done. Scale bar = 100 μm. [file 12987_2019_150_MOESM1_ESM.tif]

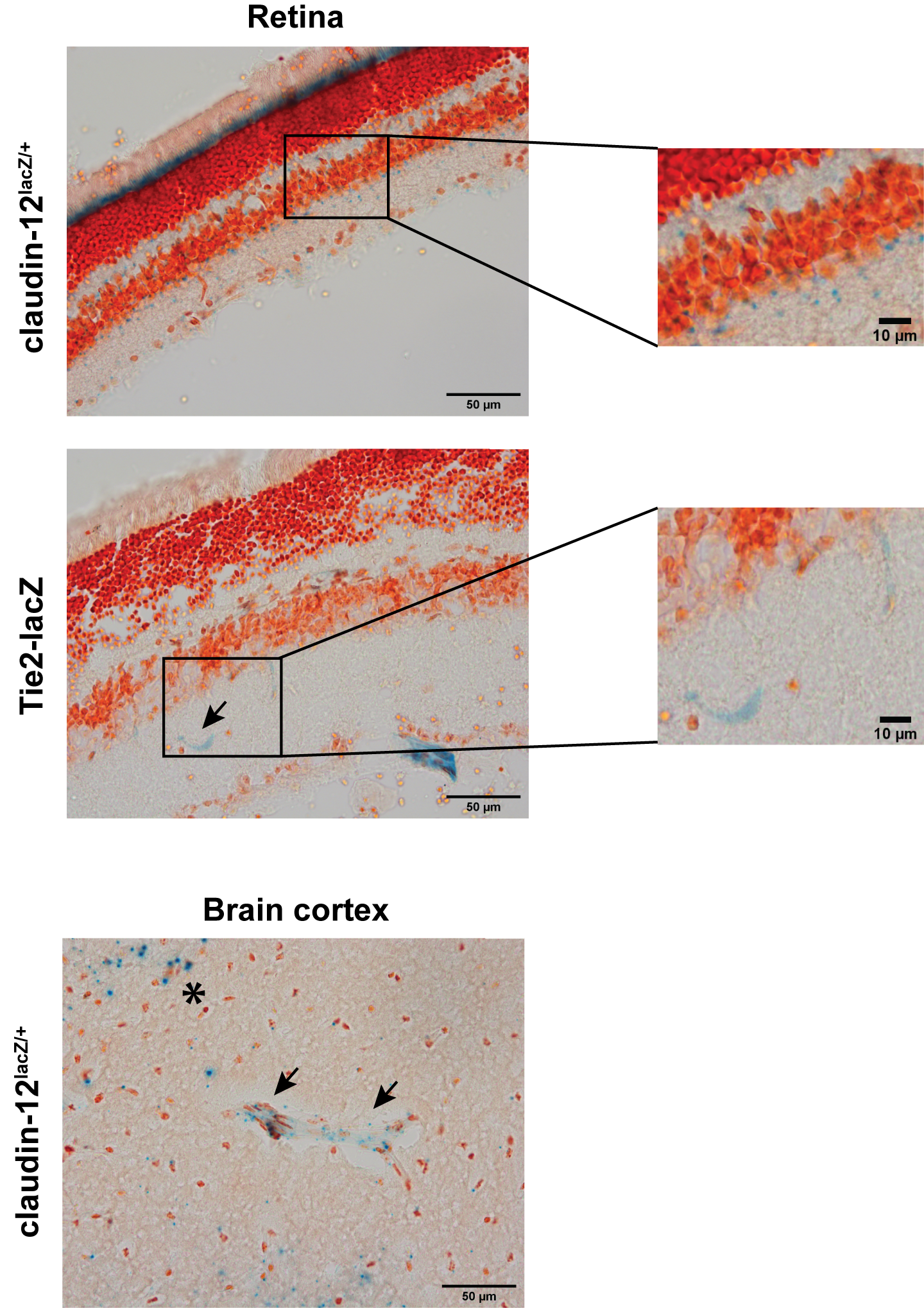

Supplement: Supplementary file 2 — Additional file 2. Claudin-12 expression in CNS tissue. Immunohistochemical staining for β-galactosidase activity (blue), in frozen sections from the eye of claudin-12lacZ/+ C57BL/6J mice and of a Tie2-lacZ mouse, as a positive control. Images show a similar detection of β-galactosidase activity in cells not associated with the vasculature in the brain cortex and in the retina of claudin-12lacZ/+ C57BL/6J mice. Arrows point to vessel associated staining in the brain of the claudin-12lacZ/+ C57BL/6J mouse and to endothelial cell specific staining in the Tie2-LacZ mouse. * highlights the typical punctate staining pattern for β-galactosidase activity in the brain. Boxed areas are shown in higher magnification. Three independent stainings were performed. Scale bars = 10 and 50 μm, as indicated. [file 12987_2019_150_MOESM2_ESM.tif]

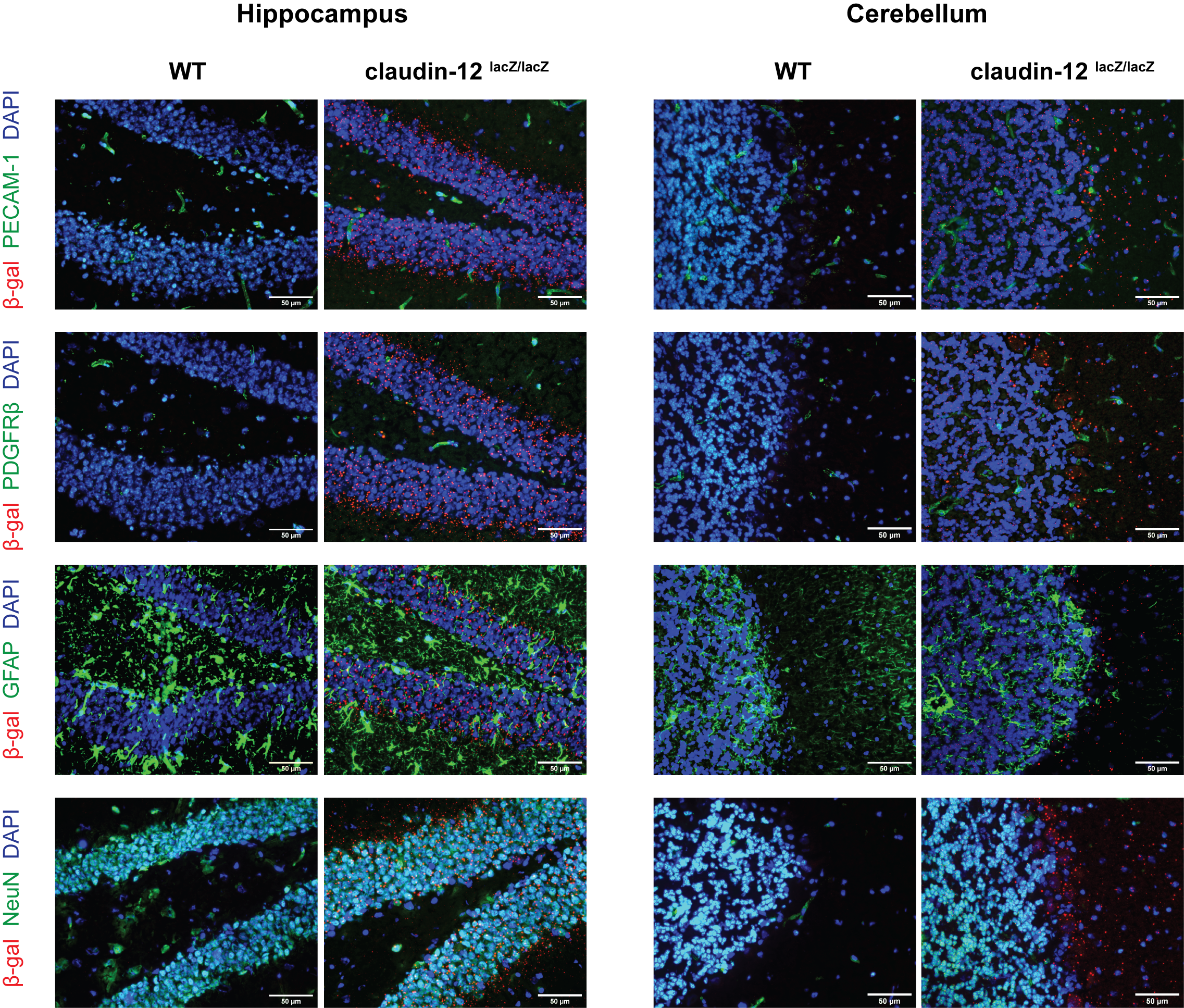

Supplement: Supplementary file 3 — Additional file 3. Claudin-12 is expressed by different CNS cell types. Multi-color immunofluorescence staining of frozen brain sections from WT and claudin-12lacZ/lacZ C57BL/6J mice, for β-galactosidase in red and PECAM-1 (endothelial cells), PDGFRβ (pericytes), GFAP (astrocytes) and NeuN (neurons) in green, in the hippocampus and cerebellum. Nuclei are stained with DAPI (blue). β-gal stands for β-galactosidase. Two independent stainings were done. Scale bar = 50 μm. [file 12987_2019_150_MOESM3_ESM.tif]

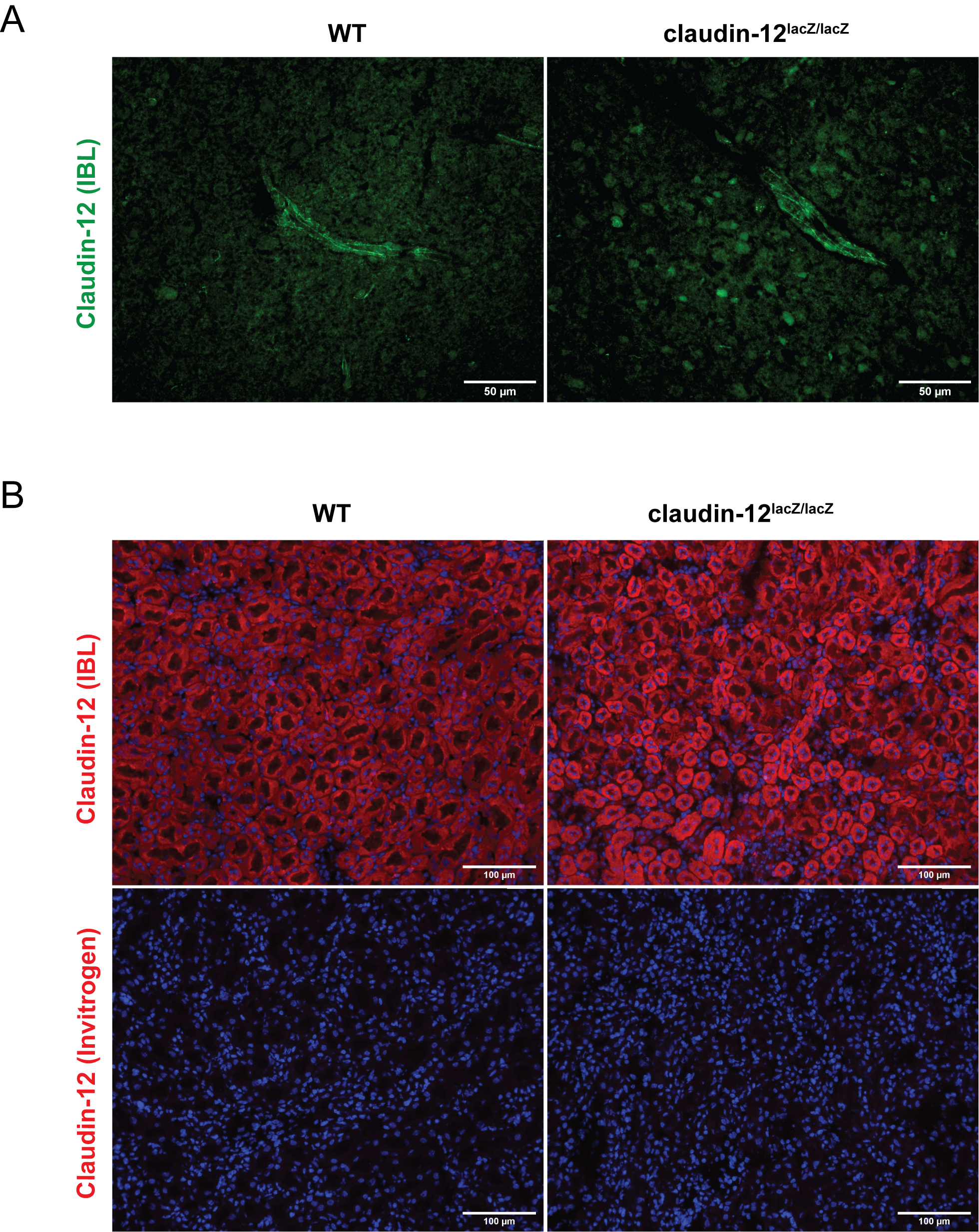

Supplement: Supplementary file 4 — Additional file 4. Lack of reagents allowing to localize expression of claudin-12 protein. (A) Immunofluorescence staining of frozen brain sections from WT and claudin-12lacZ/lacZ C57BL/6J mice with the anti-claudin-12 antibody from IBL represented in green produces indistinguishable vascular and apparently junction associated staining in the brain tissues of both, WT and the claudin-12lacZ/lacZ C57BL/6J mice. Scale bar = 50 μm. (B) Immunofluorescence staining of frozen liver sections from WT and claudin-12lacZ/lacZ C57BL/6J mice, using two different antibodies for claudin-12, represented in red. Notice how the antibody from IBL stains WT and claudin-12lacZ/lacZ tissue, while the anti-claudin-12 antibody from Invitrogen does not recognize claudin-12 in neither of the samples. Nuclei are stained with DAPI (blue). Three independent stainings were done. Scale bar = 100 μm. [file 12987_2019_150_MOESM4_ESM.tif]
