# Supplementary material for: Claudin-12 is not required for blood–brain barrier tight junction function
Source: Fluids Barriers CNS. 2019 Sep 12;16:30. doi: 10.1186/s12987-019-0150-9 (PMC6739961; doi:10.1186/s12987-019-0150-9)
Supplement: Supplementary file 5 — Additional file 5. Overview of tests performed by the German Mouse Clinic and summary of results. [file 12987_2019_150_MOESM5_ESM.docx]

**Additional File 5**

**Overview of tests performed by the German Mouse Clinic and summary of results**

| **Screen** | **Tests** | **Phenotype summary of**  ***claudin-12^lacZ/lacZ^ C57BL/6J mice*** |
| --- | --- | --- |
| **Dysmorphology, Bone and Cartilage** | Morphological observation, Clickbox test, DXA, X-Ray | Significantly decreased BMC and bone content in females. Concurrently body length was decreased in females. Changes in BMC are a secondary effect to the differences in body size and body weight. |
| **Behavior** | Pre-pulse Inhibition / Acoustic Startle Reflex | Acoustic startle reactivity tended to be decreased in the mutant mice. |
|  | Open Field | Decreased locomotor activity by the mutant mice in open field while the female mutant mice showed a pattern of decreased anxiety. |
| **Neurology** | Auditory Brainstem Response | Slightly reduced hearing sensitivity at 24 kHz. |
|  | Grip Strength, Rotarod, Modified SHIRPA, Lactate | Mutants showed more tail elevation at SHIRPA. Three female mutants showed tremor. Grip strength, rotarod performance as well as plasma lactate were without any differences with the methods applied. |
| **Eye Screen** | Optical Coherence Tomography | No changes. |
|  | Eye size, Scheimpflug, Eye Morphology | Reduced axial eye length, and a decrease of the retinal thickness of female mutants. |
| **Nociception** | Hotplate | No changes. |
| **Metabolic Screen** | Minispec and Indirect Calorimetry (TSE) | Mainly in female mutant mice body mass was decreased but fat content was slightly increased; Food uptake was lower and RER shifted towards lipid oxidation. Rearing was decreased in both, male and female mutants. |
| **Clinical Chemistry and Hematology** | Clinical Chemistry (*ad libitum fed* and fasted mice), Hematology, IpGTT | *Ad libitum fed*: Trend to decreased plasma protein and triglyceride levels and increased creatinine values and ALP activity in mutants.  *After fasting*: Subtle increase in non-HDL cholesterol; Slightly increased glucose and decreased NEFA in female mutants.  *Hematology*: Mild macrocytosis with increased MCH and decreased RBC, but increased WBC predominantly in mutant males.  *IpGTT:* Slightly decreased basal fasting glucose levels in mutants. |
| **Immunology Screen** | Flow Cytometry | No significant differences in the levels of antibodies, subtle sex-dependent changes in the frequencies of several minor leukocyte subpopulations. |
| **Allergy Screen** | IgE levels | No changes. |
|  | Transepidermal water loss | No changes. |
| **Molecular Phenotyping** | Illumina Bead Arrays | Organ: Heart  Gene regulation in heart is functionally associated with atherosclerosis, apoptosis, inflammatory response and concentration of lipid. |
| **Cardiovascular Screen** | Awake Echocardiography, Awake Electrocardiography | By echocardiography alterations were more often observed in female mutants. Mutants had smaller left ventricle (reduced inner diameter and LV Mass). The hearts performed in general better (increased FS and EF). However, the volume of blood pumped with each beat was reduced (reduced SV).  By electrocardiography, a mild decrease in atrio-ventricular conduction time (PQ and PR interval duration) was observed.  Caution in interpreting the data have to be taken as the female mutants were less heavy than female controls, which might influence heart dimensions. |
| **Pathology Screen** | Macroscopy, Microscopy | Increased absolute and normalized liver and spleen weights. Histological examination using light microscopy did not reveal any pathological changes that could be attributed to the genotype of the mice. |

Abbreviations: DXA - dual-energy X-ray absorptiometry; SHIRPA - SmithKline Beecham, Harwell, Imperial College, Royal London Hospital, phenotype assessment; IpGTT - intraperitoneal glucose tolerance test; BMC – bone mineral content; RER – respiratory exchange ratio ; ALP – alkaline phosphatase; NEFA – non-esterified fatty acids; MCH – mean corpuscular hemoglobin; RBC – red blood cell count; WBC – white blood cell count
