# Supplementary material for: Claudin-12 is not required for blood–brain barrier tight junction function
Source: Fluids Barriers CNS. 2019 Sep 12;16:30. doi: 10.1186/s12987-019-0150-9 (PMC6739961; doi:10.1186/s12987-019-0150-9)
Supplement: Supplementary file 6 — Additional file 6. Complete phenotyping report of claudin-12lacZ/lacZknock-in C57BL/6J mice. [file 12987_2019_150_MOESM6_ESM.pdf]

GMC German Mouse Clinic

The

GERMAN MOUSE CLINIC (GMC)

at the

Helmholtz Zentrum München

German Research Center for Environmental Health

Phenotyping report for

Claudin-12$^{lacZ/lacZ}$ C57BL/6J mice

HelmholtzZentrum münchen
German Research Center for Environmental Health

HELMHOLTZ
ASSOCIATION

0

Helmut Fuchs, Valérie Gailus-Durner, Thure Adler, Juan Antonio Aguilar Pimentel, Oana Veronica Amarie, Lore Becker, Julia Calzada-Wack, Lillian Garrett, Christine Gau, Manuela Gegenfurtner, Wolfgang Hans, Sabine M. Hölter, Marion Horsch, Dirk Janik, Tanja Klein-Rodewald, Christoph Lengger, Holger Maier, Kristin Moreth, Frauke Neff, Cornelia Prehn, Oliver Puk, Ildikó Rácz, Birgit Rathkolb, Jan Rozman, Ralph Steinkamp, Claudia Stöger, Annemarie Zimprich, Jerzy Adamski, Johannes Beckers, Raffi Bekeredjian, Dirk H. Busch, Jochen Graw, Martin Klingenspor, Thomas Klopstock, Markus Ollert, Tobias Stöger, Eckhard Wolf, Wolfgang Wurst, Ali Önder Yildirim, Andreas Zimmer and Martin Hrabě de Angelis

This report was generated automatically using a specific database script. The information enclosed in this report was prepared with greatest care. However, it cannot be excluded that unintentional and unexpected formatting or spelling errors may have occurred.

About the German Mouse Clinic

The German Mouse Clinic (GMC) has opened in 2001 at the Helmholtz Zentrum München - German Research Center for Environmental Health (GmbH) in Munich to provide an open access mouse phenotyping platform for the scientific community. The GMC is part of international phenotyping efforts like the International Mouse Phenotyping Consortium IMPC (www.mousephenotype.org) and contributed to the phenotyping activities within EUMODIC (www.eumodic.org), where about 500 mutant lines from the EUCOMM resource (www.knockoutmouse.org) were analyzed in a joint effort using standardized protocols (European Mouse Phenotyping Resource of Standardised Screens, EMPReSS). In addition, the GMC plays a major role within Infrafrontier, a pan European research infrastructure for phenotyping and archiving mouse models (www.infrafrontier.eu). The GMC is supported by the Bundesministerium für Forschung und Technologie.

In the GMC, experts from various fields of mouse genetics, physiology and pathology in close collaboration with clinicians work side by side at one location. We offer a primary phenotypic analysis of mouse mutants (more than 550 parameters) in the areas of allergy, behavior, bone and cartilage, cardiovascular diseases, clinical chemistry, energy metabolism, eye development and vision, immunology, lung function, molecular phenotyping, neurology, nociception, pathology, and steroid metabolism. Secondary and tertiary screening for in-depth analysis is offered by the different screens and is available on request.

Director

Prof. Dr. Martin Hrabě de Angelis

Scientific Technical Head

Dr. Helmut Fuchs

Scientific Administrative Head

Valérie Gailus-Durner

Institute of Experimental Genetics
Helmholtz Zentrum München
German Research Center for Environmental Health (GmbH)
Ingolstädter Landstraße 1
D-85764 Neuherberg München
Tel.: 089-3187-4858
Fax: 089-3187-3500

C

Contents

1 General Part **7**
1.1 Phenotyping Summary 7
1.2 Role of the Gene (Information collaboration partner) 9
1.3 Mouse Line (Information collaboration partner) 9
1.4 Known Phenotypes (Information collaboration partner) 9
1.5 Special Conditions 9
1.6 Additional Publications 9

2 Primary Screening at the GMC **11**
2.1 Housing Conditions 11
2.2 Workflow 11
2.3 Quality Assurance 14
2.4 Statistical Analysis of Data 15

3 GMC Screening Procedures and Protocols **16**
3.1 Behavior 16
3.2 Neurology 19
3.3 Nociception 25
3.4 Dysmorphology, Bone and Cartilage 27
3.5 Metabolic Screen 31
3.6 Cardiovascular Screen 33
3.7 Eye Screen 38
3.8 Clinical Chemistry and Hematology 43
3.9 Immunology Screen 46
3.10 Allergy Screen 49
3.11 Steroid Screen 50
3.12 Lung Function Screen 52
3.13 Molecular Phenotyping 54
3.14 Pathology Screen 56

4 Phenotyping Results and Discussion **57**
4.1 General Results 57
4.1.1 Body Weight Curves and Manhattan Plots 60
4.2 Behavior 63
4.2.1 Summary 63
4.2.2 Results 63
4.2.3 Discussion 71
4.2.4 Additional References 72
4.3 Neurology 73
4.3.1 Summary 73
4.3.2 Results 73
4.3.3 Discussion 83
4.4 Nociception 84
4.4.1 Summary 84
4.4.2 Results 84
4.4.3 Discussion 86
4.5 Dysmorphology, Bone and Cartilage 87
4.5.1 Summary 87

4.5.2 Results . . . 87
4.5.3 Discussion . . . 88
4.6 Metabolic Screen . . . 89
4.6.1 Summary . . . 89
4.6.2 Results . . . 89
4.6.3 Discussion . . . 95
4.7 Cardiovascular Screen . . . 96
4.7.1 Summary . . . 96
4.7.2 Results . . . 96
4.7.3 Discussion . . . 103
4.8 Eye Screen . . . 104
4.8.1 Summary . . . 104
4.8.2 Results . . . 104
4.8.3 Discussion . . . 106
4.9 Clinical Chemistry and Hematology . . . 107
4.9.1 Summary . . . 107
4.9.2 Results . . . 107
4.9.3 Discussion . . . 125
4.10 Immunology Screen . . . 127
4.10.1 Summary . . . 127
4.10.2 Results . . . 127
4.10.3 Discussion . . . 139
4.11 Allergy Screen . . . 140
4.11.1 Summary . . . 140
4.11.2 Results . . . 140
4.11.3 Discussion . . . 142
4.12 Steroid Screen . . . 143
4.12.1 Summary . . . 143
4.13 Lung Function Screen . . . 144
4.13.1 Summary . . . 144
4.14 Molecular Phenotyping . . . 145
4.14.1 Summary . . . 145
4.15 Pathology Screen . . . 146
4.15.1 Summary . . . 146
4.15.2 Results . . . 146
4.15.3 Discussion . . . 151

5 Acknowledgements 152

6 Addresses of screeners and modules 153

References 160

Appendix 175
A Abbreviations . . . 175
B List of Figures . . . 179
C List of Tables . . . 183

General Part 1

Phenotyping Summary 1.1

Behaviour

There was decreased locomotor activity by the mutant mice in open field while the female mutant mice showed a pattern of decreased anxiety. Acoustic startle reactivity tended to be decreased in the mutant mice.

Neurology

Mutants showed more tail elevation and a trend towards reduced hearing sensitivity at 24 kHz. Three female mutants showed tremor. Grip strength, rotarod performance as well as plasma lactate were without any differences.

Nociception

We could not detect any pain related phenotype in the mutant line.

Dysmorphology, Bone and Cartilage

In the DXA analysis, we detected a significantly decreased BMC and bone content in females. Concurrently body length was decreased in females.

Metabolism

Mainly female mutants showed effects. Body mass was decreased but fat content was slightly increased. Food uptake was lower and RER shifted towards lipid oxidation. Rearing was decreased in both, male and female mutants.

Cardiovascular

By echocardiography alterations were more often observed in female mutants. Mutants had smaller left ventricle (reduced innner diameter and LV Mass). The hearts performed in general better (increased FS and EF). However, the volume of blood pumped with each beat was reduced (reduced SV).

By electrocardiography, a mild decrease in atrio-ventricular conduction time (PQ and PR interval duration) was observed.

Eye

We found significantly reduced eye sizes in Cldn12 mutants, and a decrease ($p<0,01$) of retinal thickening in the case of female mutants.

Clinical Chemistry and Hematology

IpGTT: We found slightly decreased basal fasting glucose levels in mutants com-

pared to controls, but no significant genotype effect on overnight weight loss due to food withdrawal and blood glucose response to the glucose challenge.

Clinical chemistry: No significant genotype related differences in fasting plasma lipid and glucose levels were found in Cldn12 mutant mice, but decreased plasma protein and triglyceride (trend) levels and increased creatinine values and ALP activity (very mild) in ad libitum fed mutants.

Hematology data indicated mild macrocytosis with increased MCH and decreased RBC, but increased WBC predominantly in mutant males.

In total we found mainly mild differences, that might indicate effects on energy metabolism, protein metabolism or hematopoiesis. However, since the differences are small, further investigations are recommended to confirm genotype-dependence of these findings and elucidate underlying cause.

Immunology

We did not find significant differences in the levels of antibodies, and only subtle sex-dependent changes in the frequencies of several minor leukocyte subpopulations.

Allergy

The screening did not uncover an IgE phenotype or disturbances in the skin.

Steroid

Analysis of steroids was not performed for this mouse line.

Lung

Lung Challenge has been performed and methods and results will be send separately.

Molecular Phenotyping

Heart was selected for transcriptome analysis

Pathology

Increased body weight in Cldn12 deficient male mice was detected and increased absolute and normalized liver and spleen weights were measured. Histological examination using light microscopy did not reveal any pathological changes that could be attributed to the genotype of the mice.

Role of the Gene (Information collaboration partner) 1.2

Claudin 12 is a member of the claudin-family of type II 4-transmembrane spanning proteins, which are sufficient to induce tight junction strand formation in non-epithelial cells (such as fibroblasts). Claudin 12 is different from every other claudin, it does not have a C-terminal PDZ-binding domain that is required for interaction with zonula occludens-1 (ZO-1) and ZO-2 proteins. Some claudins have sealing functions, others form channels for cations or anions and yet another claudin (Cldn 2) creates permeability to water. For several others claudins, including Cldn12, a function has not been assigned yet.

Mouse Line (Information collaboration partner) 1.3

The mutation was generated on the Velocigen platform by Regeneron Pharmaceuticals (Tarrytown, NJ). The insertion of Velocigene cassette ZEN-Ub1 created a deletion of 760 bps. This deletion replaces exon 4, coding for the entire open reading frame of Cldn12, with a LacZ cassette. The mouse line was generated via injection of the targeting vector in BL6 ES-Cells, founder lines were crossed with Zp3-Cre mice to get rid of the floxed NEO-cassette. The Cldn12 KO mouse line was backcrossed to BL6J four times prior to the GMC Primary Screen.

In the analyzed mouse line the monitoring of Cldn12 gene expression was possible, due to lacZ expression. Cldn12 LacZ activity was detected in multiple tissues of heterozygous mice. The organs with the most prominent LacZ staining were skeletal muscle, heart, liver, kidney, intestine and brain. Preliminary observations suggest expression in skeletal and smooth muscle cells.

Known Phenotypes (Information collaboration partner) 1.4

There might be a tendency for growth retardation in young null mice, although with limited penetrance.

Special Conditions 1.5

Special Workflow: Lung Challenge (methods and results will be send separately)

Additional Publications 1.6

Capture, crawl, cross: the T cell code to breach the blood-brain barriers. (2012). Engelhardt B, Ransohoff RM. Trends Immunol. 2012 Dec;33(12):579-89.

Claudin-1 induced sealing of blood-brain barrier tight junctions ameliorates chronic experimental autoimmune. (2011). Pfeiffer F, Schäfer J, Lyck R, Makrides V, Brunner S, Schaeren-Wiemers N, Deutsch U, Engelhardt B. Acta Neuropathol. 2011 Nov;122(5):601-14.

Differential roles for endothelial ICAM-1, ICAM-2, and VCAM-1 in shear-resistant T cell arrest, polarization, and directed crawling on blood-brain barrier endothelium. (2010) Steiner O, Coisne C, Cecchelli R, Boscacci R, Deutsch U, Engelhardt B, Lyck R. J Immunol. 2010 Oct 15;185(8):4846-55.

Primary Screening at the GMC — 2

Housing Conditions — 2.1

In the GMC mice are housed in GM500 cages in individually ventilated caging (IVC) systems (IVC System Green Line, Tecniplast, Italy) on wood fiber (Altromin, Lage, Germany). The IVCs operate with positive pressure. Mice are transferred in weekly intervals to new cages with forceps in Laminar Flow Class II changing stations. Mice are fed with irradiated standard rodent high energy breeding diet (Altromin 1314) and given semidemineralized filtered (0.2 µm) water ad libitum. Light is adjusted to a 12h/12h light/dark cycle; temperature and relative humidity are regulated to 22 ± 1 °C and 55 ± 5 %, respectively. In specified modules husbandry conditions are ad justed according to the experiment requirements (see corresponding sections). All people attending the facility completely change their garment (jackets and trousers autoclaved) and shoes and wear caps and masks before entering the GMC (Brielmeier et al., 2002).

Outbred 8-week-old male SPF Swiss mice are used as sentinels and kept on a mixture of new bedding and aliquots of soiled bedding (50:50) from all cages of the IVC rack. Health monitoring is carried out by on-site examination of the sentinel mice by certified laboratories according to FELASA recommendations (http://www.felasa.org).

Mice are kept according to the German laws. Tests were carried out by authority of the Regierung von Oberbayern.

Workflow — 2.2

Standardized Workflow for the Primary Screen in the German Mouse Clinic

Mice entering the GMC are examined in a primary screen according to the following standard workflow (Figure 1, modified after (Gailus-Durner et al., 2005; Brown et al., 2005; Fuchs et al., 2009; Gailus-Durner et al., 2009)). Analyzed parameters are listed in Table 1. After the mice arrive at the GMC, they are acclimatized in the new environment for two weeks and are then processed according to the workflow, which is shown below (Fig. 1).

The screening starts in the Behavior Screen. The initial screening of the experimentally naïve mice includes also neurological tests and lasts two weeks. One week later, the animals are subjected to a morphological whole-body checkup in the Dysmorphology Screen and tested in the Nociceptive Screen. Then the energy metabolism is analyzed by indirect calorimetry and the body composition is monitored by qNMR. One week later an intraperitoneal glucose tolerance test (IpGTT) is performed by the Clinical Chemical Screen. In the following week, echocardiography is performed in awake mice.

Then the mice go through the tests of the Eye Screen. In the following week, blood is taken and samples are distributed to the blood-based screens for Clinical Chemistry,

Immunology, Steroid Metabolism, and Allergy. In the next week, the mice re-enter the Dysmorphology Screen for X-ray analysis and bone densitometry by DEXA (dual energy X-ray absorption). Two weeks after testing of the first blood sample, there is the option to take a second sample to confirm the findings. In the same experimental week, 12 female animals (six mutants / six controls) were subjected to a lung function analysis. Eight males (four per genotype) are used to freeze organs for future molecular phenotyping on request. The remaining animals are analyzed macro- and microscopically in the Pathology. The analysis includes the determination of organ weights.

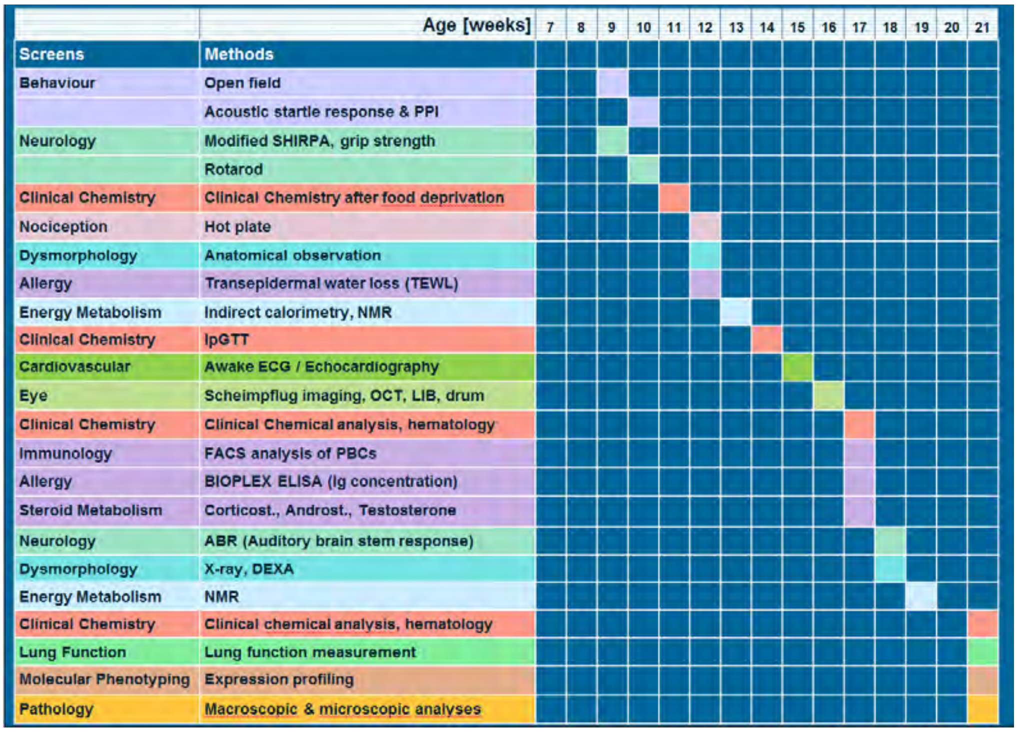

Figure 1
Workflow of the primary screen.

The accurate age of the analyzed mice and deviations from our standard operation procedure (SOP) are listed in chapter 4.1; the precise number of analyzed animals is specified in the chapter of the respective screen.

Table 1
Primary Screen at GMC

Screens	Goal	Methods
**Behavior**	locomotion and anxiety-related behavior<br>sensory motor gaiting	open field<br>acoustic startle & PPI
**Neurology**	assessment of basic neurological functions, muscle function and motor coordination/balance	modified SHIRPA protocol<br>grip strength<br>rotarod
**Nociception**	detection of altered pain response	hot plate assay
**Dysmorphology, Bone and Cartilage**	morphological analysis of body, skeleton, bone, and cartilage	morphological observation, bone densitometry, X-ray
**Cardiovascular**	assessment of functional cardio-vascular parameters	awake echo
**Eye**	assessment of morphological alterations of the eye	Scheimpflug imaging<br>optical coherence tomography<br>laser interference biometry<br>virtual drum
**Energy Metabolism**	measurement of body weight, body temperature, activity, O_2 consumption, CO_2 production, respiratory exchange ratio<br>Body composition	indirect calorimetry<br>qNMR (MiniSpec)
**Clinical Chemistry and Hematology**	determination of clinical-chemical and hematological parameters in blood<br>glucose tolerance	blood autoanalyzer,<br>ABC-animal blood counter<br>simplified IpGTT
**Immunology**	analysis of peripheral blood samples for immunological parameters	flow cytometry,<br>Bioplex multiplex bead array
**Allergy**	analysis of total plasma IgE	ELISA
**Steroid Metabolism**	analysis of steroid hormones in blood plasma: testosterone, corticosterone and androstenedione	LCMS
**Lung Function**	assessment of lung volumes and lung mechanics	Buxco FinePointeRC and Forced Maneuvers Systems
**Molecular Phenotyping**	genome-wide transcriptome analysis	Illumina Bead Array technology
**Pathology**	microscopic and macroscopic examination	histology,<br>immunochemistry

2.3 Quality Assurance

The quality assurance as part of the quality management at the GMC consists of the following elements: standardized analyses via standard operating procedures (SOP) and validation of analysis protocols by different institutions within the EUMODIC program, standardized data and project management supported by the central database system MausDB (Maier et al., 2008) and the GMC coordination tool CoordDB as well as quality control and continuous training of the staff.

Coordination of the GMC's Operations

The GMC management team (Core Facility) coordinates the scientific issues, logistics and administration of the GMC. The coordination software tool CoordDB supports the GMC management team in handling the incoming phenotyping requests and managing the complex phenotyping workflow of the primary and secondary screening. Besides the operational business activities the management team organizes the expansion of the screening services in collaboration with its partners. Additionally, the management arranges regular training of the staff members and the clinic's quality assurance.

Standardized Operation Procedures (SOP) and Validation of Protocols

The GMC developed a set of SOPs, which cover all steps from mouse import and handling to phenotyping and data analysis. These SOPs are strictly followed during the whole screening process in the GMC and all procedures are documented. The GMC is one of the major partners of the EUMODIC consortium that emerged from the EUMORPHIA program (Brown et al., 2005), a consortium for the selection, establishment, and standardization of phenotyping protocols for mice as models for human diseases and for mouse husbandry. Cross-validation of protocols by EUMORPHIA is performed by the different institutions. A collection of the protocols (EMPReSS) is posted on the EUMORPHIA web site (http://www.eumorphia.org/EMPReSS/ (Brown et al., 2005)).

Central Database System

Another tool for quality assessment is the central database system, which ensures full traceability of samples and documentation of all data. All mouse data is entered into the system (e.g. date of birth, sex, cage) and all screening results linked to the corresponding SOP as well as any changes of the mouse conditions are immediately put in (Maier et al., 2008).

Quality Control

In addition to routinely screen-specific quality control tests, control animals of selected strains (e.g. C57BL/6J, C57BL/6N and C3HeB/FeJ) are analyzed through the standard protocol for all phenotypes at regular intervals. This data is reviewed by the coordination team. A tissue archive has been established for the storage of tail and blood plasma samples taken from all mice that have ever been analyzed

in the GMC. The tail clips can be used for post-hoc genotyping in case of doubtful genotype information.

Continuous Training

Regularly specific training courses are held at the GMC. Specialists are invited to give lectures and to offer practical training at special days. Staff training is documented and maintained by the management team.

Statistical Analysis of Data 2.4

If not otherwise stated, data was analyzed using R, a language and environment for statistical computing. Tests for genotype effects were made by using t-test, Wilcoxon rank sum test, linear models, or ANOVA depending on the assumed distribution of the parameter and the questions addressed to the data. A p-value <0.05 has been used as level of significance; a correction for multiple testing has not been performed. It is necessary to note that a statistically significant difference might be on the one hand a hint for a new phenotype but might be on the other hand in another context not physiologically relevant. Depending on the effect size also statistically not significant differences might be biologically relevant. Therefore, the responsible scientist of the respective screen has evaluated and compared the data set with published data and clinical findings.

Manhattan plots

Manhattan plots, originating from GWA studies (Gibson, 2010), yield a comprehensive overview of the effect size of relevant observed parameters from all screens which represent the main disease areas based on basic statistics without the more complex and sophisticated analysis performed by the scientists of the single screens. The parameters are arranged according to their screen membership (color coded).

3 GMC Screening Procedures and Protocols

3.1 Behavior

Genetic studies in the mouse are important for the elucidation of molecular pathways underlying behavior. The goal of this endeavor is not only the identification of genes that control brain function and influence behavior, but also understanding of genetic factors involved in human psychiatric disorders (Tarantino and Bucan, 2000; Bucan and Abel, 2002).

These disorders are associated with quantitative phenotypes called ”intermediate traits” or endophenotypes, some of which, in contrast to the full complex disorder, can readily be modeled in mice. These traits are risk factors which are considered to be closer to the genetic etiology than the full syndrome. Examples are anxiety in depression, prepulse inhibition and working memory deficits in schizophrenia, and social interaction deficits in autism and schizophrenia (Seong et al., 2002; Gottesman and Gould, 2003; Inoue and Lupski, 2003).

In the attempt to efficiently screen for candidate endophenotypes within a limited time frame, we use the Open Field as a test for unconditioned behavior in rodents. The Open Field test provides a novel environment that creates a non-conditioned conflict situation between the natural tendencies of mice to explore on the one hand, and to avoid open spaces, in this case the center of the Open Field, on the other hand. Hence, it allows an evaluation of exploratory drive, reactivity to novelty and emotionality (Archer, 1973; Crawley, 1989; Weiss et al., 2000; Choleris et al., 2001).

Additionally, we use Prepulse Inhibition (PPI) to assess sensorimotor gating. PPI is considered to have face, construct, and a high predictive validity for schizophrenia and other neuropsychiatric diseases involving sensorimotor integration dysfunctions in man (Geyer et al., 2001; Swerdlow et al., 1994).

Mice

Mice were housed with food and water available *ad libitum* under standard laboratory conditions. Animals were separated based on sex, but not genotype.

Open Field (OF)

The Open Field test was carried out according to the standardized phenotyping screens developed by the EUMORPHIA partners and available at http://www.empress.har.mrc.ac

The test apparatus from ActiMot, TSE was a square-shaped frame with two pairs of light-beam strips, each pair consisting of one transmitter strip and one receiver strip. These basic light barrier strips were arranged at right angles to each other in the same plane to determine the X and Y coordinates of the animal, and thus its location (XY frame). Each strip was equipped with 16 infrared sensors with a distance between adjacent sensors of 28 mm. With two further pairs of uni-dimensional light-barrier strips (Z1 and Z2), rearing could be detected in addition to location.

The light barriers were scanned with a frequency of 100 Hz each on fast computer platforms. Whenever an even number of light beams was interrupted, the centre of gravity was calculated to lie between adjacent sensors.

The test apparatus where the mouse was placed consisted of a transparent and infrared light permeable acrylic test arena (internal measurements: 45.5 x 45.5 x 39.5 cm) with a smooth floor. The illumination levels were set at approximately 150 lux in the corners and 200 lux in the middle of the test arena.

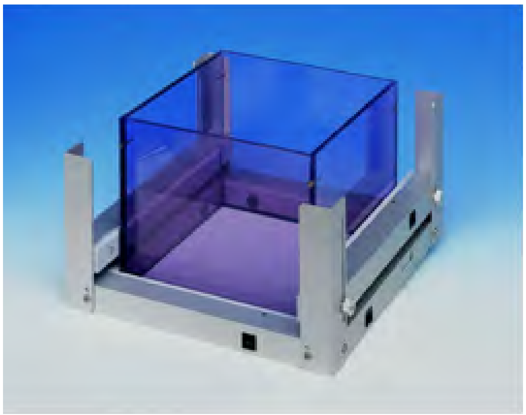

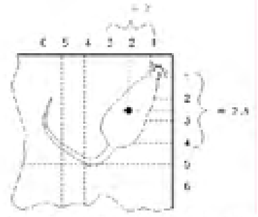

Figure 2
Test Arena for Open Field test (www.TSE-Systems.com).

At the beginning of the experiment, all animals were transported to the test room and left undisturbed for at least 30 minutes before the testing started. Then each animal was placed individually into the middle of one side of the arena facing the wall and allowed to explore it freely for 20 min. After each trial, the test arena was cleaned carefully with a disinfectant. For data analysis, the arena was divided by the computer in two areas, the periphery defined as a corridor of 8 cm width along the walls and the remaining area representing the centre of the arena (42% of the total arena in our TSE-system).

The following parameters were recorded: distance traveled, resting and permanence time as well as speed of movement for the whole arena, the periphery and the center. Additionally, rearing frequency, percentage distance traveled and percentage time spent in the center as well as the latency to first entry in center and center entry frequency were calculated. The time courses of distance traveled, rearing frequencies as well as percentage distance traveled and percentage time spent in the center were additionally analyzed in 5-min-intervals.

PPI

PPI was assessed using a startle apparatus setup (Med Associates Inc., VT, USA) including four identical sound-attenuating cubicles. The protocols were written using the Med Associates ”Advanced Startle“ software. Experiments were carried out between 08:30h and 17:00h. Background noise was 65 dB, and startle pulses were

bursts of white noise (40 msec). A session was initiated with a 5-min-acclimation period followed by five presentations of leader startle pulses (110 dB) that were excluded from statistical analysis. Trial types for the PPI included four different prepulse intensities (67, 69, 73, 81 dB); each prepulse preceded the startle pulse (110 dB) by a 50 msec inter-stimulus interval. Each trial type was presented 10 times in random order, organized in 10 blocks, each trial type occurring once per block. Inter-trial intervals varied from 20-30 sec. This protocol is based on the Eumorphia protocol (see www.eumorphia.org), adapted to the specifications of our startle equipment.

Table 2
Behaviour parameters

**Open Field behavioral phenotype evaluation**	
**Behavior**	**Measured parameters**
Forward locomotor activity	Total distance traveled in arena and periphery
Vertical exploratory activity	Rearings
Speed of movement	Average speed in arena and periphery
Immobility	Resting time in arena, centre and periphery
Anxiety-related behavior	Latency to enter centre, Percentage time in centre, Percentage centre locomotor activity, Permanence time in centre and periphery, Centre average speed, Number centre entries, Centre distance traveled
Habituation	Time courses of distance traveled, rearing frequencies and time spent in centre over the observation period

Neurology

3.2

Neurological dysfunction results in a wide variety of disorders ranging from impaired movement to severe mental illness. Studying the neurobehavioral phenotype of mutant mice is a powerful tool to understand the neural basis of behavior and the pathophysiology of neurological disorders. Comparison of the mouse and human brain transcriptomes shows a good correlation for highly expressed genes in both transcript identity and abundance (Fougerousse et al., 2000). Therefore, screening of mice with respect to neurological disorders potentially offers an understanding of etiology and pathogenesis of the human nervous system (Hafezparast et al., 2002).

The primary neurological screen is focused on investigating basic neurological functions of a mouse. Moreover, we measure grip strength to evaluate muscle performance. Since we are especially interested in mitochondria function we also measure serum lactate levels in collaboration with the Clinical Chemistry screen. For evaluation of motor coordination and balance the mice are tested with an accelerating rotarod. Hearing sensitivity is measured by recording the minimum sound pressure required to elicit a physiological response to different sound stimuli. Dependent upon results of this primary screen and due to specific questions, additional tests can be carried out for further assessment of neurological functions in a hierarchical way (Schneider et al., 2006).

Mice

First, the SHIRPA-analysis and the grip strength testing were performed and one week later the rotarod analysis was done. All animals were fed *ad libitum* during their stay in the neurological screen.

Primary screening 1: modified SHIRPA protocol

The primary observation screen is a modification of the Irwin procedure (Irwin, 1968) and was proposed as a rapid, comprehensive and semi-quantitative screening method for qualitative analysis of abnormal phenotypes in a mouse strain (Rogers et al., 1997). We examined the mice using 23 designed test parameters (See web page: http://www.har.mrc.ac.uk/services/phenotyping/neurology/shirpa.html) to detect phenotypic differences between mutant and control mice. Each test parameter contributes to an overall assessment in muscle, lower motor neuron, spinocerebellar, sensory and autonomic function and is scored qualitatively after a defined rating scale. Assessment of each animal began with observation of undisturbed behavior (Viewing Jar Behavior) in a glass cylinder (11 cm in diameter). The mice were then transferred to an arena consisting of a clear Perspex box (420 x 260 x 180 mm) in which a Perspex sheet on the floor is marked with 15 squares. Locomotor activity and motor behavior within this area was observed (Behavior recorded in the Arena). This was followed by a sequence of manipulations testing reflexes (Behavior recorded on or above the arena). Measurements were completed with the recording of body weight. The last part of the primary screen also involved the analysis of contact righting reflex. Throughout the entire procedure, abnormal behavior, biting, defecation, and vocalization were recorded. Between testing of each mouse, fecal pellets and urination were removed from the viewing jar and arena. All

experimental equipment was thoroughly cleaned with Pursept-A and dried prior to testing (Schneider et al., 2006).

Table 3
SHIRPA parameters

**Muscle/lower motor neuron function**
Body position, gait, Positional passivity, pelvic elevation, tail elevation, defecation, urination
**Spinocerebellar function**
Body position, gait, pelvic elevation, tail elevation
**Sensory function**
Transfer arousal, touch escape, gait, pinna reflex
**Autonomic function**
Palpebral closure, defecation, urination
**Neurological reflexes**
contact righting reflex, pinna reflex, corneal reflex
**General appearance**
Body weight, body position, transfer arousal, touch escape, vocalization, positional passivity, biting, spontaneous activity, locomotor activity, abnormal behavior

Primary screening 2: grip strength

The grip strength meter system determines the grip strength of the limbs, i.e. muscle strength of a mouse. The device exploits the tendency of a mouse to grasp a horizontal metal grid while being pulled by its tail. During the trial set-up, the mouse grasps a special adjustable grid mounted on a force sensor. The mouse is allowed to catch the grid with either 2 or 4 paws. Three trials were undertaken for each mouse and measurement within one minute. The mean values are used to represent the grip strength of a mouse. All experimental equipment was thoroughly cleaned with Pursept-A and dried prior subsequent tests.

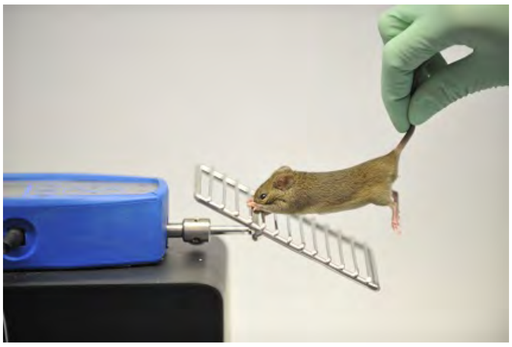

Figure 3
The grip strength apparatus

Statistical analysis of the grip strength trial results

Grip strength trial results are compared between genotypes, controlling for the effects of sex and weight, by fitting linear models (Pinheiro and Bates, 2000). A linear model is a modified analysis of variance/covariance approach allowing for dependencies in the data. In our case, dependencies arise from repeated trials within each mouse. Genotype, sex and weight are modelled as fixed effects; Interaction effects are tested for and included in the model if they show a significant contribution. The p-value for the genotype effect within the specific model found for the data indicates the significance of the statistical test of interest.

Primary Screening 3: Rotarod test

The rotarod (Bioseb, Chaville, France) was used to measure fore limb and hind limb motor coordination, balance and motor learning ability (Jones and Roberts, 1968). The machine was set up in an environment with minimal stimuli such as noise and movement.

The rotarod device is equipped with a computer controlled motor-driven rotating rod. The unit consists of a rotating spindle and five individual lanes for each mouse (Fig. 3). Magnetic sensors are used to detect when a mouse falls from the rotarod. In general, the mouse is placed perpendicular to the axis of rotation, with head facing the direction of the rotation. All mice were placed on the Rotarod at an accelerating speed from 4 to 40 rpm for 300 sec with 15 min between each trial. In motor coordination testing, mice were given three trials at the accelerating speed at one day. The mean latency to fall off the Rotarod during the trials was recorded and used in subsequent analysis. In addition, the reason for the trial end (falling, jumping or rotating passively) is recorded. Before the start of the first trial, mice were weighed.

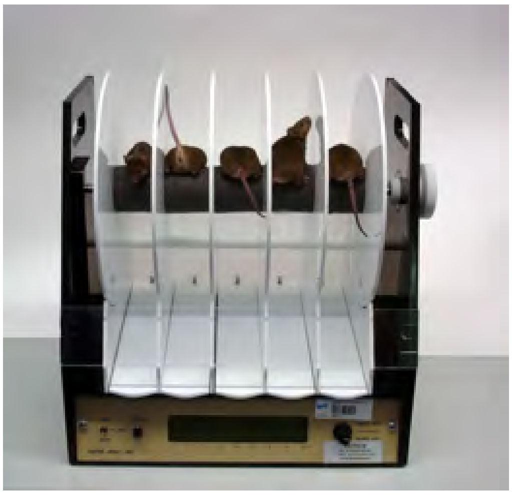

Figure 4
The rotarod apparatus (Bioseb, Chaville, France)

Statistical analysis of the Rotarod performance results

The Rotarod data contain dependencies, which are more complex than the grip

strength data. Therefore a linear mixed-effects model is used. Repeated measurements arise from three different trials with a break in between. To compare the performance results between genotypes, linear mixed-effect models are fitted, that allow for the dependencies of genotype and trial and for the effects of sex and weight. The latter are modeled as fixed effects. Interaction effects are considered and included in the model, if necessary. In each model, the parameter of interest is the coefficient of the genotype effect. A significance test or a confidence interval for this coefficient can be extracted from the model fitted.

Auditory Brainstem Response (ABR)

Auditory brainstem response (ABR) is a type of auditory evoked potential and one of the key methods in non-invasive assessment of hearing sensitivity in mice. This physiological measurement represents the electrical potentials recorded at various levels of the auditory system in response to auditory stimulation (Willott, 2001; Burkard et al., 2007). The auditory stimulus generates a response from the hair cells of the cochlea and the signal travels along the auditory pathway. This response is recorded as a series of vertex positive waves which are generated by different auditory structures. In humans, ABR allows diagnosing of various otological, audiological, and neurological abnormalities and is a part universal newborn hearing screening. In mice, similar to humans, ABR is applied for evaluation of suspected retrocochlear pathology such as acoustic neuroma, vestibular schwannoma, and hearing sensitivity. The threshold, amplitude, and latency analysis of the ABR deliver information on the peripheral hearing status and the integrity of brainstem pathways (Burkard et al., 2007).

In the primary analysis, the sound intensity thresholds are determined using different stimuli, either a broadband click or defined tone-bursts of distinct frequencies. More detailed analysis of the waveform characteristics is performed if appropriate.

Mice anaesthetised with ketamine/xylazine are transferred onto a heating blanket in the acoustic chamber and tree subcutaneous needle electrodes are placed as shown in Fig. 5 (Ingham et al., 2011). Since the stimuli are present as free-field sounds from a loudspeaker, the head of the mouse should be placed on the calibrated distance. This distance is determined by the calibration by white noise with the calibration microphone every day prior to measurements beginning.

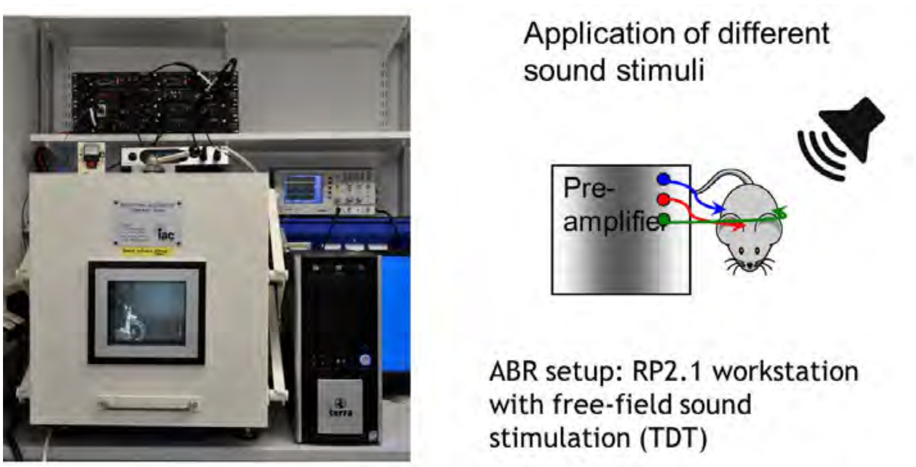

Figure 5
ABR setup: ABR RP2.1 workstation, acoustic chamber, and placement of subdermal electrodes on the skulp (blue – ground electrode, red – active electrode, green – reference electrode).

For threshold determination, the clicks (0.01 ms duration) or tone pips (6, 12, 18, 24, and 30 kHz of 5 ms duration, 1 ms rise/fall time) stimuli over a range of intensity levels from 5-85 dB SPL in 5 dB steps produced by Tucker Davis Technologies hardware with customized software, kindly provided by Welcome Trust Sanger Institute, are used. The sound intensity threshold is chosen manually from the first appearance of the characteristic waveform. For quality control, this choice is re-checked routinely by an independent observer. For each mouse, once placed in the acoustic chamber for recording, the following procedure is used:

- Initial ABR test. A response to 70 dB click broadband stimuli is recorded to ensure correct setup.
- Determination of ABR hearing threshold. A series of click-evoked ABRs is recorded in response to broadband click stimuli ranging from 0 to 85 dB SPL in 5 dB intervals.
- Determination of tone-evoked ABR thresholds. Tone-evoked ABRs are recorded for a set of frequencies (6, 12, 18, 24, and 30 kHz) over sound levels ranging from 0 to 85 dB SPL in 5 dB intervals.
- The recording of the response to 70 dB click broadband stimuli (1.) is repeated to ensure consistency of measurement.

For statistical analysis, a Wilcoxon Rank Sum test is used to analyse the thresholds of the different auditory stimuli. The audiograms normally show a characteristic pattern with higher thresholds in very low and very high frequencies. We evaluate a possible shift in this pattern using a linear mixed-effects-model with a random intercept and the frequency levels as a covariate into the analysis in addition to the effects of sex and genotype.

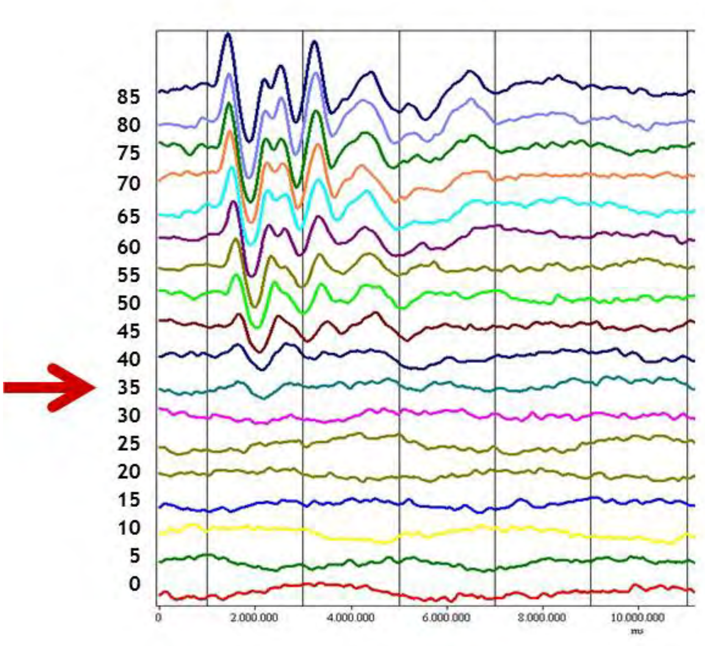

Figure 6
Click-evoked ABR traces.
ABR traces are recorded in response to clicks with 5-dB intervals from 0 to 85 dB SPL. ABR threshold is determined as the lowest stimulus level presenting any recognizable feature of the waveform (35 dB).

Nociception

3.3

Pain is the perception of an aversive or unpleasant sensation that originates from a specific region of the body. The highly subjective nature of pain is one of the factors that make it difficult to define and to treat clinically. Pain is more than a conspicuous sensory experience that warns of danger.

Nociceptors are activated by tissue injury but also by mechanical, thermal, or chemical stimuli. Harmful stimuli applied to the skin or to subcutaneous tissue, activate nociceptors, the peripheral endings of primary sensory neurons whose cell bodies are located in the dorsal root or in the trigeminal ganglia.

A noxious stimulus activates the nociceptor by depolarizing the membrane of the sensory ending. When peripheral tissues are damaged, the sensation of pain in response to subsequent stimuli is enhanced. This phenomenon termed hyperalgesia, may involve a lowering of threshold of the nociceptors or an increase in the magnitude of pain evoked by supra-threshold stimuli. Hyperalgesia can occur both at the site of tissue damage (primary hyperalgesia) and in the surrounding undamaged areas (secondary hyperalgesia; (Wall and Melzack, 1984). By means of different inbred mouse strains it could be demonstrated that rodents display large and heritable differences in both nociceptive and analgesic sensitivity (Mogil, 1999; Mogil et al., 1999; Fuchs et al., 2009; Enard et al., 2009).

Mice

In the primary screen the responsiveness of the intact somatosensory system to thermal pain was tested in animals as shown in Figure 1 by means of the hot plate test (nociceptive pain).

Hot plate test

The mice were placed on a metal surface maintained at 52 ± 0.2 C (Hot plate system was made by TSE GMBH, Germany; (Eddy and Leimbach, 1953). Locomotion of the mouse on the hot plate was constrained by 20 cm high Plexiglas wall to a circular area with a diameter of 28 cm (Fig. 3.). Mice remained on the plate until they performed one of three behaviors regarded as indicative of nociception: hind paw lick (h.p. licking), hind paw shake/flutter (h.p. shaking) or jumping.

We evaluated only hind paw but not the front paw responses, because fore paw licking and lifting are components of normal grooming behavior. Each mouse was tested only once since repeated testing leads to profound changes in response latencies. The latency was recorded to the nearest 0.1 s. To avoid tissue injury 30 s cut-off time was used. The data values are given in seconds.

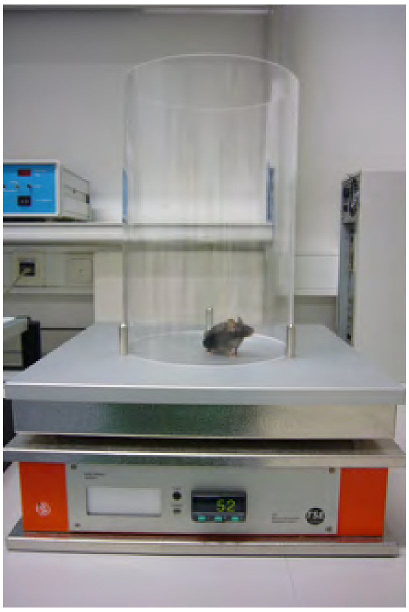

Figure 7
Hotplate system

Statistical analysis

Statistical analyses were performed using R-scripts implemented in the database (MausDB). Differences between genotypes were analyzed with ANOVAs and Tuckey's test for post hoc comparisons. Statistical significance was assumed at $p < 0.05$. Data are presented as mean values ± standard deviation.

Table 4
Nociceptive parameters

**First sign of pain:**
Reaction with mostly shaking of hind paw to the thermal pain
**Second reaction:**
Reaction with mostly licking of hind paw to the thermal pain

Dysmorphology, Bone and Cartilage 3.4

In the Dysmorphology, Bone and Cartilage Screen of the German Mouse Clinic mice are analyzed for morphological abnormalities in different organ systems with special focus on bone and cartilage development and homeostasis. The aim of the screen is to establish mouse models for human skeletal diseases like osteoporosis (McLean and Olsen, 2001; Rosen et al., 2001), scoliosis (Giampietro et al., 2003), limb defects (Mariani and Martin, 2003), osteogenesis imperfecta (Rauch and Glorieux, 2004; Chipman et al., 1993) or osteoarthritis (Abe et al., 2006).

We adapted the successful dysmorphological screening protocol from the Munich ENU-Mutagenesis Screen (Hrabě de Angelis et al., 2000) for use in the German Mouse Clinic. The nomenclature of the parameters was adapted according to the Mammalian Phenotype Ontology wording (http://www.informatics.jax.org/searches/MP_form.shtml). Further tests for defects in bone development and homeostasis were taken over from human diagnosis, and were adapted for the use in mice analysis. Such tests include: X-ray analysis, bone densitometry, and, in special cases, micro-computed tomography. Detailed protocols for screening for bone and cartilage phenotypes in mice are described by (Fuchs et al., 2006).

In secondary tests, we evaluate mutants with altered bone parameters from the primary screen in more detail using tests which include micro-computed tomography (µCT; (Schmidt et al., 2003; Turner et al., 2000)) and peripheral quantitative computed tomography (pQCT; (Gasser, 2003; Schmidt et al., 2003)), markers of bone metabolism and hormonal regulation like osteocalcin (Lee et al., 2000), fracture/stress parameters, and skeleton preparation. The aim of these tests is to further confirm our findings from the primary screen, to evaluate whether the detected phenotypes are a direct genotype-specific bone phenotype or secondary effects on bone phenotype (e.g. body size, weight, activity), and to further characterize the mutant line in more detail.

The Dysmorphology, Bone and Cartilage module of the German Mouse Clinic analyzed the mice in different phases:

- At the age of five weeks, i.e. when the mice entered the facility, the general physical condition and health were checked,
- at the age of nine weeks, a morphological observation as a whole-body checkup was performed; and
- at the age of 14 weeks, X-ray analysis and bone densitometry were performed.

Morphological Observation

The animals were screened using the protocol for morphological analysis from (Fuchs et al., 2000) as adapted for the German Mouse Clinic.

Using a clickbox (supplied by the MRC Institute of Hearing Research, Nottingham, UK) we tested the mice's ability to hear a sound of 20 kHz. The reaction of the animals was classified into six categories (0=no reaction at all, 1=no Preyer reflex,

2= retarded reaction, 3= normal reaction, 4= strong reaction, 5=particularly strong reaction).

X-ray Images

Equipment: Faxitron X-ray Model MX-20 equipped with a DC-12 digital camera (Faxitron X-ray, Illinois, USA),
Quality control: Calibration of the system is done in monthly intervals,
Settings: Automatic exposure control (AEC) selects the appropriate exposure time and kV settings for the specimen,
Procedure: The anesthetized mouse was fixed on an X-ray-permeable plate and placed in the machine. Using DX software with ImageAssistTM supplied by the manufacturer, the image was taken and analyzed. Analysis was done qualitatively by visual inspection of the images as well as quantitatively by using the ruler tool of DX software.

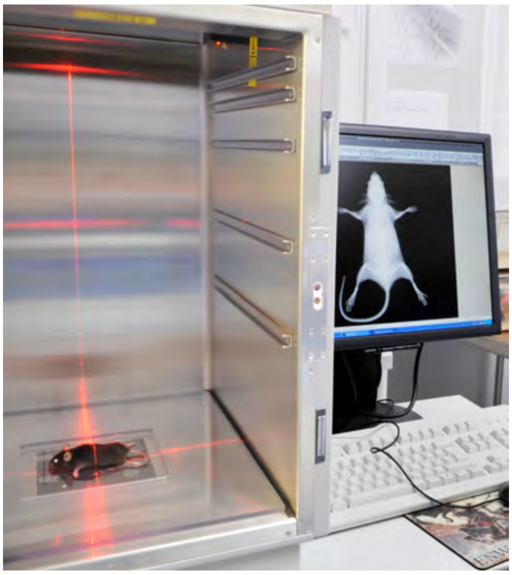

Figure 8
X-ray analysis (Faxitron X-ray Model MX-20-DC12). The red beam allows the subject to be positioned so that the area of interest is directly over the X-ray sensor.

Bone density analysis

Equipment: pDEXA Sabre X-ray Bone Densitometer (Norland Medical Systems. Inc., Basingstoke, Hampshire, UK; distributed by Stratec Medizintechnik GmbH, Pforzheim, Germany),
Quality control: Calibration of the system was done in daily intervals using the QC and the QA phantoms delivered by the manufacturer. Results from the quality control were recorded by the system.
Settings: Scan speed 20 mm/s, Resolution 0.5 mm x 1.0 mm, HAW 0.020
Procedure: After anesthesia, the weight and length of the mouse were recorded, and the mouse was placed in the analyzer. After a scout scan, the area of interest was optimized and the measure scan started.

Data-analysis: For analysis of the data, regions have to be defined. The standard analysis comprises a whole body analysis as well as a whole body analysis excluding the skull.

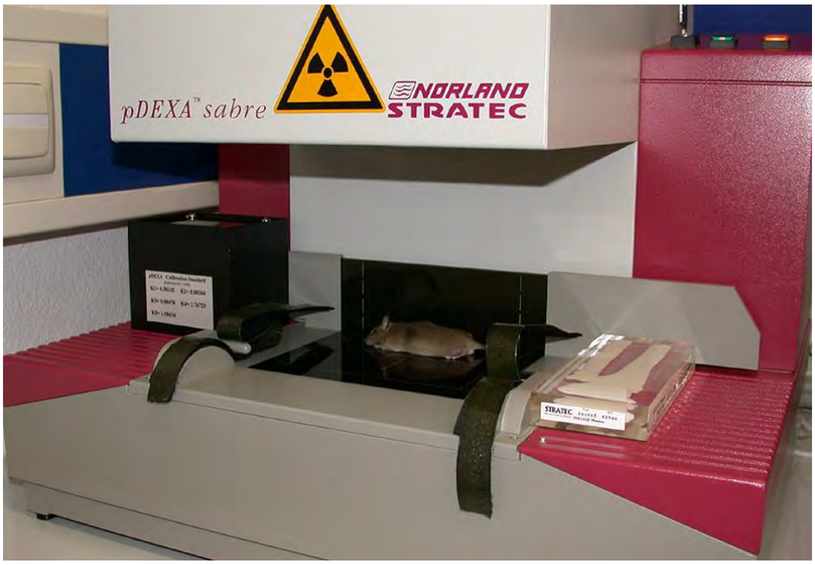

Figure 9
DEXA analysis (pDEXA Sabre X-ray Bone Densitometer)

Table 5
Dysmorphology, Bone and Cartilage parameters

**Morphological inspection**
Growth/weight/body size: abnormality
Eye: dysmorphology, corneal or lens defect
Coat: hair growth defects, hair texture defects, color anomalies, hair follicle, structure/orientation anomalies
Skin: pigmentation anomalies, texture/condition, anomalies
Vibrissae: dysmorphology
Extremities: limb dysmorphology, digit dysmorphology, tail dysmorphology
Teeth: tooth dysmorphology
Ears: auditory defects/deafness, dysmorphology
Musculature: muscle dysmorphology
Skeletal: osteogenesis/developmental anomalies, axial defects, extremities defects, craniofacial defects
Neurological / behavioral: seizures/epilepsy, motor capabilities / coordination / movement anomalies, feeding / drinking anomalies
Respiratory system: dysmorphology
Reproductive system: dysmorphology
Other aberrant phenotype
**X-ray analysis**
Skull shape, mandibles, maxilla, teeth, orbit, number of vertebrae (cervical, thoracic, lumbar, pelvic, sacral), vertebrae shape, number of ribs, rib shape, scapulas, clavicle, pelvis, femur diameter, femur shape, tibia, fibula, humerus, ulna, radius, number of digits, completeness of digits, joints
**Dual energy X-ray absorption (whole body excluding skull)**
Bone mineral density (BMD), bone mineral content (BMC), lean mass, fat mass, bone content

Metabolic Screen

3.5

The metabolic screening provides a comparative analysis of bioenergetic parameters in mice. Mechanisms which lead to disturbances in body weight regulation and energy metabolism are determined. Hence, the basal energetic demands are monitored during *ad libitum* feeding. In humans, unbalanced energy uptake and energy expenditure cause the development of obesity (Spiegelman and Flier, 2001) or anorexia nervosa with severe weight loss (Hebebrand et al., 2003). The primary metabolic screening focuses on the determination of energy expenditure by indirect calorimetry. Body mass, food intake and locomotor activity are monitored under *ad libitum* conditions. These findings serve as the origin for further investigations in secondary and tertiary screenings which go into details of energy expenditure and energy storage.

Indirect calorimetry

High precision CO_2 and O_2 sensors measure the difference in CO_2 and O_2 concentrations in air volumes flowing through control or animal cages. The amount of oxygen consumed over a given period of time can be calculated with air flow through the cage measured in parallel. Data for oxygen consumption are expressed as ml $O_2 \cdot h^{-1} \cdot animal^{-1}$. The system also monitors CO_2 production, therefore, the respiratory exchange ratio (RER) and heat production can be calculated (see below).

The RER is calculated as the ratio VCO_2/VO_2.

Heat production (HP) is calculated from VO_2 and RER using the formula:

$$HP[mW] = (4.44 + 1.43 \cdot RER) \cdot VO_2\ [ml \cdot h^{-1}]$$

The test is performed at regular room temperature (23 C) with a 12:12 hrs light/dark cycle in the room (lights on 06:00 CET, lights off 18:00 CET). Paper tissue is provided as bedding material. Each mouse is placed individually in the chamber for a period of 21 hours (from 13:00 CET to 10:00 CET next day) with free access to food and water. Metabolic cuvettes are set up in a ventilated cabinet continuously supplied with an overflow of fresh air from outside.

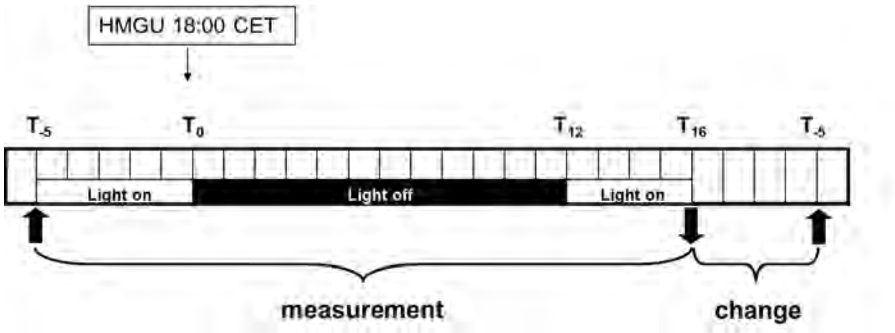

Figure 10
Indirect Calorimetry

Further data

In addition, body mass before and after gas exchange measurements are taken. Before returning the mice to their home, cage rectal body temperature is also determined. Food intake is monitored by continuously weighing food hoppers that are attached to electronic scales. Thereby, total daily food intake as well as meal size and meal duration can be determined in case genotype effects on food intake could be detected. Physical activity is measured by infrared light beam frames set up around the cages. This system allows the measurement of distance travelled and the number of rearings per time interval.

Determination of Body Composition

Most energy metabolism parameters are related to body mass and body composition. For the accurate evaluation of energy expenditure it is, therefore, of advantage to gain precise knowledge about body composition in addition to gas exchange data. Our whole body composition analyzer (Bruker MiniSpec) based on Time Domain Nuclear Magnetic Resonance (TD-NMR) provides a precise method for the measurement of lean tissue and body fat in live mice without anaesthesia. It uses TD-NMR signals from all protons in the entire sample volume and can provide data on lean and fat mass.

Statistical Analysis

Statistical analyses were performed using R-scripts implemented in the database (MausDB). Differences between genotypes were evaluated by Linear Models. Statistical significance was assumed at $p < 0.05$. Data are presented as mean values $\pm$ standard deviation.

Table 6
Metabolic parameters

**Recorded and calculated data during metabolic phenotyping**
Indirect calorimetry: Oxygen consumption (VO_2), Carbondioxide production (VCO_2), Respiratory exchange ratio (RER), heat production (HP), body mass before and after indirect calorimetry, food consumption, and rectal temperature.
Body composition analysis: body mass, fat mass and lean mass.

Cardiovascular Screen 3.6

Transthoracic echocardiography is the gold standard to determine ventricular dimensions and to identify a reduced cardiac pump function in humans and is nowadays as well established in mice (for review see (Collins et al., 2003; Syed et al., 2005)). In order to visualize the small and extremely fast beating hearts of mice the specialized high-frequency ultrasound biomicroscopy provides a high resolution and a sufficient frame rate for imaging (Zhou et al., 2004). B-mode imaging generates a 2-dimensional overview of the anatomical structures such as ventricle and large blood vessels. M-mode imaging displays the motion of tissue structures over time providing information about ventricular diameters, contractility and pump capacity. For the standard primary screening left ventricular function is evaluated by these imaging recordings.

An electrocradiogramm (ECG) is a recording of the electrical activity of the heart with surface electrodes. ECG´s are used to specifically analyse cardiac rhytms, rate, conduction and repolarization patterns within mice (Ehmke, 2003; Royer et al., 2005; Bachman et al., 2004).

As in most organ systems morphologic and anatomic alterations accompany functional abnormalities. Therefore, during the final pathologic examination the heart is inspected macroscopically and heart weight is determined and normalized to body weight and tibia length.

Echocardiography

Left ventricular function was evaluated with transthoracic echocardiography using a Vevo 2100 Imaging System (Visual Sonics) with a 30MHz probe. The echocardiographer was blinded with respect to the genotype. In order to avoid anesthetic-related impairment of cardiac function during echocardiography (Roth et al., 2002), examinations were performed on conscious animals (Schoensiegel et al., 2011). Left ventricular parasternal short and long-axis views were obtained in B-mode imaging and left ventricular parasternal short-axis views were obtained in M-mode imaging at the papillary muscle level. The short axis M-mode images were used to measure left ventricular end-diastolic internal diameter (LVEDD), left ventricular end-systolic internal diameter (LVESD), diastolic and systolic septal wall thickness (IVS) and diastolic and systolic posterior wall thickness (LVPW) in three consecutive beats according to the American Society of Echocardiography leading edge method (Sahn et al., 1978). Fractional shortening (FS) was calculated as FS%=[(LVEDD-LVESD)/LVEDD]x100. Ejection fraction (EF) was calculated as EF%=100*((LVvolD-LVvolS)LVvolD) with LVvol=((7.0/(2.4+LVID)*LVID^3). The corrected left ventricular mass (LV MassCor) was calculated as LV MassCor=0.8 (1.053 * ((LVIDD + LVPWD + IVSD)^3 - LVIDD^3)). The Stroke volume (SV) is the volume of blood pumped from one ventricle of the heart with each beat. The Stroke volume of the left ventricle was obtained by substracting end-systolic volume (ESV) from end-diastolic volume (EDV). In addition, heart rate and respiratory rate were calculated by measuring three systolic intervals, respectively three respiratory intervals.

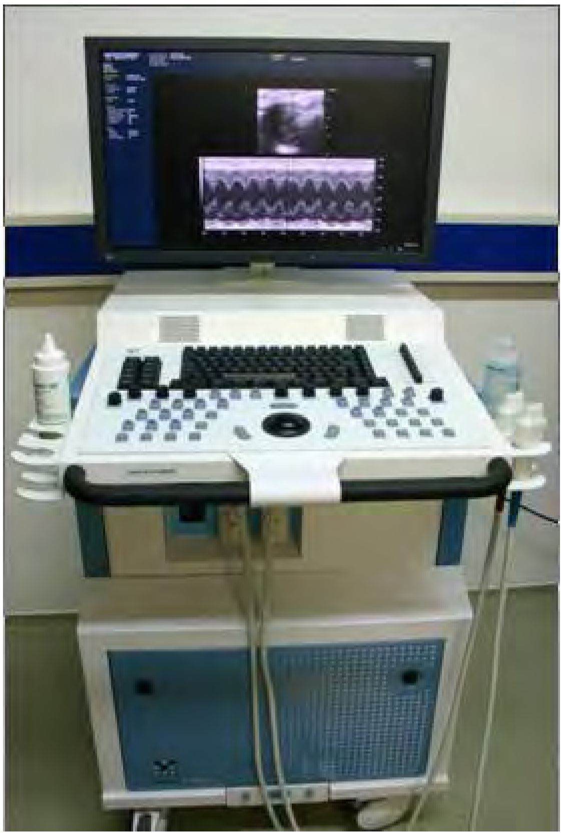

Figure 11
Vevo®2100 High-Resolution Imaging System

Table 7
Echocardiography parameters and abbreviations

Interventricular septum in systole	IVSs [mm]
Interventricular septum in diastole	IVSd [mm]
Left ventricular posterior wall in systole	LVPWs [mm]
Left ventricular posterior wall in diastole	LVPWd [mm]
Left ventricular internal dimension in systole	LVIDs [mm]
Left ventricular internal dimension in diastole	LVIDd [mm]
Fractional shortening	FS [%]
Ejection fraction	EF [%]
Stroke volume	SV [µl]
Left ventricular mass corrected	LV MassCor [mg]

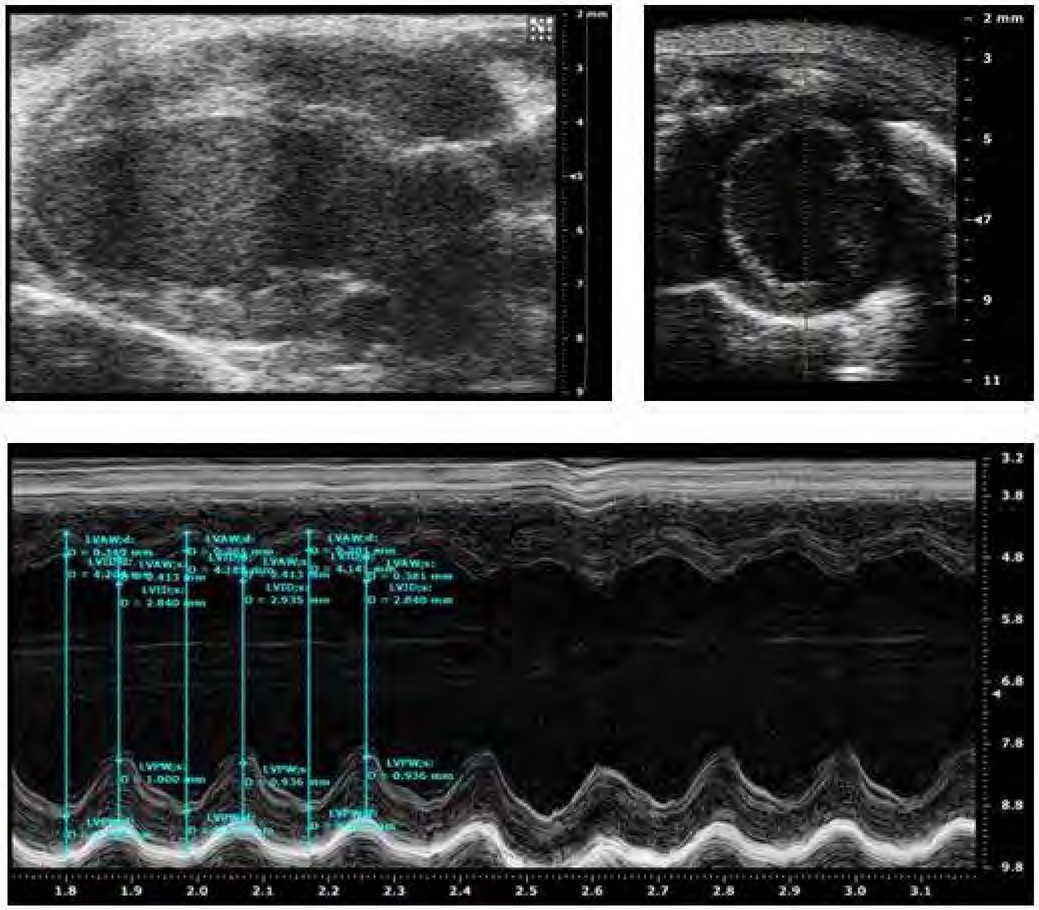

Figure 12
Echocardiographic measurements in conscious mouse, screenshots

Electrocardiography

ECG´s were recorded in conscious mice with the ECGenie (Mouse Specificy Inc., Boston, MA) and analysed using e-Mouse software (Mouse Specifics Inc.) The cardiac electrical activity was detected non-invasively through the animals´ paws. The size and arrangement of the electrodes are configured to advance contact with three of the animals' paws to provide an ECG signal that is equivalent to Einthoven lead II. For each animal, intervals and amplitudes were evaluated from continuous recordings of at least 15 ECG signals. e-MOUSE software uses peak detection to calculate the heart rate (HR). HR variability (HRV) is calculated as the mean of the differences between sequential HRs. The software plots its interpretation of P, Q, R, S, and T for each beat so that unfiltered noise or motion artifacts are rejected. This is followed by calculations of the mean of the ECG time intervals for each set of waveforms. The corrected QT interval (QTc) is calculated by dividing the QT interval by the square root of the preceeding RR interval. QT dispersion was measured as inter-lead variability of QT intervals. The QTc dispersion was calculated as the rate corrected QT dispersion.

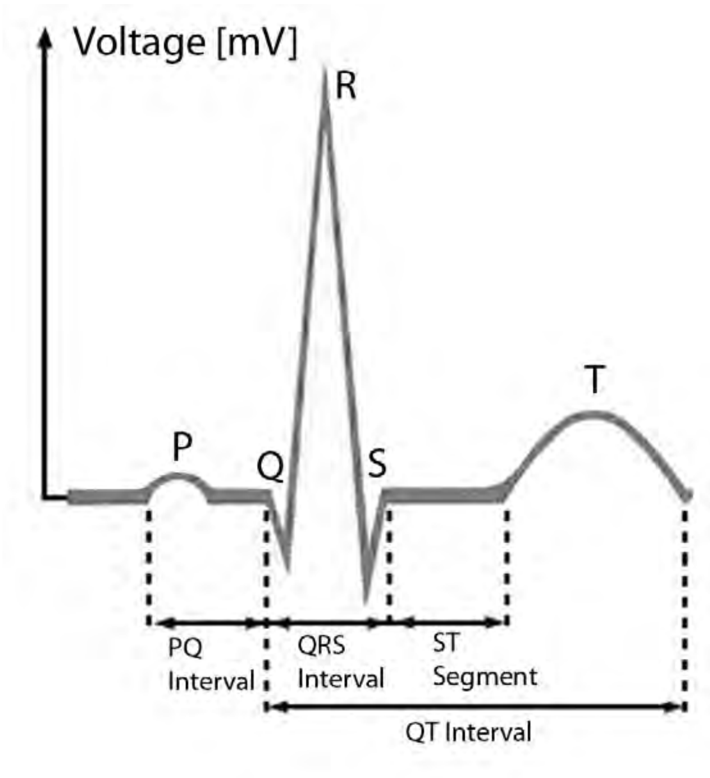

Figure 13
Example of ECG trace with analysed parameters

Table 8
Echocardiography parameters and abbreviations

Heart rate HR [bpm]
Heart rate variability HRV [bpm]
RR interval [ms]
PQ interval [ms]
PR interval [ms]
QRS complex duration [ms]
QT interval [ms]
ST segment [ms]
QTc corrected QT interval [ms]
QT-dispersion [ms]
QTc-dispersion [ms]
SR amp [mV]
R amp [mV]

Heart Weight Determination

During the final examination in the Pathology Screen the heart weight was deter-

mined together with body weight and tibia length. Briefly, mice were sacrificed by CO_2 inhalation, weighed and opened from the ventral midline. Exsanguination was achieved by cutting the dorsal aorta. Prior to dissection the heart was inspected for abnormalities or excessive fat. For excision the heart was removed from the pericardial membrane and the major vessels were cut through at the point they enter or exit the atria. The heart weight was obtained wet after blotting the organ on paper towels. The tibia length was determined from the left tibia of the mouse using a digital caliper “MarCal” (Mahr GmbH; Göttingen, Germany).

Table 9
Heart Weight Analysis

Heart Weight
Body Weight
Tibia Length
Heart Weight per Body Weight Ratio
Heart Weight per Tibia Length Ratio

Analysis of data

The data were analyzed statistically with R-scripts. Wilcoxon rank sum tests for the detection of genotype effects were performed for all animals together and for each sex separately.

3.7 Eye Screen

In the primary screen, different methods were employed to analyze the eyes of mutant mouse line in comparison to their control littermates. Mice were examined for anterior segment abnormalities by Scheimpflug Imaging, as well as for posterior segment abnormalities by Optical Coherence Tomography. The axial eye length was measured by Laser Interference Biometry (Puk et al., 2006). Furthermore, we studied visual properties in vision tests with the Virtual Drum (Prusky et al., 2004).

If required, Secondary Eye Screens can be performed. These include histological studies and/or analysis of retinal functionality by electroretinography (ERG; (Dalke et al., 2004)).

In humans blindness is caused by several different ocular diseases. Among these, the cataracts are responsible for half of all cases (Johnson and Foster, 2003). The retinal disorders cover a broad variety of clinical symptoms and many different genes are involved in the corresponding pathological conditions in humans. The two most important groups are retinitis pigmentosa (RP) and age-related-macular-degeneration (ARMD; for recent reviews, see (Rivolta et al., 2002; Stone et al., 2001). Mouse models are appropriate tools to understand the genetic and biochemical mechanisms of ocular disorders. There is a rapid increasing number of mouse mutants available suffering from various types of eye diseases (for recent reviews see (Graw, 2003; Dalke and Graw, 2005).

Mice

Mice entered the Eye Screen at the age depicted in Table 1. Mice were kept under standard laboratory conditions with food and water *ad libitum*. For Laser Interference Biometry and Optical Coherence Tomography, mice were anaesthetized with 137 mg Ketamine and 6.6 mg Xylazine per kg body weight. Eyes were further treated with 1% Atropine to ensure pupil dilation. When the mice were killed for pathological examinations, the eyes of some mice were fixed for histological analysis in the eye screen.

Laser Interference Biometry (LIB)

Eye size measurement was performed using the “AC Master” (Meditec, Carl Zeiss, Jena, Germany). Briefly, anaesthetized mice were placed on a platform and orientated in an appropriate position using light signals from six infrared LEDs arranged in a circle that must be placed in the center of the pupil. Central measurements of axial eye length were performed essentially as described (Schmucker and Schaeffel, 2004).

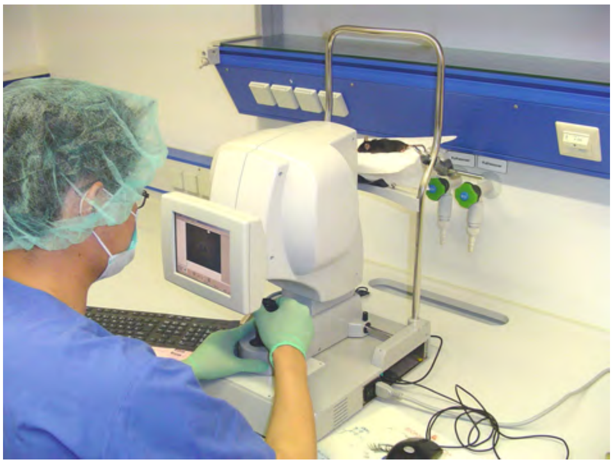

Figure 14
AC Master (Meditec, Carl Zeiss, Jena, Germany)

Optical Coherence Tomography

Eye fundus and retina were analyzed with a Spectralis OCT (Heidelberg Engineering, Heidelberg, Germany) modified with a 78 diopter double aspheric lens (Volk Optical, Inc., Mentor, OH, USA) that is fixed directly to the outlet of the device. To the eye of the mouse, a contact lens with a focal length of 10 mm (Roland Consult, Brandenburg, Germany) was applied with a drop of methyl cellulose (Methocel 2%, OmniVision, Puchheim, Germany). For measurements, anaesthetized mice were placed on a platform in front of the Spectralis OCT such that the eye is directly facing the lens of the recording unit. Images were taken as described previously (Fischer et al., 2009). Retinal thickness was calculated with the provided thickness profile tool.

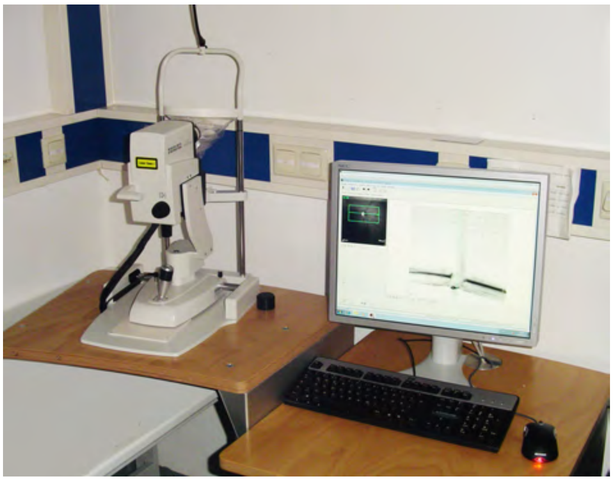

Figure 15
Spectralis OCT (Heidelberg Engineering, Heidelberg, Germany)

Scheimpflug Imaging

Images of corneas and lenses were taken with the Pentacam digital camera system (Oculus GmbH, Wetzlar, Germany). Mice were hold on a platform such that the vertical light slit (light source: LEDs, 475 nm) was orientated in the middle of the eye ball. Distance between eye and camera was fine adjusted with the help of the provided software in order to guarantee optimal focus. Subsequently, measurements were started manually. Mean density across the lens was quantified with the provided densitometry tool.

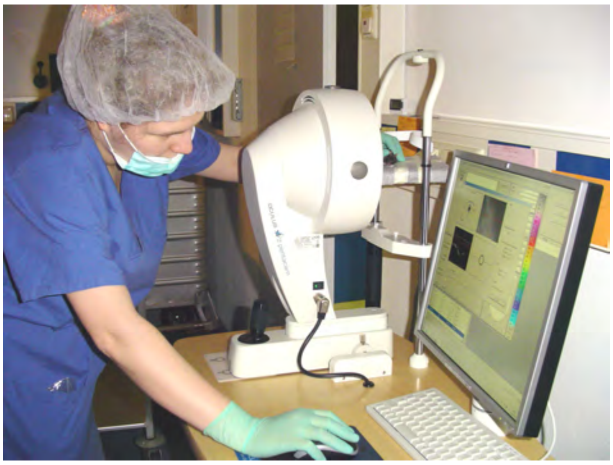

Figure 16
Pentacam digital camera system (Oculus GmbH, Wetzlar, Germany)

Virtual vision test

Vision tests were performed between 9 am and 4 pm using a virtual optomotor system (Cerebral Mechanics, Lethbridge, Canada) as described previously (Prusky et al., 2004). Briefly, a rotating cylinder covered with a vertical sine wave grating was calculated and drawn in virtual three-dimensional space on four computer monitors facing to form a square. Visually unimpaired mice track the grating with reflexive head and neck movements (head-tracking). Vision threshold of the tested mice was quantified by a randomized simple staircase test. Rotation speed and contrast were set to 12.0 d/s and 100%, respectively.

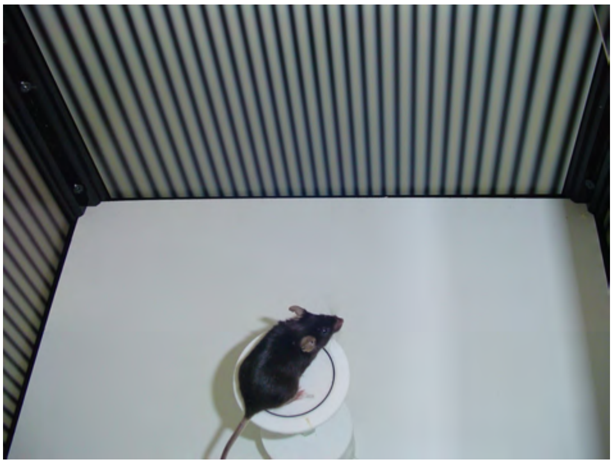

Figure 17
Vision test using a virtual optomotor system (Cerebral Mechanics, Lethbridge, Canada)

Electroretinography (ERG)

Ganzfeld ERGs were recorded simultaneously from both eyes to examine retinal functionality as described (Dalke et al., 2004). In brief, mice were dark-adapted for at least twelve hours and anaesthetized. Individual mice were fixed on a sled and gold wires (as active electrodes) were placed on the cornea. The ground electrode was a subcutaneous needle in the tail. A reference electrode was placed subcutaneously between the eyes. Mice were introduced into an ESPION ColorBurst Handheld Ganzfeld LED stimulator (Diagnosys LLC, Littleton, MA, USA) on a rail to guide the sled (High-Throughput Mouse-ERG, STZ for Biomedical Optics and Function Testing, Tübingen, Germany). 10 ms light pulses were delivered at a frequency of 0.48 Hz and a light intensity of 12.500 cd/m^2. Responses were recorded with an ESPION Console (Diagnosys LLC, Littleton, MA, USA).

Histology

Eyes were fixed 24 hours in Davidson solution, dehydrated and embedded in plastic medium. Transverse 2 µm sections were cut with an ul-tramicrotome, stained with methylene blue and basic fuchsin and evaluated with a light microscope.

Statistical Analysis

Medians, first and third quartile, and p-values were calculated by a Wilcoxon rank-sum test. Statistical significance was set at $p < 0.05$.

Table 10
Eye parameters

**Laser Interference Biometry (LIB)**
axial eye length
**Optical Coherence Tomography**
number of main blood vessels (fundus); retinal thickness; (qualitative) morphology of retinal layers
**Scheimpflug Imaging**
mean lens density; (qualitative) morphology of cornea and lens
**Virtual vision test**
spatial frequency threshold
**Electroretinography**
a-wave and b-wave amplitude
**Histology**
(qualitative) retinal lamination and morphology of cell layers and lens
**Morphology**
(qualitative) like size and degree of closure

Clinical Chemistry and Hematology 3.8

The aim of the Clinical-Chemical Screen is the detection of hematological changes, defects of various organ systems, and changes in metabolic pathways and electrolyte homeostasis by means of suitable laboratory diagnostic tools. Since most inherited metabolic disorders are known to lead directly or indirectly, via altered organ functions, to changes in the parameters investigated, this screening process provides a comprehensive investigation of clinical phenotypes with counterparts in humans and animal species (Rathkolb et al., 2000). The methods used are routine procedures, allowing the appropriate screen of large numbers of mice for a broad spectrum of clinical-chemical and hematological parameters (Champy et al., 2004; Hough et al., 2002).

A broad selection of different clinical-chemical parameters was measured including various enzyme activities, as well as plasma concentrations of specific substrates and electrolytes. Additionally, we determined basic hematological parameters and tested the animals for glucose tolerance. In selected lines we also measure blood lipid and glucose values in samples collected after overnight food-withdrawal.

Mice

All animals (usually 15 mice per sex and genotype) were used subsequently for glucose tolerance testing and determination of clinical chemical and hematological parameters in the ad libitum fed state.

Intraperitoneal Glucose-Tolerance-Test

Mice were used for the glucose tolerance test after a 16-18 hours-lasting overnight food-withdrawal. In the beginning of the test, the body weight of mice was determined. For the determination of the fasting blood glucose level, the tip of the tail was scored using a sterilized scalpel blade and a small drop of blood was analyzed with the Accu-Chek Aviva glucose analyzer (Roche/Mannheim). Thereafter mice were injected intraperitoneally with 2 g of glucose/kg body weight using a 20 % glucose solution, a 25-gauge needle and a 1-ml syringe. 15, 30, 60 and 120 minutes after glucose injection, additional blood samples (one drop each) were collected and used to determine blood glucose levels as described before. Repeated bleeding was induced by removing the clot from the first incision and massaging the tail of the mouse. After the experiment was finished, mice were placed in a cage with plentiful supply of water and food.

Blood Withdrawal and Storage

Blood samples were taken from isoflurane-anesthetized mice by puncturing the retro-bulbar sinus with non-heparinized glass capillaries (1.0 mm in diameter; Neolab; Munich, Germany). The time of sample collection was recorded in a work list. Blood taken after overnight food-withdrawal was collected in heparinized sample tubes (Li-heparin, KABE; Nümbrecht, Germany; Art.No. 078028), blood sample collected from *ad libitum* fed mice were divided into two portions. The major portion was collected in a heparinized tube (Li-heparin, KABE; Nümbrecht, Germany;

Art.No. 078028). The smaller portion was collected (using the same capillary) in an EDTA-coated tube (KABE, Art.No 078035). Each tube was immediately inverted five times to achieve a homogeneous distribution of the anticoagulant.

Samples collected from unfed mice were stored in a rack on ice, separated by centrifugation (10 min, 5000 × g; 8 C, Biofuge fresco, Heraeus; Hanau, Germany) as soon as possible and plasma was used for clinical-chemical analysis. Heparinized blood collected from *ad libitum* fed mice was left in a rack at room temperature for one to two hours. Afterwards, cells and plasma were separated by a centrifugation step (10 min, 5000 × g; 8 °C, Biofuge fresco, Heraeus; Hanau, Germany). Plasma was distributed between the Immunology Screen (30 µl), the Allergy Screen (30 µl), the Clinical Chemical Screen (110 µl) and the Steroid Screen (50 µl), while the cell pellet was given to the Immunology Screen for FACS-analysis.

The plasma samples for the clinical chemical analyses were transferred into Eppendorf tubes and either used immediately (plasma of unfed mice) or diluted 1:2 with aqua dest. (ad libitum fed mice). The solution was mixed for a few seconds (Vortex genie, Scientific Industries; New York, USA) to prevent clotting and then centrifuged again for 10 min at 5000 x g at 8 °C. In addition, the Clinical Chemical Screen received the EDTA-blood samples for hematological investigations, which were placed on a rotary agitator at room temperature until analysis.

Clinical Chemistry

The screen was performed using a Beckman-Coulter AU 480 autoanalyzer and adapted reagents from Beckman-Coulter (Krefeld, Germany), except free fatty acids (NEFA) that were measured using a kit from Wako Chemicals GmbH (NEFA-HR2, Wako Chemicals, Neuss, Germany) and Glycerol, which was measured using a kit from Randox Laboratories GmbH (Krefeld, Germany). In the primary screen, a broad set of parameters (Table 8) was measured including various enzyme activities, as well as plasma concentrations of specfic substrates and electrolytes in ad libitum fed mice. A set of six measured parameters and one calculated value (blood lipid and glucose levels) is determined in samples derived from mice after overnight food-withdrawal, if requested.

Hematology

A volume of 50 µl EDTA-blood was used to measure basic hematological parameters with a blood analyzer, which has been carefully validated for the analysis of mouse blood (ABC-Blutbild-Analyzer, Scil Animal Care Company GmbH; Viernheim, Germany). Number and size of red blood cells, white blood cells, and platelets are measured by electrical impedance and hemoglobin by spectrophotometry. Mean corpuscular volume (MCV), mean platelet volume (MPV) and red blood cell distribution width (RDW) are calculated directly from the cell volume measurements. The hematocrit (HCT) is assessed by multiplying the MCV with the red blood cell count. Mean corpuscular hemoglobin (MCH) and mean corpuscular hemoglobin concentrations (MCHC) are calculated from hemoglobin/ red blood cells count (MCH) and hemoglobin/ hematocrit (MCHC), respectively.

Second sample analysis

An additional sample was collected on request two to four weeks after the first test from a subgroup of mice (usually 10 per sex and genotype) in order to retest a subset of parameters to check the reproducibility of the first results or to measure additional blood parameters.

Analysis of Data

Data were statistically analyzed using R-Scripts. Depending on the distribution of the respective data parametric or non-parametric statistical methods are used.

Table 11
Clinical chemical parameters

**Proteins and plasma enzyme activities**
Alkaline phosphatase (ALP; EC 3.1.3.1), α-Amylase (EC 3.2.1.1), Aspartate-aminotransferase (ASAT/ GOT; EC 2.6.1.1), Alanine-aminotransferase (ALAT/ GPT; EC 2.6.1.2), Creatine kinase (CK; EC 2.7.3.2); Lactate-Dehydrogenase (LDH; EC 1.1.1.27), Total protein, Albumin
**Plasma concentrations of specific substrates**
Glucose, Cholesterol, Triglycerides, Urea, Creatinine (enzymatic methods)
**Plasma concentrations of electrolytes**
Potassium, Sodium, Chloride, Calcium, Inorganic phosphate, Iron
**Clinical chemical parameters determined in samples collected from overnight fasted mice**
Cholesterol, HDL-cholesterol, Triglycerides, non-esterified fatty acids (NEFA), Glycerol, Glucose, additionally calculated: non-HDL-Cholesterol from Cholesterol minus HDL-Cholesterol
**Basic hematology**
White blood cell count (WBC), Red blood cell count (RBC) Hematocrit (HCT), Hemoglobin (HGB), Mean corpuscular volume (MCV), Mean corpuscular hemoglobin (MCH), Mean corpuscular hemoglobin concentration (MCHC), Red blood cell distribution width (RDW), Platelet count (PLT) and Mean platelet volume (MPV)
**Intraperitoneal Glucose-Tolerance-Test (IpGTT)**
Blood glucose concentration after 16 to 18 hours fasting (time 0) as well as 15 min, 30 min, 60 min, 90 min and 120 min after glucose administration

3.9 Immunology Screen

Mouse models have been a primary source of information for understanding the intricate mechanisms of the immune system (Bluethmann H., 1994; Mak et al., 2001; Fischer, 2002; Rogner and Avner, 2003). The Immunology Screen at the GMC was set up to conduct a broad immunological phenotyping of mouse mutant lines with the intention of identifying distinct gene functions, which play key roles in the immune defenses of the organism through a complex network of cellular and soluble components (Janeway et al., 2004). In primary screen we measure leukocyte populations in peripheral blood and immunoglobulin levels in blood plasma.

The proportions of leukocyte populations in peripheral blood are genetically regulated (e.g., Mice: (Chen and Harrison, 2002); Men: (Hall et al., 2000)). As a consequence, inbred strains differ in the frequency of leukocyte subsets in the lymphoid organs and in peripheral blood. Moreover, several CD antigens are restricted to specific mouse strains (e.g., (Carlyle et al., 2006)) or interstrain differences occur concerning the level of expression of certain CD antigens (e.g., (Haegel and Ceredig, 1991)). Strain specific differences in the immune response are further reflected in different susceptibilities to infectious agents (e.g., (Medina et al., 2001)).

In individual mice, the number of circulating leukocytes and the proportions of subpopulations show daily rhythmic variations (Yellon and Tran, 2002) and depend further on homeostatic proliferation and/or retraction (Freitas and Rocha, 2000), as well as on activation through environmental and/or microbial factors (e.g., (Grewal et al., 1997)), which might be related to subtle behavioral characteristics (e.g., (Kim et al., 1999)). Furthermore, sex-dependent factors are documented to influence the immune status (Krzych et al., 1978) and have an impact on infection susceptibility (Pasche et al., 2005).

The levels of Ig classes and IgG isotypes are characteristic of a special inbred mouse strains and seem to underlie genetic control mechanisms (Sant'Anna et al., 1985).

Mice

The blood samples are taken applying the same procedure which is described for the Clinical-Chemical Screen (please see chapter 3.8).

Peripheral blood leukocytes (PBLs)

The PBLs are characterized from three whole blood samples à 10-20 µl per mouse. Each whole blood sample is incubated at 4-10°C for 5 minutes with Fc block (clone 2.4G8.14; Elisabeth Kremmer, Helmholtz-Zentrum München). Then, the corresponding antibody mix is added and incubated at 4-10°C for 15-60 minutes. For the characterization of T-and B- cell subsets, an erythrocyte-lysis and a formalin-fixation (BD FACS Lysing solution, Becton Dickinson, USA) is performed after the antibody incubation. Finally, after washing with FACS staining buffer (PBS, 0.5 % BSA, 0.02 % sodium azide, pH 7.45) samples are acquired from a 96 well plate using a HyperCyt sampler (IntelliCyt Corp., USA) and a 10-color flow cytometer (Gallios, Beckman Coulter, USA). The acquisition threshold is set on the CD45-channel (Weaver et al.,

2002).

Our analyses are based on approximately 10.000- 50.000 leukocytes per sample. Frequencies of leukocyte populations are determined by software based analysis (Flowjo, TreeStar Inc, USA; and SPICE (Roederer et al., 2011)). The frequencies of main leukocyte subsets are referred to the CD45+ gate (see figure; please note, that in our protocol the majority of CD45 negative cells are already excluded via the CD45 threshold). Gates for each parameter are based on formerly performed 'fluorescence minus one' (FMO) controls (Baumgarth and Roederer, 2000).

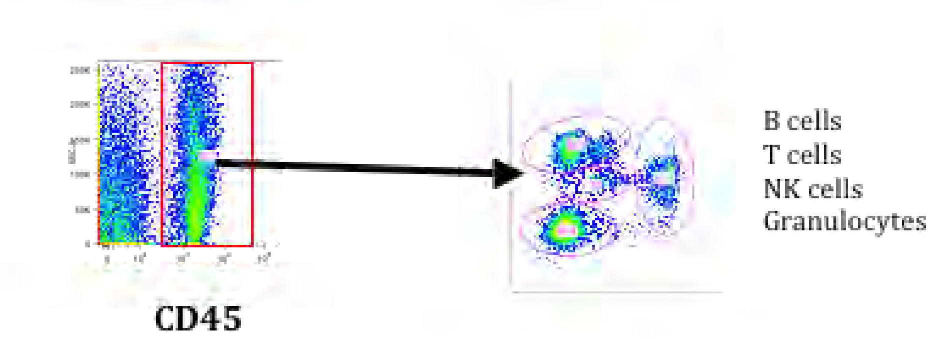

Figure 18
Frequencies of main lineages refer to a CD45+ gate

Immunoglobulins

The plasma levels of IgM, IgG1, IgG2a, IgG2b, IgG3, and IgA were determined simultaneously in the same sample using a bead-based assay (Fulton et al., 1997) with monoclonal anti-mouse antibodies conjugated to beads of different regions (Biorad, USA), and acquired on a Bioplex reader (Biorad).

The presence of rheumatoid factor and anti-DNA antibodies was evaluated by indirect ELISA with rabbit IgG (Sigma-Aldrich, Steinheim, Germany) and calf thymus DNA (Sigma-Aldrich), respectively, as antigens and AP-conjugated goat anti-mouse secondary antibody (Sigma-Aldrich). Serum samples from MRL/MpJ-Tnfrsf6lpr mice (Jackson Laboratory, Bar Harbor, USA) were used as positive controls in the autoantibody assays.

Table 12
Parameters measured by the Immunology Screen

**Flow cytometry**
Measured parameters and defined cell populations
Staining 1: main leukocyte populations and monocyte subpopulations
B1-B cells (CD5+, CD19+/B220+), B2-B cells (CD5-, CD19+B220+), T cells (CD3+/CD5+ NK-), granulocytes (CD11b+/Gr-1+), NK cells (CD5-, NKp46+ and/or NK1.1+), NK T cells cells (CD5+, NKp46+ and/or NK1.1+), monocytes (non NK cells, non granulocytes, CD11b+)<br><br>Monocyte subpopulations: CD11c (positive or negative), Ly6C (positive, very high positive, negative)
Staining 2: T cell subpopulations (CD5+NKp46-/NK1.1-)
CD4+CD8-, CD8+CD4-, CD4-CD8-, CD4+CD8+, γδTcells (γδ-T cell receptor+)<br><br>Further subsets are subdivided based on the expression of CD25 (this marker characterized Treg cells within the CD4+CD8- T cell comparment), CD44, Ly6C and CD62L
Staining 3: B cell subpopulations (CD19+):
The B cell cluster is subdivided concerning the expression on CD5, B220, MHC class-II, IgD, IgM, CD21 and CD23
**Bioplex**
IgG1, IgG2a (not in C57BL/6 background strain), IgG2b, IgG3, IgM, IgA
**Elisa**
Anti-DNA, rheumatoid factor (anti-rabbit immunoglobulin)

Allergy Screen

The goal of the Allergy screen within the German Mouse Clinic (GMC) is to search for IgE mutants in order to establish mouse models for allergic diseases and to find new strategies for antiallergic therapy. IgE-mediated atopic disorders such as allergic asthma, allergic rhinitis, and atopic dermatitis are now considered as environmentally and exposure driven immune disorders leading to the expression of various clinical phenotypes in individuals with defined genetic risk profiles (Arruda et al., 2005; Kabesch, 2006). Genome screens with classical linkage and fine mapping approaches suggest that susceptibility to asthma is determined by multiple genes with each gene having a moderate dose effect (Wjst et al., 1999). In this respect, the development of phenotypically and genotypically defined animal models will be an important step (Bochner and Busse, 2005). To detect allergy prone mouse mutants in systematic screening efforts, total plasma IgE was established as a powerful screening parameter (Alessandrini et al., 2001; Jakob et al., 2008).

Blood samples were taken by puncturing the retroorbital plexus under isoflurane anesthesia (please see chapter 3.8).

ELISA

Plasma was analyzed for total IgE, using a classical immunoassay isotype-specific sandwich ELISA. In brief, microtiter plates (96-well) were coated with 10 µg/ml anti-mouse-IgE rat monoclonal IgG (clone-PC284, The Binding Site) to detect total IgE. Serum samples were diluted 1:10 and standards for murine IgE (Mouse IgE, k clone C38-2 BD Pharmingen) were appropriately diluted. As secondary antibodies, biotinylated rat anti-mouse IgE (clone R35-118, BD Pharmingen) were used followed by incubation with BD OptEIA Reagent Set B (Cat. No. 550534 BD Pharmingen) Plates were analyzed using a standard micro well ELISA reader at 450 nm. Total murine IgE data are reported in ng/ml, based on a standard curve of purified murine IgE (Alessandrini et al., 2001).

Transepidermal Water Loss (TEWL)

Transepidermal Water Loss is defined as the measurement of the quantity of water that passes from inside a body through the epidermal layer (skin) to the surrounding atmosphere via diffusion and evaporation processes. Measurements of TEWL are useful for identifying skin damage caused by pathological conditions such as eczema, certain chemicals, or physical insult, as rates of TEWL increase in proportion to the level of damage.

Mouse skin is analysed in a non-invasive manner with a special Tewameter (AquaFlux AF200) that is placed on the skin. During a short time of 60 to 90 seconds TEWL [g/(m^2h)] is recorded.

3.11 Steroid Screen

Steroids control differentiation and proliferation processes of cells and tissues (Prehn et al., 2009). They participate in the regulation of apoptosis (Bansal et al., 1991), bone remodeling (Jerome, 2004) and neuroregeneration (Chowen et al., 2000). Several severe diseases such as congenital adrenal hyperplasia (Simard et al., 1993) or pseudohermaphoditism (Andersson et al., 1991) are caused by monogenic mutations with loss of function of steroid pathway proteins. Defects in steroid metabolism contribute as well to pathogenesis of many different complex diseases like cancer, diseases of cartilage and bone or neurological diseases (Mindnich et al., 2007; Möller and Adamski, 2006; Prehn et al., 2009). Corticosterone is a very important effector for endocrine diseases like diabetes (Alberts et al., 2005; Atanasov and Odermatt, 2007; Morton and Seckl, 2008; Seckl et al., 2004).

The main focus of the Steroid Screen is the identification of new animal models for human steroid-related diseases therewith supporting the development of their future medical treatment. For primary screening the key steroids corticosterone, testosterone, and androstenedione (Labrie et al., 1995) are quantified from plasma by LC-MS/MS.

These steroids were chosen for the tests because of the: 1) robustness of the assay, 2) applicability to mouse model, 3) lack of circadian or other rhythm, 4) indicative value for steroid biosynthesis pathways.

There is a high variability of steroid concentrations between individuals that means the biological range is very broad. Due to this and the low concentrations of plasma steroids, concentration differences can only be considered as significant when they differ in order of magnitude. Generally there are only limited published reference concentrations for the steroids in the rodents. For corticosterone in mouse was reported to be about 10 ng/mL (30 nmol/L) (Bartolomucci et al., 2009), for mouse testosterone about 5 ng/mL (20 nmol/L) (Erben et al., 2002), and for (female) mouse androstenedione about 0.5 ng/ml (2 nmol/L) (Mayer et al., 2004).

Mice

Male and female mice were screened for alterations in plasma concentrations of corticosterone, testosterone and androstenedione. Blood was collected retro-bulbar from narcotised mice (isoflurane) and plasma was prepared by centrifugation and stored at -20 C.

Sample preparation and LC-MS/MS

Steroids were quantified from 50 μL mouse plasma by validated high-throughput LC-MS/MS method using HPLC (Shimadzu Prominence) and Triple Quadrupol Mass Spectrometer (4000 QTrap, ABSciex). Concentrations were calculated upon the calibration from the respective standard curve using stable isotope labelled internal standards and are reported in nmol/L. The sensitivity of the tests is 2.8 nmol/L for Corticosteron and 0.4 nmol/L for testosterone and androstenedione each. Sam-

ple preparation and LC-MS/MS measurement is fully described in our publication in Nature Protocols (Haller et al., 2010).

3.12 Lung Function Screen

Introduction

Pulmonary function tests are important tools to describe the phenotypic characteristics of a respiratory disease or a genetically caused respiratory disorder. Using invasive pulmonary function devices it is possible to perform spirometric measurements on mice and quantify lung disease via changes in various volume and flow parameters, which has been demonstrated in animal models of diseases like emphysema or cystic fibrosis ((Vanoirbeek et al., 2010; Kneidinger et al., 2011; Hector et al., 2011)).

Mice

Female wild-type control and mutant mice were examined.

Pulmonary Function Testing

We used two different invasive pulmonary function devices (both from Buxco Research Systems; Wilmington, NC, USA): a forced maneuver system and a FinePointe RC system.

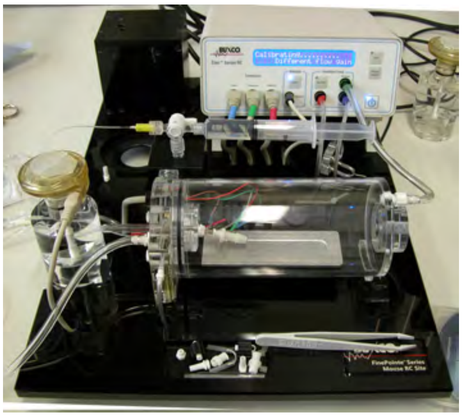

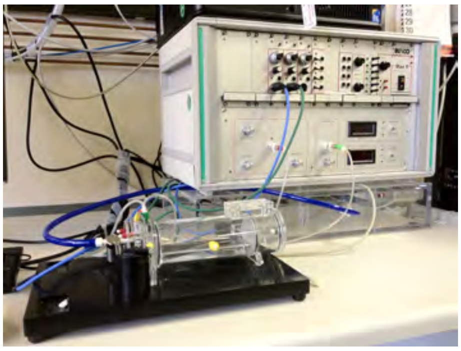

Figure 19
Buxco FinePointe RC system (left) and Buxco Forced maneuvers system (right).

All mice were anesthetized by i. p. injection of Ketamin (137 mg/kg BW) and Xylazin (6.6 mg/kg BW). After opening the trachea by a small incision, an 18-gauge cannula was inserted and fixated by ligation. The mice were then placed in a FinePointe RC system. In a heated plethysmograph chamber, mice were ventilated at an average rate of 160 breaths per minute, and flow and mouth pressure and heart rate were monitored to measure resistance and dynamic compliance. Beginning with an initial acclimation period of three minutes, a two-minute measurement was performed. After the resistance (R) and compliance (Cdyn) measures, mice were transferred to a forced pulmonary maneuvers system. Here, the forced residual capacity (FRC) was determined during spontaneous breathing of the mouse using Boyle's Law, then quasistatic pressure volume (PV) and fast flow volume (FV) maneuvers were run three times each and averaged to obtain all lung volume and flow parameters. The PV test uses a slow expiration phase after inflating the mouse to total lung capacity (TLC)

to obtain quasistatic chord compliance (Cchord), TLC, expiratory reserve volume (ERV), residual volume (RV) and inspiratory capacity (IC). The FV test applies a fast expiration after inflation to TLC, thereby measuring forced vital capacity (FVC), peak expiratory flow (PEF) and forced expiratory volume at 100 ms (FEV100).

Measurements were always performed between 8 a.m. and 1 p.m. The system was set up in a quiet room where temperature and humidity were kept constant throughout the measurements. Before each measurement, the system was calibrated and the actual barometric pressure and temperature to warrant adequate calculations of flow rates and volumes.

Statistical Analysis of Data

Statistical analyses were performed using R-scripts implemented in the database (MausDB). Differences between genotypes were evaluated by Wilcoxon test. Statistical significance was assumed at $p < 0.05$. Data are presented as mean values ± standard deviation.

Table 13
Lung parameters and abbreviations

BW	Body Weight [g]
Cchord	Chord Compliance [ml/cm H_2O]
Cdyn	Dynamic Lung Compliance [ml/cm H_2O]
ERV	Expiratory Reverse Volume [ml]
FEV100	Forced Expiratory Volume [ml]
FRC	Functional Residual Capacity [ml]
FVC	Forced Vital Capacity [ml]
IC	Inspiratory Capacity [ml]
PEF	Peak Expiratory Flow [ml/s]
R	Lung Resistance [cm H_2O/ml/s]
RV	Residual Volume [ml]
TLC	Total Lung Capacity [ml]
TV	Tidal Volume [ml]
VC	Vital Capacity [ml]

3.13 Molecular Phenotyping

Comparative genome-wide expression profiling is a powerful tool in the effort to annotate the mouse genome with biological functions. The analysis of RNA expression data reveal target genes directly or indirectly regulated by a genetic modification, affected molecular pathways, groups of co-expressed genes and potential targets for therapy. We demonstrated the feasibility to detect transcriptional affected organs employing RNA expression profiling as a tool for molecular phenotyping (Seltmann et al., 2005; Horsch et al., 2008).

Mice

The molecular phenotyping screen archives organs of mutant and control mice for subsequent DNA-chip expression profiling analysis. Usually eight male mice (four mutants and four controls) were provided to the molecular phenotyping screen.

To minimize the influence of circadian rhythm on gene expression, mice were killed between 9 and 12 am by carbon dioxide asphyxiation. The listed organs were collected and archived in liquid nitrogen following our established SOPS: spleen, kidney, liver, heart, thymus, skeletal muscle, pancreas and brain. Organs were immediately frozen and stored in liquid nitrogen until isolation of total RNA. Pancreas was stored at 4°C in RNAlater buffer (Qiagen) for 1-2 days. Afterwards the tissue was removed from the buffer and stored at -80°C. Organ samples collected in this collaboration may either be used for expression profiling analysis in the GMC or on request send to the collaboration partner. The storage time for the organs is limited to approximately 3-4 years.

RNA isolation

Total RNA was isolated just before processing the microarray experiment. For preparation of total RNA individual organs were thawed in 4 ml Trizol Reagents (Sigma) and homogenized using a Polytron homogenizer (Heidolph). Total RNA from individual samples were obtained from a 1 ml aliquot of the homogenate according to manufacturer's protocol using RNeasy Mini Kits (Qiagen). Another 2 ml homogenate was stored at -80°C as backup. 2 µg RNA aliquots were run on a formaldehyde agarose gel to check for RNA integrity. The concentration was measured by a Nanodrop device. The RNA was stored at -80°C. The storage time for the RNA is limited to approximately 2-3 years.

Illumina Bead Arrays

For each selected organ one Illumina Bead Array enabling the performance of eight samples in parallel, was processed. Usually, four biological replicates for each genotype group were performed. Therefore, 500 ng of total RNA was amplified in a single round with the Illumina TotalPrep RNA Amplification Kit (Ambion). 750 ng of amplified RNA was hybridized on Illumina MouseRef8 v2.0 Expression Bead Chips containing about 25K probes (25.600 well-annotated Ref-Seq transcripts). Staining and scanning (Illumina HiScan Array reader) was done according to the Illumina expression protocol.

Statistical analysis

Illumina Genomestudio 2011.1 software was used for background correction and normalization of the data (cubic spline). The remaining negative expression values were corrected by introducing an offset. The identification of significant gene regulation was performed using SAM (Significant Analysis of Microarrays) included the TM4 software package (Horsch et al., 2008; Saeed et al., 2003; Tusher et al., 2001). Genes were ranked according to their relative difference value d(i), a score assigned to each gene on the basis of changes in gene expression levels relative to the standard deviation. Genes with d(i) values greater than a threshold were selected as significantly differentially expressed in an one class analysis. The percentage of such genes identified by chance is the false discovery rate (FDR). To estimate the FDR, nonsense genes were identified by calculation 1000 permutations of the measurements. The selection of the top differentially expressed genes with reproducible up- or down-regulation includes less than 10% false positives (FDR $<$ 10) in combination with fold change $>$ 1.4.

Functional analysis

Ingenuity Pathway analysis (IPA, http://www.ingenuity.com) and/or Genomatix Pathway System (GePS, http://www.genomatix.de) may be utilized to identify over-represented Gene Ontology (GO) terms and regulated pathways among the differentially expressed genes. Further literature-based associations between the regulated genes were detected by IPA and GePS analysis. All relationships were graphically represented as a network diagram.

3.14 Pathology Screen

The anatomic pathological evaluation (gross and histological analysis) plays a vital role in the final analysis of mutant mouse models and provides insights into the understanding how genetic alterations influence the development of human diseases that the mutant mice are intend to model (Brayton et al., 2001). The morphological phenotyping of the mutant mice can identify anatomical changes introduced by the mutation of interest. In addition, it can detect unexpected phenotypes inherent to the mouse strain or related to environmental influences and simultaneously facilitate to establish a correlation between the anatomical abnormalities and the functional abnormalities found in other screens.

Furthermore, in cases in which the morphological data alone are not enough to reach a conclusive diagnosis, immunohistochemistry is a crucial technique to identify specific proteins on paraffin- or frozen- processed tissue sections. The positive immunostaining can be then combined with image analysis software in order to quantitatively analyze the data.

Transmission electron microscopy is another important tool in the pathology screen to examine the substructure and ultrastructure of individual cells in scenarios where conventional light microscopy identifies minor abnormalities despite clear evidence of disease (Stirling et al., 1999; Saarikangas et al., 2011).

Recently, the pathology screen has implemented the virtual slide microscopy, which enables the diagnosis and the archiving of tissue sections for discussion both remotely or in online conferences, facilitating worldwide consultations and discussions.

Macroscopial and histological analyses

Mice received in the laboratory of pathology were sacrificed with CO_2. The animals were analyzed macroscopically and weighed (http://eulep.pdn.cam.ac.uk/Necropsy_of_the_Mou[illegible] The thymus and left lobe of the liver were measured with a digital caliper (MarCal, Mahr GmbH; Göttingen, Germany). Blood samples were taken, centrifuged and the serum was saved at -20 C. Tails were preserved at -70 C for further genetic analysis. Following a complete dissection, an x-ray of the complete bone structure was taken, when indicated (Hewlett Packard, Cabinet X-Ray System Faxitron Series). All organs were fixed in 4 % buffered formalin and embedded in paraffin for histological examination. Two-µm-thick sections from skin, heart, muscle, lung, brain, cerebellum, thymus, spleen, cervical lymph nodes, thyroid, parathyroid, adrenal gland, stomach, intestine, liver, pancreas, kidney, reproductive organs, and urinary bladder were cut and stained with hematoxylin and eosin (H&E).

Phenotyping Results and Discussion 4

General Results 4.1

Table 14
Results in a nutshell.

Screen	Parameter set	Phenotype quality	Short description
Dysmorphology, Bone and Cartilage	Morphological observation	no	
Dysmorphology, Bone and Cartilage	DXA	subtle	Decreased BMC and bone content in females; decreased body length in females
Dysmorphology, Bone and Cartilage	X-Ray	no	
Behaviour	Open Field	yes	Decreased locomotor activity and pattern of decreased anxiety in the female mutant mice
Behaviour	Prepulse Inhibition / Acoustic Startle Reactivity	subtle	Tendency to decreased acoustic startle reactivity
Neurology	Modified SHIRPA	yes	More tail elevation
Neurology	Grip Strength	no	
Neurology	Rotarod	no	
Neurology	Lactate	no	
Neurology	Auditory Brainstem Response	no	
Eye Screen	Eye Size	yes	Reduced axial eye lenghts in the Cldn12 mutants
Eye Screen	OCT	no	
Eye Screen	Scheimpflug	no	
Eye Screen	Virtual Drum	na	
Nociception	Hotplate	no	
Metabolic Screen	Indirect Calorimetry (TSE)	subtle	Mainly differences in females, lower body mass, lower food intake, lower RER, less rearing both in male and female mutants
Metabolic Screen	Minispec	subtle	Lower body mass, increased fat content
Clinical Chemistry and Hematology	Clinical Chemistry (ad lib. fed mice)	yes	Decreased plasma protein and triglyceride (trend) levels and increased creatinine values and ALP activity (very mild) in mutants
Clinical Chemistry and Hematology	Hematology	yes	Mild macrocytosis with increased MCH and decreased RBC, but increased WBC predominantly in mutant males
Clinical Chemistry and Hematology	ipGTT	subtle	Slightly decreased basal fasting glucose levels in mutants
Clinical Chemistry and Hematology	Clinical Chemistry (fasting values)	subtle	Subtle increase in non-HDL cholesterol that could be accidental; Slightly increased glucose and decreased NEFA in female mutants

Screen	Parameter set	Phenotype quality	Short description
Immunology Screen	Flow Cytometry	subtle	Sex-dependent subtle changes within the monocyte (females, males), and the CD4 T cell, B cell and NK cell compartment (males)
Immunology Screen	Immunoglobulins	no	
Allergy Screen	IgE levels (new)	no	
Allergy Screen	TEWL	no	
Cardiovascular Screen	Echo	yes	Reduced LVID, LV Mass and SV; Increased FS, EF, HR and RR mainly in female mutants
Cardiovascular Screen	Electrocardiography	no	Decreased PQ and PR intervals mainly in females
Pathology Screen	Macroscopy	yes	Increased body weight in Cldn-12 deficient male mice was detected and increased absolute and normalized liver and spleen weights were measured
Pathology Screen	Microscopy	no	

Table 15
Number of analyzed mice.

sex	genotype	number
female	control	19
female	mutant	16
male	control	15
male	mutant	16

Table 16
Age of mice when the specific test has been applied.

GMC Module	Parameterset	age in weeks	age in days	age range
Behaviour	Open Field	10	69	67-71
Neurology	Modified SHIRPA	10	69	67-71
Neurology	Grip Strength	10	69	67-71
Neurology	Rotarod	11	76	74-78
Behaviour	Acoustic startle and PPI	11	76	74-78
Clinical Chemistry	Clinical Chemistry (fasting values)	12	83	81-85
Clinical Chemistry	Blood Sampling Fasted	12	83	81-85
Nociceptive	Hotplate	13	90	88-92
Allergy	Transepithelial water loss	13	90	88-92
Dysmorphology	Dysmorphology	13	90	88-92
Metabolism	Minispec MRI	14	97	95-99
Metabolism	Calorimetry TSE	14	97	95-99
Clinical Chemistry	Simplified IPGTT	15	104	102-106
Cardiovascular	ECG	16	111	109-113
Cardiovascular	Echocardiography	16	111	109-113
Clinical Chemistry	Clinical Chemistry (ad. lib. fed mice)	18	125	123-127
Clinical Chemistry	Hematology	18	125	123-127
Immunology	Determination of immunglobuline levels in blood (except for IgE)	18	125	123-127
Allergy	Immunglobulin E measurement	18	125	123-127
Immunology	Frequencies of main leukocyte and monocyte populations in peripheral blood (% of CD45+ cells,% of monocytes, flow cytometric measurement)	18	125	123-127
Clinical Chemistry	Blood Sampling	18	125	123-127
Immunology	Frequencies of T cell and NK cell subpopulations in peripheral blood (% of T/NK cells, flow cytometric measurement)	18	125	123-127
Immunology	Frequencies of B cell subpopulations in peripheral blood (% of B cells, flow cytometric measurement)	18	125	123-127
Dysmorphology	DEXA	19	132	130-134
Dysmorphology	X-Ray	19	132	130-134
Neurology	Auditory brainstem response	19	132	130-134
Metabolism	Minispec MRI	20	139	137-141
Eyes	Eye Size	21	146	144-148
Eyes	Optical coherence tomography	21	146	144-148
Eyes	Scheimpflug analysis	21	146	144-148
Eyes	Virtual Drum	21	146	144-148

4.1.1 Body Weight Curves and Manhattan Plots

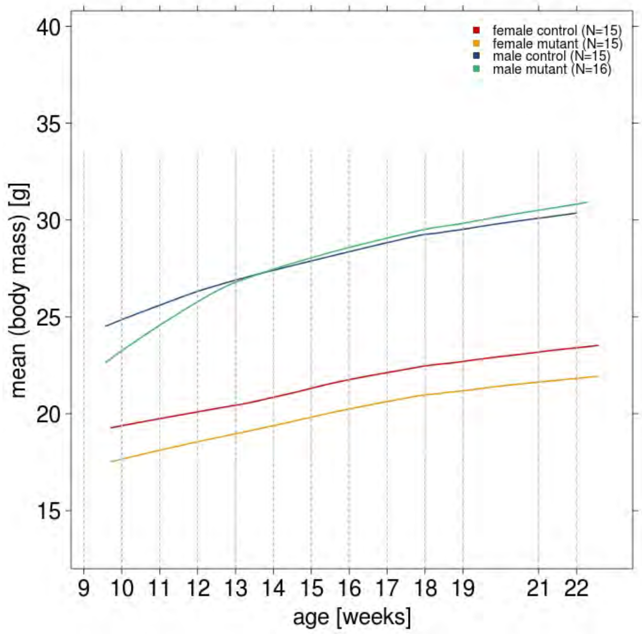

Figure 20
Body mass: Time curve of the mutant and control cohorts.

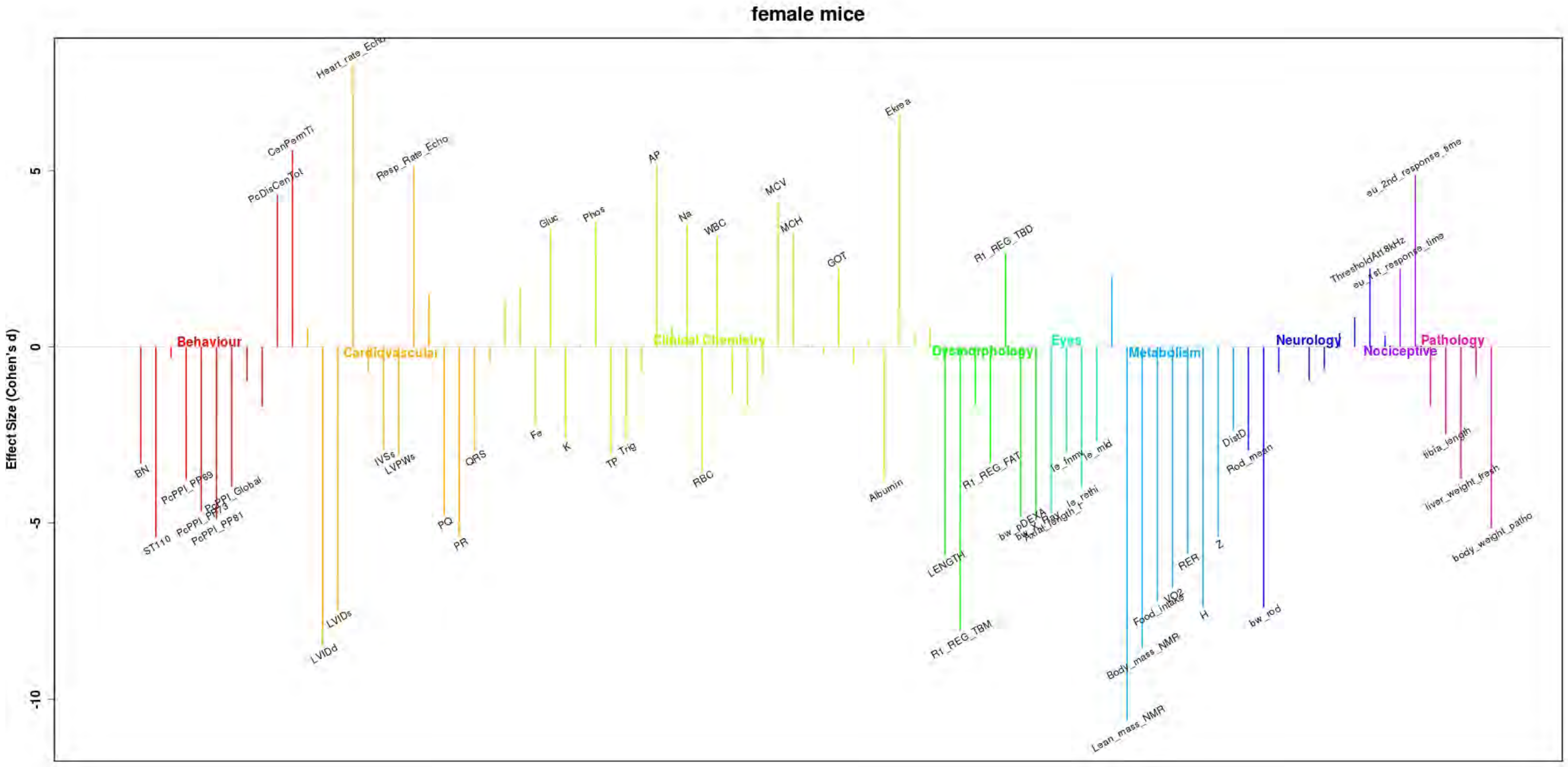

Figure 21
Manhattan Plot of Effect Sizes: Parameters are arranged according to the subsets of a screen (color coded). Each parameter is evaluated by the observed effect size.
Positive values of effect size *d* indicate an increased average value for mutants compared to controls. Negative values of effect size *d* indicate a decreased average value for mutants compared to controls.

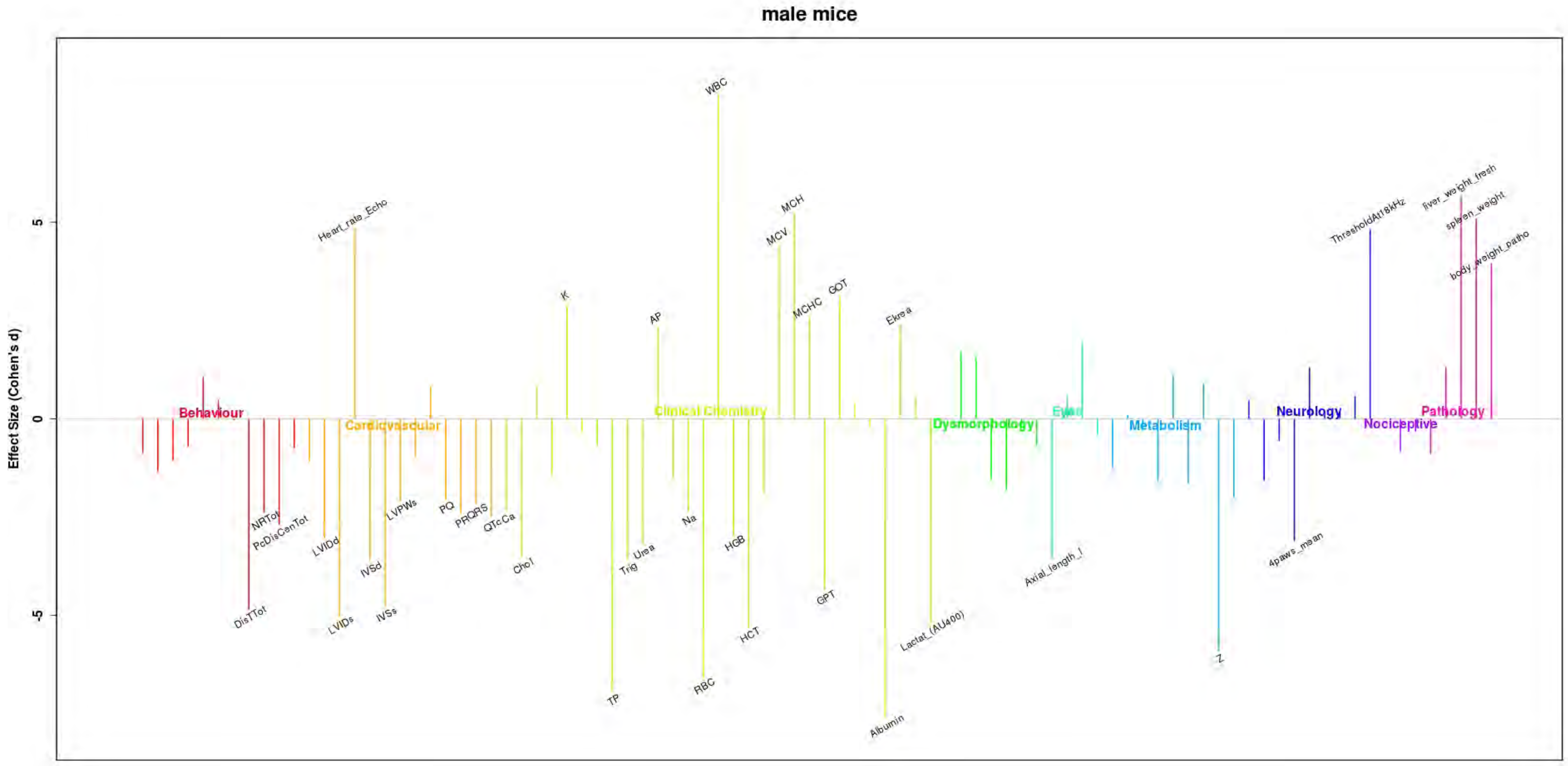

Figure 22
Manhattan Plot of Effect Sizes: Parameters are arranged according to the subsets of a screen (color coded). Each parameter is evaluated by the observed effect size.
Positive values of effect size *d* indicate an increased average value for mutants compared to controls. Negative values of effect size *d* indicate a decreased average value for mutants compared to controls.

Behavior 4.2

Summary 4.2.1

There was decreased locomotor activity by the mutant mice in open field while the female mutant mice showed a pattern of decreased anxiety. Acoustic startle reactivity tended to be decreased in the mutant mice.

Results 4.2.2

Open Field

In the open field, the mutant mice showed significantly decreased total distance travelled. Total rearing activity was not significantly different between the groups however there was a decrease in the mutant mice during the last 5 minutes of the test.

In terms of anxiety-related behavior, there was an increase in centre time and distance in the female mutant mice with a pattern of the opposite effect for the males. Centre resting time was also increased for the female mutant mice as was the number of entries into the centre.

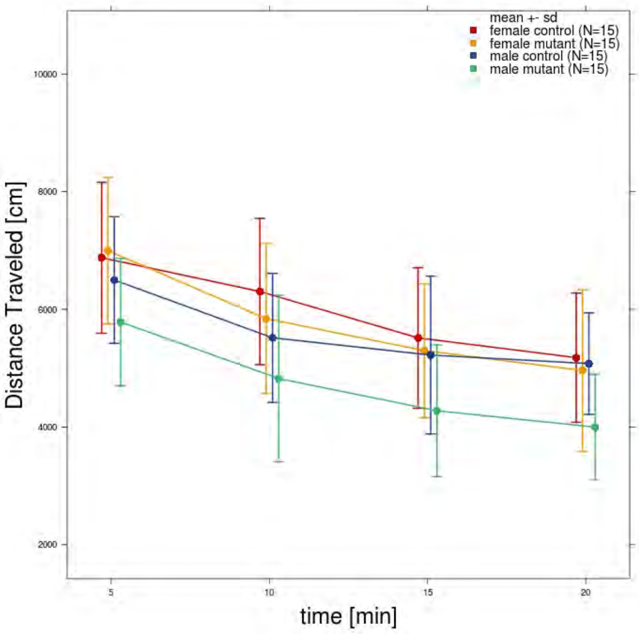

Figure 23
Open Field: Distance Traveled, average plot, split by sex and genotype

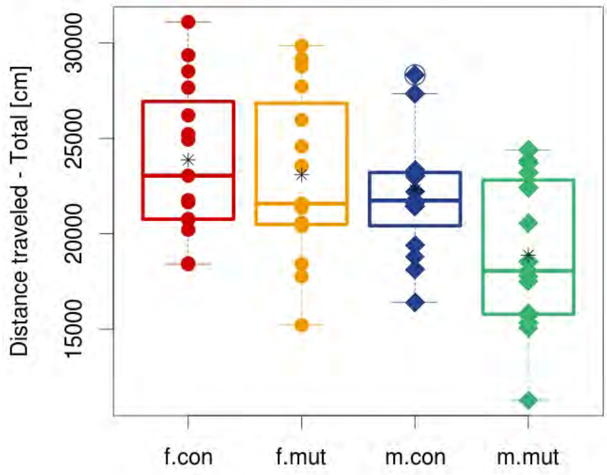

Figure 24
Open Field: Distance traveled - Total boxplot with stripchart, split by sex and genotype

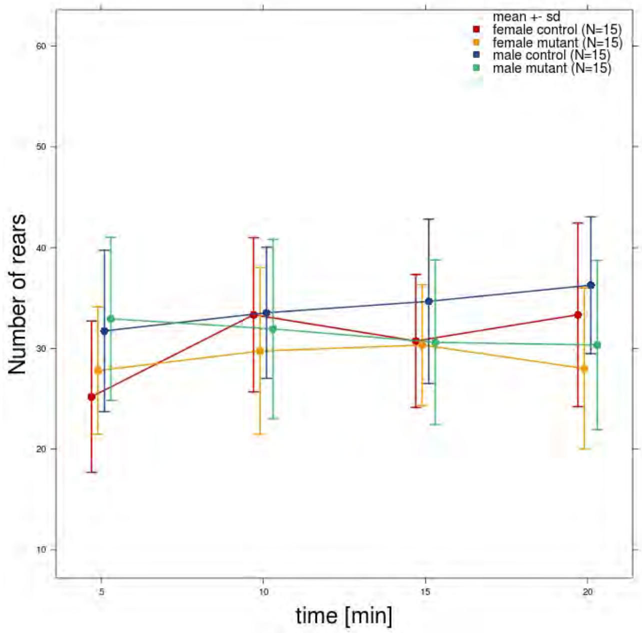

Figure 25
Open Field: Number of rears, average plot, split by sex and genotype

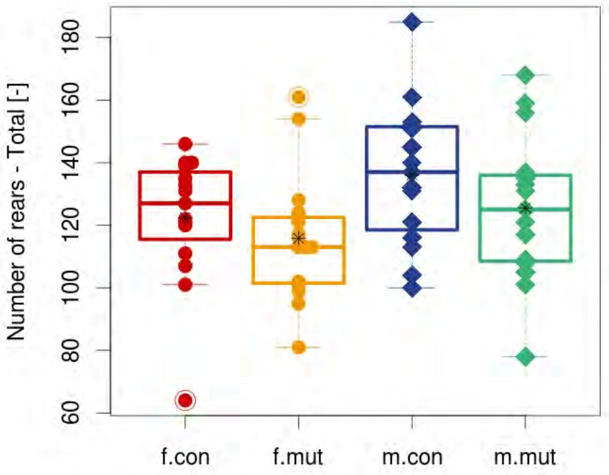

Figure 26
Open Field: Number of rears - Total boxplot with stripchart, split by sex and genotype

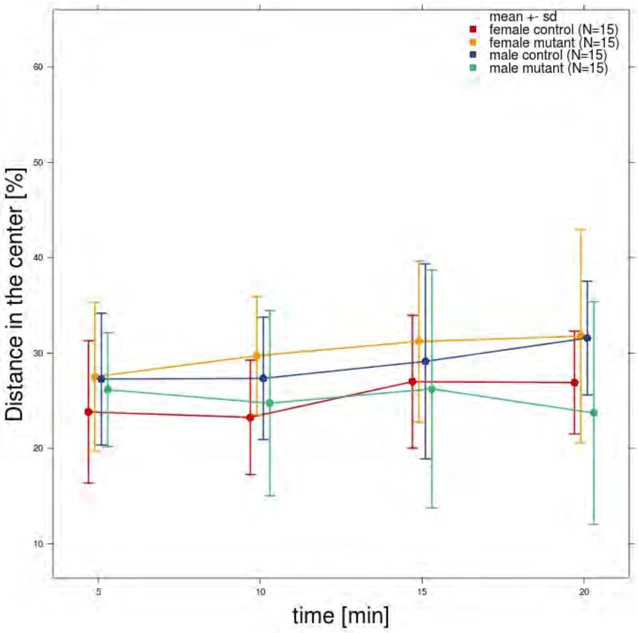

Figure 27
Open Field: Distance in the center, average plot, split by sex and genotype

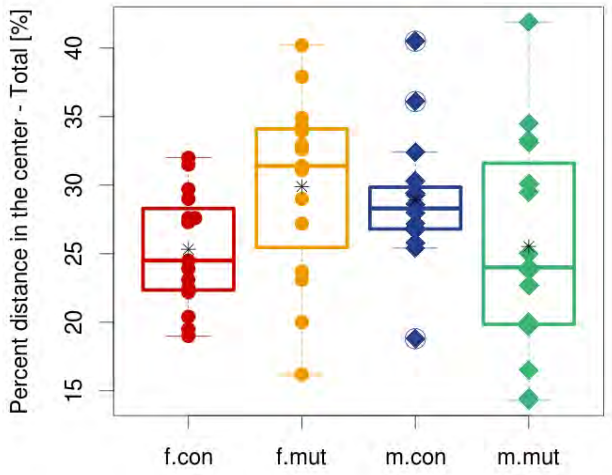

Figure 28
Open Field: Percent distance in the center - Total boxplot with stripchart, split by sex and genotype

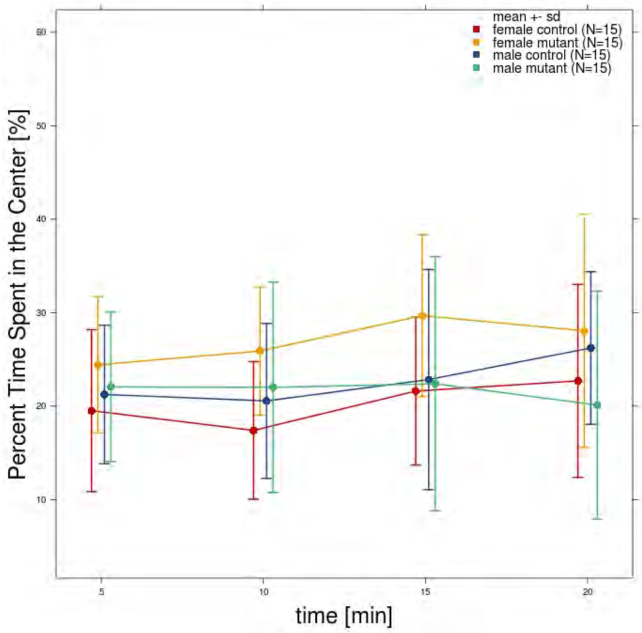

Figure 29
Open Field: Percent Time Spent in the Center, average plot, split by sex and genotype

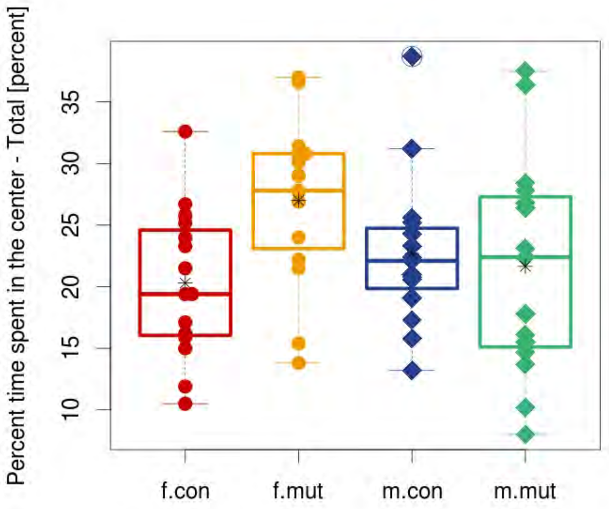

Figure 30
Open Field: Percent time spent in the center - Total boxplot with stripchart, split by sex and genotype

Table 17
Open Field: Means, standard deviation and p-values calculated by a linear model

	female		male		Linear model		
	control	mutant	control	mutant	genotype	sex	genotype:sex
	n=15	n=15	n=15	n=15			
	mean ± sd	mean ± sd	mean ± sd	mean ± sd	p-value	p-value	p-value
Distance traveled - 5 min	6876.43 ± 1282.29	6995.69 ± 1245.47	6497.43 ± 1075.2	5783.11 ± 1082.9	0.331	0.011	0.175
Distance traveled - 10 min	6302.4 ± 1243.13	5840.23 ± 1274.65	5515.83 ± 1095.82	4823.43 ± 1415.63	0.082	0.008	0.725
Distance traveled - 15 min	5514.11 ± 1194.81	5297.07 ± 1136.72	5222.43 ± 1340.59	4275.47 ± 1117.72	0.066	0.039	0.244
Distance traveled - 20 min	5177.79 ± 1098.03	4959.74 ± 1372.9	5077.68 ± 862.44	3998.18 ± 901.09	0.023	0.062	0.127
Distance traveled - Total	23870.73 ± 4042.5	23092.72 ± 4480.8	22313.35 ± 3538.22	18880.17 ± 3969.43	0.047	0.007	0.206
Number of rears - 5 min	25.2 ± 7.51	27.8 ± 6.32	31.73 ± 8	32.93 ± 8.09	0.332	0.004	0.72
Number of rears - 10 min	33.33 ± 7.65	29.73 ± 8.28	33.53 ± 6.51	31.93 ± 8.88	0.206	0.558	0.625
Number of rears - 15 min	30.73 ± 6.6	30.33 ± 6	34.67 ± 8.16	30.6 ± 8.17	0.241	0.269	0.334
Number of rears - 20 min	33.33 ± 9.12	28 ± 7.98	36.27 ± 6.79	30.33 ± 8.4	0.009	0.214	0.887
Number of rears - Total	122.47 ± 20.81	115.87 ± 20.88	136.07 ± 23	125.53 ± 24.08	0.141	0.048	0.733
Percent distance in the center - 5 min	23.83 ± 7.46	27.52 ± 7.82	27.28 ± 6.91	26.16 ± 5.96	0.484	0.569	0.193
Percent distance in the center - 10 min	23.26 ± 6	29.72 ± 6.21	27.35 ± 6.42	24.75 ± 9.71	0.307	0.814	0.019
Percent distance in the center - 15 min	27 ± 6.97	31.22 ± 8.44	29.13 ± 10.22	26.24 ± 12.47	0.792	0.573	0.163
Percent distance in the center - 20 min	26.91 ± 5.39	31.77 ± 11.18	31.57 ± 5.96	23.71 ± 11.68	0.522	0.469	0.008
Percent distance in the center - Total	25.32 ± 4.25	29.89 ± 6.7	28.91 ± 4.91	25.53 ± 8.06	0.711	0.809	0.015
Percent time spent in the center - 5 min	19.51 ± 8.66	24.42 ± 7.31	21.25 ± 7.41	22.06 ± 7.98	0.164	0.878	0.317
Percent time spent in the center - 10 min	17.4 ± 7.36	25.89 ± 6.85	20.56 ± 8.29	22.01 ± 11.25	0.029	0.872	0.119
Percent time spent in the center - 15 min	21.61 ± 7.91	29.67 ± 8.68	22.84 ± 11.78	22.39 ± 13.59	0.175	0.28	0.13
Percent time spent in the center - 20 min	22.7 ± 10.31	28.05 ± 12.49	26.22 ± 8.17	20.11 ± 12.21	0.893	0.438	0.047
Percent time spent in the center - Total	20.3 ± 6.06	27 ± 6.69	22.71 ± 6.19	21.65 ± 8.93	0.127	0.424	0.038
Whole Arena - resting time	109.55 ± 33.25	135.77 ± 23.34	115.81 ± 21.46	129.67 ± 41.79	0.015	0.992	0.444
Whole Arena - Permanence time	1200 ± 0	1200 ± 0	1200 ± 0	1200 ± 0	NA	NA	NA
Whole Arena - average speed	21.92 ± 3.73	21.71 ± 4.28	20.61 ± 3.44	17.63 ± 3.62	0.108	0.008	0.162
Periphery - distance	17834.38 ± 3264.7	16086.89 ± 2983.58	15838.71 ± 2655.01	13956.1 ± 2673.06	0.019	0.008	0.929
Periphery - resting time	99.68 ± 28.6	105.5 ± 19.48	99.22 ± 18.5	108.29 ± 33.47	0.268	0.862	0.808
Periphery - Permanence time	956.37 ± 72.59	875.93 ± 80.13	927.45 ± 74.27	940.25 ± 107.18	0.128	0.422	0.037
Periphery - average speed	20.87 ± 3.67	21.01 ± 4.03	19.22 ± 3.38	16.98 ± 3.63	0.275	0.004	0.216
center - distance	6036.35 ± 1426.01	7005.83 ± 2383.31	6474.65 ± 1523.19	4924.06 ± 2248.27	0.565	0.107	0.015
center - resting time	9.86 ± 6.72	30.28 ± 14.69	16.6 ± 7.97	21.36 ± 15.58	< 0.001	0.724	0.014
center - Permanence time	243.64 ± 72.59	324.07 ± 80.13	272.55 ± 74.26	259.75 ± 107.18	0.128	0.422	0.037
center - average speed	27.04 ± 5.61	23.73 ± 5.18	25.84 ± 4.52	20.98 ± 4.56	0.002	0.131	0.549
Latency to enter in the center	1.69 ± 1.85	2.07 ± 1.49	1.52 ± 0.9	1.45 ± 1.43	0.685	0.3	0.549
Number of entries in the center	291.4 ± 58.57	331.07 ± 95.95	299.33 ± 66.95	238.6 ± 91.8	0.612	0.045	0.018
WholeArenaDis	23870.73 ± 4042.5	23092.72 ± 4480.8	22313.35 ± 3538.22	18880.17 ± 3969.43	0.047	0.007	0.206

Prepulse Inhibition

There was a tendency (p=0.065) to decreased acoustic startle reactivity at the 110 dB intensity. There were no major differences in prepulse inhibition responding except for a decrease in the female mutant mice at the 73 (p=0.42) dB intensity and a tendency for a decrease in the female mutant mice at the 81 (p=0.09) dB intensity.

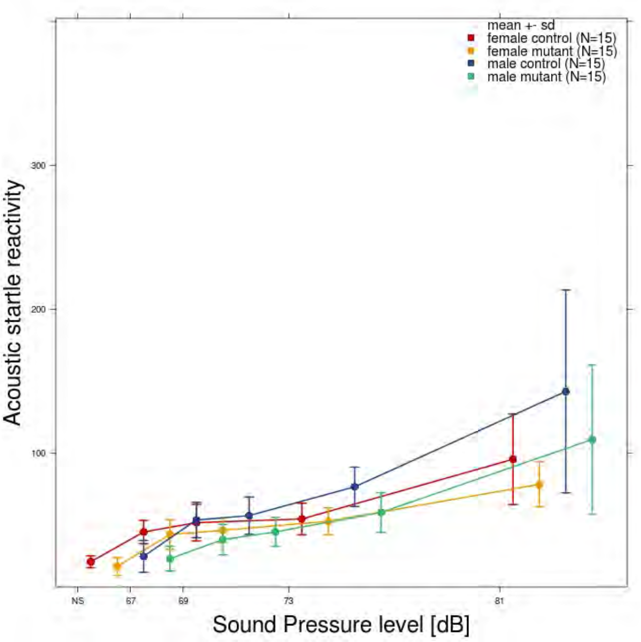

Figure 31
Acoustic Startle: Acoustic startle reactivity, average plot, split by sex and genotype

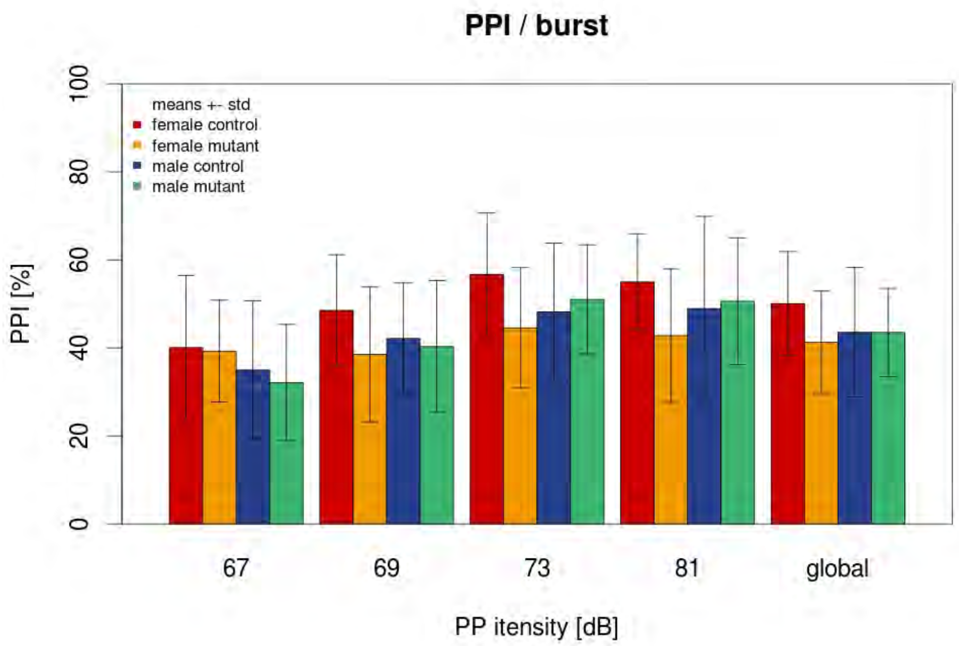

Figure 32
Prepulse Inhibition: PPI grouped barplot, split by sex and genotype

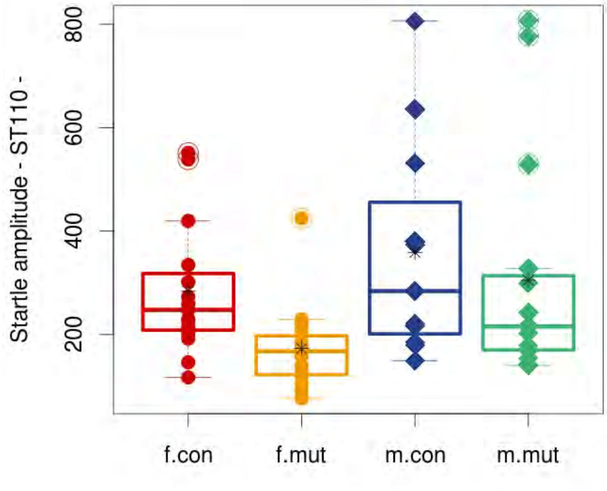

Figure 33
Prepulse Inhibition: Startle amplitude - ST110 boxplot with stripchart, split by sex and genotype

4.2.3 Discussion

In the open field, our test of spontaneous reactions to a novel environment, the mutant mice showed decreased total distance travelled. This indicates that these mice were hypoactive. Rearing activity was also decreased in these mice but this difference reached significance during the last 5 minutes of the test. This hypoactive and hypoexploratory behaviour would be consistent with the decreased rearing activity measured in the homecage environment during the indirect calorimetry test.

The female mutant mice were also spending more time and resting more in the central aversive zone, moving a greater distance within this zone and making more centre entries. Such a pattern of activity suggests that these female mice were exhibiting less anxiety-related behaviour. According to the Allen Brain Atlas, claudin 12 is not densely expressed throughout the brain. Nevertheless, it was expected that these mice may exhibit a phenotype in the blood brain barrier function. To determine whether this behaviour is indicative of a true effect of the mutation on anxiety, this finding would need to be replicated in an additional cohort of animals and further anxiety-related tests, such as the elevated plus maze, need to be performed.

The mutant mice also showed a tendency to decreased acoustic startle reactivity. A decrease in acoustic startle could indicate that these mice show impaired hearing ability, impaired neuromuscular recruitment or decreased anxiety-related behaviour. Given the findings in the female mice in the open field, it is possible that this acoustic startle effect reflects a change in anxiety responding. However, in the ABR test of the Neurology screen there was evidence of impaired hearing ability. Thus, these two findings could be complementary. Further tests of hearing ability should be carried out in these mice to determine whether this ASR finding relates specifically to auditory responding.

The female mutant mice also showed a pattern of decreased prepulse inhibition responding. In theory, alterations in PPI indicates a change in sensorimotor integration, hence alterations in brain function, which could be due to transformations in several neurochemical systems (see Geyer et al., 2001 for review). PPI is largely regulated by neuronal connections between the limbic cortex (including the entorhinal cortex, hippocampus and amygdala), ventral striatum, ventral pallidum as well as the pontine tegmentum. Glutamatergic fibers from the limbic cortex converge at the nucleus accumbens within the PPI regulatory circuitry. GABAergic projections, originating in the nucleus accumbens, then project to the globus pallidus. Regulation of PPI is carried out by GABAergic projections from the globus pallidus to the pedunculopontine nucleus. Increased PPI is associated with a reduction of GABAergic projections from the globus pallidus while decreases produce the reverse effect (Geyer and Dulawa, 2003; Swerdlow et al., 2001 for review). Based on the expression of Claudin 12 in the brain, it is not possible to say at this stage what may cause the altered prepulse inhibition in these mice. Nevertheless, this effect is small and would need to replicated in an additional batch of animals to determine whether this is a robust finding and if it should be pursued further.

4.2.4 Additional References

Geyer MA, Krebs-Thomson K, Braff DL, Swerdlow NR (2001): Pharmacological studies of prepulse inhibition models of sensorimotor gating deficits in schizophrenia: a decade in review. Psychopharmacol 156: 117-154.

Geyer MA, Dulawa SC (2003) Assessment of murine startle reactivity, prepulse inhibition and habituation. Curr. Protoc. Neurosci. 8: 8.17.

Swerdlow NR, Braff DL, Taaid N, Geyer MA (1994) Assessing the validity of an animal model of deficient sensorimotor gating in schizophrenic patients. Arch Gen Psych 51: 139-154.

Neurology 4.3

Summary 4.3.1

Mutants showed more tail elevation and a trend towards reduced hearing sensitivity at 24 kHz. Three female mutants showed tremor. Grip strength, rotarod performance as well as plasma lactate were without any differences.

Results 4.3.2

Modified SHIRPA

In the general observation series according a modified SHIRPA protocol we did observed tremor in three female mutants and significantly more tail elevation in both sexes. Overall appearance, movement and reflexes were without any significant findings.

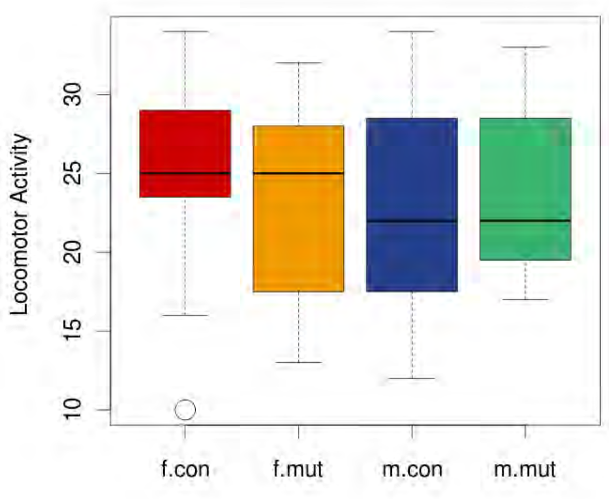

Figure 34
Modified SHIRPA: Locomotor Activity boxplot, split by sex and genotype

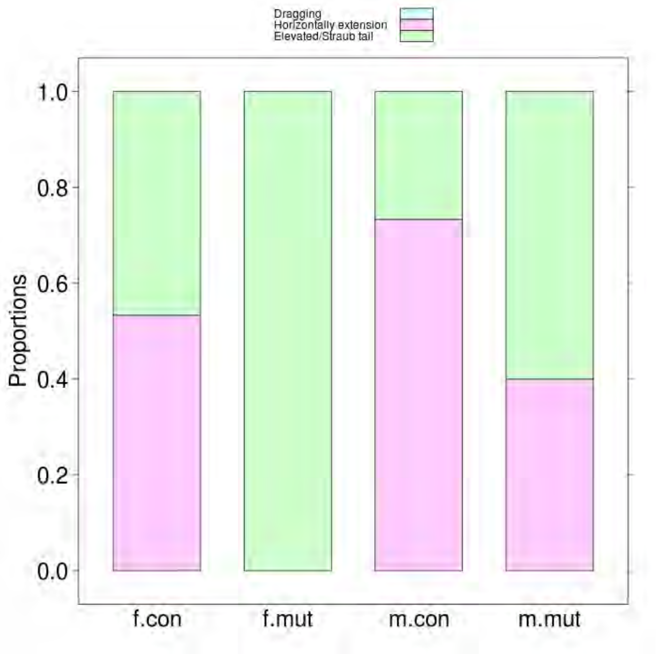

Figure 35
Modified SHIRPA: Tail Elevation proportion bar plot, split by sex and genotype

Table 18
Modified SHIRPA: Locomotor activity and body mass (SHIRPA): Means, standard deviation and p-values calculated by a linear model

	female		male		Linear model		
	control	mutant	control	mutant	genotype	sex	genotype:sex
	n=15	n=15	n=15	n=15			
	mean ± sd	mean ± sd	mean ± sd	mean ± sd	p-value	p-value	p-value
**Weight**	19.63 ± 0.95	17.95 ± 1.31	24.86 ± 2.51	23.13 ± 2.76	0.002	< 0.001	0.96
**Locomotor Activity**	24.6 ± 6.32	23.47 ± 6.3	22.67 ± 6.89	24.2 ± 5.54	0.902	0.713	0.415

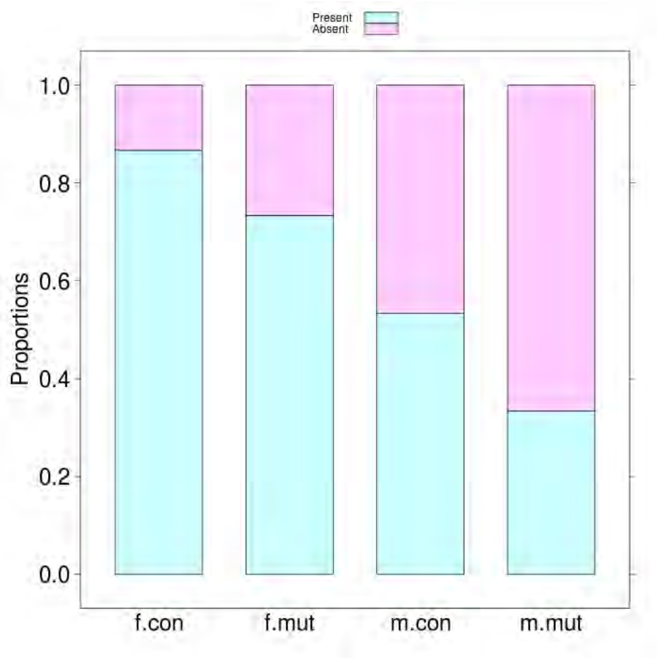

Figure 36
Modified SHIRPA: Urination proportion bar plot, split by sex and genotype

Table 19
Modified SHIRPA: Results ([1] Fisher's Exact test)

	Female		Male		Both		
	Control	Mutant	Control	Mutant	Control	Mutant	p value [1]
	n=15	n=15	n=15	n=15	50%	50%	
	Absolute		Absolute		Percent		
**Body Position**							NA
Inactive	0	0	0	0	0%	0%	
Active	15	15	15	15	100%	100%	
ExcessiveActivity	0	0	0	0	0%	0%	
**Tremor**							0.23729
Absent	15	12	15	15	100%	90%	
Present	0	3	0	0	0%	10%	
**Palpebral Closure**							NA
Eyes open	15	15	15	15	100%	100%	
Eyes closed	0	0	0	0	0%	0%	
**Lacrimation**							NA
Absent	15	15	15	15	100%	100%	
Present	0	0	0	0	0%	0%	
**Defecation**							0.33341
Present	13	14	9	12	73.3%	86.7%	
Absent	2	1	6	3	26.7%	13.3%	
**Transfer Arousal**							0.79477
Extended freeze	0	0	0	0	0%	0%	
Brief freeze	5	5	7	9	40%	46.7%	
Immediate movement	10	10	8	6	60%	53.3%	
**Gait**							NA
Fluid movement	15	15	15	15	100%	100%	
Lack Fluidity	0	0	0	0	0%	0%	
**Pelvic Elevation**							NA
less 5	0	0	0	0	0%	0%	
more 5	15	15	15	15	100%	100%	
**Tail Elevation**							0.00143
Dragging	0	0	0	0	0%	0%	
Horizontally extension	8	0	11	6	63.3%	20%	
Elevated/Straub tail	7	15	4	9	36.7%	80%	
**Startle Response**							NA
None	0	0	0	0	0%	0%	
Normal	15	15	15	15	100%	100%	
Jumping	0	0	0	0	0%	0%	

Table 20
Modified SHIRPA: Results ([1] Fisher's Exact test)

	Female		Male		Both		
	Control	Mutant	Control	Mutant	Control	Mutant	p value [1]
	n=15	n=15	n=15	n=15	50%	50%	
	Absolute		Absolute		Percent		
**Touch Escape**							NA
No response	0	0	0	0	0%	0%	
Response to touch	15	15	15	15	100%	100%	
Flees prior to touch	0	0	0	0	0%	0%	
**Positional Passivity**							NA
Struggles when held by the tail	15	15	15	15	100%	100%	
Struggles when held by the neck	0	0	0	0	0%	0%	
Struggles when laid supine	0	0	0	0	0%	0%	
No struggle	0	0	0	0	0%	0%	
**Trunk Curl**							NA
Absent	15	15	15	15	100%	100%	
Present	0	0	0	0	0%	0%	
**Limb Grasping**							NA
Absent	15	15	15	15	100%	100%	
Present	0	0	0	0	0%	0%	
**Pinna Reflex**							NA
Present	15	15	15	15	100%	100%	
Absent	0	0	0	0	0%	0%	
**Corneal Reflex**							NA
Present	15	15	15	15	100%	100%	
Absent	0	0	0	0	0%	0%	
**Urination**							0.28818
Present	13	11	8	5	70%	53.3%	
Absent	2	4	7	10	30%	46.7%	
**Contact Righting Reflex**							1
Present	15	15	14	15	96.7%	100%	
Absent	0	0	1	0	3.3%	0%	
**Evidence Of Biting**							0.29898
None	12	13	11	14	76.7%	90%	
Biting	3	2	4	1	23.3%	10%	
**Vocalisation**							1
No	7	7	9	10	53.3%	56.7%	
Yes	8	8	6	5	46.7%	43.3%	

Grip Strength

No genotype effect was detected for grip strength.

Table 21
Grip Strength: Linear model for 2-paws grip strength (mean of 3 trials)

	Estimate	CI	p-value
(Intercept)	71.75	[37.48 , 106.03]	<0.001
genotype	0.07	[-3.26 , 3.4]	0.97
sex	1.81	[-3.4 , 7.02]	0.49
weight	0.89	[-0.71 , 2.48]	0.27

Table 22
Grip Strength: Group means and standard deviation

	female		male	
	control	mutant	control	mutant
	n=15	n=15	n=15	n=15
	mean ± sd	mean ± sd	mean ± sd	mean ± sd
**Force 2 paws (mean)**	91.01 ± 13.07	89.51 ± 8.37	92.01 ± 11.5[a]	90.58 ± 13.92[a]
**Force 4 paws (mean)**	191.35 ± 22.6	191.43 ± 10.57	193.96 ± 17.27[a]	181.84 ± 21.65[a]

Table 23
Grip Strength: Linear model for 4-paws grip strength (mean of 3 trials)

	Estimate	CI	p-value
(Intercept)	128.65	[76.18 , 181.12]	<0.001
genotype	0.72	[-4.38 , 5.81]	0.78
sex	9.16	[1.19 , 17.13]	0.03
weight	2.85	[0.41 , 5.29]	0.02

Rotarod

Assessment of motor coordination and balance revealed reduced latencies on the rod with mutants more or less worse over all trials. There was the usual improvement over time reflected by the significant measurement terms. We also recorded the reason for trial ending: either falling jumping or rotating passively without detecting genotype effects.

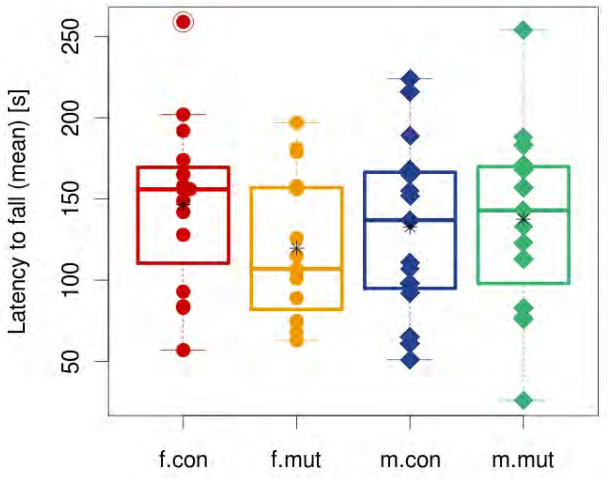

Figure 37
Rotarod: Latency to fall (mean) boxplot with stripchart, split by sex and genotype

Table 24
Rotarod: Latency to fall

	mean (all)	std (all)	mean (1.)	std (1.)	mean (2.)	std (2.)	mean (3.)	std (3.)
f.control	146.53	68.51	122	60.37	162.27	73.28	155.33	68.86
f.mutant	119.73	66.66	105	68.16	109.53	67.7	144.67	61.04
m.control	132.71	69.32	109.07	71.57	142.73	66.71	146.33	67.99
m.mutant	137.6	72.31	121.8	70.33	146.27	74.58	144.73	74.3

Table 25
Rotarod: Linear Mixed Effect Model

	Estimate	p-value
(Intercept)	141.571	0.061
sexm	9.372	0.734
genotypemutant	-12.296	0.388
measurement2	25.733	0.011
measurement3	33.3	0.001
bwrod	-1.133	0.76

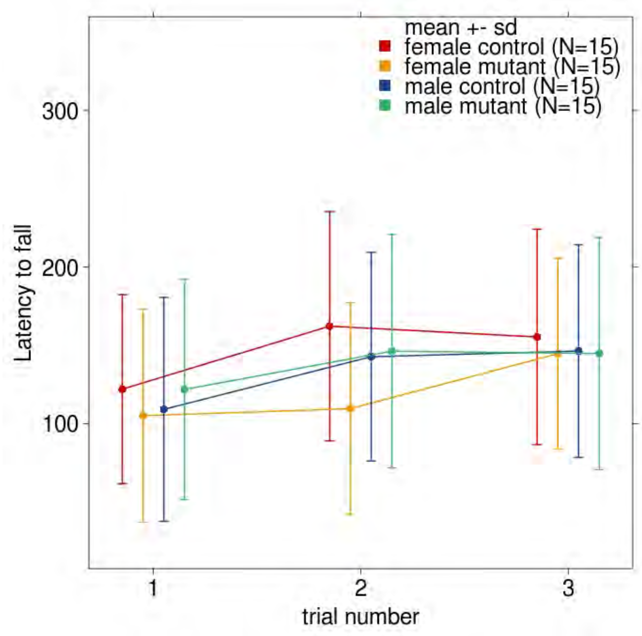

Figure 38
Rotarod: Latency to fall, average plot, split by sex and genotype

Table 26
Rotarod: Passive Rotation
[1] Fisher's Exact test (α =0.05)

	Male		Female		Both				
	**Control**	**Mutant**	**Control**	**Mutant**	**Control**	**Mutant**	**Control**	**Mutant**	**p value [1]**
	**n=45**	**n=45**	**n=45**	**n=45**	**n=90**	**n=90**	**50%**	**50%**	
	**Absolute**	**Absolute**	**Absolute**	**Absolute**	**Absolute**	**Absolute**	**Percent**	**Percent**	
**Passive rotation**									**1**
falling	27	26	27	27	54	53	60%	58.9%	
passive rotation	18	19	18	18	36	37	40%	41.1%	
jumping	0	0	0	0	0	0	0%	0%	

Lactate

Since we are especially interested in mitochondria functions we measure in collaboration with the Clinical-Chemistry Screen plasma lactate levels without detecting any differences in the mice analyzed here.

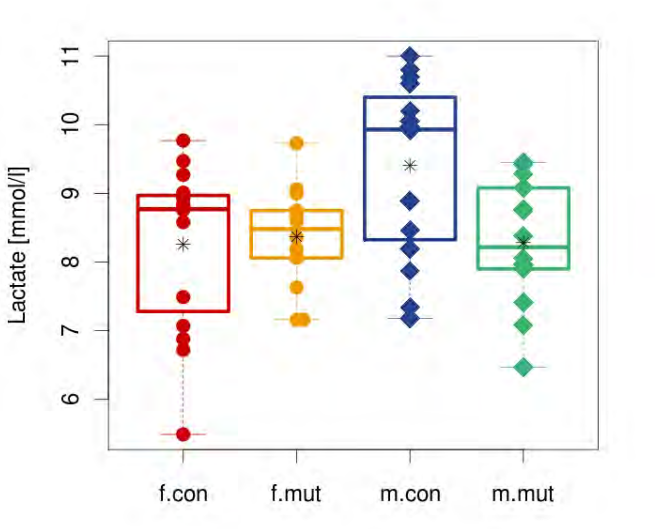

Figure 39
Lactate: Boxplot with stripchart, split by sex and genotype

Table 27
Lactate: Mean and standard deviation, split by sex and genotype

	female		male	
	control	mutant	control	mutant
	n=15	n=15	n=15	n=14
	mean ± sd	mean ± sd	mean ± sd	mean ± sd
**Lactate**[mmol/l]	8.26 ± 1.22	8.37 ± 0.72[a]	9.41 ± 1.3	8.28 ± 0.91

Table 28
Lactate: Linear Model with interactions Lactate

	Estimate	CI	p-value
(Intercept)	8.58	[8.3 , 8.86]	<0.001
genotype	0.25	[-0.03 , 0.54]	0.08
sex	-0.27	[-0.55 , 0.02]	0.06
genotype:sex	-0.31	[-0.59 , -0.03]	0.03

Auditory Brain Stem Response (ABR)

Hearing sensitivity was assessed by measuring the auditory brainstem response (ABR) of the mice to different auditory stimuli in a subset of mice. There were no clear difference in sound pressure thresholds needed to elicit a response. However, there was a trend at 24 kHz towards increased SPLs.

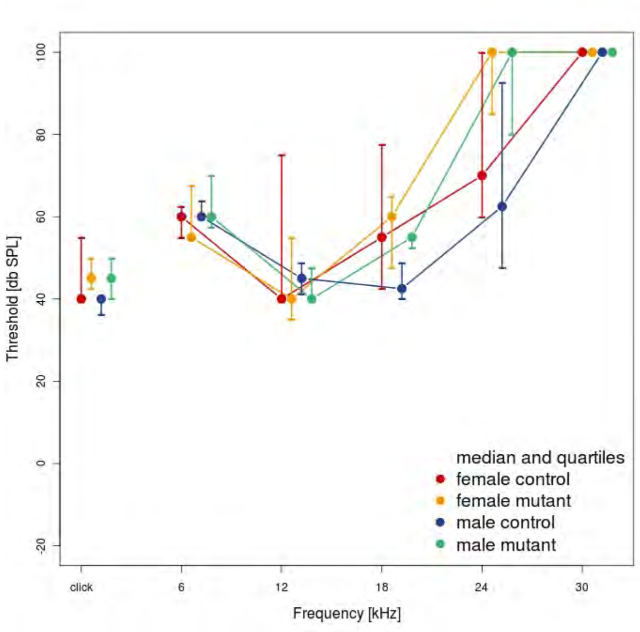

Figure 40
ABR: Stimulus intensity thresholds measured for different frequencies, medians and quartiles. Values above measurement limit (85db) are replaced by 100.

Table 29
ABR: Means, standard deviation and p-values calculated by a Wilcoxon rank-sum test. Values above measurement limit (85db) are replaced by 100.
[a] Number not based on the full number of animals (missing values)

	female		male		female	male	overall
	control	mutant	control	mutant			
	n=7	n=7	n=6	n=7			
	mean ± sd	mean ± sd	mean ± sd	mean ± sd	p-value	p-value	p-value
Click ABR [dB]	48 ± 15	46 ± 7	45 ± 17	46 ± 7	0.936	0.148	0.329
Threshold at 6 kHz [dB]	60 ± 6	61 ± 7	62 ± 7	63 ± 7	1	1	0.92
Threshold at 12 kHz [dB]	59 ± 29	45 ± 14	53 ± 23	46 ± 12	0.291	0.46	0.204
Threshold at 18 kHz [dB]	62 ± 27	61 ± 20	52 ± 24	59 ± 19	0.73	0.149	0.247
Threshold at 24 kHz [dB]	78 ± 21	89 ± 19	69 ± 26	89 ± 20	0.396	0.127	0.07
Threshold at 30 kHz [dB]	100 ± 0	98 ± 6	97 ± 8	100 ± 0	1	0.462	0.741
Body weight ABR [g]	22.8 ± 1	21 ± 0.9	29.6 ± 2.6	30.7 ± 1.5	0.012	0.509	0.624

Table 30
ABR: Linear Mixed Effect Model. With replacement for values above measurement limit.

	Estimate	p-value
Intercept	38.525	<0.001
genotype	2.957	0.426
frequency	1.799	<0.001
genotype:frequency	-0.21	0.206

Discussion 4.3.3

Increased tail elevation could be an indicator for more excitation/alertness or altered muscle tone. Since, the Behaviour screen detected reduced anxiety, the increased tail elevation is hypothesized to be linked to more audacity and alterness.

In addition, three mutant females presented with tremor, hinting towards an imbalance of excitation/inhibition. There was a trend towards decreased hearing sensitivity at 24 kHz and although this is quite small, it could also be linked to an imbalance of excitation/inhibition.

If the mouse line is analyzed further, we recommend to analyze the hearing sensitivity again.

4.4 Nociception

4.4.1 Summary

We could not detect any pain related phenotype in the mutant line.

4.4.2 Results

Hotplate

We did not find a significant difference in the first reaction latency between the genotypes.

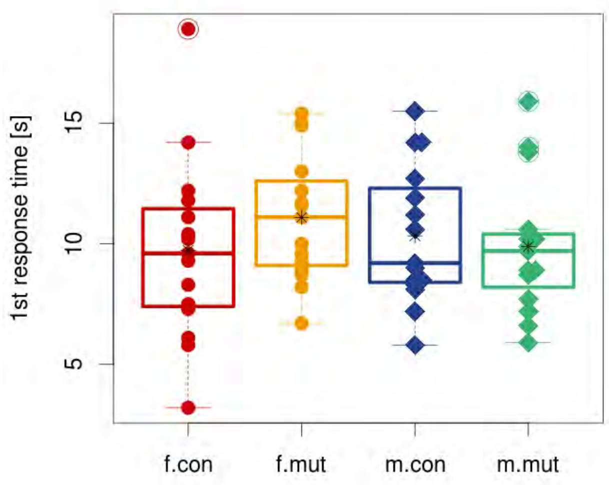

Figure 41
Hot Plate Test: 1st response time boxplot with stripchart, split by sex and genotype

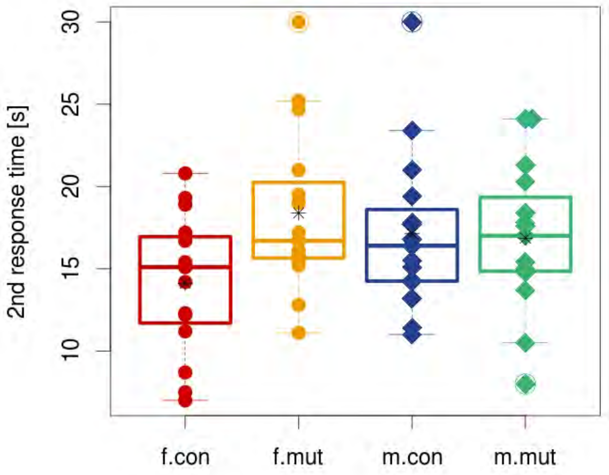

Figure 42
Hot Plate Test: 2nd response time boxplot with stripchart, split by sex and genotype

Table 31
Hot Plate Test: Observed reaction

	Female		Male	
	Control	Mutant	Control	Mutant
	n=15	n=15	n=15	n=15
**1st response type**				
hind paw licking	0	2	0	0
hind paw shaking	15	13	15	15
**2nd response type**				
hind paw licking	15	12	14	15
hind paw shaking	0	2	0	0
no reaction	0	1	1	0

Table 32
Hot Plate Test: First and second response time, group mean and standard deviation and ANOVA including post hoc calculation

	mean ± sd	mean ± sd	mean ± sd	mean ± sd	pairwise (Tukey)	pairwise (Tukey)	ANOVA	ANOVA	ANOVA
	female	female	male	male	females	males			
	control	mutant	control	mutant	mutant-control	mutant-control	genotype	sex	sex:genotype
	N=15	N=15	N=15	N=15	adj. p-value	adj. p-value	p-value	p-value	p-value
**Bodyweight**	20.68 ± 1.03	19.3 ± 1.2	27.59 ± 2.27	27.68 ± 2.17	0.15	0.999	0.162	< 0.001	0.11
**First response time**	9.73 ± 3.79	11.09 ± 2.65	10.33 ± 2.86	9.88 ± 2.82	0.62	0.978	0.566	0.703	0.258
**Second response time**	14.11 ± 4.26	18.39 ± 5.03	17.15 ± 4.9	16.86 ± 4.49	0.071	0.998	0.105	0.536	0.064

4.4.3 Discussion

The mutant animals did not show any significant difference in the first pain reaction compared to wild type mice.

Dysmorphology, Bone and Cartilage 4.5

Summary 4.5.1

In the DXA analysis, we detected a significantly decreased BMC and bone content in females. Concurrently body length was decreased in females.

Results 4.5.2

Morphological observeration

In the morphological investigation via visual inspection, no genotype-specific differences were found.

Dual-energy X-ray Absorptiometry (DXA)

In the DXA analysis, we detected a significantly decreased BMC and bone content in females. Concurrently body length was decreased in females.

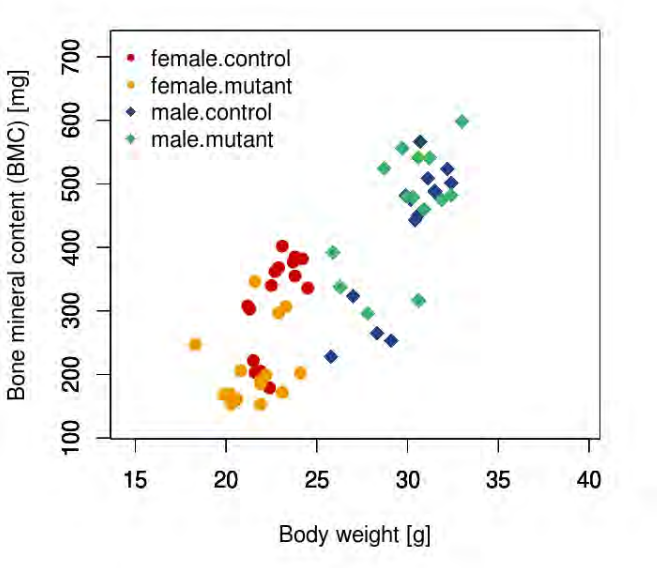

Figure 43
DXA analysis: Bone mineral content (BMC) vs. Body weight scatterplot

Table 33
DXA analysis: Group means, standard deviation and ANOVA (Tukey multiple comparisons of means)

	mean ± sd	mean ± sd	mean ± sd	mean ± sd	pairwise (Tukey)	pairwise (Tukey)	ANOVA	ANOVA	ANOVA
	female	female	male	male	females	males			
	control	mutant	control	mutant	mutant-control	mutant-control	genotype	sex	sex:genotype
	N=15	N=15	N=14	N=14	adj. p-value	adj. p-value	p-value	p-value	p-value
**BMD** [mg/cm^2]	47 ± 4	50 ± 5	49 ± 5	48 ± 4	0.472	0.816	0.665	0.819	0.107
**BMC** [mg]	315 ± 76	210 ± 61	428 ± 111	463 ± 94	0.009	0.722	0.106	< 0.001	0.004
**Bone content** [%]	1.38 ± 0.30	0.98 ± 0.28	1.41 ± 0.31	1.54 ± 0.27	0.002	0.646	0.059	< 0.001	0.001
**Body length** [cm]	9.25 ± 0.17	9.00 ± 0.26	9.64 ± 0.27	9.64 ± 0.17	0.02	1	0.035	< 0.001	0.041
**Body weight** [g]	22.74 ± 1.09	21.53 ± 1.53	30.05 ± 1.92	29.95 ± 2.12	0.221	0.999	0.137	< 0.001	0.22
**Fat Mass** [g]	0.98 ± 0.81	0.49 ± 0.77	3.14 ± 1.79	2.62 ± 1.69	0.746	0.73	0.155	< 0.001	0.965
**Lean Mass** [g]	17.63 ± 1.06	17.24 ± 1.48	21.85 ± 1.54	22.44 ± 2.23	0.91	0.77	0.849	< 0.001	0.253

X-Ray

In the morphological investigation via X-ray analysis, no genotype-specific differences were found.

4.5.3 Discussion

The Cldn12 mutant mouse line was analyzed in the Dysmorphology, Bone and Cartilage module of the German Mouse Clinic. In the DXA analysis, we detected a significantly decreased BMC and bone content in females. Concurrently body length was decreased in females. We expect that the changes in BMC are a secondary effect to the differences in body size and body weight. The sex differences which were observed here are common in many mouse strains, and thus are not abnormal (unpublished data).

Metabolic Screen 4.6

Summary 4.6.1

Mainly female mutants showed effects. Body mass was decreased but fat content was slightly increased. Food uptake was lower and RER shifted towards lipid oxidation. Rearing was decreased in both, male and female mutants.

Results 4.6.2

Indirect Calorimetry

During the indirect calorimetry trial no major effects of the mutation on energy turnover could be detected. Again body mass was slightly reduced in female mutants. Females also showed reduced food uptake and a consequent shift in RER towards lipid oxidation. Rearing behavior was reduced both in male and female mutants whereas no difference could be detected in distance travelled.

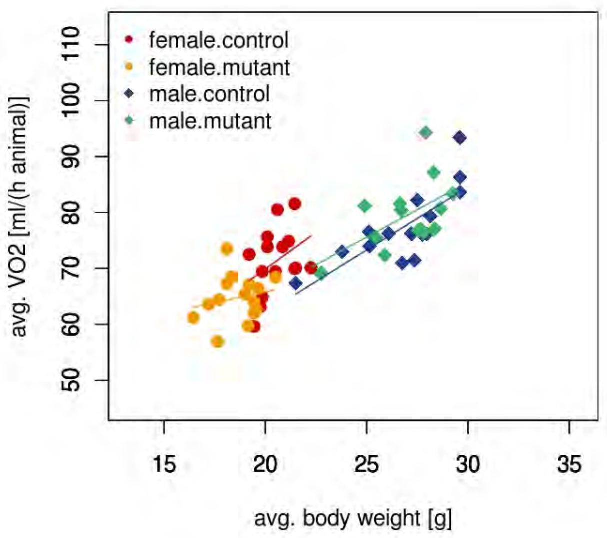

Figure 44
Indirect Calorimetry: Mean oxygen consumption [ml/(h animal)] versus body mass [g] scatterplot with regression lines

Table 34
Indirect Calorimetry: Means, standard deviation and p-values of a Linear Model

	female		male		Linear model			
	control	mutant	control	mutant	sex	genotype	body mass	sex:genotype
	n=15	n=15	n=15	n=15				
	mean ± sd	mean ± sd	mean ± sd	mean ± sd	p-value	p-value	p-value	p-value
**avg. body weight [g]**	20.5 ± 0.9	18.6 ± 1.1	26.9 ± 2.3	26.7 ± 2.1	< 0.001	0.019	NA	0.056
**Food intake [g]**	1.8 ± 0.7	0.9 ± 0.6	1.7 ± 0.8	1.5 ± 0.8	0.493	0.016	0.184	0.176
**avg. VO2 [ml/(h animal)]**	71.297 ± 5.928	64.731 ± 4.069	77.52 ± 6.641	78.87 ± 6.477	0.157	0.711	< 0.001	0.087
**min. VO2 [ml/(h animal)]**	44.867 ± 5.317	38.133 ± 8.043	49 ± 6.866	46.8 ± 7.939	0.408	0.108	0.013	0.55
**max. VO2 [ml/(h animal)]**	105.067 ± 13.818	95.2 ± 6.753	113.6 ± 10.048	117.533 ± 9.643	0.105	0.799	< 0.001	0.091
**avg. RER VCO2/VO2**	0.865 ± 0.046	0.811 ± 0.051	0.849 ± 0.043	0.837 ± 0.039	0.668	0.006	NA	0.081
**avg. distance [cm]**	2687 ± 832	2341 ± 721	8720 ± 22674	2664 ± 1559	0.284	0.28	NA	0.335
**avg. rearing [counts]**	116 ± 53	74 ± 26	103 ± 51	60 ± 20	0.195	< 0.001	NA	0.977

Determination of Body Composition by Time Domain Nuclear Magnetic Resonance (TD-NMR)

Both during the first and the second body composition analysis body mass trended to be decreased in mutant mice. Fat mass and lean mass were not significantly affected by genotype as a main effect but there was a sex X genotype interaction for body fat content when adjusted to body mass indicating that body fat content was slightly higher in female mutants. This effect could be detected at both measurements.

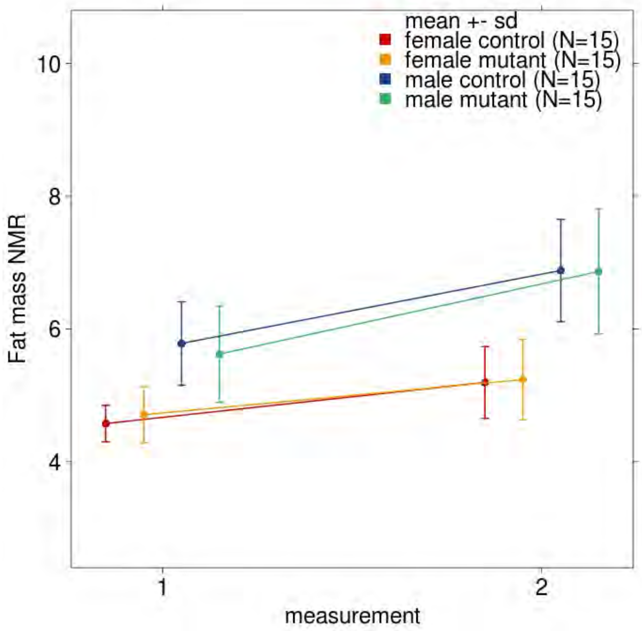

Figure 45
Body Composition Analysis: Fat mass NMR, average plot, split by sex and genotype

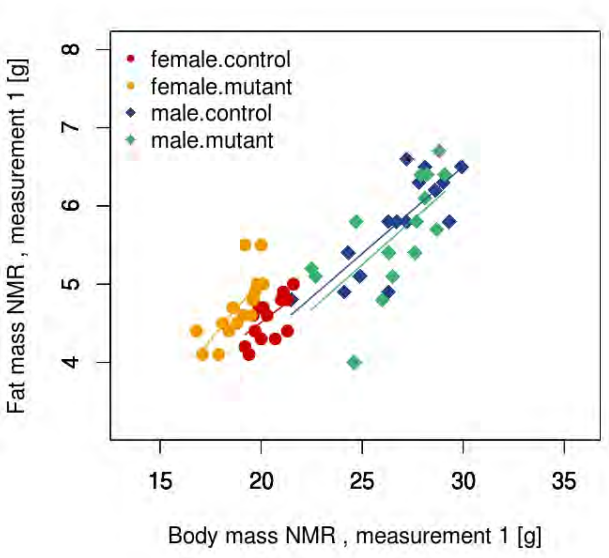

Figure 46
Body Composition Analysis: Fat mass vs. body mass scatterplot with regression lines

Table 35
Body Composition Analysis: p-values of Linear Mixed Models

	sex	genotype	measurement	Bodymass	genotype:measurement
Body mass NMR	< 0.001	0.145	< 0.001	NA	0.301
Fat mass NMR	< 0.001	0.069	0.358	< 0.001	0.507
Lean mass NMR	< 0.001	0.31	0.106	< 0.001	0.331

Table 36
Body Composition Analysis: Means, standard deviation and p-values of a Linear Model, first measurement

	female		male		Linear model			
	control	mutant	control	mutant	sex	genotype	body mass	sex:genotype
	n=15	n=15	n=15	n=15				
	mean ± sd	mean ± sd	mean ± sd	mean ± sd	p-value	p-value	p-value	p-value
Body mass NMR	20.3 ± 0.8	18.8 ± 1	26.7 ± 2.3	26.6 ± 2.1	< 0.001	0.073	NA	0.127
Fat mass NMR	4.6 ± 0.3	4.7 ± 0.4	5.8 ± 0.6	5.6 ± 0.7	0.015	0.094	< 0.001	0.004
Lean mass NMR	12.7 ± 0.6	11.5 ± 0.6	17.1 ± 1.4	17.2 ± 1.4	0.003	0.254	< 0.001	0.031

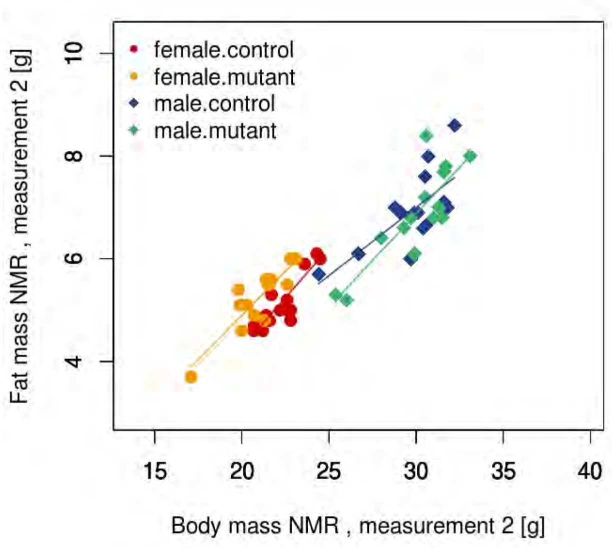

Figure 47
Body Composition Analysis: Fat mass vs. body mass scatterplot with regression lines

Table 37
Body Composition Analysis: Means, standard deviation and p-values of a Linear Model, second measurement

	female		male		Linear model			
	control	mutant	control	mutant	sex	genotype	body mass	sex:genotype
	n=15	n=14	n=15	n=14				
	mean ± sd	mean ± sd	mean ± sd	mean ± sd	p-value	p-value	p-value	p-value
**Body mass NMR**	22.4 ± 1.2	21 ± 1.5	29.6 ± 2.1	30 ± 2.2	< 0.001	0.313	NA	0.063
**Fat mass NMR**	5.2 ± 0.5	5.2 ± 0.6	6.9 ± 0.8	6.9 ± 0.9	0.001	0.135	< 0.001	0.007
**Lean mass NMR**	14 ± 0.6	13 ± 0.9	18.8 ± 1.4	19.2 ± 1.2	< 0.001	0.625	< 0.001	0.063

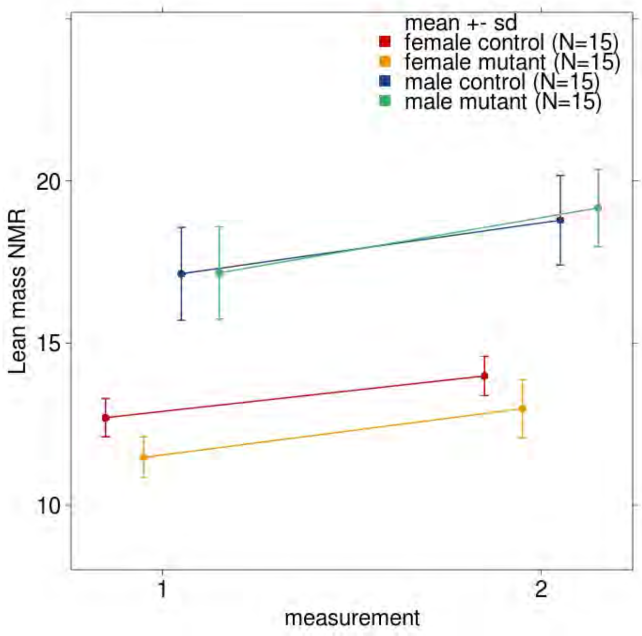

Figure 48
Body Composition Analysis: Lean mass NMR, average plot, split by sex and genotype

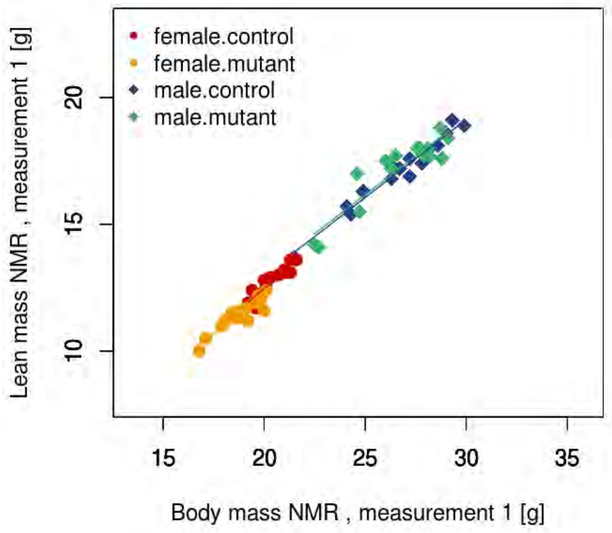

Figure 49
Body Composition Analysis: Fat mass vs. body mass scatterplot with regression lines

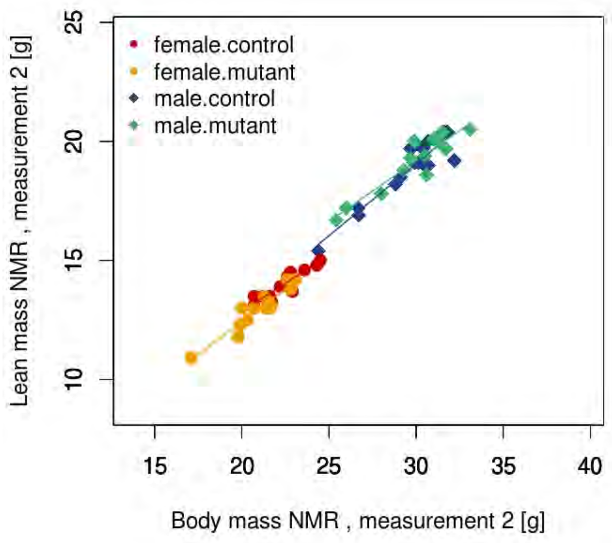

Figure 50
Body Composition Analysis: Fat mass vs. body mass scatterplot with regression lines

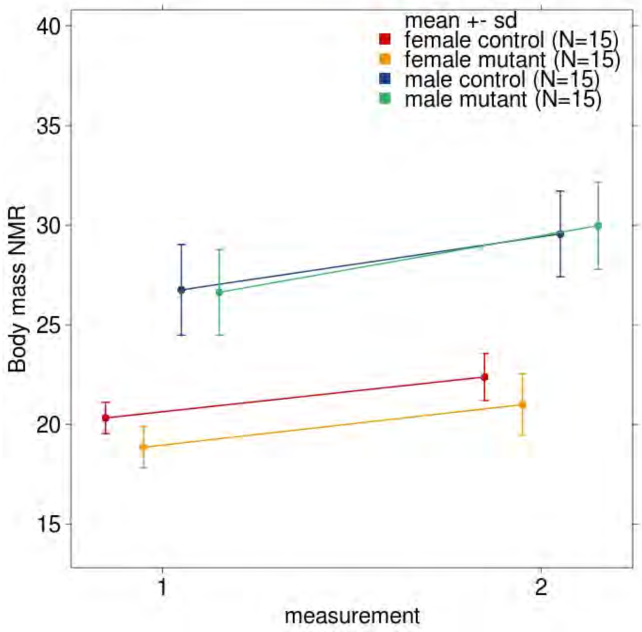

Figure 51
Body Composition Analysis: Body mass NMR, average plot, split by sex and genotype

4.6.3 Discussion

Only small effects on body mass, body composition, energy turnover and physical activity could be detected. We showed a consistent reduction in body mass in females but not males. Interestingly, body fat content was increased in female mutants. The differences were small and it is difficult to unravel the physiological mechanism resulting in increased fat accumulation. During the indirect calorimetry no hint was found why body composition was shifted. The decrease in food uptake and the consequent small shift towards lipid oxidation indicated by reduced RER are likely due to incompliance with the testing situation. However, this was related to the genotype. Reduced rearing behavior also suggest that rather behavioral traits than metabolic functions were affected.

4.7 Cardiovascular Screen

4.7.1 Summary

By echocardiography alterations were more often observed in female mutants. Mutants had smaller left ventricle (reduced innner diameter and LV Mass). The hearts performed in general better (increased FS and EF). However, the volume of blood pumped with each beat was reduced (reduced SV).

By electrocardiography, a mild decrease in atrio-ventricular conduction time (PQ and PR interval duration) was observed.

4.7.2 Results

Echocardiography

By echocardiography several alterations were found mainly in female mutants, namely:

Reduced LVID in systole and diastole, LV mass and Stroke Volume.

Increased Heart rate and respiration rate, and a better heart performance (increased EF and FS).

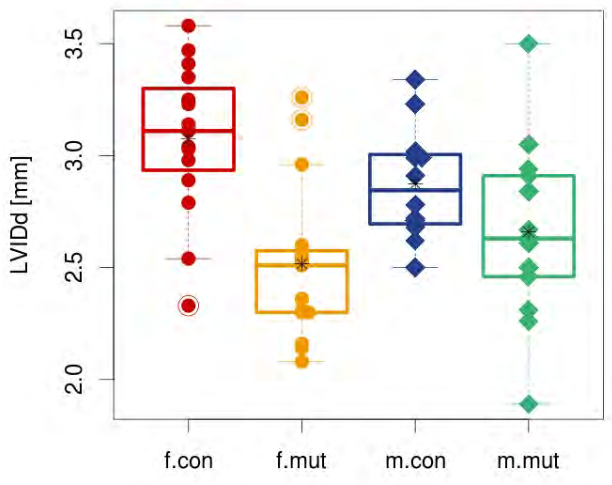

Figure 52
Echocardiography: LVIDd boxplot with stripchart, split by sex and genotype

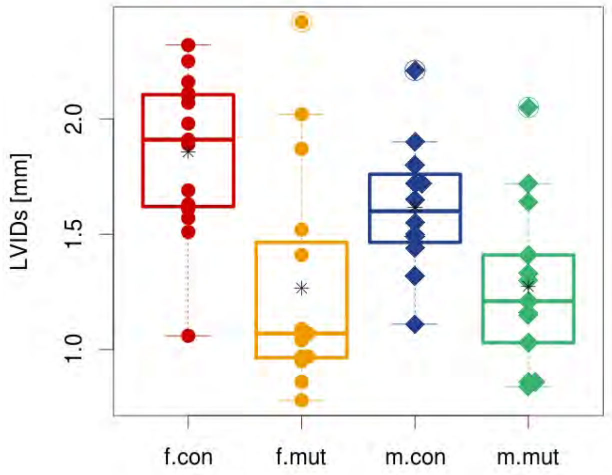

Figure 53
Echocardiography: LVIDs boxplot with stripchart, split by sex and genotype

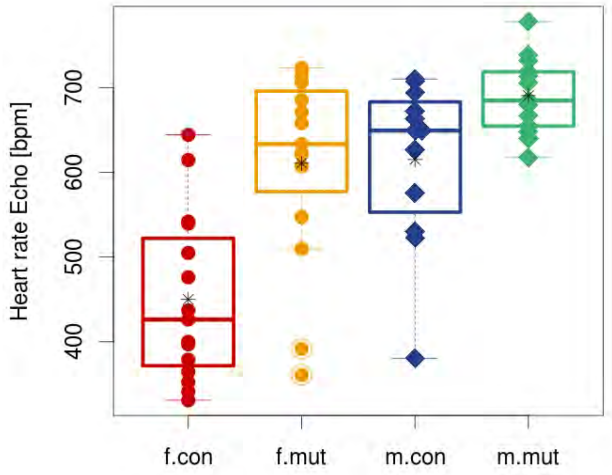

Figure 54
Echocardiography: Heart rate Echo boxplot with stripchart, split by sex and genotype

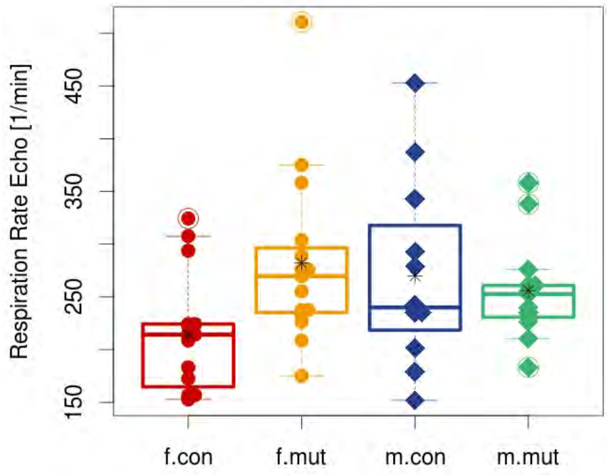

Figure 55
Echocardiography: Respiration Rate Echo boxplot with stripchart, split by sex and genotype

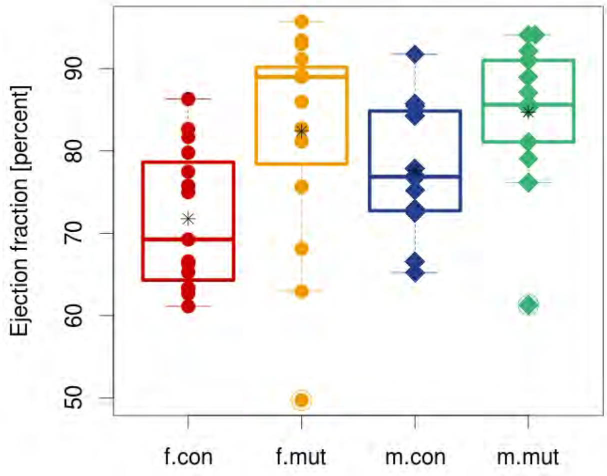

Figure 56
Echocardiography: Ejection fraction boxplot with stripchart, split by sex and genotype

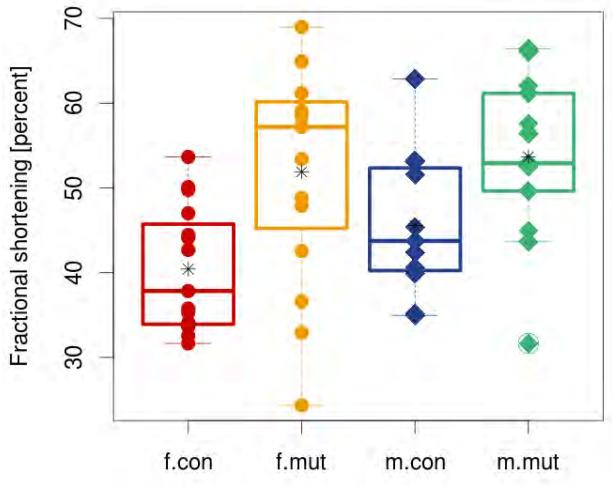

Figure 57
Echocardiography: Fractional shortening boxplot with stripchart, split by sex and genotype

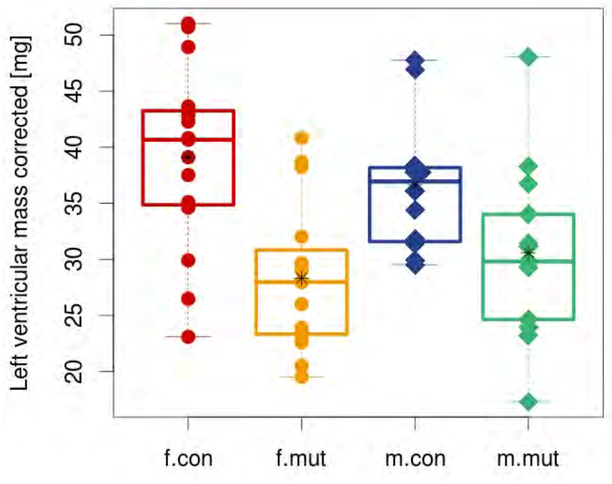

Figure 58
Echocardiography: Left ventricular mass corrected boxplot with stripchart, split by sex and genotype

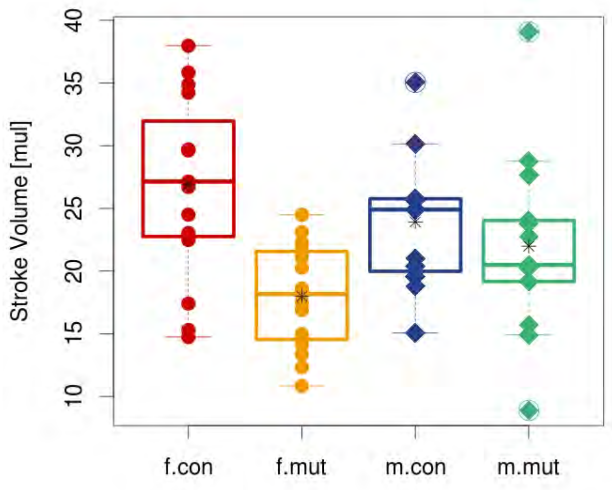

Figure 59
Echocardiography: Stroke Volume boxplot with stripchart, split by sex and genotype

Table 38
Echocardiography: Mean and standard deviation, split by sex and genotype

	female		male	
	control	mutant	control	mutant
	n=15	n=15	n=12	n=13
	mean ± sd	mean ± sd	mean ± sd	mean ± sd
**IVSs**[mm]	0.54 ± 0.03	0.53 ± 0.02	0.54 ± 0.02	0.51 ± 0.03
**IVSd**[mm]	0.51 ± 0.02	0.51 ± 0.04	0.54 ± 0.03	0.51 ± 0.03
**LVPWs**[mm]	0.58 ± 0.03	0.55 ± 0.04	0.56 ± 0.02	0.55 ± 0.05
**LVPWd**[mm]	0.57 ± 0.03	0.57 ± 0.02	0.57 ± 0.04	0.56 ± 0.03
**LVIDs**[mm]	1.86 ± 0.34	1.27 ± 0.48	1.62 ± 0.29	1.27 ± 0.36
**LVIDd**[mm]	3.08 ± 0.34	2.52 ± 0.36	2.87 ± 0.25	2.66 ± 0.41
**Weight**[g]	21.9 ± 1.2	20.3 ± 1.2	28.1 ± 2.1	28.5 ± 2.2
**Heart rate Echo**[bpm]	449.97 ± 99.61	610.67 ± 113.01	615.16 ± 97.33	690.61 ± 45.54
**Respiration Rate Echo**[1/min]	214.08 ± 56.09	282.14 ± 82.12	269.82 ± 87.46	256 ± 47.6
**Fractional shortening**[percent]	40.43 ± 7.36	51.9 ± 12.92	45.56 ± 8.23	53.66 ± 9.77
**Ejection fraction**[percent]	71.76 ± 8.44	82.39 ± 13.03	77.6 ± 7.99	84.72 ± 8.96
**Left ventricular mass corrected**[mg]	39.12 ± 8.29	28.32 ± 6.68	36.65 ± 5.95	30.59 ± 7.79
**Stroke Volume**[mul]	26.88 ± 7.35	18.01 ± 4.21	23.92 ± 5.38	21.99 ± 7.39
**Cardiac output**[ml/min]	11.857 ± 4.114	10.784 ± 2.688	14.447 ± 2.802	15.281 ± 5.704

Table 39

Echocardiography: Medians, first and third quartile and p-values calculated by a Wilcoxon rank-sum test

[a] Number not based on the full number of animals (missing values)

	female		male		female	male	overall
	control	mutant	control	mutant			
	n=15	n=15	n=12	n=13			
	median [25%, 75%]	median [25%, 75%]	median [25%, 75%]	median [25%, 75%]	p-value	p-value	p-value
IVSs [mm]	0.54 [0.52 , 0.56]	0.52 [0.51 , 0.55]	0.55 [0.52 , 0.55]	0.52 [0.5 , 0.54]	0.159	0.018	0.008
IVSd [mm]	0.51 [0.5 , 0.52]	0.51 [0.49 , 0.52]	0.54 [0.52 , 0.55]	0.51 [0.5 , 0.52]	0.861	0.056	0.181
LVPWs [mm]	0.58 [0.57 , 0.59]	0.57 [0.54 , 0.57]	0.56 [0.55 , 0.57]	0.54 [0.51 , 0.57]	0.052	0.296	0.058
LVPWd [mm]	0.57 [0.55 , 0.58]	0.57 [0.56 , 0.58]	0.56 [0.55 , 0.58]	0.58 [0.55 , 0.58]	0.689	0.859	0.628
LVIDs [mm]	1.91 [1.62 , 2.1]	1.07 [0.96 , 1.47]	1.6 [1.48 , 1.74]	1.21 [1.03 , 1.41]	0.001	0.013	< 0.001
LVIDd [mm]	3.11 [2.94 , 3.3]	2.51 [2.3 , 2.58]	2.85 [2.7 , 3]	2.63 [2.46 , 2.91]	0.001	0.095	< 0.001
Weight [g]	22.2 [21.1 , 22.6]	20.2 [19.7 , 21.2]	28.2 [27.6 , 29.6]	29.3 [28.2 , 29.8]	0.002	0.398	0.405
Heart rate Echo [bpm]	426.3 [371.68 , 522.2]	633.45 [577.28 , 695.89]	649.35 [564.3 , 677.71]	684.72 [654.55 , 718.65]	0.001	0.033	< 0.001
Respiration Rate Echo [1/min]	214.29 [164.76 , 224.3]	269.66 [235.31 , 296.48]	240.02 [226.89 , 305.23]	252.63 [230.77 , 260.87]	0.006	0.883	0.038
Fractional shortening [percent]	37.84 [33.91 , 45.73]	57.2 [45.23 , 60.15]	43.74 [40.43 , 51.98]	52.91 [49.63 , 61.17]	0.009	0.046	< 0.001
Ejection fraction [percent]	69.24 [64.31 , 78.63]	88.99 [78.4 , 90.17]	76.86 [72.86 , 84.55]	85.62 [81.09 , 91]	0.006	0.029	< 0.001
Left ventricular mass corrected [mg]	40.67 [34.87 , 43.25]	27.98 [23.34 , 30.84]	36.95 [31.7 , 38.09]	29.82 [24.65 , 34.02]	0.001	0.029	< 0.001
Stroke Volume [mul]	27.14 [22.76 , 31.96]	18.17 [14.55 , 21.57]	24.91 [20.19 , 25.75]	20.49 [19.17 , 24.04]	0.001	0.347	0.001
Cardiac output [ml/min]	12.627 [8.742 , 14.233]	9.857 [9.181 , 12.491]	14.681 [12.348 , 16.285]	14.624 [12.421 , 17.075]	0.486	0.979	0.457

Electrocardiography

By electrocardiography, a mild decrease in PQ and PR interval duration mainly in female mutants was observed.

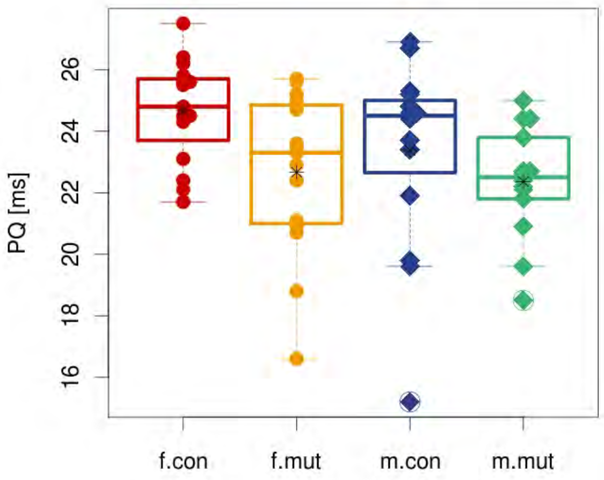

Figure 60
Electrocardiography: PQ boxplot with stripchart, split by sex and genotype

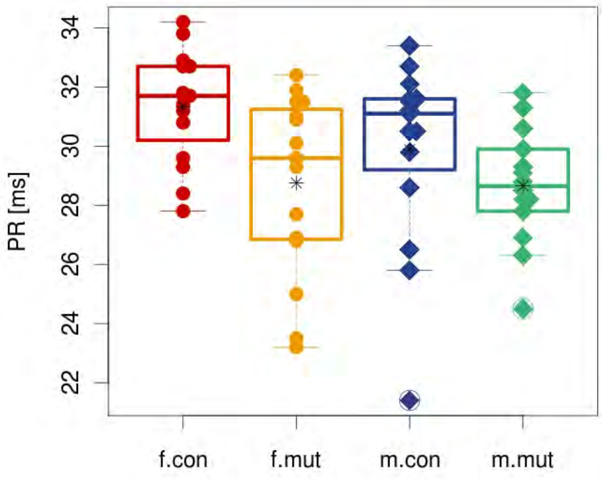

Figure 61
Electrocardiography: PR boxplot with stripchart, split by sex and genotype

Discussion 4.7.3

Alterations obtained by recording ECGs might reflect the findings by echocardiography. In smaller hearts the signal needs less time and thus the intervals are shorter. The biological relevance is unclear.

As the HR and the RR are increased mutants, might be more accessible to stress. The increased HR might cause reduction of inner diamter and thus increased FS and EF.

Caution in interpreting the data have to be taken as the female mutants are less heavy than female controls which might influences dimensions.

4.8 Eye Screen

4.8.1 Summary

We found significantly reduced eye sizes in Cldn12 mutants, and a decrease (p<0,01) of retinal thickening in the case of female mutants.

4.8.2 Results

Eye Size Measurement by Laser Interference Biometry

Laser interference biometry indicated significantly reduced axial eye lenghts (distance between cornea and retina; p<0.01) in the Cldn12 mutants.

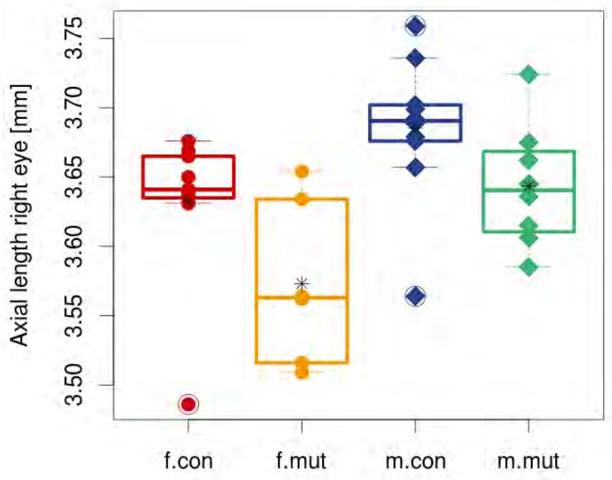

Figure 62
Eye Size: Axial length right eye boxplot with stripchart, split by sex and genotype
Axial eye length is significantly reduced in Cldn12 male mutants.

Optical Coherence Tomography (OCT)

Concerning the posterior part of the eye, OCT investigations demonstrated a normally developed fundus in the Cldn12 mutant eyes. Number of main blood vessels and retinal thicknesses were not significantly altered in the mutants.

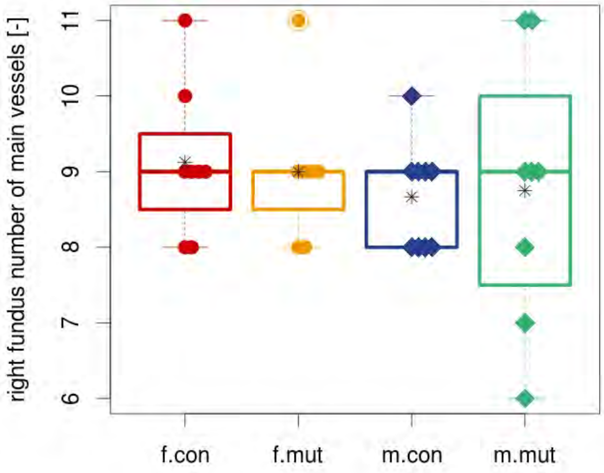

Figure 63
OCT: right fundus number of main vessels boxplot with stripchart, split by sex and genotype
Number of main blood vessels were not significantly altered in the ocular fundus of the Cldn12 mutants. The same was found for the left eyes.

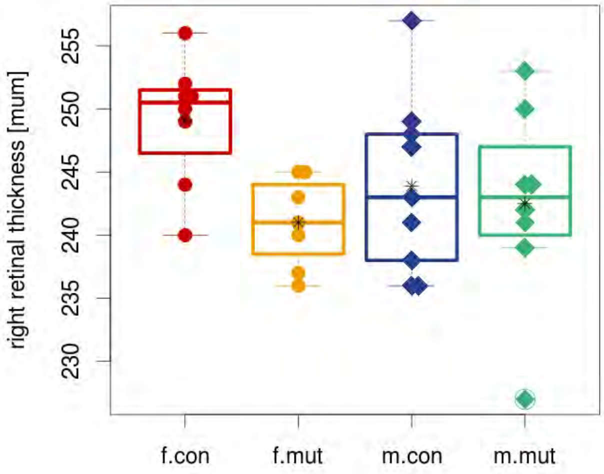

Figure 64
OCT: right retinal thickness boxplot with stripchart, split by sex and genotype
All retinal layers were present in the Cldn12 mutants. The same was found for the left eyes.

Anterior Eye Investigation by Scheimpflug Imaging

We did not find abnormalities of the anterior eye segment associated with the Cldn12 mutation. Scheimpflug imaging indicated normal developed lenses and corneas in all tested animals of both controls and mutants.

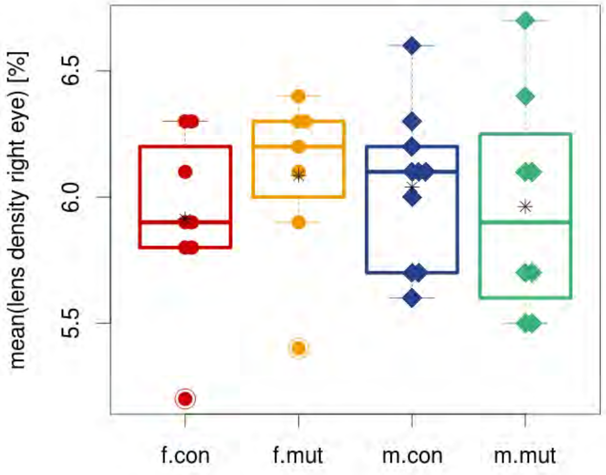

Figure 65

Anterior Eye Investigation: mean(lens density right eye) boxplot with stripchart, split by sex and genotype

Lenses of the Cldn12 mutants were well developed, no difference in transparency was recorded when compared to the control littermates. The same was found for the left eyes.

Virtual Vision Test

Due to uncooperative mice the test could not be conducted completely.

4.8.3 Discussion

The Cldn12 mutants have been investigated for the morphological changes in the anterior (cornea apearence and lens apearence/transparency) and posterior part of the eye (main blood vessels apearence and number, retina apearence and thickness of the retina, along with retinal layers structure). Laser interference biometry indicated significantly reduced axial eye lenghts (distance between cornea and retina; $p<0.01$) in the Cldn12 mutants.

Clinical Chemistry and Hematology 4.9

Summary 4.9.1

IpGTT: We found slightly decreased basal fasting glucose levels in mutants compared to controls, but no significant genotype effect on overnight weight loss due to food withdrawal and blood glucose response to the glucose challenge.

Clinical chemistry: No significant genotype related differences in fasting plasma lipid and glucose levels were found in Cldn12 mutant mice, but decreased plasma protein and triglyceride (trend) levels and increased creatinine values and ALP activity (very mild) in ad libitum fed mutants.

Hematology data indicated mild macrocytosis with increased MCH and decreased RBC, but increased WBC predominantly in mutant males.

In total we found mainly mild differences, that might indicate effects on energy metabolism, protein metabolism or hematopoiesis. However, since the differences are small, further investigations are recommended to confirm genotype-dependence of these findings and elucidate underlying cause.

Results 4.9.2

Intraperitoneal Glucose Tolerance Test (IpGTT)

In the glucose tolerance test (ipGTT) we did not see clearly genotype-related effects, besides a very mild decrease in basal fasting glucose levels in mutants compared to controls.

Table 40

IpGTT: Linear model including sex, genotype and body mass before overnight food withdrawal as well as sex:genotype interaction as factors influencing fasting-induced body mass loss.

	Estimate	CI	p-value
(Intercept)	0.25	[-0.82 , 1.32]	0.64
genotypemutant	-0.06	[-0.31 , 0.19]	0.65
sexm	-0.16	[-0.57 , 0.24]	0.42
bw_before	0.09	[0.04 , 0.14]	<0.001
genotypemutant:sexm	0.24	[-0.11 , 0.58]	0.18

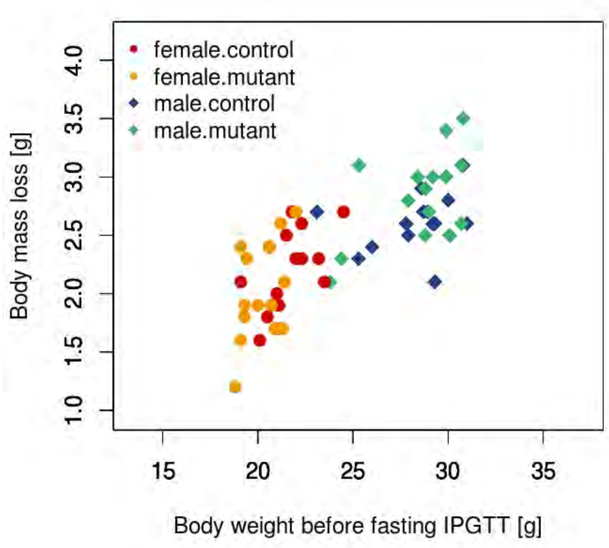

Figure 66
IpGTT: Body mass loss vs. body weight before overnight food withdrawal

Table 41
IpGTT: Basal glucose level and area under the curve (AUC) for the first 30 minutes and the remaining 90 minutes of the test obtained from glucose tolerance testing: Means, standard deviation and p-values calculated by a linear model.

	female		male		Linear model		
	control	mutant	control	mutant	genotype	sex	genotype:sex
	n=15	n=15	n=15	n=15			
	mean ± sd	mean ± sd	mean ± sd	mean ± sd	p-value	p-value	p-value
**Glucose (T=0)**	4.79 ± 0.64	4.42 ± 0.73[a]	5.34 ± 0.62	4.8 ± 0.64	0.01	0.009	0.632
**AUC 0-30**	284.65 ± 55.15	308.96 ± 30.82[a]	362.81 ± 50.21	340.13 ± 39.45	0.945	< 0.001	0.051
**AUC 30-120**	562.46 ± 246.09[a]	448.07 ± 123.64[a]	763.52 ± 193.27[a]	781.03 ± 160.56[a]	0.353	< 0.001	0.208

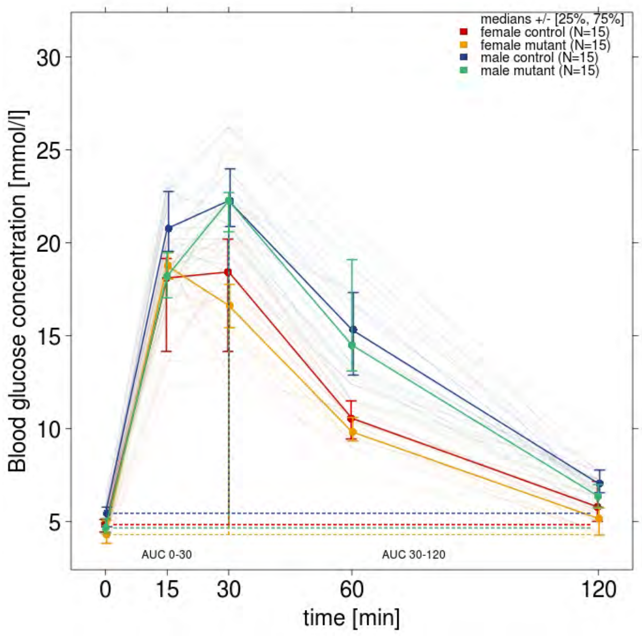

Figure 67
IpGTT: Blood glucose concentration average plot, split by sex and genotype

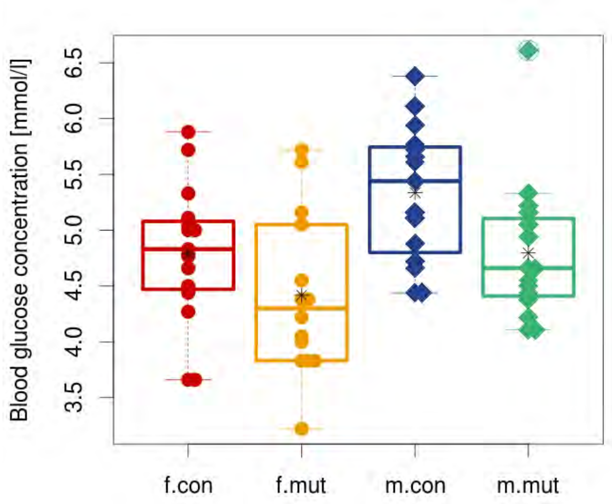

Figure 68
IpGTT: Glucose (T=0) boxplot with stripchart, split by sex and genotype

Clinical Chemistry (after overnight food withdrawal)

Fasting plasma lipid and glucose levels did not reveal clearly genotype-associated differences. A borderline significant genotype effect was found for calculated non-HDL cholesterol levels, with slightly higher values in mutants. However, neither total cholesterol nor measured HDL-cholesterol concentrations showed significant genotype-related differences, suggesting that the difference seen in non-HDL cholesterol might be accidental. Additionally we found significant genotype x sex interaction effects for fasting NEFA and glucose, with increased glucose levels and decreased NEFA and in trend glycerol concentrations in mutant females compared to female controls.

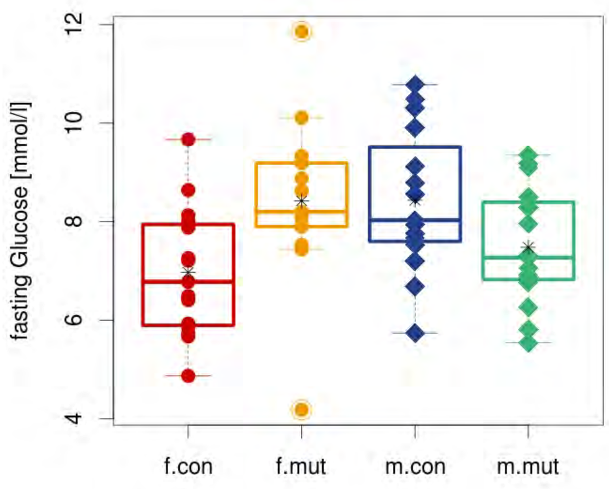

Figure 69
Clinical Chemistry (unfed mice): fasting Glucose boxplot with stripchart, split by sex and genotype

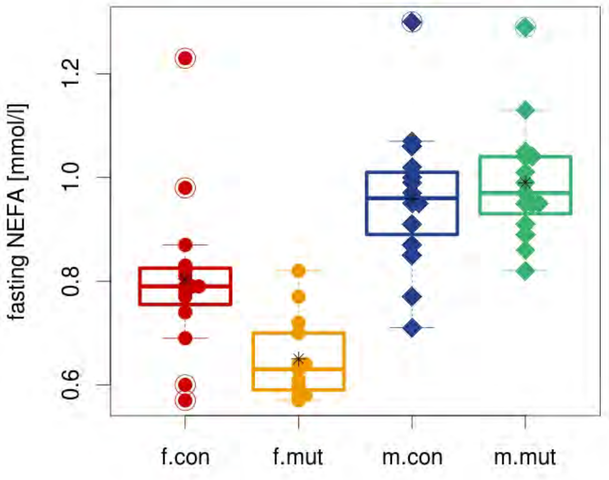

Figure 70
Clinical Chemistry (unfed mice): fasting NEFA boxplot with stripchart, split by sex and genotype

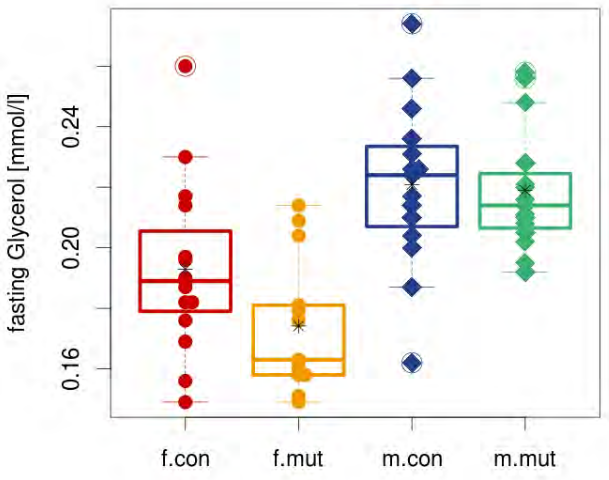

Figure 71
Clinical Chemistry (unfed mice): fasting Glycerol boxplot with stripchart, split by sex and genotype

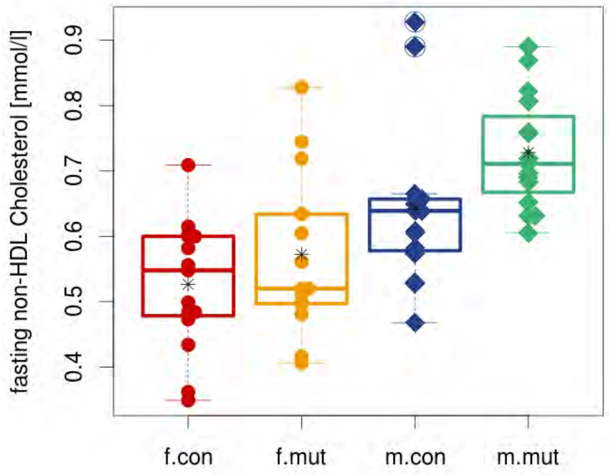

Figure 72
Clinical Chemistry (unfed mice): fasting non-HDL Cholesterol boxplot with stripchart, split by sex and genotype

Table 42
Clinical Chemistry (unfed mice): Fasting plasma lipid and glucose levels determined after overnight food-withdrawal. Means, standard deviations and p-values for genotype, sex and genotype × sex interaction effects calculated by a linear model.

	female		male		Linear model		
	control	mutant	control	mutant	genotype	sex	genotype:sex
	n=15	n=15	n=15	n=15			
	mean ± sd	mean ± sd	mean ± sd	mean ± sd	p-value	p-value	p-value
**fasting Glucose** [mmol/l]	6.97 ± 1.31	8.42 ± 1.75[a]	8.43 ± 1.47	7.48 ± 1.21	0.513	0.492	0.002
**fasting Cholesterol** [mmol/l]	2.311 ± 0.197	2.382 ± 0.185[a]	2.801 ± 0.151	2.851 ± 0.139	0.183	< 0.001	0.809
**fasting HDL-cholesterol** [mmol/l]	1.785 ± 0.122	1.81 ± 0.089[a]	2.16 ± 0.131	2.122 ± 0.09	0.826	< 0.001	0.285
**fasting non-HDL Cholesterol** [mmol/l]	0.527 ± 0.099	0.572 ± 0.128[a]	0.641 ± 0.123	0.728 ± 0.088	0.026	< 0.001	0.48
**fasting Triglycerides** [mmol/l]	0.96 ± 0.34	1.122 ± 0.379[a]	1.306 ± 0.412	1.34 ± 0.348	0.32	0.006	0.512
**fasting NEFA** [mmol/l]	0.8 ± 0.15	0.65 ± 0.08[a]	0.96 ± 0.14	0.99 ± 0.12	0.07	< 0.001	0.007
**fasting Glycerol** [mmol/l]	0.193 ± 0.029	0.174 ± 0.022[a]	0.221 ± 0.028	0.219 ± 0.021	0.122	< 0.001	0.206

Clinical Chemistry

Clinical chemistry analyses of plasma samples revealed genotype related differences in plasma protein levels, creatinin concentrations and alkaline phosphatase activity. Protein levels for both, total protein (TP) and albumin, were decreased in mutant mice compared to controls, with a more prominent effect in males. Additionally plasma triglyceride levels very slightly decreased. Creatinine concentrations in contrast were slightly higher in mutant mice than in controls, with a stronger effect in females. Furthermore we found some borderline significant effects, like genotype x sex interaction effects for sodium, potassium and cholesterol values, a trend towards higher plasma ASAT activities in mutants, but rather lower ALAT activity values in mutants compared to controls, that might represent accidental findings.

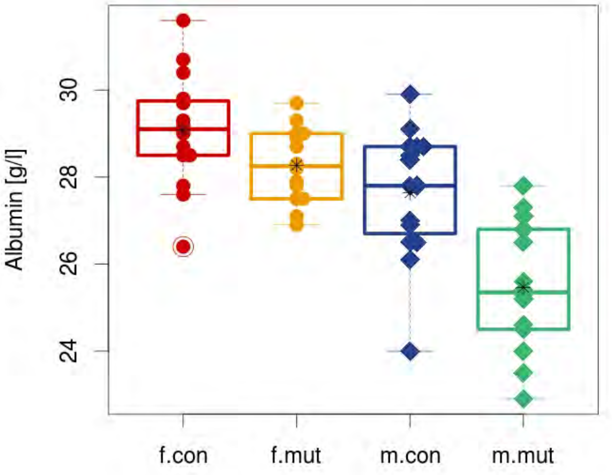

Figure 73
Clinical Chemistry: Albumin boxplot with stripchart, split by sex and genotype

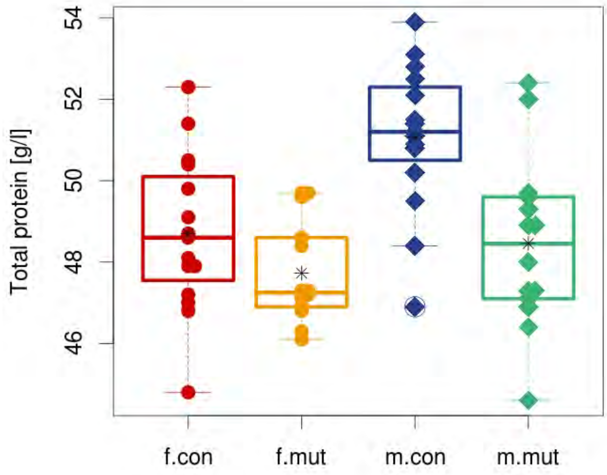

Figure 74
Clinical Chemistry: Total protein boxplot with stripchart, split by sex and genotype

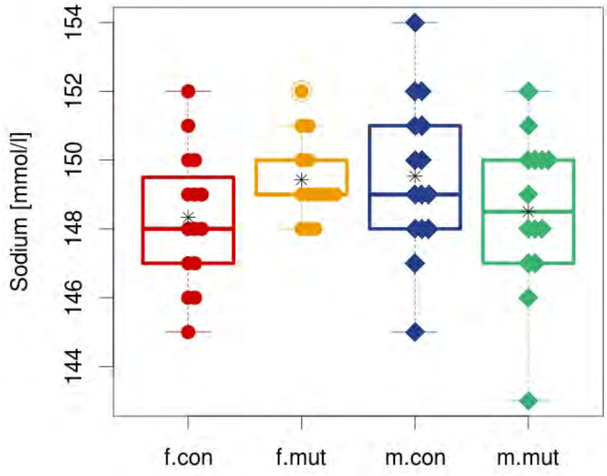

Figure 75
Clinical Chemistry: Sodium boxplot with stripchart, split by sex and genotype

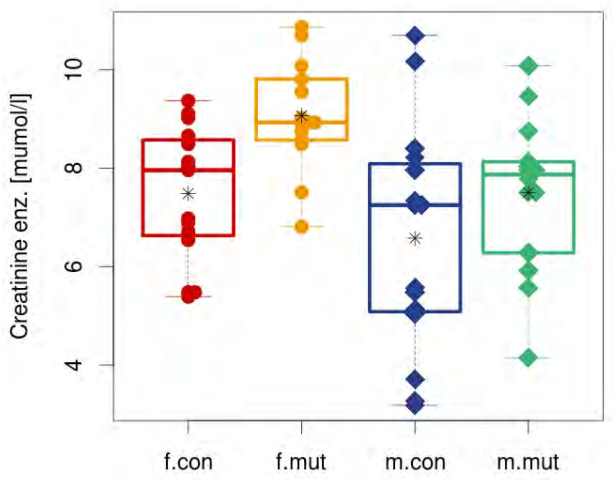

Figure 76
Clinical Chemistry: Creatinine enz. boxplot with stripchart, split by sex and genotype

Table 43
Clinical Chemistry: Plasma electrolyte, protein, creatinine and urea concentrations of ad libitum fed mice. Means, standard deviations and p-values for genotype, sex and genotype × sex interaction effects calculated by a linear model.

	female		male		Linear model		
	control	mutant	control	mutant	genotype	sex	genotype:sex
	n=15	n=15	n=15	n=14			
	mean ± sd	mean ± sd	mean ± sd	mean ± sd	p-value	p-value	p-value
**Sodium** [mmol/l]	148 ± 2	149 ± 1[a]	150 ± 2	148 ± 2	0.953	0.796	0.047
**Potassium** [mmol/l]	4 ± 0.4	3.8 ± 0.2[a]	4.2 ± 0.2	4.3 ± 0.2	0.72	< 0.001	0.058
**Chloride** [mmol/l]	111.9 ± 1.8	112.1 ± 1.2[a]	109.9 ± 1.5	109.5 ± 1.7	0.73	< 0.001	0.451
**Total protein** [g/l]	48.7 ± 2	47.7 ± 1.2[a]	51.1 ± 1.8	48.5 ± 2.1	< 0.001	0.002	0.088
**Albumin** [g/l]	29.1 ± 1.3	28.3 ± 0.9[a]	27.6 ± 1.5	25.5 ± 1.5	< 0.001	< 0.001	0.054
**Creatinine enz.** [mumol/l]	7.48 ± 1.37	9.07 ± 1.11[a]	6.58 ± 2.34	7.51 ± 1.57	0.006	0.007	0.46
**Urea** [mmol/l]	11.7 ± 1.13	11.49 ± 1.96[a]	12.92 ± 1.13	12.08 ± 1.61	0.183	0.025	0.424

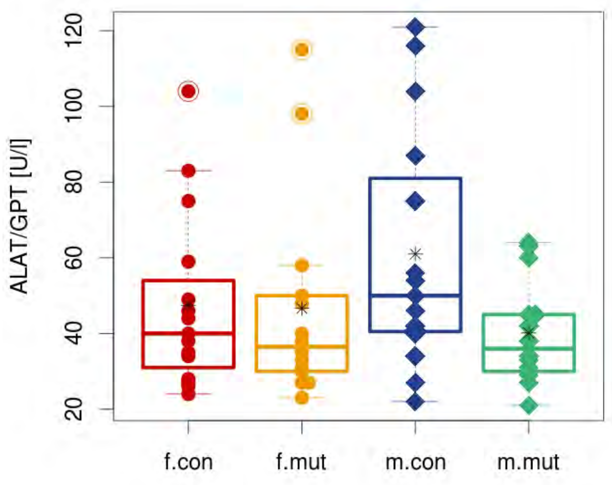

Figure 77
Clinical Chemistry: ALAT/GPT boxplot with stripchart, split by sex and genotype

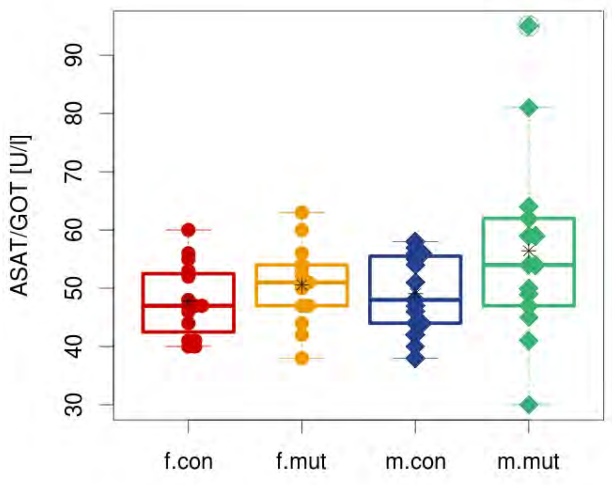

Figure 78
Clinical Chemistry: ASAT/GOT boxplot with stripchart, split by sex and genotype

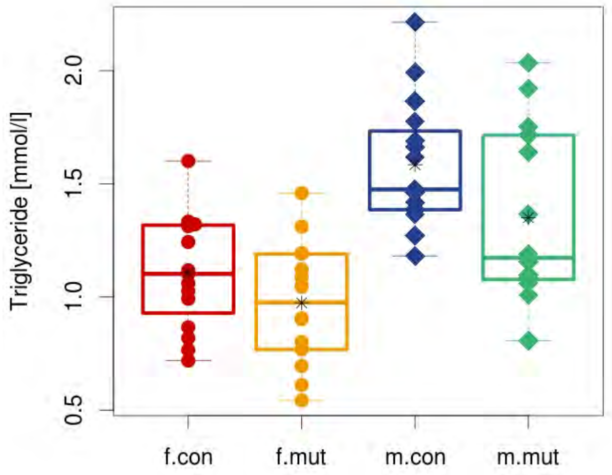

Figure 79
Clinical Chemistry: Triglyceride boxplot with stripchart, split by sex and genotype

Table 44
Clinical Chemistry: Lipid and glucose levels as well as selected enzyme activities in plasma of ad libitum fed mice. Means, standard deviations and p-values for genotype, sex and genotype × sex interaction effects calculated by a linear model.

	female		male		Linear model		
	control	mutant	control	mutant	genotype	sex	genotype:sex
	n=15	n=15	n=15	n=14			
	mean ± sd	mean ± sd	mean ± sd	mean ± sd	p-value	p-value	p-value
**Cholesterol** [mmol/l]	1.646 ± 0.179	1.695 ± 0.116[a]	2.298 ± 0.201	2.156 ± 0.217	0.336	< 0.001	0.051
**Triglyceride** [mmol/l]	1.107 ± 0.25	0.974 ± 0.272[a]	1.584 ± 0.285	1.351 ± 0.386	0.025	< 0.001	0.527
**ALAT/GPT** [U/l]	47 ± 23	47 ± 27[a]	61 ± 32	40 ± 14	0.107	0.597	0.136
**ASAT/GOT** [U/l]	48 ± 6	51 ± 7[a]	49 ± 7	56 ± 16	0.055	0.174	0.378
**alpha-Amylase** [U/l]	673.03 ± 182.15	684.87 ± 227.98[a]	625.06 ± 133.87	650.73 ± 319.21	0.751	0.489	0.907
**Glucose** [mmol/l]	13.78 ± 1.39	14.63 ± 1.28[a]	14.13 ± 1.28	13.74 ± 1.58	0.528	0.457	0.093
**LDH** [U/l]	255.2 ± 48.5	255.5 ± 54.1[a]	234.2 ± 69.3	229.4 ± 87.3	0.897	0.181	0.885
**Fructosamine** [umol/l]	350.3 ± 62.3	362.9 ± 104.2[a]	297 ± 14.1	292.4 ± 17.4	0.807	< 0.001	0.596

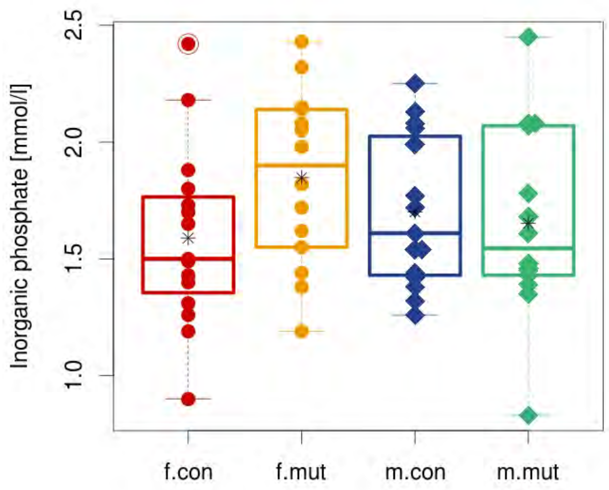

Figure 80
Clinical Chemistry: Inorganic phosphate boxplot with stripchart, split by sex and genotype

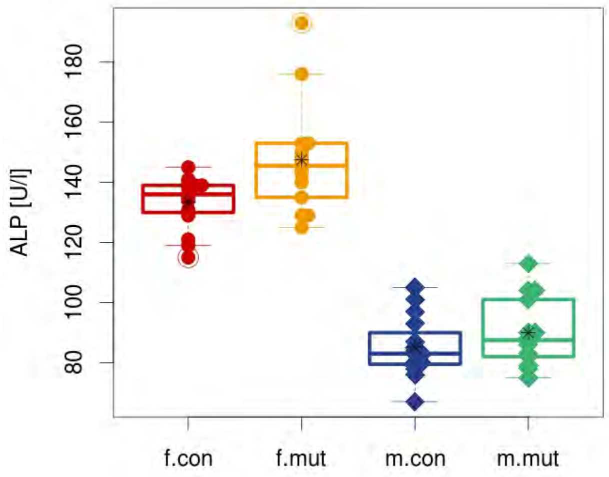

Figure 81
Clinical Chemistry: ALP boxplot with stripchart, split by sex and genotype

Table 45
Clinical Chemistry: Plasma concentrations of minerals, iron and ALP activity of ad libitum fed mice. Means, standard deviations and p-values for genotype, sex and genotype × sex interaction effects calculated by a linear model.

	female		male		Linear model		
	control	mutant	control	mutant	genotype	sex	genotype:sex
	n=15	n=15	n=15	n=14			
	mean ± sd	mean ± sd	mean ± sd	mean ± sd	p-value	p-value	p-value
**Calcium** [mmol/l]	2.32 ± 0.05	2.33 ± 0.05[a]	2.27 ± 0.08	2.24 ± 0.07	0.529	< 0.001	0.175
**Inorganic phosphate** [mmol/l]	1.59 ± 0.39	1.85 ± 0.37[a]	1.7 ± 0.33	1.65 ± 0.41	0.29	0.673	0.126
**Iron** [mumol/l]	23.749 ± 4.715	21.989 ± 3.265[a]	20.13 ± 1.453	20.4 ± 2.05	0.372	0.003	0.225
**ALP** [U/l]	133 ± 9	148 ± 18[a]	85 ± 10	90 ± 11	0.006	< 0.001	0.166
**Unsaturated iron binding capacity** [mumol/l]	29.5 ± 5.1	29.6 ± 5.1[a]	35.4 ± 4.2	35.1 ± 6.5	0.984	< 0.001	0.895
**Total iron binding capacity** [mumol/l]	53.2 ± 3.3	51.6 ± 3.4[a]	55.5 ± 4	55.5 ± 5.4	0.474	0.006	0.441
**Calc transferrin saturation** [%]	44.7 ± 8.5	42.9 ± 7.4[a]	36.4 ± 3.5	37.2 ± 6.2	0.76	< 0.001	0.478

Hematology

Hematological data indicated a mild increase in erythrocyte size of mutant animals, associated with decreased red blood cell counts, hemoglobin and hematocrit values mainly in male mutants. Since calculated values of mean corpuscular hemoglobin content (MCH) were also increased in mutants compared to controls, but cellular hemoglobin concentration (MCHC) was not significantly affected by the genotype, we consider our findings as a hint towards an effect of the mutation on hematopoiesis, rather than a secondary effect due to changes in plasma osmolarity. Furthermore, we saw increased white blood cell counts (WBC) in mutant mice as compared to controls.

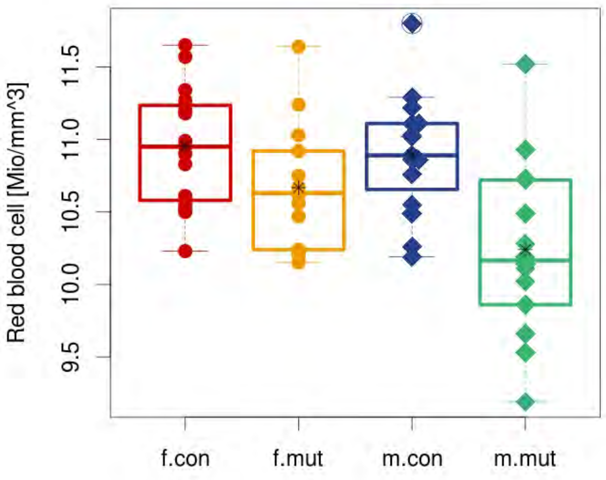

Figure 82
Hematology: Red blood cell boxplot with stripchart, split by sex and genotype

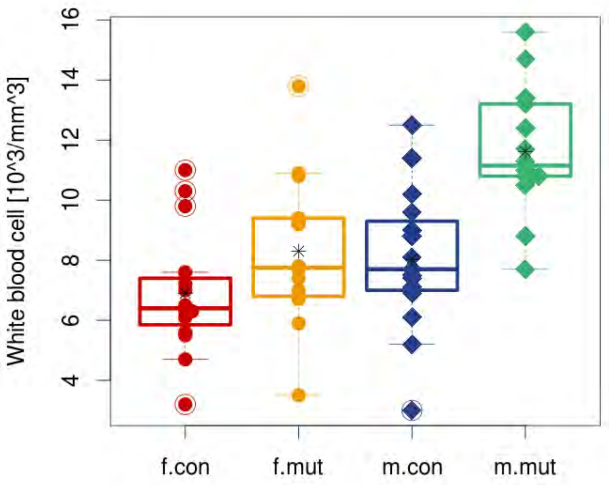

Figure 83
Hematology: White blood cell boxplot with stripchart, split by sex and genotype

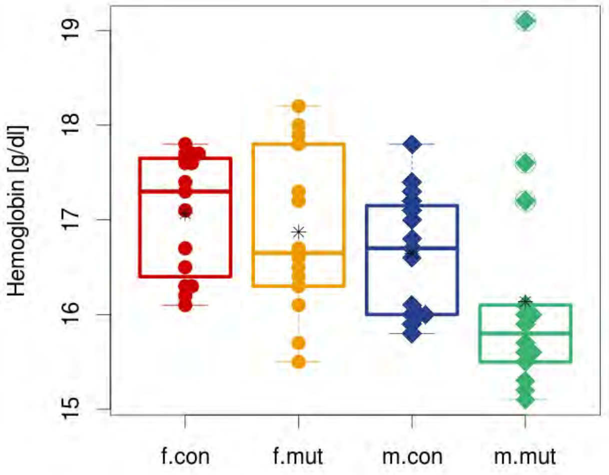

Figure 84
Hematology: Hemoglobin boxplot with stripchart, split by sex and genotype

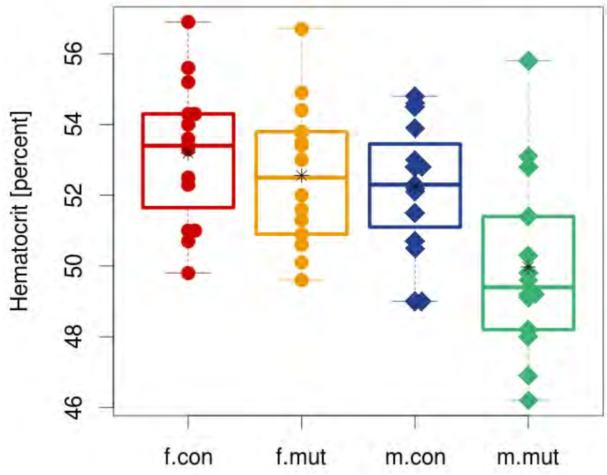

Figure 85
Hematology: Hematocrit boxplot with stripchart, split by sex and genotype

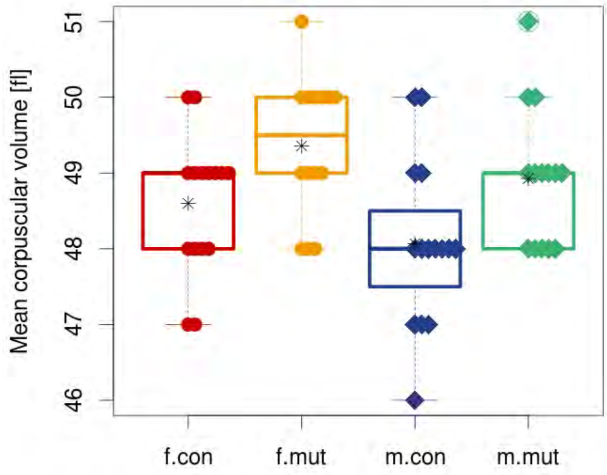

Figure 86
Hematology: Mean corpuscular volume boxplot with stripchart, split by sex and genotype

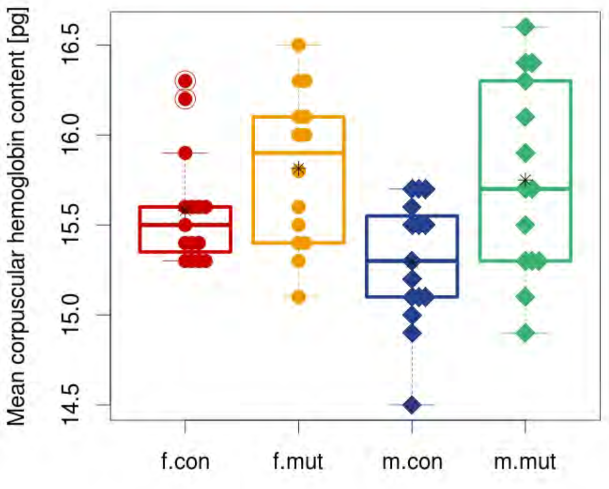

Figure 87
Hematology: Mean corpuscular hemoglobin content boxplot with stripchart, split by sex and genotype

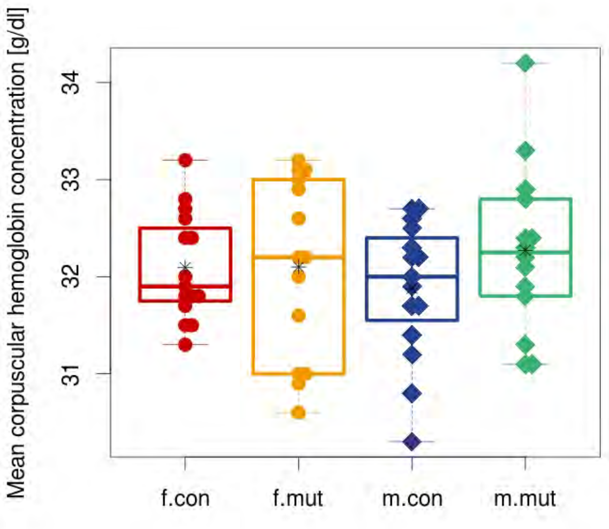

Figure 88
Hematology: Mean corpuscular hemoglobin concentration boxplot with stripchart, split by sex and genotype

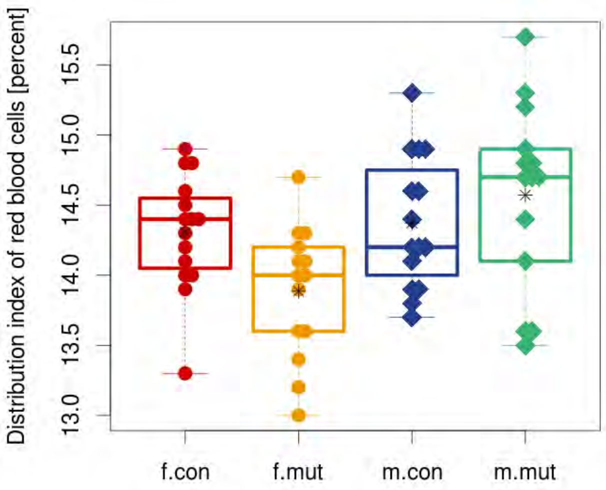

Figure 89
Hematology: Distribution index of red blood cells boxplot with stripchart, split by sex and genotype

Table 46
Hematology: Values measured in EDTA-blood samples. Means, standard deviations and p-values for genotype, sex and genotype × sex interaction effects calculated by a linear model.

	female		male		Linear model		
	control	mutant	control	mutant	genotype	sex	genotype:sex
	n=15	n=14	n=15	n=14			
	mean ± sd	mean ± sd	mean ± sd	mean ± sd	p-value	p-value	p-value
RBC [Mio/mm^3]	10.95 ± 0.42	10.67 ± 0.43	10.9 ± 0.41	10.24 ± 0.61	< 0.001	0.057	0.143
HGB [g/dl]	17.07 ± 0.64	16.87 ± 0.87	16.65 ± 0.64	16.14 ± 1.11	0.113	0.011	0.474
HCT [percent]	53.19 ± 1.99	52.56 ± 2.03	52.25 ± 1.85	49.97 ± 2.58	0.012	0.003	0.148
MCV [fl]	48.6 ± 0.91	49.36 ± 0.93	48.07 ± 1.1	48.93 ± 0.92	0.002	0.064	0.838
MCH [pg]	15.58 ± 0.32	15.81 ± 0.43	15.29 ± 0.35	15.75 ± 0.54	0.003	0.115	0.315
MCHC [g/dl]	32.09 ± 0.56	32.1 ± 0.93	31.88 ± 0.71	32.27 ± 0.86	0.331	0.918	0.348
RDW [percent]	14.31 ± 0.41	13.89 ± 0.47	14.37 ± 0.48	14.57 ± 0.66	0.412	0.007	0.026
WBC [10^3/mm^3]	6.9 ± 2.09	8.3 ± 2.53	8.03 ± 2.39	11.62 ± 2.12	< 0.001	0.001	0.075
PLT [10^3/mm^3]	1156.87 ± 130.41	1137.64 ± 118.91	1279.6 ± 132.79	1234.21 ± 111.49	0.326	0.001	0.69
MPV [fl]	6.29 ± 0.17	6.28 ± 0.14	6.37 ± 0.19	6.39 ± 0.14	0.978	0.031	0.75
PDW [fL]	5.65 ± 0.22	5.61 ± 0.2	5.73 ± 0.29	5.72 ± 0.23	0.684	0.139	0.828
PLCR [percent]	2.31 ± 0.48	2.36 ± 0.5	2.79 ± 0.78	2.69 ± 0.54	0.873	0.014	0.628
PCT [percent]	0.73 ± 0.09	0.71 ± 0.07	0.82 ± 0.09	0.79 ± 0.08	0.325	< 0.001	0.742

Discussion 4.9.3

Differences between mutant and control mice detected by the Clinical Chemistry and Hematology Screen were mostly mild to moderate.

We saw some mild differences in plasma parameters associated with energy metabolism, namely fasting glucose level in the IpGTT as well as cholesterol and triglyceride levels in the fasting ad fed state. However, theses differences were quite small and unlikely to indicate a direct effect on energy metabolism, but rather secondary effects or accidental findings.

More prominent were the genotype-related differences in plasma protein levels. Here we saw a decrease in plasma total protein and albumin and an increase in creatinine levels. The decreases for total protein and albumin were within a similar range, suggesting that decreased albumin levels were the main reason for the decrease in total protein. Albumin is the major component of plasma proteins and exclusively sythesized in the liver. In cases of albumin loss, e.g. due to albuminuria or increased albumin degradation, albumin and lipoprotein synthesis in the liver is usually activated resulting in almost normal albumin values and increased cholesterol levels in plasma (Stein et al. 2011). Since cholesterol levels were not increased, the decrease of albumin is unlikely to be due to increased loss but rather caused by decreased sythesis.

We also found a mild increase in plasma creatinine levels. Creatinine is a product of creatine metabolism in musculature and therefore creatinine levels usually correlate with total muscle mass. However, since mutant mice were rather lighter than controls, an increase in muscle mass can not be the cause for this difference. Creatinine is eliminated from blood by renal filtration and therefore dependent on renal function. However, in this case further indications of renal dysfunction are missing. Further investigations would be necessary to test reproducibility of this finding and to clarify the cause.

We also found slightly increased ALP activities in plasma. Plasma ALP activity is mainly derived from two different sources - bone metabolism and liver cells. However, small amounts are also produced by the gut. Taking the finding of reduced bone masss and bone content in female mutant mice (Dysmorphology Screen) into account, increased ALP activity could be a sign of effects on bone metabolism.

The hematological analyses revealed mild macrocytosis (MCV) with reduced erythrocyte counts (RBC) and increased cellular hemoglobin content (MCH) in mutant animals. Such changes of the peripheral blood cell counts can be observed in case of an impaired cellcycle during erythropoiesis, e.g. as a consequence of vitamin B12 or folic acid deficiency, or due to an impaired DNA-replication in the case of Diamond-Blackfan anemia. Furthermore white blood cell counts were increased in mutants, although the Immunology Screen did not detect major effects on leukocyte composition in peripheral blood.

In total we found mainly mild differences, that might indicate effects on energy metabolism, protein metabolism or hematopoiesis. However, since the differences

are small, further investigations are recommended to confirm genotype-dependency of these findings and elucidate underlying cause.

Immunology Screen 4.10

Summary 4.10.1

We did not find significant differences in the levels of antibodies, and only subtle sex-dependent changes in the frequencies of several minor leukocyte subpopulations.

Results 4.10.2

Flow Cytometry

First, we determined the frequencies of the following main leukocyte lineages: B cells (CD19 positive cells; B-1 B cells = CD5positive Bcells; B-2 B cells = CD5negative), T cells (CD5positive CD3positive NKnegative), granulocytes (CD11bpositive Ly6G positive), NK cells (NK1.1/NKp46positive), NKT cells, and monocytes (subdivided into Ly6C positive and Ly6Cnegative) within one antibody panel (panel 1) as the percentages of all CD45positive cells. Herein, we found a significantly higher frequency of granulocytes in samples from male mutants and a lower frequency of granulocytes and Ly6Cpositive monocytes in female mutants. Monocytes were further subdivided concerning their Ly6C expression (high, medium, negative) and their CD11c expression (positive, negative). There, we found that the frequencies - referring to all monocytes (CD11b positive cells, negative for Gr-1 and NK cell markers NKp46 and NK1.1) - of Ly6C high expressing cells were decreased in female mutants, whereas in male mutants the frequencies of Ly6C medium expressing cells and of Ly6C negative cells, both of these populations negative for CD11c, were decreased.

With a further antibody panel (2) we analyzed the T cell compartment. A subdivision of T cells into CD4 and CD8 single positive, CD4 CD8 double positive, and CD4 CD8 double T cells revealed no differences in the proportions of main T cell subsets. The determination of gamma-delta TCR positive cells within the T cell population revealed a slightly increased frequency in male mutants. Within the CD4 T cell cluster the frequency of CD25 expressing cells and of Ly6C expressing cells was lower in mutant males, whereas no differences between mutants and controls were found in the frequencies of CD8 subpopulations.

B cells (CD19 positive cells) were specified concerning the expression of the B cell markers IgD, IgM, CD21, CD23, MHC class 2, B220 and CD5 with antibody panel 3. Also, the frequencies of cells, expressing CD11b, Ly6C were determined in staining with antibody panel 1 (main leukocyte subsets). Subdividing the B2- B cell (CD5 negative Bcells) by Boolean gates concerning the co-expression of IgD, IgM, CD21 and CD23, revealed a similar B cell pattern in all groups, being the main population of a mature naive B cell phenotype (CD21 positive, CD23 positive, IgD positive, IgM negative). In males we found, however, slightly decreased frequencies of IgD and IgM co-expressing cells negative for CD21 either positive or negative for CD23. Furthermore, the frequency of CD11b expressing cells within the B cell compartment was slightly increased in male mutants.

The analysis of NK cells, concerning the expression of CD11b, CD11c, CD44, CD62L and Ly6C revealed a slightly decreased frequency of CD62L positive cells within the NK cell compartment in male mutants.

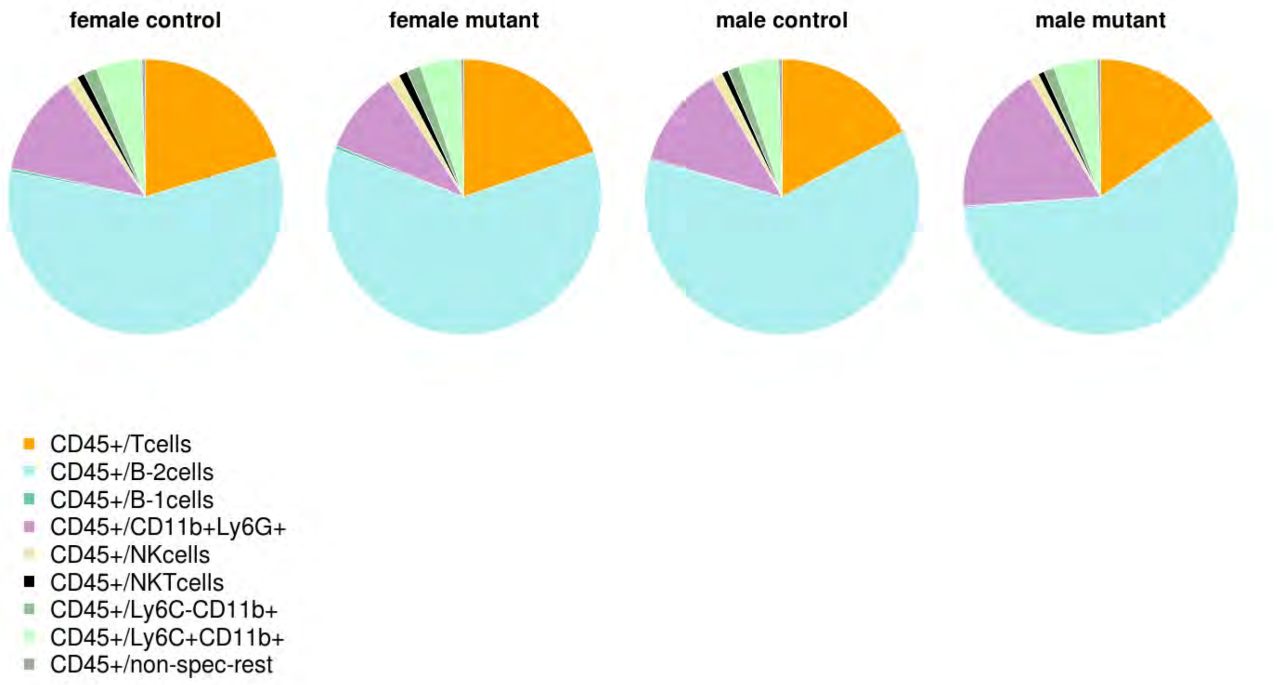

Figure 90
Main leukocyte populations: Leukocytes pie chart, split by sex and genotype

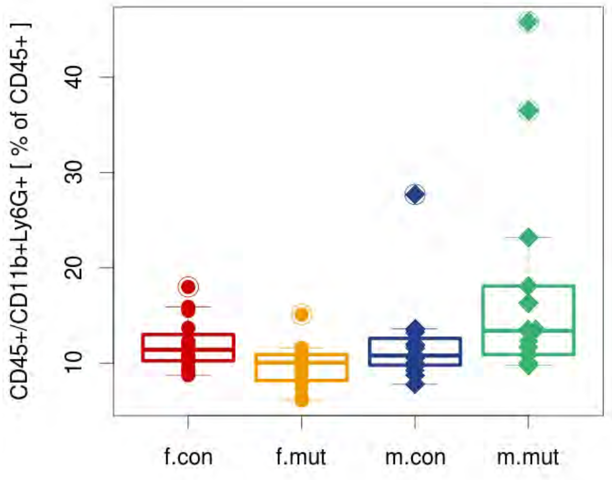

Figure 91
Main leukocyte populations: CD45+/CD11b+Ly6G+ boxplot with stripchart, split by sex and genotype

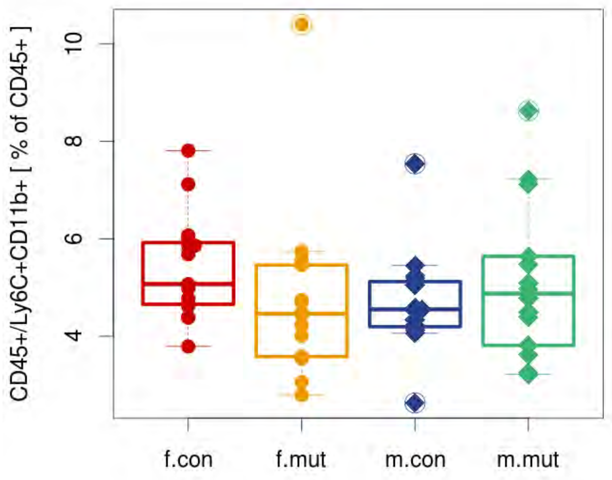

Figure 92
Main leukocyte populations: CD45+/Ly6C+CD11b+ boxplot with stripchart, split by sex and genotype

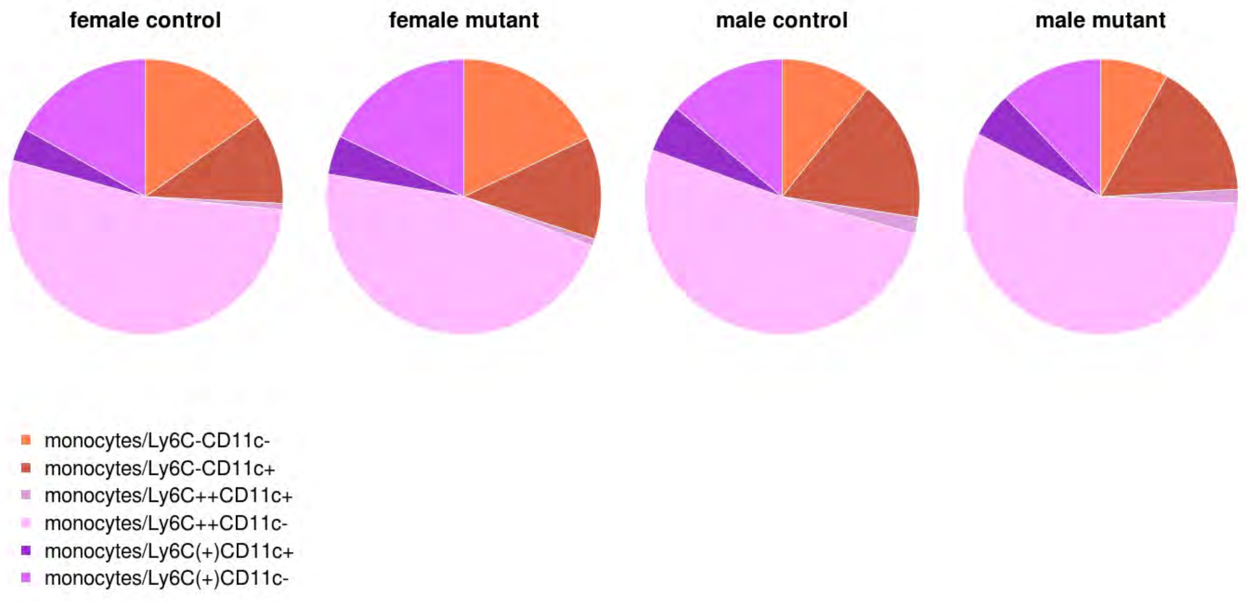

Figure 93
Monocyte subsets: Monocyte pie chart, split by sex and genotype

Table 47
Main leukocyte populations: Frequencies of main leukocyte populations in peripheral blood (protocol without erythrocyte lysis) [percentage of all leukocytes (CD45+ cells)]. Medians, first and third quartile and p-values calculated by a Wilcoxon rank-sum test
[a] Number not based on the full number of animals (missing values)

	female		male		female	male	overall
	control	mutant	control	mutant			
	n=15	n=14	n=15	n=14			
	median [25%, 75%]	median [25%, 75%]	median [25%, 75%]	median [25%, 75%]	p-value	p-value	p-value
CD45+/Tcells	20.4 [18.3 , 21.75]	19.65 [19.32 , 20.55]	17.2 [15.55 , 18.4]	15 [13.47 , 16.15]	0.554	0.063	0.168
CD45+/B-2cells	57.9 [52.9 , 62.05]	60.65 [57.67 , 65.28]	63.3 [59.9 , 65.4]	61.35 [58.67 , 65.22]	0.131	0.431	0.607
CD45+/B-1cells	0.35 [0.34 , 0.47]	0.32 [0.29 , 0.52]	0.17 [0.15 , 0.24]	0.14 [0.12 , 0.21]	0.974	0.335	0.743
CD45+/CD11b+Ly6G+	11.4 [10.25 , 13]	10.05 [8.36 , 10.82]	10.8 [9.8 , 12.6]	13.4 [11.1 , 17.65]	0.024	0.042	0.929
CD45+/NKcells	1.33 [1.2 , 1.47]	1.32 [1.2 , 1.48]	1.16 [1.1 , 1.35]	1.07 [0.98 , 1.22]	0.889	0.12	0.283
CD45+/NKTcells	0.87 [0.74 , 1.07]	1.15 [0.64 , 1.31]	0.78 [0.68 , 0.84]	0.8 [0.64 , 0.85]	0.23	0.974	0.554
CD45+/Ly6C-CD11b+	1.5 [1.32 , 1.69]	1.46 [1.28 , 1.75]	1.36 [1.21 , 1.53]	1.17 [1.02 , 1.42]	0.889	0.155	0.29
CD45+/Ly6C+CD11b+	5.07 [4.66 , 5.92]	4.46 [3.69 , 5.28]	4.55 [4.2 , 5.12]	4.88 [3.96 , 5.6]	0.04	0.583	0.347
CD45+/non-spec-rest	0.41 [0.36 , 0.48]	0.34 [0.3 , 0.37]	0.39 [0.34 , 0.44]	0.34 [0.32 , 0.4]	0.07	0.246	0.034

Table 48
Monocyte subsets: Frequencies of monocyte subsets in peripheral blood (protocol without erythrocyte lysis) [percentage of all monocytes]. Medians, first and third quartile and p-values calculated by a Wilcoxon rank-sum test
[a] Number not based on the full number of animals (missing values)

	female		male		female	male	overall
	control	mutant	control	mutant			
	n=15	n=14	n=15	n=14			
	median [25%, 75%]	median [25%, 75%]	median [25%, 75%]	median [25%, 75%]	p-value	p-value	p-value
monocytes/Ly6C-CD11c-	15 [12.95 , 17.3]	17.85 [14.72 , 22.23]	11.3 [9.05 , 12.65]	8.71 [5.75 , 10.21]	0.087	0.013	0.663
monocytes/Ly6C-CD11c+	10.7 [9.46 , 12.3]	11.4 [9.64 , 13.38]	16.6 [13.85 , 19.85]	15.4 [13.88 , 17.82]	0.371	0.444	0.899
monocytes/Ly6C++CD11c+	0.66 [0.54 , 0.91]	0.71 [0.62 , 0.98]	1.92 [1.48 , 2.27]	1.42 [1.27 , 1.83]	0.497	0.19	0.838
monocytes/Ly6C++CD11c-	53.8 [48.4 , 56.5]	47.55 [42.92 , 49.12]	50.1 [46.45 , 54.7]	56.5 [53.25 , 63.58]	0.027	0.061	0.802
monocytes/Ly6C(+)CD11c+	3.65 [3.28 , 4.19]	4.08 [3.77 , 4.37]	5.53 [4.84 , 6.58]	5.61 [4.82 , 6.25]	0.083	0.583	0.635
monocytes/Ly6C(+)CD11c-	16.9 [14.95 , 18.7]	18.1 [16 , 20.4]	13.6 [13.2 , 15]	11.45 [9.84 , 12.85]	0.326	0.02	0.575

Table 49
Main leukocyte populations: Summary of total leukocyte count

	Min	Median	Mean	Max
cell_count	9782	32210	33870	74615

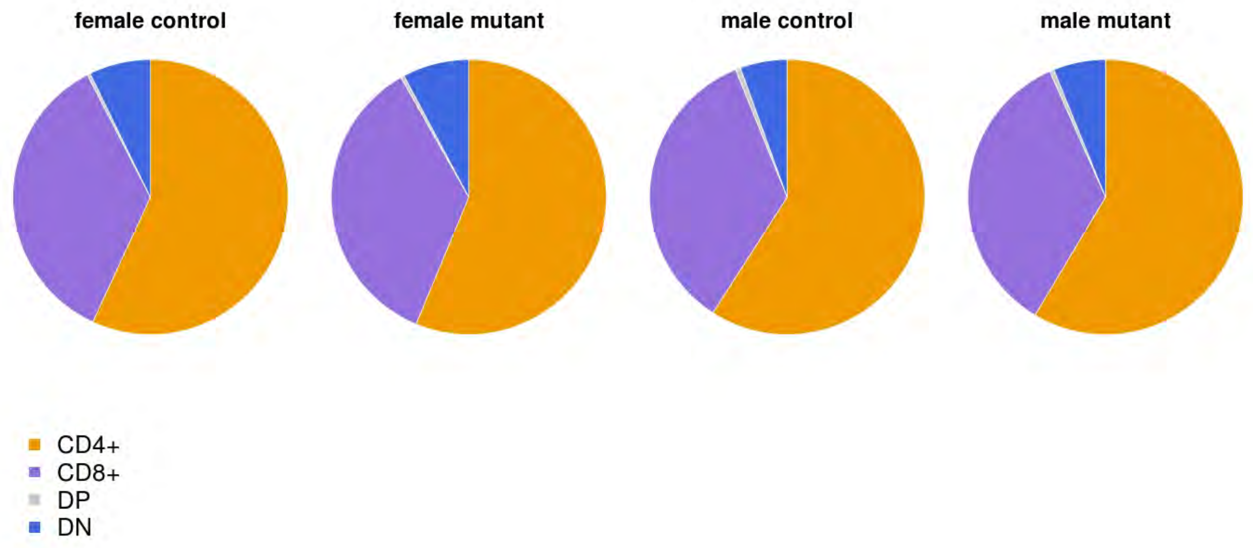

Figure 94
T cell subsets: T cell subpopulations pie chart, split by sex and genotype

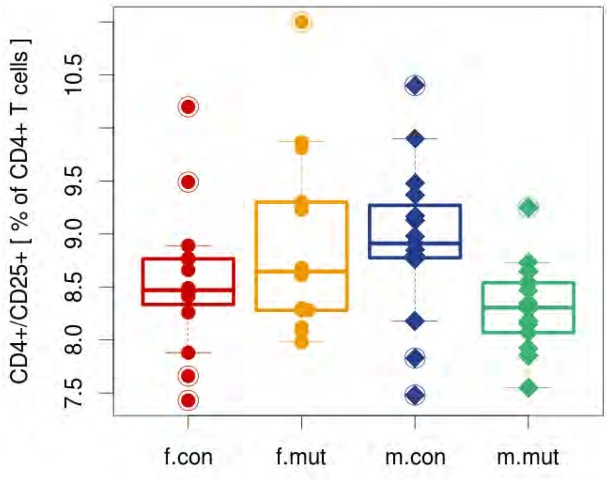

Figure 95
T cell subsets: CD4+/CD25+ boxplot with stripchart, split by sex and genotype

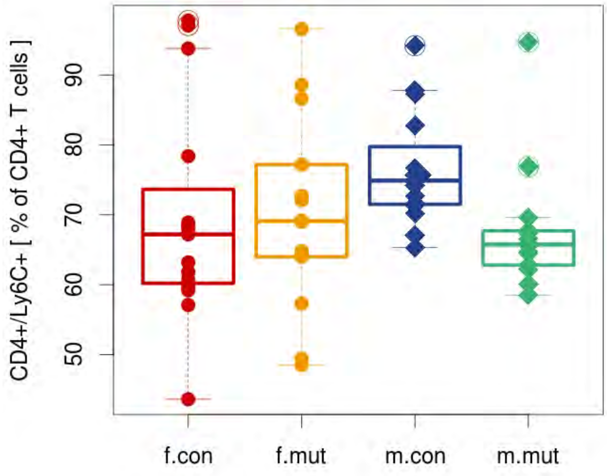

Figure 96
T cell subsets: CD4+/Ly6C+ boxplot with stripchart, split by sex and genotype

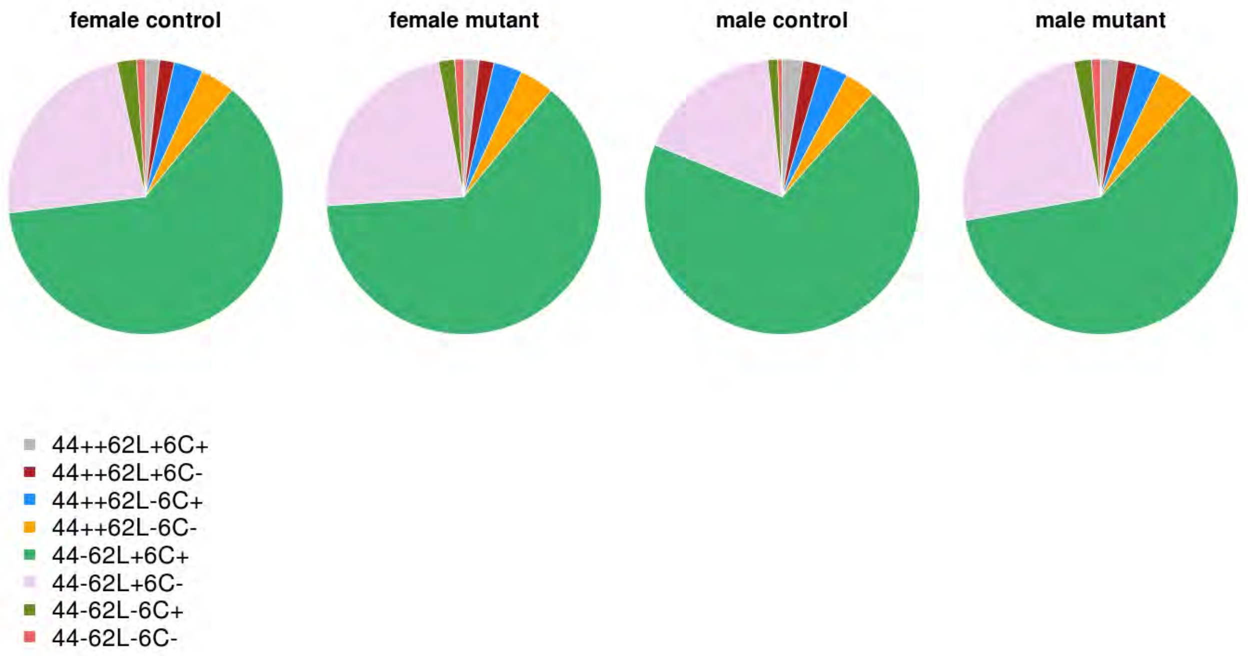

Figure 97
T cell subsets: CD4 T cell subpopulations pie chart, split by sex and genotype

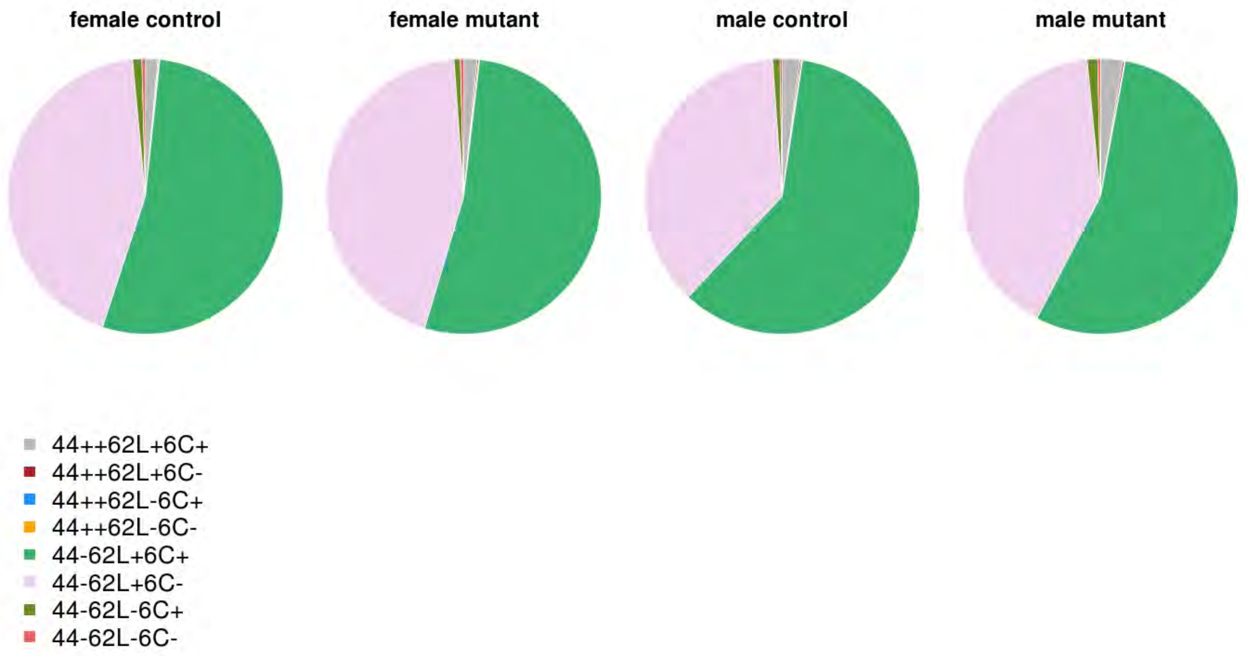

Figure 98
T cell subsets: CD8 T cell subpopulations pie chart, split by sex and genotype

Table 50
NK cell subsets: Frequencies of NK cell subpopulations [percentage of NK cells]. Medians, first and third quartile and p-values calculated by a Wilcoxon rank-sum test
[a] Number not based on the full number of animals (missing values)

	female		male		female	male	overall
	control	mutant	control	mutant			
	n=15	n=14	n=15	n=14			
	median [25%, 75%]	median [25%, 75%]	median [25%, 75%]	median [25%, 75%]	p-value	p-value	p-value
NK/CD11b+	68 [63.15 , 71.05]	68.6 [66.4 , 72.15]	65.6 [62.3 , 67.25]	67 [64.22 , 68.78]	0.505	0.265	0.205
NK/CD11c+	16.9 [12.95 , 20.55]	17.85 [14.53 , 20.25]	21.5 [19.85 , 24.95]	20.2 [19.52 , 23.12]	0.629	0.37	0.702
NK/44+	6.96 [6.22 , 8.61]	7.96 [7.3 , 9.67]	8.03 [6.56 , 9.46]	7.64 [6.36 , 9.22]	0.205	0.847	0.514
NK/62L+	71 [69.7 , 75]	72.45 [68.4 , 77.8]	85.9 [79.85 , 88.15]	79.25 [77.55 , 81.7]	0.715	0.02	0.442
NK/Ly6C+	35.1 [31.4 , 41.75]	34.4 [31.93 , 38.27]	35.1 [33.4 , 41.45]	32.3 [30.6 , 38.35]	0.805	0.095	0.181

Table 51
T cell subsets: Frequencies of main T cell subpopulations (CD4, CD8, gamma-delta) [percentage of T cells]. Medians, first and third quartile and p-values calculated by a Wilcoxon rank-sum test
[a] Number not based on the full number of animals (missing values)

	female		male		female	male	overall
	control	mutant	control	mutant			
	n=15	n=14	n=15	n=14			
	median [25%, 75%]	median [25%, 75%]	median [25%, 75%]	median [25%, 75%]	p-value	p-value	p-value
T/CD4+	57.2 [54.95 , 58.05]	56.5 [53.23 , 58.75]	59 [57.8 , 59.75]	58 [57.23 , 59.7]	0.691	0.554	0.618
T/CD8+	36.4 [35.25 , 36.65]	35.9 [33.92 , 36.83]	35 [34.9 , 36.05]	35.15 [32.98 , 36.55]	0.771	0.738	0.725
T/DP	0.36 [0.24 , 0.4]	0.38 [0.32 , 0.44]	0.56 [0.51 , 0.68]	0.55 [0.36 , 0.64]	0.37	0.568	0.954
T/DN	6.92 [5.93 , 8.07]	7.16 [6.06 , 9.48]	5.49 [4.63 , 6.12]	6.08 [5.57 , 6.42]	0.554	0.109	0.183
T/gdTCR+	1.9 [1.73 , 2.5]	2.23 [1.73 , 2.9]	1.36 [1.15 , 1.56]	1.72 [1.52 , 1.83]	0.505	0.03	0.1
gdTCR+/DN	72.4 [69.8 , 75.8]	75.55 [73.22 , 79.05]	65.4 [60.95 , 70.85]	65.8 [61.65 , 69.22]	0.085	0.723	0.298

Table 52
T cell subsets: Frequencies of CD4 AND CD8 T cell subpopulations (CD25, CD44, CD62L, Ly6C). Medians, first and third quartile and p-values calculated by a Wilcoxon rank-sum test
[a] Number not based on the full number of animals (missing values)

	female		male		female	male	overall
	control	mutant	control	mutant			
	n=15	n=14	n=15	n=14			
	median [25%, 75%]	median [25%, 75%]	median [25%, 75%]	median [25%, 75%]	p-value	p-value	p-value
CD4+/CD25+	8.47 [8.34 , 8.77]	8.64 [8.28 , 9.29]	8.91 [8.78 , 9.27]	8.3 [8.09 , 8.52]	0.47	0.006	0.194
CD4+CD25+/44++	11.9 [9.56 , 13.75]	10.45 [8.95 , 13.07]	11.5 [9.22 , 13.35]	15.25 [10.6 , 16.43]	0.406	0.112	0.46
CD4+/CD44++	10.9 [10.25 , 12.1]	10.65 [9.75 , 11.7]	11.4 [10.5 , 13.05]	11.9 [9.99 , 12.78]	0.628	0.872	0.838
CD4+/CD62L+	89.4 [88.5 , 90.5]	89.55 [87.6 , 91.75]	91.5 [90.55 , 93.45]	89.3 [88.72 , 90.8]	0.723	0.055	0.263
CD4+/Ly6C+	67.2 [60.2 , 73.65]	69.1 [64.17 , 76.08]	74.9 [71.5 , 79.75]	65.75 [63.23 , 67.62]	0.621	0.002	0.131
CD8+/CD25+	1.29 [1.09 , 1.6]	1.28 [1.02 , 1.8]	1.27 [1.06 , 1.57]	1.08 [1.01 , 1.47]	0.706	0.406	0.331
CD8+/CD44++	1.49 [1.34 , 1.89]	1.82 [1.34 , 2.34]	2.2 [1.72 , 2.72]	3.02 [1.9 , 3.7]	0.444	0.247	0.234
CD8+/CD62L+	98.3 [98.05 , 98.8]	98.65 [98.4 , 99.38]	98.9 [98.3 , 99.25]	98.85 [98.62 , 99.08]	0.114	0.821	0.266
CD8+/Ly6C+	48 [44.35 , 55.7]	48.15 [44.33 , 59.95]	60.4 [57.05 , 64.95]	56.9 [53.67 , 59.88]	0.992	0.087	0.484

Table 53
T cell subsets: Frequencies of CD4 and CD8 T cell subpopulations (Boolean gates CD44 high, CD62L, Ly6C). Medians, first and third quartile and p-values calculated by a Wilcoxon rank-sum test
[a] Number not based on the full number of animals (missing values)

	female		male		female	male	overall
	control	mutant	control	mutant			
	n=15	n=14	n=15	n=14			
	median [25%, 75%]	median [25%, 75%]	median [25%, 75%]	median [25%, 75%]	p-value	p-value	p-value
4+/44++62L+6C+	1.12 [0.94 , 2.41]	1.48 [1.14 , 2.04]	2.44 [2.15 , 2.65]	2.02 [1.68 , 2.35]	0.406	0.075	0.504
4+/44++62L+6C-	1.96 [1.61 , 2.25]	1.82 [1.33 , 2.25]	2.21 [1.62 , 2.76]	2.21 [1.93 , 2.65]	0.974	0.906	0.893
4+/44++62L-6C+	3.05 [2.37 , 4.2]	2.92 [2.23 , 3.74]	3.05 [2.38 , 3.88]	2.89 [1.86 , 3.21]	0.856	0.47	0.499
4+/44++62L-6C-	4.73 [3.19 , 4.92]	4.11 [3.15 , 4.79]	3.43 [3.15 , 4.54]	4.63 [3.96 , 5.09]	0.771	0.05	0.305
4+/44-62L+6C+	60 [54.25 , 67.2]	62.6 [57.1 , 70.05]	67.8 [65.7 , 72.75]	59.95 [56.65 , 61.83]	0.621	0.001	0.114
4+/44-62L+6C-	25.5 [19.55 , 30.9]	24.15 [19.12 , 28.43]	19 [13.75 , 20.6]	26.35 [23.77 , 27.7]	0.739	0.002	0.109
4+/44-62L-6C+	2.1 [1.56 , 2.52]	1.74 [1.4 , 2.23]	0.77 [0.52 , 1.4]	1.66 [1.05 , 2.05]	0.407	0.102	0.554
4+/44-62L-6C-	0.9 [0.63 , 1.39]	0.9 [0.5 , 1.55]	0.47 [0.3 , 0.63]	0.88 [0.55 , 1.17]	0.906	0.022	0.1
8+/44++62L+6C+	1.27 [1.15 , 1.75]	1.57 [1.12 , 1.86]	1.7 [1.57 , 2.42]	2.71 [1.72 , 3.33]	0.561	0.201	0.277
8+/44++62L+6C-	0.11 [0.05 , 0.2]	0.16 [0.11 , 0.21]	0.16 [0.1 , 0.2]	0.2 [0.1 , 0.28]	0.185	0.336	0.121
8+/44++62L-6C+	0.08 [0.03 , 0.12]	0.04 [0.02 , 0.08]	0.1 [0.04 , 0.12]	0.06 [0.02 , 0.14]	0.551	0.673	0.408
8+/44++62L-6C-	0.03 [0 , 0.05]	0.03 [0 , 0.05]	0.03 [0 , 0.04]	0.03 [0 , 0.05]	0.745	0.973	0.801
8+/44-62L+6C+	45.2 [42.2 , 52.35]	45.35 [42.45 , 58.35]	55.1 [54.35 , 62]	53.6 [50.02 , 55]	1	0.075	0.514
8+/44-62L+6C-	51.3 [43.7 , 55.15]	51.1 [39.55 , 55.15]	38.8 [34.65 , 42.45]	42.6 [39.58 , 45.75]	0.983	0.109	0.519
8+/44-62L-6C+	0.99 [0.7 , 1.42]	0.78 [0.47 , 0.92]	0.67 [0.54 , 1.19]	0.76 [0.58 , 1.07]	0.071	0.644	0.247
8+/44-62L-6C-	0.41 [0.31 , 0.51]	0.42 [0.18 , 0.57]	0.22 [0.16 , 0.36]	0.28 [0.16 , 0.34]	0.974	0.755	0.935

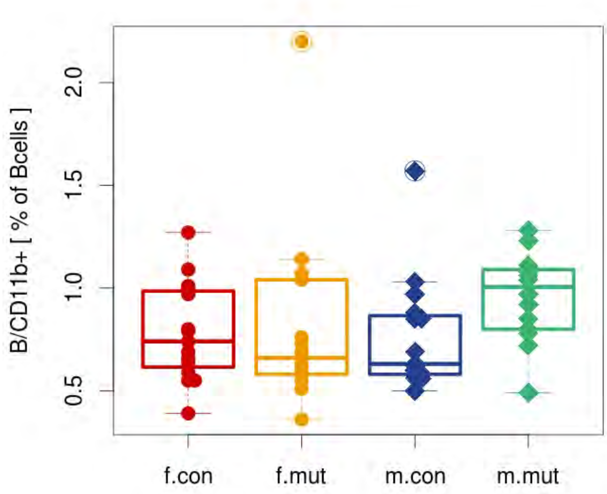

Figure 99
B cell subsets: B/CD11b+ boxplot with stripchart, split by sex and genotype

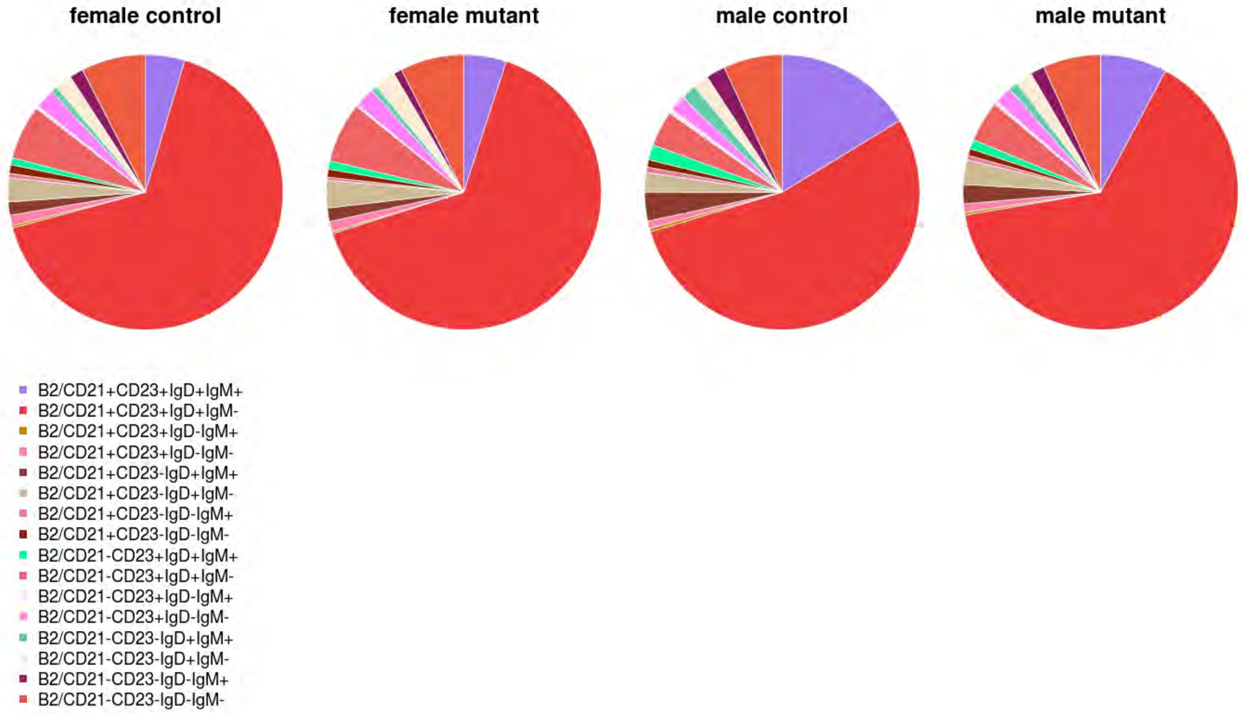

Figure 100
B cell subsets: B-2 B cell subpopulations pie chart, split by sex and genotype

Table 54

B cell subsets: Frequencies of B cell subpopulations [percentage of all B cells]. Medians, first and third quartile and p-values calculated by a Wilcoxon rank-sum test

[a] Number not based on the full number of animals (missing values)

	female		male		female	male	overall
	control	mutant	control	mutant			
	n=15	n=14	n=15	n=14			
	median [25%, 75%]	median [25%, 75%]	median [25%, 75%]	median [25%, 75%]	p-value	p-value	p-value
B/CD11b+	0.74 [0.62 , 0.98]	0.66 [0.58 , 0.97]	0.63 [0.58 , 0.86]	1 [0.81 , 1.09]	0.659	0.016	0.143
B/Ly6C+	0.46 [0.38 , 0.64]	0.44 [0.34 , 0.64]	0.54 [0.46 , 0.69]	0.67 [0.56 , 0.8]	0.675	0.182	0.586
B/IgD+	84.8 [82.4 , 86.55]	85.95 [84.1 , 86.75]	86.6 [83.1 , 87.65]	86.05 [84.72 , 88.28]	0.275	0.872	0.446
B/IgM+	9.52 [5.75 , 15.05]	8.55 [5.42 , 13.07]	19.4 [12.25 , 22.2]	13.95 [9.12 , 16.05]	0.813	0.115	0.256
B/CD21+	77.9 [73.8 , 82.5]	77.95 [74.6 , 80.17]	79.2 [77.1 , 81.8]	81.1 [78.95 , 82.9]	0.871	0.247	0.679
B/CD23+	81.9 [80.05 , 83.55]	81.75 [80.75 , 82.3]	79.2 [77.45 , 82.95]	81 [78.78 , 84.7]	0.957	0.19	0.351
B/B220+	99.5 [99.4 , 99.6]	99.55 [99.5 , 99.6]	99.6 [99.6 , 99.65]	99.65 [99.6 , 99.7]	0.445	0.37	0.243
B/MHC2+	92.7 [91.3 , 94.25]	92.55 [91.5 , 93.38]	93.9 [92.95 , 94.55]	93.45 [92.78 , 94.88]	0.821	0.821	0.874
B/CD5+	1.25 [1.13 , 1.47]	1.3 [1.15 , 1.42]	1.02 [0.86 , 1.15]	1.06 [0.94 , 1.27]	0.855	0.31	0.376

Table 55

B cell subsets: B-2 B cell subpopulations (Boolean gates CD21,CD23, IgD, IgM). Medians, first and third quartile and p-values calculated by a Wilcoxon rank-sum test

[a] Number not based on the full number of animals (missing values)

	female		male		female	male	overall
	control	mutant	control	mutant			
	n=15	n=14	n=15	n=14			
	median [25%, 75%]	median [25%, 75%]	median [25%, 75%]	median [25%, 75%]	p-value	p-value	p-value
B2/CD21+CD23+IgD+IgM+	4.6 [2.19 , 6.16]	3.27 [1.79 , 4.92]	7.52 [5.64 , 10.95]	6.04 [3.31 , 6.76]	0.613	0.105	0.225
B2/CD21+CD23+IgD+IgM-	65.1 [62.55 , 69.7]	65.45 [64.03 , 67.35]	61.6 [59.05 , 64.8]	65.75 [61.35 , 70.92]	0.683	0.131	0.324
B2/CD21+CD23+IgD-IgM+	0.29 [0.1 , 0.44]	0.2 [0.12 , 0.3]	0.36 [0.24 , 0.5]	0.3 [0.24 , 0.44]	0.382	0.54	0.26
B2/CD21+CD23+IgD-IgM-	1.2 [0.94 , 1.34]	1.12 [0.93 , 1.3]	0.82 [0.68 , 1]	1 [0.87 , 1.09]	0.659	0.155	0.618
B2/CD21+CD23-IgD+IgM+	1.43 [0.92 , 2]	1.3 [0.82 , 2.46]	2.9 [1.78 , 3.63]	2.48 [1.12 , 3.02]	0.992	0.354	0.47
B2/CD21+CD23-IgD+IgM-	2.83 [2.36 , 3.12]	3.33 [2.49 , 4.06]	2.43 [1.84 , 2.96]	3.04 [2.52 , 3.41]	0.201	0.131	0.049
B2/CD21+CD23-IgD-IgM+	0.54 [0.21 , 0.73]	0.34 [0.18 , 0.46]	0.74 [0.38 , 0.79]	0.52 [0.32 , 0.72]	0.161	0.326	0.092
B2/CD21+CD23-IgD-IgM-	0.89 [0.77 , 1.21]	0.96 [0.82 , 1.05]	0.72 [0.6 , 1.06]	0.76 [0.65 , 0.92]	0.872	0.805	0.911
B2/CD21-CD23+IgD+IgM+	0.8 [0.44 , 1.03]	0.86 [0.74 , 1.09]	1.43 [1.22 , 1.6]	1.02 [0.88 , 1.23]	0.54	0.016	0.376
B2/CD21-CD23+IgD+IgM-	5.25 [4.51 , 8]	6.39 [5.02 , 8.04]	3.51 [2.84 , 4.72]	4.21 [3.36 , 5.94]	0.477	0.256	0.257
B2/CD21-CD23+IgD-IgM+	0.21 [0.14 , 0.52]	0.18 [0.13 , 0.29]	0.42 [0.28 , 0.64]	0.38 [0.28 , 0.47]	0.315	0.371	0.152
B2/CD21-CD23+IgD-IgM-	2.3 [1.86 , 2.9]	2.38 [1.99 , 2.69]	1.83 [1.66 , 2.06]	1.83 [1.66 , 2.47]	0.991	0.66	0.624
B2/CD21-CD23-IgD+IgM+	0.69 [0.42 , 0.99]	0.69 [0.58 , 0.8]	1.61 [1.21 , 2.28]	1.15 [0.94 , 1.4]	0.914	0.026	0.208
B2/CD21-CD23-IgD+IgM-	1.82 [1.44 , 2.57]	2.39 [1.9 , 2.98]	1.76 [1.31 , 2.38]	1.88 [1.56 , 2.14]	0.087	0.755	0.154
B2/CD21-CD23-IgD-IgM+	1.25 [0.55 , 2.63]	0.76 [0.45 , 1.4]	2.1 [1.14 , 3.09]	1.83 [1.18 , 2.22]	0.23	0.316	0.098
B2/CD21-CD23-IgD-IgM-	7.96 [6.44 , 9.37]	7.91 [6.66 , 8.74]	6.75 [6.05 , 7.93]	6.51 [5.9 , 7.43]	0.822	0.683	0.868

Table 56
B cell subsets: Summary of total leukocyte count and B cells count

	Min	Median	Mean	Max
acquired.cells	17505	66010	67570	111000
acquired.B.cells	10857	37750	39260	70323

Immunoglobulin Levels (except IgE)

We analyzed the levels of IgM, IgG1, IgG2b and IgG3 with a bead-array (bioplex) from the same sample of blood plasma of each mouse and did not see significant differences between the groups. We furthermore determined the presence of autoantibodies (anti-DNA, and anti-Ig) in blood plasma via ELISA but neither found no hints towards a genotype-related change.

Discussion 4.10.3

The found subtle differences are of unclear relevance and might represent physiological changes due to unknown factors.

4.11 Allergy Screen

4.11.1 Summary

The screening did not uncover an IgE phenotype or disturbances in the skin.

4.11.2 Results

Total IgE in Plasma

The analysis of total IgE levels in plasma did not reveal genotype-specific differences.

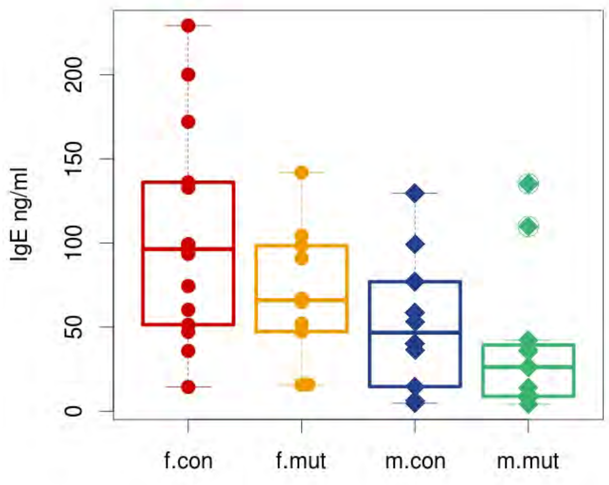

Figure 101

Total IgE in Plasma: IgE boxplot with stripchart, split by sex and genotype

We observed no statistically significant differences in the IgE levels between control and mutants mice, levels all groups of animal were within the normal range of IgE from C57BL/6 background.

Table 57
Total IgE in Plasma: Medians, first and third quartile and p-values calculated by a Wilcoxon rank-sum test

	female		male		female	male	overall
	control	mutant	control	mutant			
	14	10	10	11			
	median	median	median	median	p-value	p-value	p-value
	[25%, 75%]	[25%, 75%]	[25%, 75%]	[25%, 75%]			
IgE	96.35	65.95	46.7	26.2	0.305	0.387	0.124
	[53.62,135.3]	[48.5,96.45]	[20.05,72.4]	[8.85,39.35]			

Transepidermal Water Loss (TEWL)

The analysis of transepidermal water loss (TEWL) from the skin of the mice did not reveal genotype-specific differences. Many male controls and mutant mice showed outlier values for that reason the significances show no evidence for biological relevance.

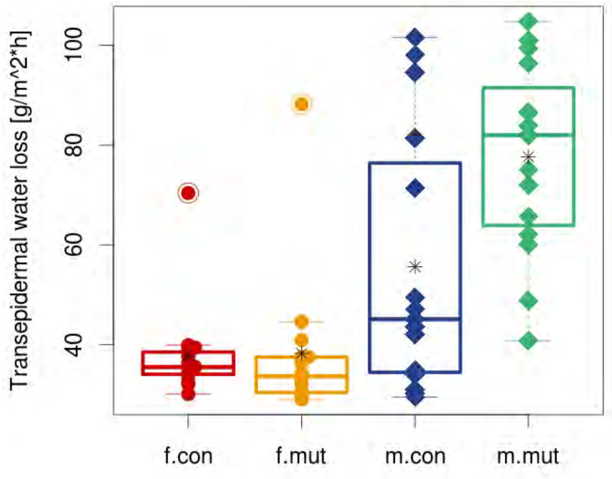

Figure 102
TEWL: Transepidermal water loss boxplot with stripchart, split by sex and genotype
We observed statistically significant differences in the TEWL levels between control and mutant mice due to ouliers and without any relevance.

Table 58
TEWL: Medians, first and third quartile and p-values calculated by a Wilcoxon rank-sum test

	female		male		female	male	overall
	control	mutant	control	mutant			
	15	14	15	15			
	median	median	median	median	p-value	p-value	p-value
	[25%, 75%]	[25%, 75%]	[25%, 75%]	[25%, 75%]			
**TEWL**	35.5	33.65	45.1	82	0.394	0.016	0.15
	[34.05,38.5]	[30.75,37.5]	[34.45,76.4]	[63.9,91.5]			

4.11.3 Discussion

The analysis of basal total IgE levels in plasma revealed values within the range expected for mice on C57BL/6 genetic background.

Steroid Screen 4.12

Summary 4.12.1

Analysis of steroids was not performed for this mouse line.

4.13 Lung Function Screen

4.13.1 Summary

Lung Challenge has been performed and methods and results will be send separately.

4.14 Molecular Phenotyping

4.14.1 Summary

Heart was selected for transcriptome analysis

4.15 Pathology Screen

4.15.1 Summary

Increased body weight in Cldn12 deficient male mice was detected and increased absolute and normalized liver and spleen weights were measured. Histological examination using light microscopy did not reveal any pathological changes that could be attributed to the genotype of the mice.

4.15.2 Results

Macroscopy

Increased body weight in Cldn12 deficient male mice was detected. Increased absolute and normalized liver and spleen weights were measured but no histological changes associated with hepato-splenomegaly were observed. Heart weight of the mutant mice was in the normal range.

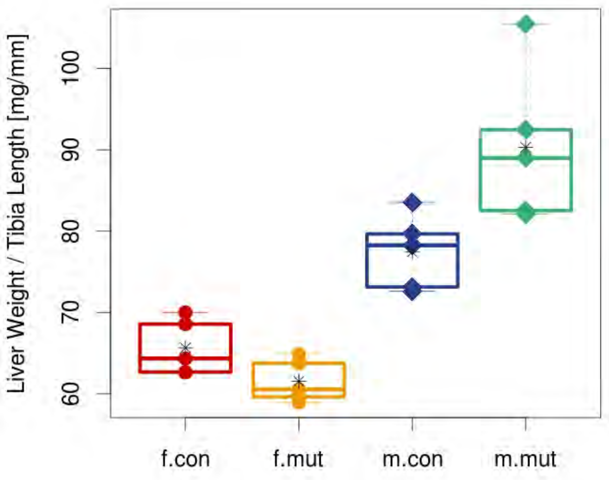

Figure 103
Organ Weight: Liver Weight / Tibia Length boxplot with stripchart, split by sex and genotype

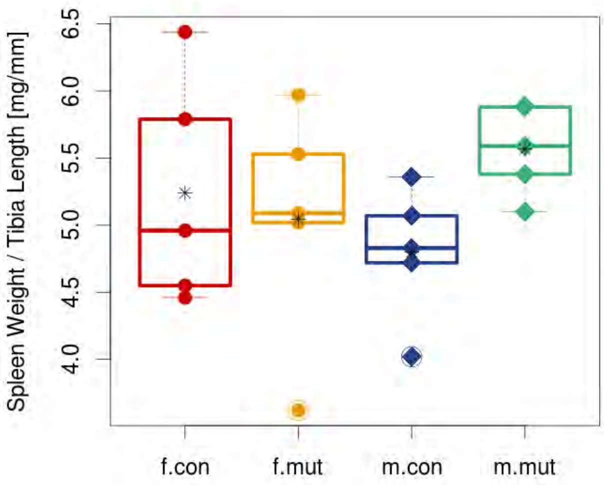

Figure 104
Organ Weight: Spleen Weight / Tibia Length boxplot with stripchart, split by sex and genotype

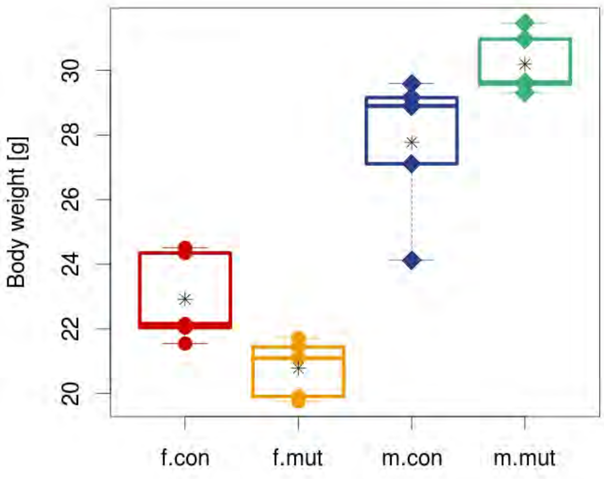

Figure 105
Organ Weight: Body weight boxplot with stripchart, split by sex and genotype

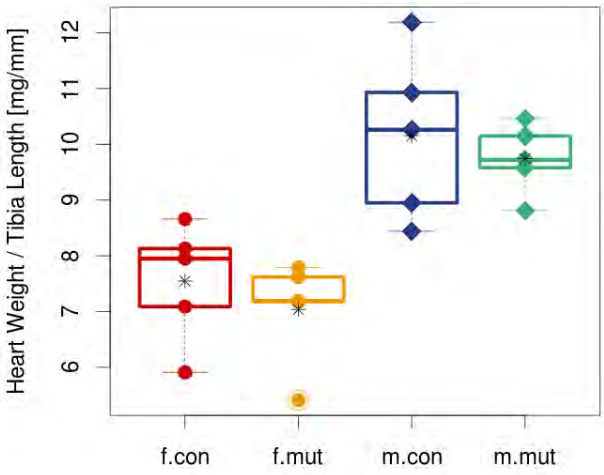

Figure 106
Organ Weight: Heart Weight / Tibia Length boxplot with stripchart, split by sex and genotype

Table 59
Organ Weight: Medians, first and third quartile and p-values calculated by a Wilcoxon rank-sum test
[a] Number not based on the full number of animals (missing values)

	female		male		female	male	overall
	control	mutant	control	mutant			
	n=5	n=5	n=5	n=5			
	median [25%, 75%]	median [25%, 75%]	median [25%, 75%]	median [25%, 75%]	p-value	p-value	p-value
Heart weight [mg]	145 [130 , 146]	131 [130 , 139]	189 [165 , 206]	181 [178 , 190]	0.333	1	0.7
Tibia length [mm]	18.33 [18.23 , 18.47]	18.11 [18.1 , 18.24]	18.44 [18.43 , 18.72]	18.63 [18.59 , 18.72]	0.31	0.738	0.494
Liver weight [g]	1.155 [1.149 , 1.288]	1.095 [1.08 , 1.164]	1.465 [1.348 , 1.501]	1.658 [1.583 , 1.719]	0.222	0.008	0.684
Spleen weight [g]	0.091 [0.083 , 0.107]	0.092 [0.091 , 0.101]	0.089 [0.089 , 0.095]	0.102 [0.1 , 0.11]	1	0.024	0.159
Body weight [g]	22.152 [22.038 , 24.348]	21.096 [19.911 , 21.444]	28.898 [27.109 , 29.156]	29.629 [29.572 , 30.97]	0.016	0.032	0.971
Heart Weight / Tibia Length [mg/mm]	7.95 [7.09 , 8.13]	7.19 [7.18 , 7.62]	10.26 [8.95 , 10.93]	9.72 [9.58 , 10.15]	0.421	0.69	0.631
Heart Weight / Body Weight [mg/g]	6.57 [6.03 , 6.58]	6.06 [6.03 , 6.59]	6.97 [6.84 , 7.07]	6.07 [6.04 , 6.12]	0.802	0.151	0.271
Liver Weight / Tibia Length [mg/mm]	64.35 [62.68 , 68.58]	60.56 [59.64 , 63.78]	78.26 [73.14 , 79.67]	89 [82.53 , 92.47]	0.151	0.032	0.853
Liver Weight / Body Weight [mg/g]	52.56 [52.14 , 53.1]	54 [53.62 , 54.24]	51.48 [50.7 , 51.64]	56.07 [53.43 , 58.64]	0.151	0.222	0.035
Spleen Weight / Tibia Length [mg/mm]	4.96 [4.55 , 5.79]	5.09 [5.02 , 5.53]	4.83 [4.72 , 5.07]	5.59 [5.38 , 5.88]	1	0.016	0.123
Spleen Weight / Body Weight [mg/g]	4.22 [3.77 , 4.39]	4.57 [4.29 , 4.65]	3.29 [3.05 , 3.31]	3.41 [3.29 , 3.5]	0.548	0.341	0.592

Microscopy

According to the information of the collaboration partner, gene expression was observed in muscle, liver, kidney and heart. Therefore these organs were of special interest in this mouse line. The overview below lists all the organs analyzed in the pathology screen, but the histological examination using light microscopy did not reveal any pathological changes that could be attributed to the genotype of the mice. Representative photographs are shown in figure **??**

Table 60
Pathology: Genotype-specific differences - overview

Organ	Phenotype	Organ	Phenotype
Skin	No	Brain	No
Cerebellum	No	Heart	No
Blood vessels	No	Trachea	No
Lung	No	Salivary glands	No
Esophagus	No	Stomach	No
Duodenum	No	Jejunum	No
Colon	No	Rectum	No
Liver	No	Mammary gland	No
Pancreas	No	Lymph nodes	No
Thymus	No	Spleen	No
Thyroid	No	Adrenal gland	No
Kidneys	No	Urinary bladder	No
Testis	No	Epididymis	No
Funiculus spermaticus	No	Prostate	No
Male accessory sex glands	No	Ovaries	No
Uterus	No	Cervix	No
Vagina	No	Body weight	No
Heart weight	No	Liver weight	No
Spleen weight	No		

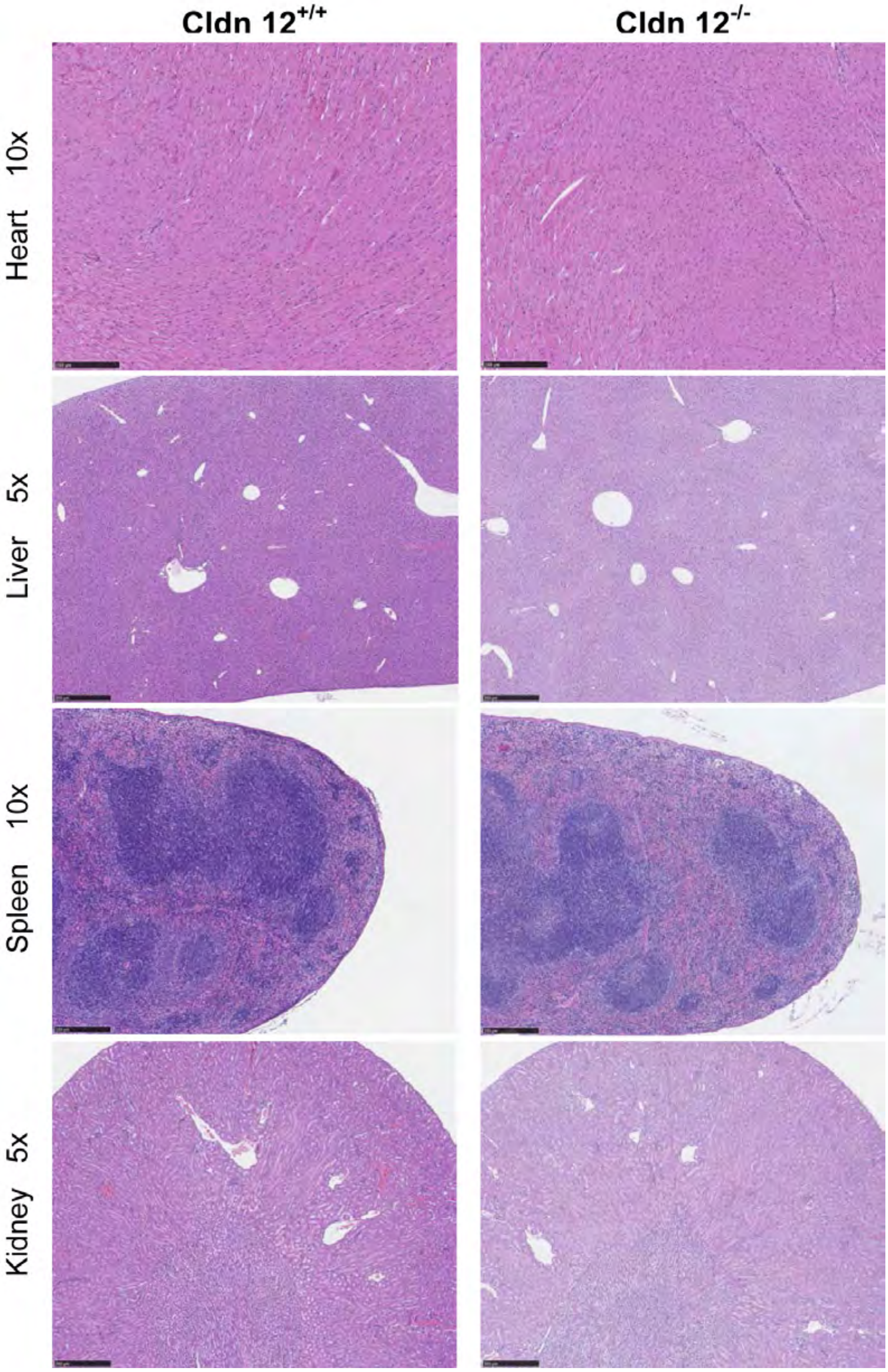

Figure 107

Special Analyses

Additional analysis of the skeletal muscle of hind limbs was done after request from the collaboration partner during results presentation. In FFPE-tissue stained with hematoxylin and eosin, no obvious abnormalities were detected as given in figure 108 .

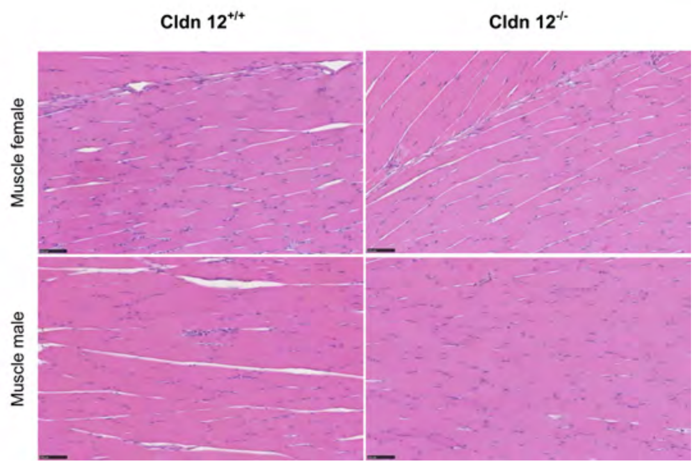

Figure 108

Discussion 4.15.3

Due to the fact that a strong gene expression was observed in muscle and there was decreased locomotor activity by the mutant mice, we performed addditional analysis of the skeletal muscle of hind limbs. The histological examination did not reveal any pathological changes that could be correlated to decreased locomotor activity.

5 Acknowledgements

A large team consisting of scientists, technicians and animal caretakers all contribute to the success of the German Mouse Clinic.

We want to thank Reinhard Seeliger, Anke Bettenbrock, Jan Einicke, Michael Färberböck, Daniel Feeser, Ralf Fischer, Anna Fuchs, Corinne Graf, Brigitte Herrmann, Christine Hollauer, Sebastian Kaidel, Maria Kugler, Sören Kundt, Jacqueline Müller, Philipp Niesl, Anna Nießer, Elenore Samson, Florian Schleicher, Ann-Elisabeth Schwarz, Nadine Senger, Verena Simion, Yvonne Sonntag, Bettina Sperling, Alida Theil, Lucie Thurmann, Susanne Wittich, Julia Wittmann, and Anja Wohlbier for expert technical help as well as Horst Wenig, Manuela Huber, Tina Reichelt, Michael Gerstlauer, Heidi Marr, Annica Miedl, Renate Huber, Richard Klein and Ramona Böswald for the care of the mice.

Addresses of screeners and modules

6

GMC Director

Prof. Dr. Martin Hrabě de Angelis
Institute of Experimental Genetics
Helmholtz Zentrum München
German Research Center for Environmental Health (GmbH)
Ingolstädter Landstraße 1
D-85764 Neuherberg
Tel.: 089-3187-3502
Fax: 089-3187-3500

Management

Dr. Valérie Gailus-Durner (Scientific administrative head)
Dr. Helmut Fuchs (Scientific technical head)
Dr. Claudia Stöger
Dr. Stefanie Leuchtenberger
Theresa Schwarzfischer
Cindy Gonda
Institute of Experimental Genetics
Helmholtz Zentrum München
German Research Center for Environmental Health (GmbH)
Ingolstädter Landstraße 1
D-85764 Neuherberg
Tel.: 089-3187-3613
Fax: 089-3187-3500
E-mail: gailus@helmholtz-muenchen.de

Software Development and Statistics

Dr. Holger Maier
Dr. Ralph Steinkamp
Dr. Anja Hurt
Philipp Gormanns
Elida Schneltzer
Christine Gau
Manuela Gegenfurtner
Institute of Experimental Genetics
Helmholtz Zentrum München
German Research Center for Environmental Health (GmbH)
Ingolstädter Landstraße 1
D-85764 Neuherberg
Tel.: 089-3187-4631
Fax: 089-3187-3500
E-mail: holger.maier@helmholtz-muenchen.de

Data Management and User Support

Dr. Christoph Lengger
Dr. Karlheinz Schäble
Philipp Schmid
Dr. Ralf Schneider
Institute of Experimental Genetics
Helmholtz Zentrum München
German Research Center for Environmental Health (GmbH)
Ingolstädter Landstraße 1
D-85764 Neuherberg
Tel.: 089-3187-3056
Fax: 089-3187-3500
E-mail: lengger@helmholtz-muenchen.de

Behaviour Screen

Dr. Sabine M. Hölter
Dr. Lillian Garrett
Prof. Dr. Wolfgang Wurst
Dr. Annemarie Zimprich
Institute of Developmental Genetics
Helmholtz Zentrum München
German Research Center for Environmental Health (GmbH)
Ingolstädter Landstraße 1
D-85764 Neuherberg
Tel.: 089-3187-3674
Fax: 089-3187-3099
E-mail: hoelter@helmholtz-muenchen.de

Dysmorphology Screen

Dr. Helmut Fuchs
Dr. Wolfgang Hans
Prof. Dr. Martin Hrabě de Angelis
Institute of Experimental Genetics
Helmholtz Zentrum München
German Research Center for Environmental Health (GmbH)
Ingolstädter Landstraße 1
D-85764 Neuherberg
Tel.: 089-3187-3151
Fax: 089-3187-3500
E-mail: hfuchs@helmholtz-muenchen.de

Neurology Screen

Dr. Lore Becker
Dr. Alexandra Vernaleken
Ingrid Liliana Vargas Panesso
German Mouse Clinic (GMC)/Neurology
Institute of Experimental Genetics
Helmholtz Zentrum München
German Research Center for Environmental Health (GmbH)
Ingolstädter Landstraße 1
D-85764 Neuherberg
Tel.: 089-3187-3654
Fax: 089-3187-3500
E-mail: lore.becker@helmholtz-muenchen.de

Prof. Dr. Thomas Klopstock
Friedrich-Baur-Institut,
Neurologische Klinik
Ludwig-Maximilians-Universität München
Ziemssenstraße 1
D-80336 München
Tel: 089-5160-7474
Fax: 089-5160-7402
E-mail: Thomas.Klopstock@med.uni-muenchen.de

Eye Screen

Dr. Oana Veronica Amarie
Prof. Dr. Jochen Graw
Institute of Developmental Genetics
Helmholtz Zentrum München
German Research Center for Environmental Health (GmbH)
Ingolstädter Landstraße 1
D-85764 Neuherberg
Tel.: 089-3187-2910
Fax: 089-3187-2210
E-mail: oliver.puk@helmholtz-muenchen.de

Clinical-Chemical Screen

Dr. Birgit Rathkolb
German Mouse Clinic (GMC)/Clinical-Chemical Screen
Institute of Experimental Genetics
Helmholtz Zentrum München
German Research Center for Environmental Health (GmbH)
Ingolstädter Landstraße 1
D-85764 Neuherberg
Tel.: 089-3187-3654
Fax: 089-3187-3500
E-mail: rathkolb@helmholtz-muenchen.de

Prof. Dr. Eckhard Wolf
Institute of Molecular Animal Breeding
and Biotechnology
Gene Center, LMU München
Feodor Lynen-Strasse 25
D-81377 München
Tel: 089-21807-6800
E-mail: ewolf@lmb.uni-muenchen.de

Immunology Screen

Dr. Thure Adler
Irina Treise
German Mouse Clinic (GMC)/Immunology Screen
Institute of Experimental Genetics
Helmholtz Zentrum München
German Research Center for Environmental Health (GmbH)
Ingolstädter Landstraße 1
D-85764 Neuherberg
Tel.: 089-3187-3283
Fax: 089-3187-3500
E-mail: thure.adler@helmholtz-muenchen.de

Prof. Dr. Dirk H. Busch
Institute for Medical Microbiology,
Immunology and Hygiene
Technische Universität München
Trogerstrasse 30
D-81675 München
Tel: 089-4140-4121
Fax: 089-4140-4868
E-mail: dirk.busch@mikrobio.med.tum.de

Allergy Screen

Dr. Juan Antonio Aguilar Pimentel
German Mouse Clinic (GMC)/Allergy Screen
Institute of Experimental Genetics
Helmholtz Zentrum München
German Research Center for Environmental Health (GmbH)
Ingolstädter Landstraße 1
D-85764 Neuherberg
Tel.: 089-3187-2554
Fax: 089-3187-3500

Prof. Dr. Markus Ollert
Klinik und Poliklinik für Dermatologie
und Allergologie am Biederstein
Technische Universität München (TUM)
Biedersteinerstraße 29
D-80802 München
Tel.: 089-4140-3551
Fax: 089-4140-3552
E-mail: ollert@lrz.tum.de

Steroid Screen

Dr. Cornelia Prehn
Dr. Gabriele Möller
Prof. Dr. Jerzy Adamski
GAC/Institute of Experimental Genetics
Helmholtz Zentrum München
German Research Center for Environmental Health (GmbH)
Ingolstädter Landstraße 1
D-85764 Neuherberg
Tel.: 089-3187-3231
Fax: 089-3187-3500
E-mail: prehn@helmholtz-muenchen.de

Nociceptive Screen

PD PhD Ildikó Rádz
Institute of Molecular Psychiatry
University of Bonn
Sigmund-Freud-Straße 25
D-53127 Bonn
Tel.: 0228-688-5316
Fax: 0228-688-5301
E-mail: iracz@uni-bonn.de

Prof. Dr. Andreas Zimmer
Institute of Molecular Psychiatry
University of Bonn
Sigmund-Freud-Straße 25
D-53105 Bonn
Tel.: 0228-688-5316

Lung Function Screen

Dr. Tobias Stöger
Dr. Ali Önder Yildirim
Prof. Dr. Oliver Eickelberg
Institute of Lung Biology and Disease
Helmholtz Zentrum München
German Research Center for Environmental Health (GmbH)
Ingolstädter Landstraße 1
D-85764 Neuherberg
Tel.: 089-3187-4119
Fax: 089-3187-2400
E-mail: tobias.stoeger@helmholtz-muenchen.de

Molecular Phenotyping

Dr. Marion Horsch
Prof. Dr. Johannes Beckers
Institute of Experimental Genetics
Helmholtz Zentrum München
German Research Center for Environmental Health (GmbH)
Ingolstädter Landstraße 1
D-85764 Neuherberg
Tel.: 089-3187-3513
Fax: 089-3187-3500
E-mail: horsch@helmholtz-muenchen.de

Metabolic Screen

Dr. Jan Rozman
Institute of Experimental Genetics
German Mouse Clinic (GMC)/Metabolic Screen
Helmholtz Zentrum München
German Research Center for Environmental Health (GmbH)
Ingolstädter Landstraße 1
D-85764 Neuherberg
Tel.: 089-3187-3807
Fax: 089-3187-3500
E-mail: jan.rozman@helmholtz-muenchen.de

Prof. Dr. Martin Klingenspor
Molecular Nutritional Medicine,
Else Kröner-Fresenius Center
Technische Universität München
Am Forum 5
D-85350 Freising-Weihenstephan

Cardiovascular Screen

Dr. Kristin Moreth
Institute of Experimental Genetics
GMC - German Mouse Clinic
Cardiovascular Screen
Helmholtz Zentrum München
German Research Center for Environmental Health (GmbH)
Ingolstädter Landstraße 1
D-85764 Neuherberg
Tel.: 089-3187-3646
Fax: 089-3187-3500
E-mail: kristin.moreth@helmholtz-muenchen.de

Prof. Dr. Raffi Bekeredjian
Prof. Dr. Hugo Katus
Innere Medizin III
Kardiologie
Otto-Meyerhof-Zentrum
Im Neuenheimer Feld 350
D-69120 Heidelberg
Tel.: 06221-56-1505
E-mail: raffi.bekeredjian@med.uni-heidelberg.de

Pathology Screen

Dr. Frauke Neff
Dr. Julia Calzada-Wack
Dr. Dirk Janik
Dr. Tanja Klein-Rodewald
Dr. Laura Brachthäuser Institute of Pathology
Helmholtz Zentrum München
German Research Center for Environmental Health (GmbH)
Ingolstädter Landstraße 1
D-85764 Neuherberg
Tel.: 089-3187-3241
Fax: 089-3187-3360
E-mail: frauke.neff@helmholtz-muenchen.de

Prof. Dr. med. Heinz Höfler
Institut für Allgemeine Pathologie und Pathologische Anatomie
Technische Universität München
Trogerstr. 18
D-81675 München

References

Abe, K., Fuchs, H., Lisse, T., Hans, W., and Hrabě de Angelis, M. (2006). New ENU-induced semidominant mutation, Ali18, causes inflammatory arthritis, dermatitis, and osteoporosis in the mouse. *Mamm Genome*, 17(9):915–26.

Aguilar-Pimentel, J. A., Alessandrini, F., Huster, K. M., Jakob, T., Schulz, H., Behrendt, H., Ring, J., Hrabě de Angelis, M., Busch, D. H., Mempel, M., and Ollert, M. (2010). Specific CD8 T cells in IgE-mediated allergy correlate with allergen dose and allergic phenotype. *Am J Respir Crit Care Med*, 1;181(1):7–16.

Alberts, P., Ronquist-Nii, Y., Larsson, C., Klingstrom, G., Engblom, L., Edling, N., Lidell, V., Berg, I., Edlund, P. O., Ashkzari, M., Sahaf, N., Norling, S., Berggren, V., Bergdahl, K., Forsgren, M., and Abrahmsen, L. (2005). Effect of high-fat diet on KKAy and ob/ob mouse liver and adipose tissue corticosterone and 11-dehydrocorticosterone concentrations. *Horm Metab Res*, 37(7):402–7.

Alessandrini, F., Jakob, T., Wolf, A., Wolf, E., Balling, R., Hrabě de Angelis, M., Ring, J., and Behrendt, H. (2001). ENU mouse mutagenesis: generation of mouse mutants with aberrant plasma IgE levels. *Int Arch Allergy Immunol*, 124(1-3):25–8.

Alessandrini, F., Schulz, H., Takenaka, S., Lentner, B., Karg, E., Behrendt, H., and Jakob, T. (2006). Effects of ultrafine carbon particle inhalation on allergic inflammation of the lung. *J Allergy Clin Immunol*, 117(4):824–30.

Andersson, S., Berman, D. M., Jenkins, E. P., and Russell, D. W. (1991). Deletion of steroid 5 alpha-reductase 2 gene in male pseudohermaphroditism. *Nature*, 354(6349):159–61.

Archer, J. (1973). Tests for emotionality in rats and mice: a review. *Anim Behav*, 21(2):205–35.

Arruda, L. K., Sole, D., Baena-Cagnani, C. E., and Naspitz, C. K. (2005). Risk factors for asthma and atopy. *Curr Opin Allergy Clin Immunol*, 5(2):153–9.

Atanasov, A. G. and Odermatt, A. (2007). Readjusting the glucocorticoid balance: an opportunity for modulators of 11beta-hydroxysteroid dehydrogenase type 1 activity? *Endocr Metab Immune Disord Drug Targets*, 7(2):125–40.

Bachman, E. S., Hampton, T. G., Dhillon, H., Amende, I., Wang, J., Morgan, J. P., and Hollenberg, A. N. (2004). The metabolic and cardiovascular effects of hyperthyroidism are largely independant of beta-adrenergic stimulation. 145(6):2767 – 74.

Bansal, N., Houle, A., and Melnykovych, G. (1991). Apoptosis: mode of cell death induced in T cell leukemia lines by dexamethasone and other agents. *FASEB J*, 5(2):211–6.

Bartolomucci, A., Cabassi, A., Govoni, P., Ceresini, G., Cero, C., Berra, D., Dadomo, H., Franceschini, P., Dell'Omo, G., Parmigiani, S., and Palanza, P. (2009). Metabolic consequences and vulnerability to diet-induced obesity in male mice under chronic social stress. *PLoS One*, 4(1):e4331.

Baumgarth, N. and Roederer, M. (2000). A practical approach to multicolor flow cytometry for immunophenotyping. *J Immunol Methods*, 243(1-2):77–97.

Bluethmann H., O. P., ed (1994). Transgenesis and targeted mutagenesis in immunology, Academic Press, San Diego.

Bochner, B. S. and Busse, W. W. (2005). Allergy and asthma. *J Allergy Clin Immunol*, 115(5):953–9.

Bolivar, V. J., Caldarone, B. J., Reilly, A. A., and Flaherty, L. (2000). Habituation of activity in an open field: A survey of inbred strains and F1 hybrids. *Behav Genet*, 30(4):285–93.

Brayton, C., Justice, M., and Montgomery, C. A. (2001). Evaluating mutant mice: anatomic pathology. *Vet Pathol*, 38(1):1–19.

Brielmeier, M., Fuchs, H., Przemeck, G., Gailus-Durner, V., Hrabě de Angelis, M., and Schmidt, J. (2002). The GSF-Phenotype Analysis Center (German Mouse Clinic, GMC): a sentinel based health monitoring concept in a multi-user unit for standardized characterization of mouse mutants. In Guenet, J.-L. and Herweg, C., eds, *Laboratory Animals Science - Basis and Strategy for Animal Experimentation*, volume 11 of *Proceedings of the 8th Symposium of the Federation of European Laboratory Animal Science Associations (FELASA)*, pages 19–22, Laboratory Animals Ltd, Aachen.

Brown, S. D., Chambon, P., and Hrabě de Angelis, M. (2005). EMPReSS: standardized phenotype screens for functional annotation of the mouse genome. *Nat Genet*, 37(11):1155.

Bucan, M. and Abel, T. (2002). The mouse: genetics meets behaviour. *Nat Rev Genet*, 3(2):114–23.

Burkard, R. F., Don, M., and Eggermont, J. J. (2007). Auditory Evoked Potentials, Lippincott Williams and Wilkins, Philadelphia, PA 19103, USA.

Calvano, S. E., Xiao, W., Richards, D. R., Felciano, R. M., Baker, H. V., Cho, R. J., Chen, R. O., Brownstein, B. H., Cobb, J. P., Tschoeke, S. K., Miller-Graziano, C., Moldawer, L. L., Mindrinos, M. N., Davis, R. W., Tompkins, R. G., and Lowry, S. F. (2005). A network-based analysis of systemic inflammation in humans. *Nature*, 437(7061):1032–7.

Carlyle, J. R., Mesci, A., Ljutic, B., Belanger, S., Tai, L. H., Rousselle, E., Troke, A. D., Proteau, M. F., and Makrigiannis, A. P. (2006). Molecular and genetic basis for strain-dependent NK1.1 alloreactivity of mouse NK cells. *J Immunol*, 176(12):7511–24.

Champy, M. F., Selloum, M., Piard, L., Zeitler, V., Caradec, C., Chambon, P., and Auwerx, J. (2004). Mouse functional genomics requires standardization of mouse handling and housing conditions. *Mamm Genome*, 15(10):768–83.

Chen, J. and Harrison, D. E. (2002). Quantitative trait loci regulating relative lymphocyte proportions in mouse peripheral blood. *Blood*, 99(2):561–6.

Chipman, S. D., Sweet, H. O., McBride, D. J., J., Davisson, M. T., Marks, S. C., J., Shuldiner, A. R., Wenstrup, R. J., Rowe, D. W., and Shapiro, J. R. (1993). Defective pro alpha 2(I) collagen synthesis in a recessive mutation in mice: a model of human osteogenesis imperfecta. *Proc Natl Acad Sci U S A*, 90(5):1701–5.

Choleris, E., Thomas, A. W., Kavaliers, M., and Prato, F. S. (2001). A detailed ethological analysis of the mouse open field test: effects of diazepam, chlordiazepoxide and an extremely low frequency pulsed magnetic field. *Neurosci Biobehav Rev*, 25(3):235–60.

Chowen, J. A., Azcoitia, I., Cardona-Gomez, G. P., and Garcia-Segura, L. M. (2000). Sex steroids and the brain: lessons from animal studies. *J Pediatr Endocrinol Metab*, 13(8):1045–66.

Collins, K. A., Korcarz, C. E., and Lang, R. M. (2003). Use of echocardiography for the phenotypic assessment of genetically altered mice. *Physiol Genomics*, 13(3):227–39.

Corteling, R. and Trifilieff, A. (2004). Gender comparison in a murine model of allergen-driven airway inflammation and the response to budesonide treatment. *BMC Pharmacol*, 4(4).

Covelli, V. (2012). Vincenzo Covelli - Guide to the necropsy of the mouse. Electronic. http://eulep.pdn.cam.ac.uk/Necropsy_of_the_Mouse/index.php.

Crawley, J. (1989). Animal models of anxiety. *Current Opinion in Psychiatry*, 2:773–776.

Dalke, C. and Graw, J. (2005). Mouse mutants as models for congenital retinal disorders. *Exp Eye Res*, 81(5):503–12.

Dalke, C., Löster, J., Fuchs, H., Gailus-Durner, V., Soewarto, D., Favor, J., Neuhauser-Klaus, A., Pretsch, W., Gekeler, F., Shinoda, K., Zrenner, E., Meitinger, T., Hrabě de Angelis, M., and Graw, J. (2004). Electroretinography as a screening method for mutations causing retinal dysfunction in mice. *Invest Ophthalmol Vis Sci*, 45(2):601–9.

Dennis, G., J., Sherman, B. T., Hosack, D. A., Yang, J., Gao, W., Lane, H. C., and Lempicki, R. A. (2003). David: Database for annotation, visualization, and integrated discovery. *Genome Biol*, 4(5):P3.

Deschepper, C. F., Olson, J. L., Otis, M., and Gallo-Payet, N. (2004). Characterization of blood pressure and morphological traits in cardiovascular-related organs in 13 different inbred mouse strains. *J Appl Physiol*, 97(1):369–76.

Diaz, D., Prieto, A., Barcenilla, H., Monserrat, J., Prieto, P., Sanchez, M. A., Reyes, E., Hernandez-Fuentes, M. P., de la Hera, A., Orfao, A., and Alvarez-Mon, M. (2004). Loss of lineage antigens is a common feature of apoptotic lymphocytes. *J Leukoc Biol*, 76(3):609–15.

Doevendans, P. A., Daemen, M. J., de Muinck, E. D., and Smits, J. F. (1998). Cardiovascular phenotyping in mice. *Cardiovasc Res*, 39(1):34–49.

Drorbaugh, J. E. and Fenn, W. O. (1955). A barometric method for measuring ventilation in newborn infants. *Pediatrics*, 16(1):81–7.

Drozdz, A. (1975). Food habits and food assimilation in mammals., In: Methods for Ecological Bioenergetics (Grodzinski, W., Klekowski, R., and Duncan, A., eds), Oxford, UK: Blackwell, 23–47.

Duffy, P. H., Feuers, R. J., and Hart, R. W. (1990). Effect of chronic caloric restriction on the circadian regulation of physiological and behavioral variables in old male B6C3F1 mice. *Chronobiol Int*, 7(4):291–303.

Eddy, N. B. and Leimbach, D. (1953). Synthetic analgesics. II. dithienylbutenyl- and dithienylbutylamines. *J Pharmacol Exp Ther*, 107(3):385–93.

Ehmke, H. (2003). Mouse gene targeting in cardiovascular physiology. *Am J Physiol Regul Integr Comp Physiol*, 284(1):R28–30.

Enard, W., Gehre, S., Hammerschmidt, K., Hölter, S. M., Blass, T., Somel, M., Bruckner, M. K., Schreiweis, C., Winter, C., Sohr, R., Becker, L., Wiebe, V., Nickel, B., Giger, T., Müller, U., Groszer, M., Adler, T., Aguilar, A., Bolle, I., Calzada-Wack, J., Dalke, C., Ehrhardt, N., Favor, J., Fuchs, H., Gailus-Durner, V., Hans, W., Holzlwimmer, G., Javaheri, A., Kalaydjiev, S., Kallnik, M., Kling, E., Kunder, S., Mossbrugger, I., Naton, B., Raćz, I., Rathkolb, B., Rozman, J., Schrewe, A., Busch, D. H., Graw, J., Ivandic, B., Klingenspor, M., Klopstock, T., Ollert, M., Quintanilla-Martinez, L., Schulz, H., Wolf, E., Wurst, W., Zimmer, A., Fisher, S. E., Morgenstern, R., Arendt, T., Hrabě de Angelis, M., Fischer, J., Schwarz, J., and Pääbo, S. (2009). A humanized version of Foxp2 affects cortico-basal ganglia circuits in mice. *Cell*, 137(5):961–71.

Erben, R. G., Soegiarto, D. W., Weber, K., Zeitz, U., Lieberherr, M., Gniadecki, R., Möller, G., Adamski, J., and Balling, R. (2002). Deletion of deoxyribonucleic acid binding domain of the Vitamin D receptor abrogates genomic and nongenomic functions of Vitamin D. *Mol Endocrinol*, 16(7):1524–37.

Exner, C., Hebebrand, J., Remschmidt, H., Wewetzer, C., Ziegler, A., Herpertz, S., Schweiger, U., Blum, W. F., Preibisch, G., Heldmaier, G., and Klingenspor, M. (2000). Leptin suppresses semi-starvation induced hyperactivity in rats: implications for anorexia nervosa. *Mol Psychiatry*, 5(5):476–81.

Favor, J. (1983). A comparison of the dominant cataract and recessive specific-locus mutation rates induced by treatment of male mice with ethylnitrosourea. *Mutat Res*, 110(2):367–82.

Fischer, A. (2002). Natural mutants of the immune system: a lot to learn! *Eur J Immunol*, 32(6):1519–1523.

Fischer, M. D., Huber, G., Beck, S. C., Tanimoto, N., Muehlfriedel, R., Fahl, E., Grimm, C., Wenzel, A., Reme, C. E., van de Pavert, S. A., Wijnholds, J., Pacal, M., Bremner, R., and Seeliger, M. W. (2009). Noninvasive, in vivo assessment of mouse retinal structure using optical coherence tomography. *PLoS ONE,*, 4(10):7507.

Fluhr, J. W., Feingold, K. R., and Elias, P. M. (2006). Transepidermal water loss reflects permeability barrier status: validation in human and rodent in vivo and ex vivo models. *Exp. Dermatol.*, 15(70):483 – 492.

Fougerousse, F., Bullen, P., Herasse, M., Lindsay, S., Richard, I., Wilson, D., Suel, L., Durand, M., Robson, S., Abitbol, M., Beckmann, J. S., and Strachan, T. (2000). Human-mouse differences in the embryonic expression patterns of developmental control genes and disease genes. *Hum Mol Genet*, 9(2):165–73.

Freitas, A. A. and Rocha, B. (2000). Population biology of lymphocytes: the flight for survival. *Annu Rev Immunol*, 18:83–111.

Fuchs, H., Gailus-Durner, V., Adler, T., Pimentel, J. A., Becker, L., Bolle, I., Brielmeier, M., Calzada-Wack, J., Dalke, C., Ehrhardt, N., Fasnacht, N., Ferwagner, B., Frischmann, U., Hans, W., Hölter, S. M., Hölzlwimmer, G., Horsch, M., Javaheri, A., Kallnik, M., Kling, E., Lengger, C., Maier, H., Mossbrugger, I., Mörth, C., Naton, B., Nöth, U., Pasche, B., Prehn, C., Przemeck, G., Puk, O., Raćz, I., Rathkolb, B., Rozman, J., Schäble, K., Schreiner, R., Schrewe, A., Sina, C., Steinkamp, R., Thiele, F., Willershäuser, M., Zeh, R., Adamski, J., Busch, D. H., Beckers, J., Behrendt, H., Daniel, H., Esposito, I., Favor, J., Graw, J., Heldmaier, G., Höfler, H., Ivandic, B., Katus, H., Klingenspor, M., Klopstock, T., Lengeling, A., Mempel, M., Müller, W., Neschen, S., Ollert, M., Quintanilla-Martinez, L., Rosenstiel, P., Schmidt, J., Schreiber, S., Schughart, K., Schulz, H., Wolf, E., Wurst, W., Zimmer, A., and Hrabě de Angelis, M. (2009). The German Mouse Clinic: a platform for systemic phenotype analysis of mouse models. *Curr Pharm Biotechnol*, 10(2):236–43.

Fuchs, H., Lisse, T., Abe, K., and Hrabě de Angelis, M. (2006). Screening for bone and cartilage phenotypes in mice., In: Phenotyping of the Laboratory Mouse (Hrabě de Angelis, M., Chambon, P., and Brown, S., eds), Wiley-VCH, Weinheim, 35-86.

Fuchs, H., Schughart, K., Wolf, E., Balling, R., and Hrabě de Angelis, M. (2000). Screening for dysmorphological abnormalities–a powerful tool to isolate new mouse mutants. *Mamm Genome*, 11(7):528–30.

Fulton, R. J., McDade, R. L., Smith, P. L., Kienker, L. J., and Kettman, J. R. (1997). Advanced multiplexed analysis with the flowmetrix system. *Clin Chem*, 43(9):1749–1756.

Gailus-Durner, V., Fuchs, H., Adler, T., Aguilar Pimentel, A., Becker, L., Bolle, I., Calzada-Wack, J., Dalke, C., Ehrhardt, N., Ferwagner, B., Hans, W., Hölter, S., Hölzlwimmer, G., Horsch, M., Javaheri, A., Kallnik, M., Kling, E., Lengger, C., Mörth, C., Mossbrugger, I., Naton, B., Prehn, C., Puk, O., Rathkolb, B., Rozman, J., Schrewe, A., Thiele, F., Adamski, J., Aigner, B., Behrendt, H., Busch, D., Favor, J., Graw, J., Heldmaier, G., Ivandic, B., Katus, H., Klingenspor, M., Klopstock, T., Kremmer, E., Ollert, M., Quintanilla-Martinez, L., Schulz, H., Wolf, E., Wurst, W., and Hrabě de Angelis, M. (2009). Systemic First-Line Phenotyping, In: Methods in Molecular Biology volume 530, Gene Knockout Protocols, Second Edition, (Kühn, R. and Wurst, W., eds), Humana Press, 463-509.

Gailus-Durner, V., Fuchs, H., Becker, L., Bolle, I., Brielmeier, M., Calzada-Wack, J., Elvert, R., Ehrhardt, N., Dalke, C., Franz, T. J., Grundner-Culemann, E., Hammelbacher, S., Hölter, S. M., Hölzlwimmer, G., Horsch, M., Javaheri, A., Kalaydjiev, S. V., Klempt, M., Kling, E., Kunder, S., Lengger, C., Lisse, T., Mijalski, T., Naton, B., Pedersen, V., Prehn, C., Przemeck, G., Raćz, I., Reinhard, C., Reitmeir, P., Schneider, I., Schrewe, A., Steinkamp, R., Zybill, C., Adamski, J., Beckers, J., Behrendt, H., Favor, J., Graw, J., Heldmaier, G., Höfler, H., Ivandic, B., Katus, H., Kirchhof, P., Klingenspor, M., Klopstock, T., Lengeling, A., Müller, W., Ohl, F., Ollert, M., Quintanilla-Martinez, L., Schmidt, J., Schulz, H., Wolf, E., Wurst, W., Zimmer, A., Busch, D. H., and Hrabě de Angelis, M. (2005). Introducing the German Mouse Clinic: open access platform for standardized phenotyping. *Nat Methods*, 2(6):403–4.

Ganguly, K., Depner, M., Fattman, C., Bein, K., Oury, T. D., Wesselkamper, S. C., Borchers, M. T., Schreiber, M., Gao, F., von Mutius, E., Kabesch, M., Leikauf, G. D., and Schulz, H. (2009). Superoxide dismutase 3, extracellular (SOD3) variants and lung function. *Physiol Genomics*, 37(3):260–7.

Ganguly, K. and Schulz, H. (2008). Association studies of lung function in mice. *Dtsch Tierarztl Wochenschr*, 115(7):276–84.

Ganguly, K., Stoeger, T., Wesselkamper, S. C., Reinhard, C., Sartor, M. A., Medvedovic, M., Tomlinson, C. R., Bolle, I., Mason, J. M., Leikauf, G. D., and Schulz, H. (2007). Candidate genes controlling pulmonary function in mice: transcript profiling and predicted protein structure. *Physiol Genomics*, 31(3):410–21.

Gasser, J. A. (2003). Bone measurements by peripheral quantitative computed tomography in rodents. *Methods Mol Med*, 80:323–41.

Geyer, M. A., Krebs-Thomson, K., Braff, D. L., and Swerdlow, N. R. (2001). Pharmacological studies of prepulse inhibition models of sensorimotor gating deficits in schizophrenia: a decade in review. *Psychopharmacology (Berl)*, 156(2-3):117–54.

Ghadially, R., Brown, B. E., Sequeira-Martin, S. M., Feingold, K. R., and Elias, P. M. (1995). The aged epidermal permeability barrier. Structural, functional, and lipid biochemical abnormalities in humans and a senescent murine model. *J. Clin. Invest.*, 95(5):2281–2290.

Giampietro, P. F., Blank, R. D., Raggio, C. L., Merchant, S., Jacobsen, F. S., Faciszewski, T., Shukla, S. K., Greenlee, A. R., Reynolds, C., and Schowalter, D. B. (2003). Congenital and idiopathic scoliosis: clinical and genetic aspects. *Clin Med Res*, 1(2):125–36.

Gibson, G. (2010). Hints of hidden heritability in GWAS. *Nat Genet*, 42(7):558–560.

Gottesman, I. and Gould, T. D. (2003). The endophenotype concept in psychiatry: etymology and strategic intentions. *Am J Psychiatry*, 160(4):636–45.

Graw, J. (2003). The genetic and molecular basis of congenital eye defects. *Nat Rev Genet*, 4(11):876–88.

Grewal, I. S., Heilig, M., Miller, A., and Sercarz, E. E. (1997). Environmental regulation of T-cell function in mice: group housing of males affects accessory cell function. *Immunology*, 90(2):165–8.

Haegel, H. and Ceredig, R. (1991). Transcripts encoding mouse CD44 (Pgp-1, Ly-24) antigen: strain variation and induction by mitogen. *Eur J Immunol*, 21(6):1549–53.

Hafezparast, M., Ahmad-Annuar, A., Wood, N. W., Tabrizi, S. J., and Fisher, E. M. (2002). Mouse models for neurological disease. *Lancet Neurol*, 1(4):215–24.

Hall, M. A., Ahmadi, K. R., Norman, P., Snieder, H., MacGregor, A. J., Vaughan, R. W., Spector, T. D., and Lanchbury, J. S. (2000). Genetic influence on peripheral blood T lymphocyte levels. *Genes Immun*, 1(7):423–427.

Haller, F., Prehn, C., and Adamski, J. (2010). Quantification of steroids in human and mouse plasma using online solid phase extraction coupled to liquid chromatography tandem mass spectrometry. Electronic. 10.1038/nprot.2010.22.

Hebebrand, J., Exner, C., Hebebrand, K., Holtkamp, C., Casper, R. C., Remschmidt, H., Herpertz-Dahlmann, B., and Klingenspor, M. (2003). Hyperactivity in patients with anorexia nervosa and in semistarved rats: evidence for a pivotal role of hypoleptinemia. *Physiol Behav*, 79(1):25–37.

Hector, A., Kormann, M. S. D., Mack, I., Latzin, P., Casaulta, C., Kieninger, E., Zhou, Z., Yildirim, A. O., Bohla, A., Rieber, N., Kappler, M., Koller, B., Eber, E., Eickmeier, O., Zielen, S., Eickelberg, O., Griese, M., Mall, M. A., and Hartl, D. (2011). The chitinase-like protein YKL-40 modulates cystic fibrosis lung disease. *PLoS One*, 6(9):e24399.

Hegde, P., Qi, R., Abernathy, K., Gay, C., Dharap, S., Gaspard, R., Hughes, J. E., Snesrud, E., Lee, N., and Quackenbush, J. (2000). A concise guide to cDNA microarray analysis. *Biotechniques*, 29(3):548–562.

Hermiston, M. L., Xu, Z., and Weiss, A. (2003). CD45: a critical regulator of signaling thresholds in immune cells. *Annu Rev Immunol*, 21:107–37.

Horsch, M., Schädler, S., Gailus-Durner, V., Fuchs, H., Meyer, H., Hrabě de Angelis, M., and Beckers, J. (2008). Systematic gene expression profiling of mouse model series reveals coexpressed genes. *Proteomics*, 8(6):1248–56.

Hough, T. A., Nolan, P. M., Tsipouri, V., Toye, A. A., Gray, I. C., Goldsworthy, M., Moir, L., Cox, R. D., Clements, S., Glenister, P. H., Wood, J., Selley, R. L., Strivens, M. A., Vizor, L., McCormack, S. L., Peters, J., Fisher, E. M., Spurr, N., Rastan, S., Martin, J. E., Brown, S. D. M., and Hunter, A. J. (2002). Novel phenotypes identified by plasma biochemical screening in the mouse. *Mamm Genome*, 13(10):595–602.

Hrabě de Angelis, M. H., Flaswinkel, H., Fuchs, H., Rathkolb, B., Soewarto, D., Marschall, S., Heffner, S., Pargent, W., Wuensch, K., Jung, M., Reis, A., Richter, T., Alessandrini, F., Jakob, T., Fuchs, E., Kolb, H., Kremmer, E., Schäble, K., Rollinski, B., Roscher, A., Peters, C., Meitinger, T., Strom, T., Steckler, T., Holsboer, F., Klopstock, T., Gekeler, F., Schindewolf, C., Jung, T., Avraham, K.,

Behrendt, H., Ring, J., Zimmer, A., Schughart, K., Pfeffer, K., Wolf, E., and Balling, R. (2000). Genome-wide, large-scale production of mutant mice by ENU mutagenesis. *Nat Genet*, 25(4):444–7.

Ingham, N. J., Pearson, S., and Steel, K. P. (2011). Using the Auditory Brainstem Response (ABR) to determine sensitivity of hearing in mutant mice. *Current Protocols in Mouse Biology*, 1:279–287.

Inoue, K. and Lupski, J. R. (2003). Genetics and genomics of behavioral and psychiatric disorders. *Curr Opin Genet Dev*, 13(3):303–9.

Irwin, S. (1968). Comprehensive observational assessment: Ia. A systematic, quantitative procedure for assessing the behavioral and physiologic state of the mouse. *Psychopharmacologia*, 13(3):222–57.

Iwakawa, M., Noda, S., Ohta, T., Ohira, C., Lee, R., Goto, M., Wakabayashi, M., Matsui, Y., Harada, Y., and Imai, T. (2003). Different radiation susceptibility among five strains of mice detected by a skin reaction. *J. Radiat. Res.*, 44(1):7–13.

Jakob, T., Köllisch, G. V., Howaldt, M., Bewersdorff, M., Rathkolb, B., Müller, M. L., Sandholzer, N., Nitschke, L., Schiemann, M., Mempel, M., Ollert, M., Neubauer, A., Soewarto, D. A., Kremmer, E., Ring, J., Behrendt, H., and Flaswinkel, H. (2008). Novel mouse mutants with primary cellular immunodeficiencies generated by genome-wide mutagenesis. *J Allergy Clin Immunol*, 121(1):179–184.

Janeway, C., Travers, P., Walport, M., Shlomchik, M., and Shlomchik, M. (2004). Immunobiology: The Immune System in Health and Disease. 6th Edition, Garland Publishing, London.

Jerome, C. P. (2004). Hormonal therapies and osteoporosis. *ILAR J*, 45(2):170–8.

Johnson, G. and Foster, A. (2003). Prevalence, incidence and distribution of visual impairment., In: The epidemiology of the eye disease (Johnson, G., Minassian, D., Weale, R., and West, S., eds), Arnold, London, UK, 3-28.

Jones, B. and Roberts, D. (1968). The quantitative measurement of motor incoordination in naive mice using an accelerating Rota-Rod. *J Pharm Pharmacol*, 20:302–304.

Kabesch, M. (2006). Gene by environment interactions and the development of asthma and allergy. *Toxicol Lett*, 162(1):43–8.

Katz, D. M. (2003). Neuronal growth factors and development of respiratory control. *Respir Physiol Neurobiol*, 135(2-3):155–65.

Kettunen, R. V., Leppaluoto, J., Jounela, A., and Vuolteenaho, O. (1994). Plasma n-terminal atrial natriuretic peptide in acute myocardial infarction. *Am Heart J*, 127(6):1449–55.

Kilic, A., Bubikat, A., Gassner, B., Baba, H. A., and Kuhn, M. (2007). Local actions of atrial natriuretic peptide counteract angiotensin II stimulated cardiac remodeling. *Endocrinology*, 148(9):4162–9.

Kim, D., Carlson, J. N., Seegal, R. F., and Lawrence, D. A. (1999). Differential immune responses in mice with left- and right-turning preference. *J Neuroimmunol*, 93(1-2):164–171.

Kimura, M., Tanaka, S., Yamada, Y., Kiuchi, Y., Yamakawa, T., and Sekihara, H. (1998). Dehydroepiandrosterone decreases serum tumor necrosis factor-alpha and restores insulin sensitivity: independent effect from secondary weight reduction in genetically obese Zucker fatty rats. *Endocrinology*, 139(7):3249–53.

Kneidinger, N., Yildirim, A. O., Callegari, J., Takenaka, S., Stein, M. M., Dumitrascu, R., Bohla, A., Bracke, K. R., Morty, R. E., Brusselle, G. G., Schermuly, R. T., Eickelberg, O., and Königshoff, M. (2011). Activation of the WNT/beta-catenin pathway attenuates experimental emphysema. *Am J Respir Crit Care Med*, 183(6):723–733.

Krege, J. H., Hodgin, J. B., Hagaman, J. R., and Smithies, O. (1995). A noninvasive computerized tail-cuff system for measuring blood pressure in mice. *Hypertension*, 25(5):1111–5.

Krzych, U., Strausser, H. R., Bressler, J. P., and Goldstein, A. L. (1978). Quantitative differences in immune responses during the various stages of the estrous cycle in female BALB/c mice. *J Immunol*, 121(4):1603–1605.

Labrie, F., Belanger, A., Simard, J., Van, L.-T., and Labrie, C. (1995). DHEA and peripheral androgen and estrogen formation: intracinology. *Ann N Y Acad Sci*, 774:16–28.

Lang, R. E., Unger, T., and Ganten, D. (1987). Atrial natriuretic peptide: a new factor in blood pressure control. *J Hypertens*, 5(3):255–71.

Lee, A. J., Hodges, S., and Eastell, R. (2000). Measurement of osteocalcin. *Ann Clin Biochem*, 37 (Pt 4):432–46.

Lerman, A., Gibbons, R. J., Rodeheffer, R. J., Bailey, K. R., McKinley, L. J., Heublein, D. M., and Burnett, J. C. (1993). Circulating n-terminal atrial natriuretic peptide as a marker for symptomless left-ventricular dysfunction. *Lancet*, 341(8853):1105–1109.

Lorenz, J. N. (2002). A practical guide to evaluating cardiovascular, renal, and pulmonary function in mice. *Am J Physiol Regul Integr Comp Physiol*, 282(6):R1565–82.

Maier, H., Lengger, C., Simic, B., Fuchs, H., Gailus-Durner, V., and Hrabě de Angelis, M. (2008). MausDB: an open source application for phenotype data and mouse colony management in large-scale mouse phenotyping projects. *BMC Bioinformatics*, 9:169.

Mak, T. W., Penninger, J. M., and Ohashi, P. S. (2001). Knockout mice: a paradigm shift in modern immunology. *Nat Rev Immunol*, 1(1):11–19.

Mallon, A. M., Blake, A., and Hancock, J. M. (2008). EuroPhenome and EMPReSS: online mouse phenotyping resource. *Nucleic Acids Res*, 36(Database issue):D715–8.

Mariani, F. V. and Martin, G. R. (2003). Deciphering skeletal patterning: clues from the limb. *Nature*, 423(6937):319–25.

Mayer, L. P., Devine, P. J., Dyer, C. A., and Hoyer, P. B. (2004). The follicle-deplete mouse ovary produces androgen. *Biol Reprod*, 71(1):130–8.

McLean, W. and Olsen, B. R. (2001). Mouse models of abnormal skeletal development and homeostasis. *Trends Genet*, 17(10):S38–43.

Mead, J. (1963). The control of respiratory frequency. *Ann N Y Acad Sci*, 109:724–9.

Medina, E., Goldmann, O., Rohde, M., Lengeling, A., and Chhatwals, G. S. (2001). Genetic control of susceptibility to group A streptococcal infection in mice. *J Infect Dis*, 184(7):846–852.

Melgert, B. N., Postma, D. S., Kuipers, I., Geerlings, M., Luinge, M. A., van der Strate, B. W., Kerstjens, H. A., Timens, W., and Hylkema, M. N. (2005). Female mice are more susceptible to the development of allergic airway inflammation than male mice. *Clin Exp Allergy*, 35(11):1496–1503.

Mindnich, R., Hrabě de Angelis, M., and Adamski, J. (2007). Functional genome analysis indicates loss of 17beta-hydroxysteroid dehydrogenase type 2 enzyme in the zebrafish. *J Steroid Biochem Mol Biol*, 103(1):35–43.

Mogil, J. S. (1999). The genetic mediation of individual differences in sensitivity to pain and its inhibition. *Proc Natl Acad Sci U S A*, 96(14):7744–51.

Mogil, J. S., Wilson, S. G., Bon, K., Lee, S. E., Chung, K., Raber, P., Pieper, J. O., Hain, H. S., Belknap, J. K., Hubert, L., Elmer, G. I., Chung, J. M., and Devor, M. (1999). Heritability of nociception I: Responses of 11 inbred mouse strains on 12 measures of nociception. *Pain*, 80(1-2):67–82.

Möller, G. and Adamski, J. (2006). Multifunctionality of human 17beta-hydroxysteroid dehydrogenases. *Mol Cell Endocrinol*, 248(1-2):47–55.

Moro, C. and Berlan, M. (2006). Cardiovascular and metabolic effects of natriuretic peptides. *Fundam Clin Pharmacol*, 20(1):41–9.

Morton, N. M. and Seckl, J. R. (2008). 11beta-hydroxysteroid dehydrogenase type 1 and obesity. *Front Horm Res*, 36:146–64.

Nishikimi, T., Maeda, N., and Matsuoka, H. (2006). The role of natriuretic peptides in cardioprotection. *Cardiovasc Res*, 69(2):318–28.

Ohta, T., Iwakawa, M., Oohira, C., Noda, S., Minfu, Y., Goto, M., Tanaka, H., Harada, Y., and Imai, T. (2004). Fractionated irradiation augments inter-strain variation of skin reactions among three strains of mice. *J. Radiat. Res.*, 45(4):515–519.

Otis, A. B., Fenn, W. O., and Rahn, H. (1950). Mechanics of breathing in man. *J Appl Physiol*, 2(11):592–607.

Park, S. R., Seo, G. Y., Choi, A. J., Stavnezer, J., and Kim, P. H. (2005). Analysis of transforming growth factor-beta1-induced Ig germ-line gamma2b transcription and its implication for IgA isotype switching. *Eur J Immunol*, 35(3):946–56.

Pasche, B., Kalaydjiev, S., Franz, T. J., Kremmer, E., Gailus-Durner, V., Fuchs, H., Hrabě de Angelis, M., Lengeling, A., and Busch, D. H. (2005). Sex-dependent susceptibility to Listeria monocytogenes infection is mediated by differential interleukin-10 production. *Infect Immun*, 73(9):5952–5960.

Petkova, S. B., Yuan, R., Tsaih, S. W., Schott, W., Roopenian, D. C., and Paigen, B. (2008). Genetic influence on immune phenotype revealed strain-specific variations in peripheral blood lineages. *Physiol Genomics*, 34(3):304–14.

Pinheiro, J. and Bates, D. (2000). Mixed-Effect Models in S and S-PLUS, Springer, New York.

Prehn, C., Möller, G., and Adamski, J. (2009). Recent advances in 17beta-hydroxysteroid dehydrogenases. *J Steroid Biochem Mol Biol*, 114(1-2):72–7.

Prehn, C., Ströhle, F., Haller, F., Keller, B., Hrabě de Angelis, M., Adamski, J., and Mindnich, R. (2007). A Comparison Of Methods For Assays Of Steroidogenic Enzymes: New GC/MS Versus HPLC And TLC., volume 13, In: Enzymology and Molecular Biology of Carbonyl Metabolism (Weiner, H., Plapp, B., Lindhal, R., and Maser, E., eds), Purdue University Press, West Lafayette, Indiana, USA, 277-283.

Prusky, G. T., Alam, N. M., Beekman, S., and Douglas, R. M. (2004). Rapid quantification of adult and developing mouse spatial vision using a virtual optomotor system. *Invest Ophthalmol Vis Sci*, 45(12):4611–4616.

Puk, O., Dalke, C., Favor, J., Hrabě de Angelis, M., and Graw, J. (2006). Variations of eye size parameters among different strains of mice. *Mamm Genome*, 17(8):851–7.

Quackenbush, J. (2002). Microarray data normalization and transformation. *Nat Genet*, 32 Suppl:496–501.

Quimby, F. (1999). The Mouse., In: The clinical chemistry of laboratory animals (Loeb, W. and Quimby, F., eds), Taylor and Francis, New York, 3-31.

R Development Core Team (2009). R: A Language and Environment for Statistical Computing. R Foundation for Statistical Computing, Vienna, Austria. ISBN 3-900051-07-0.

Rainer, J., Sanchez-Cabo, F., Stocker, G., Sturn, A., and Trajanoski, Z. (2006). CARMAweb: comprehensive R- and bioconductor-based web service for microarray data analysis. *Nucleic Acids Res*, 34(Web Server issue):W498–503.

Rathkolb, B., Decker, T., Fuchs, E., Soewarto, D., Fella, C., Heffner, S., Pargent, W., Wanke, R., Balling, R., Hrabě de Angelis, M., Kolb, H. J., and Wolf, E. (2000). The clinical-chemical screen in the munich ENU mouse mutagenesis project: screening for clinically relevant phenotypes. *Mamm Genome*, 11(7):543–6.

Rauch, F. and Glorieux, F. H. (2004). Osteogenesis imperfecta. *Lancet*, 363(9418):1377–85.

Reinhard, C., Eder, G., Fuchs, H., Ziesenis, A., Heyder, J., and Schulz, H. (2002). Inbred strain variation in lung function. *Mamm Genome*, 13(8):429–37.

Reinhard, C., Meyer, B., Fuchs, H., Stoeger, T., Eder, G., Ruschendorf, F., Heyder, J., Nurnberg, P., Hrabě de Angelis, M., and Schulz, H. (2005). Genomewide linkage analysis identifies novel genetic loci for lung function in mice. *Am J Respir Crit Care Med*, 171(8):880–8.

Rissmann, R., Oudshoorn, M. H., Hennink, W. E., Ponec, M., and Bouwstra, J. A. (2009). Skin barrier disruption by acetone: observations in a hairless mouse skin model. *Arch. Dermatol. Res.*, 301(8):609–613.

Rivolta, C., Sharon, D., Hrabě de Angelis, M., and Dryja, T. P. (2002). Retinitis pigmentosa and allied diseases: numerous diseases, genes, and inheritance patterns. *Hum Mol Genet*, 11(10):1219–27.

Roederer, M., Nozzi, J. L., and Nason, M. X. (2011). SPICE: Exploration and analysis of post-cytometric complex multivariate datasets. *Cytometry A*, 79(2):167–74.

Rogers, D. C., Fisher, E. M., Brown, S. D., Peters, J., Hunter, A. J., and Martin, J. E. (1997). Behavioral and functional analysis of mouse phenotype: SHIRPA, a proposed protocol for comprehensive phenotype assessment. *Mamm Genome*, 8(10):711–3.

Rogner, U. C. and Avner, P. (2003). Congenic mice: cutting tools for complex immune disorders. *Nat Rev Immunol*, 3(3):243–52.

Rosen, C. J., Beamer, W. G., and Donahue, L. R. (2001). Defining the genetics of osteoporosis: using the mouse to understand man. *Osteoporos Int*, 12(10):803–810.

Roth, D. M., Swaney, J. S., Dalton, N. D., Gilpin, E. A., and Ross, J. (2002). Impact of anesthesia on cardiac function during echocardiography in mice. *Am J Physiol Heart Circ Physiol*, 282(6):H2134–H2140.

Royer, A., van Veen, T. A., Le Bouter, S., Marionneau, C., Griol-Charhbili, V., Leoni, A. L., Steenman, M., van Rijen, H. V., Demolombe, S., Goddard, C. A., Richer, C., Escoubet, B., Jarry-Guichard, T., Colledge, W. H., Gros, D., de Bakker, J. M., Grace, A. A., Escande, D., and Charpentier, F. (2005). Mouse model of SCN5A-linked hereditary Lenegre's disease: age-related conduction slowing and myocardial fibrosis. *Circulation*, 111(14):1738–46.

Rubinstein, L. J. and Stein, K. E. (1988). Murine immune response to the Neisseria meningitidis group C capsular polysaccharide. I. ontogeny. *J Immunol*, 141(12):4352–6.

Saarikangas, J., Mattila, P. K., Varjosalo, M., Bovellan, M., Hakanen, J., Calzada-Wack, J., Tost, M., Jennen, L., Rathkolb, B., Hans, W., Horsch, M., Hyvönen, M. E., Perälä, N., Fuchs, H., Gailus-Durner, V., Esposito, I., Wolf, E., Hrabě de Angelis, M., Frilander, M. J., Savilahti, H., Sariola, H., Sainio, K., Lehtonen, S., Taipale, J., Salminen, M., and Lappalainen, P. (2011). Missing-in-metastasis MIM/MTSS1 promotes actin assembly at intercellular junctions and is required for integrity of kidney epithelia. *J Cell Sci*, 124(Pt 8):1245–1255.

Saeed, A. I., Sharov, V., White, J., Li, J., Liang, W., Bhagabati, N., Braisted, J., Klapa, M., Currier, T., Thiagarajan, M., Sturn, A., Snuffin, M., Rezantsev, A., Popov, D.,

Ryltsov, A., Kostukovich, E., Borisovsky, I., Liu, Z., Vinsavich, A., Trush, V., and Quackenbush, J. (2003). TM4: a free, open-source system for microarray data management and analysis. *Biotechniques*, 34(2):374–8.

Sahn, D. J., DeMaria, A., Kisslo, J., and Weyman, A. (1978). Recommendations regarding quantitation in M-mode echocardiography: results of a survey of echocardiographic measurements. *Circulation*, 58(6):1072–83.

Sant'Anna, O. A., Mouton, D., Ibanez, O. M., Bouthillier, Y., Mevel, J. C., Reis, M. H., and Biozzi, G. (1985). Basal immunoglobulin serum concentration and isotype distribution in relation to the polygenic control of antibody responsiveness in mice. *Immunogenetics*, 22(2):131–139.

Schmidt, C., Priemel, M., Kohler, T., Weusten, A., Muller, R., Amling, M., and Eckstein, F. (2003). Precision and accuracy of peripheral quantitative computed tomography (pQCT) in the mouse skeleton compared with histology and microcomputed tomography (μCT). *J Bone Miner Res*, 18(8):1486–96.

Schmucker, C. and Schaeffel, F. (2004). In vivo biometry in the mouse eye with low coherence interferometry. *Vision Res*, 44(21):2445–56.

Schmucker, C., Seeliger, M., Humphries, P., Biel, M., and Schaeffel, F. (2005). Grating acuity at different luminances in wild-type mice and in mice lacking rod or cone function. *Invest Ophthalmol Vis Sci*, 46(1):398–407.

Schneider, I., Tirsch, W. S., Faus-Kessler, T., Becker, L., Kling, E., Busse, R. L., Bender, A., Feddersen, B., Tritschler, J., Fuchs, H., Gailus-Durner, V., Englmeier, K. H., Hrabě de Angelis, M., and Klopstock, T. (2006). Systematic, standardized and comprehensive neurological phenotyping of inbred mice strains in the German Mouse Clinic. *J Neurosci Methods*, 157(1):82–90.

Schoensiegel, F., Bekeredjian, R., Schrewe, A., Weichenhan, D., Frey, N., Katus, H. A., and Ivandic, B. T. (2007). Atrial natriuretic peptide and osteopontin are useful markers of cardiac disorders in mice. *Comp Med*, 57(6):546–53.

Schoensiegel, F., Ivandic, B., Geis, N. A., Schrewe, A., Katus, H. A., and Bekeredjian, R. (2011). High throughput echocardiography in conscious mice: training and primary screens. *Ultraschall Med*, 32 Suppl 1:S124–S129.

Seckl, J. R., Morton, N. M., Chapman, K. E., and Walker, B. R. (2004). Glucocorticoids and 11beta-hydroxysteroid dehydrogenase in adipose tissue. *Recent Prog Horm Res*, 59:359–93.

Seltmann, M., Horsch, M., Drobyshev, A., Chen, Y., Hrabě de Angelis, M., and Beckers, J. (2005). Assessment of a systematic expression profiling approach in ENU-induced mouse mutant lines. *Mamm Genome*, 16(1):1–10.

Seong, E., Seasholtz, A. F., and Burmeister, M. (2002). Mouse models for psychiatric disorders. *Trends Genet*, 18(12):643–50.

Seymour, B., Friebertshauser, K. E., Peake, J., Pinkerton, K. E., Coffman, R. L., and Gershwin, L. J. (2002). Gender differences in the allergic response of mice neonatally ex-posed to environmental tobacco smoke. *Dev Immunol*, 9(1):47–54.

Simard, J., Rheaume, E., Sanchez, R., Laflamme, N., de Launoit, Y., Luu-The, V., van Seters, A. P., Gordon, R. D., Bettendorf, M., Heinrich, U., and et al. (1993). Molecular basis of congenital adrenal hyperplasia due to 3 beta-hydroxysteroid dehydrogenase deficiency. *Mol Endocrinol*, 7(5):716–28.

Spiegelman, B. M. and Flier, J. S. (2001). Obesity and the regulation of energy balance. *Cell*, 104(4):531–43.

Stevens, T. L., Bossie, A., Sanders, V. M., Fernandez-Botran, R., Coffman, R. L., Mosmann, T. R., and Vitetta, E. S. (1988). Regulation of antibody isotype secretion by subsets of antigen-specific helper T cells. *Nature*, 334(6179):255–8.

Stirling, J., Coleman, M., Thomas, A., and Woods, A. (1999). Role of transmission electron microscopy in tissue diagnosis: diseases of the kidney, skeletal muscle and myocardium. *J Cell Pathol*, 4:223–243.

Stone, E. M., Sheffield, V. C., and Hageman, G. S. (2001). Molecular genetics of age-related macular degeneration. *Hum Mol Genet*, 10(20):2285–92.

Swerdlow, N. R., Braff, D. L., Taaid, N., and Geyer, M. A. (1994). Assessing the validity of an animal model of deficient sensorimotor gating in schizophrenic patients. *Arch Gen Psychiatry*, 51(2):139–54.

Syed, F., Diwan, A., and Hahn, H. S. (2005). Murine echocardiography: a practical approach for phenotyping genetically manipulated and surgically modeled mice. *J Am Soc Echocardiogr*, 18(9):982–90.

Tankersley, C. G. (1999). Genetic control of ventilation: what are we learning from murine models? *Curr Opin Pulm Med*, 5(6):344–8.

Tankersley, C. G., Fitzgerald, R. S., Levitt, R. C., Mitzner, W. A., Ewart, S. L., and Kleeberger, S. R. (1997). Genetic control of differential baseline breathing pattern. *J Appl Physiol*, 82(3):874–81.

Tarantino, L. M. and Bucan, M. (2000). Dissection of behavior and psychiatric disorders using the mouse as a model. *Hum Mol Genet*, 9(6):953–65.

Teichholz, L. E., Cohen, M. V., Sonnenblick, E. H., and Gorlin, R. (1974). Study of left ventricular geometry and function by B-scan ultrasonography in patients with and without asynergy. *N Engl J Med*, 291(23):1220–6.

Turner, C. H., Hsieh, Y. F., Muller, R., Bouxsein, M. L., Baylink, D. J., Rosen, C. J., Grynpas, M. D., Donahue, L. R., and Beamer, W. G. (2000). Genetic regulation of cortical and trabecular bone strength and microstructure in inbred strains of mice. *J Bone Miner Res*, 15(6):1126–31.

Tusher, V. G., Tibshirani, R., and Chu, G. (2001). Significance analysis of microarrays applied to the ionizing radiation response. *Proc Natl Acad Sci U S A*, 98(9):5116–21.

Vanoirbeek, J. A. J., Rinaldi, M., Vooght, V. D., Haenen, S., Bobic, S., Gayan-Ramirez, G., Hoet, P. H. M., Verbeken, E., Decramer, M., Nemery, B., and Janssens, W. (2010). Noninvasive and invasive pulmonary function in mouse models of obstructive and restrictive respiratory diseases. *Am J Respir Cell Mol Biol*, 42(1):96–104.

Wall, P. and Melzack, R., eds (1984). Textbook of Pain, Churchill Livingstone, London.

Weaver, J. L., Broud, D. D., McKinnon, K., and Germolec, D. R. (2002). Serial phenotypic analysis of mouse peripheral blood leukocytes. *Toxicol Mech Methods*, 12(2):95–118.

Weiss, S. M., Lightowler, S., Stanhope, K. J., Kennett, G. A., and Dourish, C. T. (2000). Measurement of anxiety in transgenic mice. *Rev Neurosci*, 11(1):59–74.

Williams, T. D., Chambers, J. B., Henderson, R. P., Rashotte, M. E., and Overton, J. M. (2002). Cardiovascular responses to caloric restriction and thermoneutrality in C57BL/6J mice. *Am J Physiol Regul Integr Comp Physiol*, 282(5):R1459–67.

Willott, J. F., ed (2001). In: Handbook of Mouse Auditory Research. From Behaviour to molecular Biology (Willott, J. F., ed), CRS Press, Boca Raton, FL, USA.

Wjst, M., Fischer, G., Immervoll, T., Jung, M., Saar, K., Rueschendorf, F., Reis, A., Ulbrecht, M., Gomolka, M., Weiss, E. H., Jaeger, L., Nickel, R., Richter, K., Kjellman, N. I., Griese, M., von Berg, A., Gappa, M., Riedel, F., Boehle, M., van Koningsbruggen, S., Schoberth, P., Szczepanski, R., Dorsch, W., Silbermann, M., Wichmann, H. E., and et al. (1999). A genome-wide search for linkage to asthma. German Asthma Genetics Group. *Genomics*, 58(1):1–8.

Yang, Y. H., Dudoit, S., Luu, P., Lin, D. M., Peng, V., Ngai, J., and Speed, T. P. (2002). Normalization for cDNA microarray data: a robust composite method addressing single and multiple slide systematic variation. *Nucleic Acids Res*, 30(4):e15.

Yap, L. B., Mukerjee, D., Timms, P. M., Ashrafian, H., and Coghlan, J. G. (2004). Natriuretic peptides, respiratory disease, and the right heart. *Chest*, 126(4):1330–6.

Yellon, S. M. and Tran, L. T. (2002). Photoperiod, reproduction, and immunity in select strains of inbred mice. *J Biol Rhythms*, 17(1):65–75.

Zamai, L., Falcieri, E., Marhefka, G., and Vitale, M. (1996). Supravital exposure to propidium iodide identifies apoptotic cells in the absence of nucleosomal dna fragmentation. *Cytometry*, 23(4):303–11.

Zhou, Y. Q., Foster, F. S., Nieman, B. J., Davidson, L., Chen, X. J., and Henkelman, R. M. (2004). Comprehensive transthoracic cardiac imaging in mice using ultrasound biomicroscopy with anatomical confirmation by magnetic resonance imaging. *Physiol Genomics*, 18(2):232–44.

Appendix

Abbreviations

Ab	Antibody
ABR	Auditory Brainstem Response
ALAT/GPT	alanine-aminotransferase
ALP	alkaline phosphatase
ALUM	Aluminum hydroxide
ANOVA	analysis of variance
ANP	atrial natriuretic peptide
ARMD	age-related-macular-degeneration
ASAT/GOT	aspartate aminotransferase
ASR	acoustic startle reactivity
AST	apartate aminotransferase
AUC	area under curve
BAL	bronchoalveolar lavage
BALF	bronchoalveolar lavage fluid
BMC	bone mineral content (excluding skull)
BMD	bone mineral density (excluding skull)
BP	blood pressure
bm	body mass (g)
bw	body weight (g)
CBC	complete peripheral blood count
Cchord	Chord Compliance
CD	cluster of differentiation
Cdyn	Dynamic Lung Compliance
CET	Central European Time
CK	creatine kinase
Creatinine enz.	Creatinine values determined using an enzymatic method
DEXA/DXA	dual-energy X-ray absorptiometry
Echo	echocardiography
ECG	electrocardiography
EDTA	ethylenediaminetetraacetic acid
EDV	end-diastolic volume
EF	Ejection fraction
E_{fec}	energy content of the feces
ELISA	enzyme-linked immunosorbent assay
EMPReSS	**E**uropean **M**ouse **P**henotyping **Re**source for **S**tandardised **S**creens (http://empress.har.mrc.ac.uk/)

EUMODIC	The **Eu**ropean **Mo**use **Di**sease **C**linic (http://www.eumodic.org/)
EUMORPHIA	**E**uropean **U**nion **Mo**use **R**esearch for **P**ublic **H**ealth and **I**ndustrial **A**pplications (http://www.eumorphia.org/index.html)
ENU	N-ethyl-N-nitrosourea
E_{met}	metabolizable energy
ERG	electroretinography
ERV	Expiratory Reverse Volume
EuroPhenome	database to hold phenome data obtained from the EMPReSS SOPs (http://www.europhenome.org/)
E_{up}	energy uptake
ES cell	embryonic stem cell
ESV	end-systolic volume
f	respiratory rate (1/min)
FACS	fluorescence-activated cell sorting
Fass	food assimilation coefficient
Fcon	food consumption
FDR	false discovery rate
Fec	daily feces production
FEV100	Forced Expiratory Volume
FELASA	**F**ederation of **E**uropean **L**aboratory **A**nimal **S**cience **A**ssociations, 25 Shaftesbury Avenue, London W1D 7EG, UK, http://www.felasa.org
FRC	Functional Residual Capacity
FS	Fractional shortening
FVC	Forced Vital Capacity
GLM	Generalized Linear Model
GMC	German Mouse Clinic
GO	Gene Ontology
GWA study	genome wide association study
H&E	hematoxylin and eosin
HCT	hematocrit
HGB	hemoglobin
h.p.	hind paw
HP	heat production
HPLC	high pressure liquid chromatography
HTC	hematocrit
HR	heart rate
HRV	heart rate variability
IC	Inspiratory Capacity

Ig	immunoglobulin
i.p.	intraperitoneal
IpGTT	intraperitoneal glucose tolerance test
i.v.	Intravenously
IVC	individually ventilated cage
IVSd	Interventricular septum in diastole
IVSs	Interventricular septum in systole
KI	knock-in
KO	knock-out
LCMS / LC-MS/MS	liquid chromatography mass spectrometry
LDH	lactate-dehydrogenase
LED	light-emitting diode
LIB	laser interference biometry
LVIDd	Left ventricular internal dimension in diastole
LVIDs	Left ventricular internal dimension in systole
LV MassCor	Left ventricular mass corrected
LVPWd	Left ventricular posterior wall in diastole
LVPWs	Left ventricular posterior wall in systole
LVvolD	Left ventricular volume in diastole
LVvolS	Left ventricular volume in systole
mAb	Monoclonal antibody
MCH	mean corpuscular hemoglobin
MCHC	mean corpuscular hemoglobin concentrations
MCV	mean corpuscular volume
mean_f	mean of all respiratory rates (1/min)
µCT	micro computed tomography
MPV	mean platelet volume
MPV	mean platelet volume
MV	minute ventilation (ml/min)
n.a.	not analyzed
n.s.	not significant
NAD	no abnormality detected
NEFA	non-esterified fatty acid
OCT	optical coherence tomography
OF	Open Field
OVA	Ovalbumin
P1	pipeline 1

P2	pipeline 2
PBC	peripheral blood cells
PBL	peripheral blood leukocyte
PEF	peak expiratory flow rate (ml/s)
PLT	platelet count
PPI	Prepulse Inhibition
pQCT	peripheral quantitative computed tomography
R	Lung Resistance
RBC	red blood cell count
RDW	red blood cell distribution width
RV	Residual Volume
SAM	Significance Analysis of Microarray
sd	standard deviation
SHIRPA	**S**mithKline Beecham Pharmaceuticals, **H**arwell, MRC Mouse Genome Centre and Mammalian Genetics Unit, **I**mperial College School of Medicine at St Mary's **R**oyal London Hospital, St Bartholomew's and the Royal London School of Medicine **P**henotype **A**ssessment http://www.har.mrc.ac.uk/services/phenotyping/neurology/shirpa.html
SLP	Sound Pressure Level
SOP	standard operation protocol / procedure
sTV	specific tidal volume (µl/g)
SV	Stroke volume
TD-NMR	Time Domain NMR
TIBC	total iron-binding capacity
TLC	Total Lung Capacity
TP	total protein
Tre	rectal temperature
TLC	Total Lung Capacity
TV	Tidal Volume
VC	Vital Capacity
WBC	white blood cell count

List of Figures

Appendix B

1 Workflow of the primary screen. 12
2 Test Arena for Open Field test (www.TSE-Systems.com). 17
3 The grip strength apparatus 20
4 The rotarod apparatus (Bioseb, Chaville, France) 21
5 ABR setup 23
6 Click-evoked ABR traces 24
7 Hotplate system 26
8 X-ray analysis (Faxitron X-ray Model MX-20-DC12). The red beam allows the subject to be positioned so that the area of interest is directly over the X-ray sensor. 28
9 DEXA analysis (pDEXA Sabre X-ray Bone Densitometer) 29
10 Indirect Calorimetry 31
11 Vevo®2100 High-Resolution Imaging System 34
12 Echocardiographic measurements in conscious mouse, screenshots 35
13 Example of ECG trace with analysed parameters 36
14 AC Master 39
15 Spectralis OCT 39
16 Pentacam digital camera system 40
17 Virtual Vision Test 41
18 Frequencies of main lineages refer to a CD45+ gate 47
19 Buxco FinePointe RC system (left) and Buxco Forced maneuvers system (right). 52
20 **Body mass:** Time curve of the mutant and control cohorts. 60
21 Manhattan Plot of Effect Sizes (female mice). 61
22 Manhattan Plot of Effect Sizes (male mice). 62
23 **Open Field:** Distance Traveled, average plot, split by sex and genotype 63
24 **Open Field:** Distance traveled - Total boxplot with stripchart, split by sex and genotype 64
25 **Open Field:** Number of rears, average plot, split by sex and genotype 64
26 **Open Field:** Number of rears - Total boxplot with stripchart, split by sex and genotype 65
27 **Open Field:** Distance in the center, average plot, split by sex and genotype 65
28 **Open Field:** Percent distance in the center - Total boxplot with stripchart, split by sex and genotype 66
29 **Open Field:** Percent Time Spent in the Center, average plot, split by sex and genotype 66
30 **Open Field:** Percent time spent in the center - Total boxplot with stripchart, split by sex and genotype 67
31 **Acoustic Startle:** Acoustic startle reactivity, average plot, split by sex and genotype 69
32 **Prepulse Inhibition:** PPI grouped barplot, split by sex and genotype 70
33 **Prepulse Inhibition:** Startle amplitude - ST110 boxplot with stripchart, split by sex and genotype 70
34 **Modified SHIRPA:** Locomotor Activity boxplot, split by sex and genotype 73

35 **Modified SHIRPA:** Tail Elevation proportion bar plot, split by sex and genotype 74
36 **Modified SHIRPA:** Urination proportion bar plot, split by sex and genotype 75
37 **Rotarod:** Latency to fall (mean) boxplot with stripchart, split by sex and genotype 79
38 **Rotarod:** Latency to fall, average plot, split by sex and genotype . . . 80
39 **Lactate:** Boxplot with stripchart, split by sex and genotype 81
40 **ABR:** Stimulus intensity thresholds measured for different frequencies, medians and quartiles. Values above measurement limit (85db) are replaced by 100. 82
41 **Hot Plate Test:** 1st response time boxplot with stripchart, split by sex and genotype 84
42 **Hot Plate Test:** 2nd response time boxplot with stripchart, split by sex and genotype 85
43 **DXA analysis:** Bone mineral content (BMC) vs. Body weight scatterplot 87
44 **Indirect Calorimetry:** Mean oxygen consumption [ml/(h animal)] versus body mass [g] scatterplot with regression lines 89
45 **Body Composition Analysis:** Fat mass NMR, average plot, split by sex and genotype 90
46 **Body Composition Analysis:** Fat mass vs. body mass scatterplot with regression lines 91
47 **Body Composition Analysis:** Fat mass vs. body mass scatterplot with regression lines 92
48 **Body Composition Analysis:** Lean mass NMR, average plot, split by sex and genotype 93
49 **Body Composition Analysis:** Fat mass vs. body mass scatterplot with regression lines 93
50 **Body Composition Analysis:** Fat mass vs. body mass scatterplot with regression lines 94
51 **Body Composition Analysis:** Body mass NMR, average plot, split by sex and genotype 94
52 **Echocardiography:** LVIDd boxplot with stripchart, split by sex and genotype 96
53 **Echocardiography:** LVIDs boxplot with stripchart, split by sex and genotype 97
54 **Echocardiography:** Heart rate Echo boxplot with stripchart, split by sex and genotype 97
55 **Echocardiography:** Respiration Rate Echo boxplot with stripchart, split by sex and genotype 98
56 **Echocardiography:** Ejection fraction boxplot with stripchart, split by sex and genotype 98
57 **Echocardiography:** Fractional shortening boxplot with stripchart, split by sex and genotype 99
58 **Echocardiography:** Left ventricular mass corrected boxplot with stripchart, split by sex and genotype 99
59 **Echocardiography:** Stroke Volume boxplot with stripchart, split by sex and genotype 100

60 **Electrocardiography:** PQ boxplot with stripchart, split by sex and genotype . . . 102
61 **Electrocardiography:** PR boxplot with stripchart, split by sex and genotype . . . 103
62 **Eye Size:** Axial length right eye boxplot with stripchart, split by sex and genotype . . . 104
63 **OCT:** right fundus number of main vessels boxplot with stripchart, split by sex and genotype . . . 105
64 **OCT:** right retinal thickness boxplot with stripchart, split by sex and genotype . . . 105
65 **Anterior Eye Investigation:** mean(lens density right eye) boxplot with stripchart, split by sex and genotype . . . 106
66 **IpGTT:** Body mass loss vs. body weight before overnight food withdrawal . . . 108
67 **IpGTT:** Blood glucose concentration average plot, split by sex and genotype . . . 109
68 **IpGTT:** Glucose (T=0) boxplot with stripchart, split by sex and genotype 109
69 **Clinical Chemistry (unfed mice):** fasting Glucose boxplot with stripchart, split by sex and genotype . . . 110
70 **Clinical Chemistry (unfed mice):** fasting NEFA boxplot with stripchart, split by sex and genotype . . . 111
71 **Clinical Chemistry (unfed mice):** fasting Glycerol boxplot with stripchart, split by sex and genotype . . . 111
72 **Clinical Chemistry (unfed mice):** fasting non-HDL Cholesterol boxplot with stripchart, split by sex and genotype . . . 112
73 **Clinical Chemistry:** Albumin boxplot with stripchart, split by sex and genotype . . . 113
74 **Clinical Chemistry:** Total protein boxplot with stripchart, split by sex and genotype . . . 114
75 **Clinical Chemistry:** Sodium boxplot with stripchart, split by sex and genotype . . . 114
76 **Clinical Chemistry:** Creatinine enz. boxplot with stripchart, split by sex and genotype . . . 115
77 **Clinical Chemistry:** ALAT/GPT boxplot with stripchart, split by sex and genotype . . . 116
78 **Clinical Chemistry:** ASAT/GOT boxplot with stripchart, split by sex and genotype . . . 116
79 **Clinical Chemistry:** Triglyceride boxplot with stripchart, split by sex and genotype . . . 117
80 **Clinical Chemistry:** Inorganic phosphate boxplot with stripchart, split by sex and genotype . . . 118
81 **Clinical Chemistry:** ALP boxplot with stripchart, split by sex and genotype . . . 118
82 **Hematology:** Red blood cell boxplot with stripchart, split by sex and genotype . . . 120
83 **Hematology:** White blood cell boxplot with stripchart, split by sex and genotype . . . 121
84 **Hematology:** Hemoglobin boxplot with stripchart, split by sex and genotype . . . 121

85 **Hematology:** Hematocrit boxplot with stripchart, split by sex and genotype . . . 122
86 **Hematology:** Mean corpuscular volume boxplot with stripchart, split by sex and genotype . . . 122
87 **Hematology:** Mean corpuscular hemoglobin content boxplot with stripchart, split by sex and genotype . . . 123
88 **Hematology:** Mean corpuscular hemoglobin concentration boxplot with stripchart, split by sex and genotype . . . 123
89 **Hematology:** Distribution index of red blood cells boxplot with stripchart, split by sex and genotype . . . 124
90 **Main leukocyte populations:** Leukocytes pie chart, split by sex and genotype . . . 128
91 **Main leukocyte populations:** CD45+/CD11b+Ly6G+ boxplot with stripchart, split by sex and genotype . . . 129
92 **Main leukocyte populations:** CD45+/Ly6C+CD11b+ boxplot with stripchart, split by sex and genotype . . . 129
93 **Monocyte subsets:** Monocyte pie chart, split by sex and genotype . 130
94 **T cell subsets:** T cell subpopulations pie chart, split by sex and genotype . . . 131
95 **T cell subsets:** CD4+/CD25+ boxplot with stripchart, split by sex and genotype . . . 132
96 **T cell subsets:** CD4+/Ly6C+ boxplot with stripchart, split by sex and genotype . . . 132
97 **T cell subsets:** CD4 T cell subpopulations pie chart, split by sex and genotype . . . 133
98 **T cell subsets:** CD8 T cell subpopulations pie chart, split by sex and genotype . . . 133
99 **B cell subsets:** B/CD11b+ boxplot with stripchart, split by sex and genotype . . . 136
100 **B cell subsets:** B-2 B cell subpopulations pie chart, split by sex and genotype . . . 136
101 **Total IgE in Plasma:** IgE boxplot with stripchart, split by sex and genotype . . . 140
102 **TEWL:** Transepidermal water loss boxplot with stripchart, split by sex and genotype . . . 141
103 **Organ Weight:** Liver Weight / Tibia Length boxplot with stripchart, split by sex and genotype . . . 146
104 **Organ Weight:** Spleen Weight / Tibia Length boxplot with stripchart, split by sex and genotype . . . 147
105 **Organ Weight:** Body weight boxplot with stripchart, split by sex and genotype . . . 147
106 **Organ Weight:** Heart Weight / Tibia Length boxplot with stripchart, split by sex and genotype . . . 148
107 . . . 150
108 . . . 151

List of Tables

1 Primary Screen at GMC . . . 13
2 Behaviour parameters . . . 18
3 SHIRPA parameters . . . 20
4 Nociceptive parameters . . . 26
5 Dysmorphology, Bone and Cartilage parameters . . . 30
6 Metabolic parameters . . . 32
7 Echocardiography parameters and abbreviations . . . 34
8 Echocardiography parameters and abbreviations . . . 36
9 Heart Weight Analysis . . . 37
10 Eye parameters . . . 42
11 Clinical chemical parameters . . . 45
12 Parameters measured by the Immunology Screen . . . 48
13 Lung parameters and abbreviations . . . 53
14 Results in a nutshell. . . . 57
15 Number of analyzed mice. . . . 58
16 Age of mice when the specific test has been applied. . . . 59
17 **Open Field:** Means, standard deviation and p-values calculated by a linear model . . . 68
18 **Modified SHIRPA:** Locomotor activity and body mass (SHIRPA): Means, standard deviation and p-values calculated by a linear model 74
19 **Modified SHIRPA:** Results ([1] Fisher's Exact test) . . . 76
20 **Modified SHIRPA:** Results ([1] Fisher's Exact test) . . . 77
21 **Grip Strength:** Linear model for 2-paws grip strength (mean of 3 trials) 78
22 **Grip Strength:** Group means and standard deviation . . . 78
23 **Grip Strength:** Linear model for 4-paws grip strength (mean of 3 trials) 78
24 **Rotarod:** Latency to fall . . . 79
25 **Rotarod:** Linear Mixed Effect Model . . . 79
26 **Rotarod:** Passive Rotation [1] Fisher's Exact test (α =0.05) . . . 80
27 **Lactate:** Mean and standard deviation, split by sex and genotype . . 81
28 **Lactate:** Linear Model with interactions Lactate . . . 82
29 **ABR:** Means, standard deviation and p-values calculated by a Wilcoxon rank-sum test. Values above measurement limit (85db) are replaced by 100.[a] Number not based on the full number of animals (missing values) . . . 83
30 **ABR:** Linear Mixed Effect Model. With replacement for values above measurement limit. . . . 83
31 **Hot Plate Test:** Observed reaction . . . 85
32 **Hot Plate Test:** First and second response time, group mean and standard deviation and ANOVA including post hoc calculation 85
33 **DXA analysis:** Group means, standard deviation and ANOVA (Tukey multiple comparisons of means) . . . 88
34 **Indirect Calorimetry:** Means, standard deviation and p-values of a Linear Model . . . 90
35 **Body Composition Analysis:** p-values of Linear Mixed Models . . . 91
36 **Body Composition Analysis:** Means, standard deviation and p-values of a Linear Model, first measurement . . . 91

37 **Body Composition Analysis:** Means, standard deviation and p-values of a Linear Model, second measurement 92
38 **Echocardiography:** Mean and standard deviation, split by sex and genotype 100
39 **Echocardiography:** Medians, first and third quartile and p-values calculated by a Wilcoxon rank-sum test[a] Number not based on the full number of animals (missing values) 101
40 **IpGTT:** Linear model including sex, genotype and body mass before overnight food withdrawal as well as sex:genotype interaction as factors influencing fasting-induced body mass loss. 107
41 **IpGTT:** Basal glucose level and area under the curve (AUC) for the first 30 minutes and the remaining 90 minutes of the test obtained from glucose tolerance testing: Means, standard deviation and p-values calculated by a linear model. 108
42 **Clinical Chemistry (unfed mice):** Fasting plasma lipid and glucose levels determined after overnight food-withdrawal. Means, standard deviations and p-values for genotype, sex and genotype × sex interaction effects calculated by a linear model. 112
43 **Clinical Chemistry:** Plasma electrolyte, protein, creatinine and urea concentrations of ad libitum fed mice. Means, standard deviations and p-values for genotype, sex and genotype × sex interaction effects calculated by a linear model. 115
44 **Clinical Chemistry:** Lipid and glucose levels as well as selected enzyme activities in plasma of ad libitum fed mice. Means, standard deviations and p-values for genotype, sex and genotype × sex interaction effects calculated by a linear model. 117
45 **Clinical Chemistry:** Plasma concentrations of minerals, iron and ALP activity of ad libitum fed mice. Means, standard deviations and p-values for genotype, sex and genotype × sex interaction effects calculated by a linear model. 119
46 **Hematology:** Values measured in EDTA-blood samples. Means, standard deviations and p-values for genotype, sex and genotype × sex interaction effects calculated by a linear model. 124
47 **Main leukocyte populations:** Frequencies of main leukocyte populations in peripheral blood (protocol without erythrocyte lysis) [percentage of all leukocytes (CD45+ cells)]. Medians, first and third quartile and p-values calculated by a Wilcoxon rank-sum test[a] Number not based on the full number of animals (missing values) 130
48 **Monocyte subsets:** Frequencies of monocyte subsets in peripheral blood (protocol without erythrocyte lysis) [percentage of all monocytes]. Medians, first and third quartile and p-values calculated by a Wilcoxon rank-sum test[a] Number not based on the full number of animals (missing values) 131
49 **Main leukocyte populations:** Summary of total leukocyte count . . 131
50 **NK cell subsets:** Frequencies of NK cell subpopulations [percentage of NK cells]. Medians, first and third quartile and p-values calculated by a Wilcoxon rank-sum test[a] Number not based on the full number of animals (missing values) 134

51 **T cell subsets:** Frequencies of main T cell subpopulations (CD4, CD8, gamma-delta) [percentage of T cells]. Medians, first and third quartile and p-values calculated by a Wilcoxon rank-sum test[a] Number not based on the full number of animals (missing values) 134
52 **T cell subsets:** Frequencies of CD4 AND CD8 T cell subpopulations (CD25, CD44, CD62L, Ly6C). Medians, first and third quartile and p-values calculated by a Wilcoxon rank-sum test[a] Number not based on the full number of animals (missing values) 135
53 **T cell subsets:** Frequencies of CD4 and CD8 T cell subpopulations (Boolean gates CD44 high, CD62L, Ly6C). Medians, first and third quartile and p-values calculated by a Wilcoxon rank-sum test[a] Number not based on the full number of animals (missing values) 135
54 **B cell subsets:** Frequencies of B cell subpopulations [percentage of all B cells]. Medians, first and third quartile and p-values calculated by a Wilcoxon rank-sum test[a] Number not based on the full number of animals (missing values) 137
55 **B cell subsets:** B-2 B cell subpopulations (Boolean gates CD21,CD23, IgD, IgM). Medians, first and third quartile and p-values calculated by a Wilcoxon rank-sum test[a] Number not based on the full number of animals (missing values) 137
56 **B cell subsets:** Summary of total leukocyte count and B cells count 138
57 **Total IgE in Plasma:** Medians, first and third quartile and p-values calculated by a Wilcoxon rank-sum test 141
58 **TEWL:** Medians, first and third quartile and p-values calculated by a Wilcoxon rank-sum test 142
59 **Organ Weight:** Medians, first and third quartile and p-values calculated by a Wilcoxon rank-sum test[a] Number not based on the full number of animals (missing values) 148
60 **Pathology:** Genotype-specific differences - overview 149

German
Mouse Clinic

The
GERMAN MOUSE CLINIC (GMC)
at the
Helmholtz Zentrum München
German Research Center for Environmental Health

Molecular Phenotyping
Report for Cldn12

HelmholtzZentrum münchen
German Research Center for Environmental Health

HELMHOLTZ
ASSOCIATION

Confidential Data

This report was generated automatically using a specific database script. The information enclosed in this report was prepared with greatest care. However, it cannot be excluded that unintentional and unexpected formatting or spelling errors may have occurred.

Molecular Phenotyping 4.14

Summary 4.14.1

In this report we describe the results of transcript profiling of the *Cldn12* mutant mice. Heart was chosen for transcriptome analysis due to the finding in the echocardiography performed in the cardiovascular screen. Experiments were performed for four male mutant and four wild type mice as controls. Data analysis using various statistical methods detected moderate but significant differences in gene expression patterns associated with atherosclerosis, apoptosis, inflammatory response and concentration of lipid.

Results 4.14.2

Molecular Phenotyping
For transcriptome analysis heart was selected as organ for the *Clnd12* mouse line. We isolated RNA of this organ of four male mutant mice and four control animals at the age of 22 weeks and performed 8 hybridizations.

Inspection of expression patterns of heart from individual mice revealed a stronger correlation of regulated genes in mutant samples 30299069, 30299070 and 30299071. In sample 30299068, gene expression was not significantly changed or showed a reverse tendency compared to the other mutant samples (Table 103). Maybe biological variability in gene expression, oscillation or stress responsive genes were potential reasons for anti-correlation in the expression patterns between single individuals. Several recent publications have provided evidence for biological variability of expression levels for particular genes (Churchill, 2002; Drobyshev et al, 2003; Oishi et al, 2003; Pritchard et al, 2001; Seltmann et al, 2005). For statistical analysis only three mutant samples were compared to the mean value of four control mice.

Statistical analysis of gene expression patterns identified 34 significantly regulated genes. The estimated number of falsely significant genes was calculated for 1000 random permutations, yielding a FDR of 0%. Table 61 summarizes the results of the chip hybridizations: The expression differences between mutant and control tissues are represented by the fold changes (mutant/mean control). The genes are listed from strong to moderated overexpression, switch to moderate down-regulation and ends with those genes strongly down-regulated. The scale bar encodes the ratio of the fold induction. Blue color corresponds to transcriptional repression, yellow corresponds to transcriptional induction in mutant mice, and the color intensity reflects the magnitude of the change in average gene expression.

The significantly regulated gene of heart were classified by their molecular functions (GO term analysis) using the Ingenuity pathway analysis software. Tabel 60 summarizes those categories that are overrepresented and selected due to their p-values.

Table 59
Scale bare encoding the linear ratio of the fold induction.

Value	Color		Value	Color
(-∞ , -6]			(1 , 1.5]	
(-6 , -5.5]			(1.5 , 2]	
(-5.5 , -5]			(2 , 2.5]	
(-5 , -4.5]			(2.5 , 3]	
(-4.5 , -4]			(3 , 3.5]	
(-4 , -3.5]			(3.5 , 4]	
(-3.5 , -3]			(4 , 4.5]	
(-3 , -2.5]			(4.5 , 5]	
(-2.5 , -2]			(5 , 5.5]	
(-2 , -1.5]			(5.5 , 6]	
(-1.5 , 1]			(6 , ∞)	

Table 60
Functional classification of regulated genes in heart.

Category	Molecules
atherosclerosis	Adipoq, Fmn2, Hp, Lep, S100a8, S100a9, Scd, Tkt
apoptosis	Acly, Adipoq, Ca3, Cidec, Cyp2e1, Lep, Ntrk2, Plac8, S100a8, S100a9, Scd, Slc1a3, Slpi, Ucp1
inflammatory response	Adipoq, Cdo1, Hp, Lep, S100a8, S100a9, Slpi
concentration of lipid	Acly, Adipoq, Cfd, Cidec, Cyp2e1, Hp, Lep, Ntrk2, S100a8, S100a9, Scd, Thrsp, Ucp1

Table 61
Heat map of heart from gene expression profiling experiments mutants versus control mice. Blue/yellow color corresponds to transcriptional repression/induction in the mutant mice.
Mouse IDs: A=30299069, B=30299070, C=30299071

Mean	Mouse			Gene symbol	Gene name
ratio	A	B	C		
-1.72				Defb19	defensin beta 19
-1.72				Fmn2	formin 2
-1.79				Cib2	calcium and integrin binding family member 2
-1.82				Tkt	transketolase
-1.93				Slc1a3	solute carrier family 1, member 3
-2.03				Slc25a1	solute carrier family 25, member 1
-2.04				Otop1	otopetrin 1
-2.06				Cib2	calcium and integrin binding family member 2

Table 61
Heat map of heart.

Mean	Mouse			Gene symbol	Gene name
ratio	A	B	C		
-2.19				Acly	ATP citrate lyase
-2.2				S100a9	S100 calcium binding protein A9
-2.23				Aacs	acetoacetyl-CoA synthetase
-2.28				Cd52	CD52 antigen
-2.29				Plac8	placenta-specific 8
-2.4				S100a8	S100 calcium binding protein A8
-2.43				Aldh1a2	aldehyde dehydrogenase family 1, subfamily A2
-2.44				Ntrk2	neurotrophic tyrosine kinase, receptor, type 2
-2.44				Thrsp	thyroid hormone responsive
-2.44				Slc25a1	solute carrier family 25, member 1
-2.63				Glb1l2	galactosidase, beta 1-like 2
-2.77				BC054059	adipogenin
-2.88				Slpi	secretory leukocyte peptidase inhibitor
-3.21				Hp	haptoglobin
-3.23				Arxes2	adipocyte-related X-chromosome expressed sequence 2
-3.88				Hp	haptoglobin
-3.99				Lep	leptin
-4.31				Cyp2e1	cytochrome P450, family 2, subfamily e, polypeptide 1
-4.17				Cldn12	claudin 12
-4.41				Orm1	orosomucoid 1
-5.17				Hp	haptoglobin
-5.52				Cidec	cell death-inducing DFFA-like effector c
-5.61				Ucp1	uncoupling protein 1
-6.92				Cdo1	cysteine dioxygenase 1, cytosolic
-7.15				Tmem45b	transmembrane protein 45b
-7.53				Elovl6	ELOVL family member 6, elongation of long chain fatty acids
-10.99				Cfd	complement factor D (adipsin)
-10.77				Adipoq	adiponectin, C1Q and collagen domain containing
-10.13				Car3	carbonic anhydrase 3
-14				Scd1	stearoyl-Coenzyme A desaturase 1

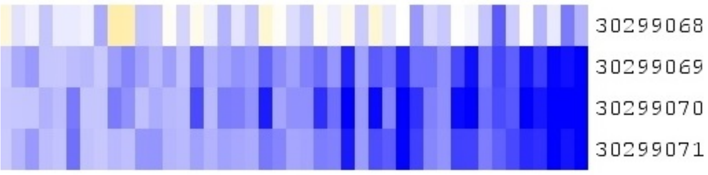

Figure 103
The heat map represent the differentially regulated genes in the mutant samples 30299069, 30299070, and 30299071 and display the differences in sample 30299068.

4.14.3 Discussion

The transcriptome profiling of the *Cldn12* mutant mice revealed moderate alterations in heart. The moderate differences on molecular level give evidence for alteration associated with lipid concentration and atherosclerosis. In addition, several of the regulated genes play roles in immunological processes.

4.14.4 Additional References

Churchill, G. A. (2002) Fundamentals of experimental design for cDNA microarrays. *Nature genetics* **32 Suppl**, 490-49

Drobyshev, A. L., Machka, C., Horsch, M., Seltmann, M., Liebscher, V., Hrabe de Angelis, M., and Beckers, J. (2003) Specificity assessment from fractionation experiments (SAFE): a novel method to evaluate microarray probe specificity based on hybridisation stringencies. *Nucleic Acids Res* **31**, E1-1

Oishi, K., Miyazaki, K., Kadota, K., Kikuno, R., Nagase, T., Atsumi, G., Ohkura, N., Azama, T., Mesaki, M., Yukimasa, S., Kobayashi, H., Iitaka, C., Umehara, T., Horikoshi, M., Kudo, T., Shimizu, Y., Yano, M., Monden, M., Machida, K., Matsuda, J., Horie, S., Todo, T., and Ishida, N. (2003) Genome-wide expression analysis of mouse liver reveals CLOCK-regulated circadian output genes. *The Journal of biological chemistry* **278**, 41519-41527

Pritchard, C. C., Hsu, L., Delrow, J., and Nelson, P. S. (2001) Project normal: defining normal variance in mouse gene expression. *Proceedings of the National Academy of Sciences of the United States of America* **98**, 13266-13271

Seltmann, M., Horsch, M., Drobyshev, A., Chen, Y., de Angelis, M. H., and Beckers, J. (2005) Assessment of a systematic expression profiling approach in ENU-induced mouse mutant lines. *Mammalian genome : official journal of the International Mammalian Genome Society* **16**, 1-10
